# Supplementary material for: Evaluation of de novo transcriptome assemblies from RNA-Seq data
Source: Genome Biol. 2014 Dec 21;15(12):553. doi: 10.1186/s13059-014-0553-5 (PMC4298084; doi:10.1186/s13059-014-0553-5)
Supplement: Additional file 1 — Supplementary methods, tables and figures. [file 13059_2014_553_MOESM1_ESM.pdf]

Evaluation of *de novo* transcriptome assemblies from RNA-Seq data (Supplementary Materials)

Bo Li Nathanael Fillmore Yongsheng Bai Mike Collins
James A. Thomson Ron Stewart Colin N. Dewey

1 Relationship between relative expression levels, read generating probabilities and expected read coverage

For simplicity, we assume that RNA-Seq reads are sequenced uniformly across the transcriptome. In addition, we only consider fixed length single-end RNA-Seq reads. We denote the read length as L. Given a set of M_T transcripts, we denote the relative expression levels as $\tau = (\tau_1, \tau_2, \ldots, \tau_{M_T})$, the read generating probabilities as $\Theta = (\theta_0, \theta_1, \ldots, \theta_{M_T})$ and the expected read coverage as $\Xi = (\xi_0, \xi_1, \xi_2, \ldots, \xi_{M_T})$. We further denote the lengths of transcripts as $\mathbf{L} = (l_0, l_1, \ldots, l_{M_T})$ and let $l_0 = L$. Here transcript 0 refers to a non-existing "noise" transcript. It represents all reads that are generated from the background noise.

According to [1],

$$\theta_i = (1 - \theta_0) \cdot \frac{\tau_i (l_i - L + 1)}{\sum_{j=1}^{M_T} \tau_j (l_j - L + 1)}, \qquad i > 0.$$

Then the expected read coverage of a transcript is defined as

$$\xi_j = \frac{N\theta_j}{l_j - L + 1}, \qquad j \geq 0.$$

It is easy to see that $\xi_j \propto \tau_j$ for $j > 0$.

Because the expected read coverage of a contig is defined as the expected read coverage of its parent transcript, the expected read coverage of the ith contig is

$$\lambda_i = \xi_{t(i)},$$

where $t(i)$ is contig i's parent transcript's index number and we define $t(0) = 0$.

2 The RSEM-EVAL model as an approximation of the "natural" model

2.1 The "natural" model

As described in the main text, Figure S1(a) shows a natural way of generating both the RNA-Seq data set and the assembly. We will refer to it as the "natural" model. In the "natural" model, we first generate the number of transcripts, M_T. Then a set of transcript sequences, T, and their relative expressions, τ, are generated. Given T and τ, we first generate the read generating probabilities, Θ and then use the RSEM model described in [1, 2] to generate a single-end RNA-Seq read data set. In the end, the "true" assembly A with overlap length $w = 0$ is constructed from the transcript sequences T with the help of hidden information that specifies the origin of each read (generated by the RSEM model).

For simplicity of presentation, we describe and depict in Figure S1(a) the basic RSEM model, which was introduced in [1]. In practice, RSEM-EVAL uses a fuller extended model as described in [2]. In the basic RSEM model, given the probabilities, Θ, of a read being generated from each of the possible transcripts, N reads are generated. Then for each read n, the transcript from which it is derived, G_n, its start position on that transcript, S_n, and its orientation, O_n, are generated. Finally, the read sequence, R_n, is generated based on its true alignment and a sequencing error model. In the RSEM model, only R_n is observed. G_n, S_n and O_n are all hidden variables. We denote all of these hidden variables by $H = (G, S, O)$.

The joint probability of an assembly and the RNA-Seq data in the "natural" model can be expressed as

$$P(A, D) = \sum_{M_T} P(M_T) \sum_{T,\tau,\Theta} P(T|M_T)P(\tau|M_T)P(\Theta|T, \tau) \sum_{H} P(H|\Theta)P(D|H, T)P(A|H, T).$$

2.2 Derivation of the RSEM-EVAL model from the "natural" model

Unfortunately, directly calculating $P(A, D)$ under the "natural" model is computationally infeasible because it requires us to sum over all possible transcript sets. Therefore, we use some approximations of the "natural" model so that we can calculate $P(A, D)$ more efficiently. We can rewrite the probability $P(A, D)$ as follows:

$$\begin{aligned} P(A, D) &= \int_{\Lambda} P(M, \Lambda, A, D)d\Lambda, \\ &= P(M) \int_{\Lambda} P(\Lambda|M)P(A|\Lambda)P(D|A, \Lambda)d\Lambda. \end{aligned}$$

In the "natural" model, $P(A|\Lambda)$ is hard to compute because the contigs are not conditionally independent given Λ. Similarly, $P(D|A, \Lambda)$ is also hard to compute. Therefore we make the following two approximations so that we can compute $P(A|\Lambda)$ and $P(D|A, \Lambda)$ efficiently:

1. We assume that given the expected read coverage, the contigs are generated independently, i.e.,
$$P(A|\Lambda) = \prod_{i=1}^{M} P(A_i|\lambda_i).$$

2. To calculate $P(D|A, \Lambda)$, we first ignore the dependency between RNA-Seq reads and treat the assembly as the true transcript set. That is, we calculate the RSEM likelihood:
$$P_{RSEM}(D|T = A, \Theta) = \prod_{i=1}^{N} P_{RSEM}(R_i|T = A, \Theta).$$
We then correct $P_{RSEM}(D|T = A, \Theta)$ by a term that takes into account the dependencies between the reads to obtain our approximation of $P(D|A, \Lambda)$. We will see later that this approximation is principled.

With these two approximations, we obtain the RSEM-EVAL model shown in Figure S1(b). Therefore, the RSEM-EVAL model can be viewed as an approximation to the "natural" model.

3 Derivation and calculation of the contig length distribution

Our goal is to define and calculate $P(\ell|\lambda)$ such that it closely matches the contig length distribution implied by the "natural" model.

3.1 A procedure to generate contig lengths that is closely related to the "natural" model

First, let us consider the following procedure (Procedure 1) for generating contig lengths with a given expected read coverage λ (We assume that the overlap length w is already given). This procedure is closely related to the "natural" model we mentioned before. We use this procedure to help us define the contig length distribution.

Procedure 1 Generation of contig lengths given a λ.

1) Sample one transcript length t from the transcript length distribution, $P(t)$.
2) Sample the number of reads generated from each of the $t-L+1$ valid positions in the transcript based on Poisson distributions parameterized with λ.
3) Merge any two reads that overlap at least w bases to construct "true" contigs.
return The contig lengths obtained from step 3).

Procedure 1 defines a conditional distribution over the tuple of transcript length, contig start position and contig length, $P(t, pos, \ell|\lambda)$. It represents the conditional probability of existing a contig of length ℓ, which starts at position pos (ranges from 0 to $t - L$) of a transcript with length t, given the expected read coverage λ. We can decompose $P(t, pos, \ell|\lambda)$ as

$$P(t, pos, \ell|\lambda) = P(t|\lambda)P(pos|t, \lambda)P(\ell|pos, t, \lambda). \tag{1}$$

We will discuss each term in the right hand side of (1) separately.

$P(t|\lambda)$ We assume that

$$P(t|\lambda) = P(t), \tag{2}$$

and t, the transcript length follows a negative binomial distribution,

$$t \sim NB(r, p), \quad P(t = k) = \begin{pmatrix} k + r - 1 \\ r - 1 \end{pmatrix} p^r (1 - p)^k, \quad k = 0, 1, \ldots$$

To justify our use of the negative binomial distribution here, we fit the empirical transcript length distribution obtained from real mouse Ensembl annotation with Poisson, geometric and negative binomial distributions. We found that the negative binomial distribution fits the empirical transcript length distribution the best (likelihood-ratio test, $p \approx 0$).

$P(pos|t, \lambda)$ Because the number of reads generated from a position in a transcript approximately follows a Poisson distribution with parameter λ, we use iid Poisson distributions to approximate the read generating process from a transcript. Furthermore, we do not distinguish between reads coming from the forward or reverse strands and let a read's start position be its leftmost position in the forward strand. We denote

$$p_\lambda = e^{-\lambda},$$

which is the probability of generating no reads from a position in the transcript under the Poisson assumptions.

In order to have a contig start at position pos, we need to make sure that

- There are no reads generated at the $\min(L - w, pos)$ positions before pos. Otherwise, these reads will merge with the segment at pos to form a contig starting before pos. The probability of this event is $p_\lambda^{\min(L-w,pos)}$.
- There is at least one read generated at position pos. The probability of this event is $1 - p_\lambda$.

Therefore we have

$$P(pos|t, \lambda) = p_\lambda^{\min(L-w,pos)}(1 - p_\lambda). \tag{3}$$

$P(\ell|pos, t, \lambda)$ Because the transcript must contain the contig, we must have $pos + \ell \leq t$. That means if $\ell > t - pos$, then $P(\ell|pos, t, \lambda) = 0$. Now let us assume that $\ell \leq t - pos$. In order to have a contig of length ℓ start at pos, we need to make sure that

- The reads starting at pos can be extended to a segment of length ℓ. This implies that there must be at least one read at position $pos + \ell - L$.

- No reads are generated at the $\min(L-w, t-pos-\ell)$ positions after position $pos+\ell-L$. Otherwise, the generated reads would merge with the segment to form a contig with a longer length. The probability of this event is $p_\lambda^{\min(L-w,t-pos-\ell)}$.

We denote the function $f_\lambda(\ell)$ as the probability of extending a read to a segment of length ℓ by merging reads to its right. $f_\lambda(\ell)$ can be calculated using the following recurrence:

$$f_\lambda(\ell) = \begin{cases} 0 & , \quad \ell < L \\ 1 & , \quad \ell = L \\ \sum_{j=w}^{L-1} f_\lambda(\ell-(L-j)) p_\lambda^{L-j-1}(1-p_\lambda) & , \quad \ell > L \end{cases} . \tag{4}$$

When $\ell > L$, we need at least one read to cover the last L bases, which explains $1-p_\lambda$. Because we need at least w bases of overlap to merge a segment and the read(s) at its end, any segment that overlaps the read(s) at its end by w to $L-1$ bases should be considered. For each possible overlap length j, $f_\lambda(\ell-(L-j))$ denotes the probability of having a segment that overlaps j bases with the read(s) at its end. p_λ^{L-j-1} guarantees that the overlap length will not exceed j.

$P(\ell|pos, t, \lambda)$ can be expressed in terms of $f_\lambda(\ell)$:

$$P(\ell|pos, t, \lambda) = \begin{cases} 0 & , \quad \ell > t-pos \\ f_\lambda(\ell) p_\lambda^{\min(L-w,t-pos-\ell)} & , \quad \ell \le t-pos \end{cases} . \tag{5}$$

Putting things together by plugging (2), (3) and (5) into (1), we can express $P(t, pos, \ell|\lambda)$ as

$$P(t, pos, \ell|\lambda) = \begin{cases} 0 & , \quad \ell > t-pos \\ P(t) f_\lambda(\ell)(1-p_\lambda) p_\lambda^{\min(L-w,pos)+\min(L-w,t-pos-\ell)} & , \quad \ell \le t-pos \end{cases} . \tag{6}$$

3.2 Defining the contig length distribution $P(\ell|\lambda)$

Given $P(t, pos, \ell|\lambda)$ defined in the last section, it is natural to define $P(\ell|\lambda)$ as

$$P(\ell|\lambda) = \sum_{t,pos} P(t, pos, \ell|\lambda). \tag{7}$$

However, the events (t, pos, ℓ) are not mutually exclusive because one pass of Procedure 1 can produce multiple contigs. Therefore, the definition in (7) will result in an invalid distribution for that $\sum_\ell P(\ell|\lambda) > 1$. Thus we have to define $P(\ell|\lambda)$ by an alternative procedure (Procedure 2):

In RSEM-EVAL, we define $P(\ell|\lambda)$ as the limit distribution of Procedure 2 when $N \to \infty$. By defining

$$c_\lambda(\ell) = \frac{\sum_t P(t) \sum_{pos=0}^{t-\ell} p_\lambda^{\min(L-w,pos)+\min(L-w,t-\ell-pos)}}{\sum_{t'} P(t') \sum_{pos'=0}^{t'-L} p_\lambda^{\min(L-w,pos')}}, \tag{8}$$

$P(\ell|\lambda)$ can be expressed as

$$P(\ell|\lambda) = c_\lambda(\ell) \cdot f_\lambda(\ell). \tag{9}$$

Procedure 2 Define $P(\ell|\lambda)$.

Initialize an empty bag.
repeat
1) Run one pass of Procedure 1.
2) Put all contigs produced from 1) into the bag.
until The number of iterations, N, is large enough
Define $P(\ell|\lambda)$ as the frequency of contigs with length ℓ in the bag.

If we set $N = M_t$ in Procedure 2 and let all transcripts' expected read coverage be λ, $P(\ell|\lambda)$ defined above is roughly equivalent to the probability of a randomly picked contig having length ℓ in an instance of the "natural" model. Below we provide a proof for the correctness of (9).

Proof: We define $X_{t,pos,\ell}$ as the indicator variable that there is a contig generated from position *pos* of a length t transcript with length ℓ in one iteration of Procedure 2. Then $E(X_{t,pos,\ell})$ is

$$E(X_{t,pos,\ell}) = P(X_{t,pos,\ell} = 1) = P(t)f_\lambda(\ell)(1-p_\lambda)p_\lambda^{\min(L-w,pos)+\min(L-w,t-pos-\ell)},$$

for all $\ell \leq t - pos$. We also denote $X_{t,pos} = \sum_\ell X_{t,pos,\ell}$ be the indicator variable that there is a contig generated from position *pos* of a length t transcript. It's easy to see that

$$E(X_{t,pos}) = P(t)p_\lambda^{\min(L-w,pos)}(1-p_\lambda).$$

Let X_ℓ be the number of contigs with length ℓ in one iteration and X_{tot} be the total number of contigs in one iteration. We have

$$\begin{aligned} X_\ell &= \sum_{t,pos} X_{t,pos,\ell}, \\ X_{tot} &= \sum_{t,pos} X_{t,pos}. \end{aligned}$$

Therefore by the property of expectation, we have

$$\begin{aligned} E(X_\ell) &= \sum_{t,pos} E(X_{t,pos,\ell}) = f_\lambda(\ell)(1-p_\lambda)\sum_t P(t)\sum_{pos=0}^{t-\ell} p_\lambda^{\min(L-w,pos)+\min(L-w,t-\ell-pos)}, \\ E(X_{tot}) &= \sum_{t,pos} E(X_{t,pos}) = (1-p_\lambda)\sum_t P(t)\sum_{pos=0}^{t-L} p_\lambda^{\min(L-w,pos)}. \end{aligned}$$

Now we define the sample mean of X_ℓ and X_{tot} as

$$\begin{aligned} \bar{X}_\ell &= \frac{1}{N}\sum_{i=1}^{N} X_\ell^{(i)}, \\ \bar{X}_{tot} &= \frac{1}{N}\sum_{i=1}^{N} X_{tot}^{(i)}, \end{aligned}$$

where $X_\ell^{(i)}$ and $X_{tot}^{(i)}$ are the corresponding indicator variables in the ith iteration of Procedure 2.

By the law of large numbers, when $N \to \infty$, the sample mean converges in probability to the expectation. Thus, we have

$$\bar{X}_\ell \xrightarrow{P} E(X_\ell) = f_\lambda(\ell)(1-p_\lambda)\sum_t P(t) \sum_{pos=0}^{t-\ell} p_\lambda^{\min(L-w,pos)+\min(L-w,t-\ell-pos)},$$

$$\bar{X}_{tot} \xrightarrow{P} E(X_{tot}) = (1-p_\lambda)\sum_t P(t) \sum_{pos=0}^{t-L} p_\lambda^{\min(L-w,pos)}.$$

Then according to Procedure 2,

$$\begin{aligned}
P(\ell|\lambda) &= \frac{\sum_{i=1}^N X_\ell^{(i)}}{\sum_{i=1}^N X_{tot}^{(i)}}, &(10)\\
&= \frac{\bar{X}_\ell}{\bar{X}_{tot}}, &(11)\\
&\xrightarrow{P} \frac{f_\lambda(\ell)(1-p_\lambda)\sum_t P(t)\sum_{pos=0}^{t-\ell} p_\lambda^{\min(L-w,pos)+\min(L-w,t-\ell-pos)}}{(1-p_\lambda)\sum_{t'} P(t')\sum_{pos'=0}^{t'-L} p_\lambda^{\min(L-w,pos')}}, &(12)\\
&= \frac{\sum_t P(t)\sum_{pos=0}^{t-\ell} p_\lambda^{\min(L-w,pos)+\min(L-w,t-\ell-pos)}}{\sum_{t'} P(t')\sum_{pos'=0}^{t'-L} p_\lambda^{\min(L-w,pos')}} f_\lambda(\ell), &(13)\\
&= c_\lambda(\ell)\cdot f_\lambda(\ell). &(14)
\end{aligned}$$

where (12) follows from (11) by Slutsky's theorem. ■

3.3 Practical considerations

In practice, transcript lengths are not likely to be available. However, we can estimate the transcript length distribution from a related species with a known transcript set. Because we do not estimate the distribution directly from the species whose transcriptome is sequenced, we want to make sure that the RSEM-EVAL score is not sensitive to the estimated transcript length distribution. Therefore, we conducted the following experiment.

Note that in (9), only $c_\lambda(\ell)$ involves the transcript length distribution. Thus, we experimented by removing $c_{\lambda_i}(\ell_i)$s from the RSEM-EVAL scores for the assemblies on the simulated mouse data set. We then calculated Spearman's rank correlation coefficients between the modified RSEM-EVAL scores and reference-based measures (Table S1). The results suggest that $c_{\lambda_i}(\ell_i)$ has little impact on the RSEM-EVAL score. Thus, the RSEM-EVAL score should not be sensitive to the estimated transcript length distribution.

For users' convenience, RSEM-EVAL provides a script (`rsem-eval-estimate-transcript-length-distribution`) to estimate the negative binomial parameters from a given transcript set.

4 Calculation of the likelihood term

As noted in the main text, the likelihood term can be written as

$$P(D|A, \Lambda_{MLE}) = \frac{P_{RSEM}(D|T = A, \Theta^c_{MLE})}{P_{RSEM}(C = 1|T = A, \Theta^c_{MLE})}, \tag{15}$$

where Θ^c_{MLE} is the contig-level read generating probabilities converted from Λ_{MLE} by

$$\theta^c_{MLE,i} = \frac{\lambda_{MLE,i}(\ell_i - L + 1)}{\sum_{j=0}^{M} \lambda_{MLE,j}(\ell_j - L + 1)}.$$

We approximate the likelihood correction term (denominator) by the following procedure:

$$P_{RSEM}(C = 1|T = A, \Theta^c_{MLE}) \approx \prod_{i=1}^{M} P(C_i = 1|a_i, \lambda'_i),$$
$$P(C_i = 1|a_i, \lambda'_i) \approx (1 - p_{\lambda'_i}) f_{\lambda'_i}(\ell_i),$$
$$\lambda'_i = \frac{N\theta^c_{MLE,i}}{\ell_i - L + 1}.$$

Here we decompose the probability of covering the assembly into the products of probabilities of covering each contig. The probability of covering a contig is further approximated using the Poisson assumptions we used in calculating the contig length distribution. In order to cover a contig, we need first generate reads from the leftmost position of the contig and then extend these read(s) to the end. The $p_{\lambda'_i}$ and $f_{\lambda'_i}(\ell_i)$ are defined the same as before, except that we replace the transcript-level expected read coverage, λ, with the contig-level expected read coverage, λ'.

4.1 Justification of RSEM-EVAL's likelihood correction term

A slightly modified "natural" model. Our justification is based on a slightly modified "natural" model: given the true transcripts and their expression levels, we first generate the number of reads starting from each position on each transcript. Let n'_{ij} be the number of reads generated at the jth position (forward strand) of transcript i. We assume that the n'_{ij}s are generated independently and that

$$n'_{ij} \sim Poisson(\xi_i),$$

where ξ_i is the expected read coverage of the ith transcript. We denote by $\mathcal{T} = \{n'_{ij}\}$ the set of the number of reads generated from each position. Note that n'_{ij} indicates location information but not order information (e.g., which reads are generated at the jth position of transcript i). Thus, the next step is to recover the read generating order information. The probability of any possible read generating order is

$$\frac{\prod_{ij} n'_{ij}!}{(\sum_{ij} n'_{ij})!},$$

which is the inverse of the multinomial coefficient. Thus far, we know each read's original transcript and location in the forward strand. In the next step, we generate each read's orientation and read sequence, which is similar to the "natural" model. Lastly, the assembly is constructed based on $\mathcal{T}$ and the transcript sequences.

Represent $\mathcal{T}$ by contig positions. We can further decompose $\mathcal{T}$ as

$$\mathcal{T} = \{\mathcal{C}, \mathcal{P}\}, \quad \mathcal{C} = \{n_{ij}\} \text{ and } \mathcal{P} = \{t(\cdot), pos(t(\cdot), \cdot)\}.$$

where n_{ij} is the number of reads generated at the jth position of the ith contig in the assembly. $t(i)$ maps the ith contig to the index of its parent transcript. $pos(t(i), j)$ maps position j in contig i to the corresponding position in transcript $t(i)$. Then we have

$$n_{ij} = n'_{t(i),pos(t(i),j)}, \text{ and } \lambda_i = \xi_{t(i)}.$$

In addition, $\mathcal{C}$ collects all positions in the transcript set that contain a positive number of reads, i.e., $n_{ij} > 0$.

Derivation of the likelihood correction term. The probability of generating an assembly count vector, $\mathcal{C}$, given the assembly, the assembly expected read coverage, the true transcripts, the true transcript expected read coverage and the location of each contig in the assembly ($\mathcal{P}$) is

$$\begin{aligned}
P(\mathcal{C}|\mathcal{P}, A, \Lambda, T, \Xi) &= \frac{P(\mathcal{C}, \mathcal{P}, A, \Lambda|T, \Xi)}{P(\mathcal{P}, A, \Lambda|T, \Xi)}, &(16)\\
&= \frac{P(\mathcal{C}, \mathcal{P}, A, \Lambda|T, \Xi)}{\sum_{\mathcal{C}'} P(\mathcal{C}', \mathcal{P}, A, \Lambda|T, \Xi)}, &(17)\\
&= \frac{\prod_{i=1}^{M} \prod_{j=0}^{\ell_i - L} \frac{\lambda_i^{n_{ij}}}{n_{ij}!} e^{-\lambda_i}}{\prod_{i=1}^{M} (1 - p_{\lambda_i}) f_{\lambda_i}(\ell_i)}, &(18)
\end{aligned}$$

provided that $P(\mathcal{P}, A, \Lambda|T, \Xi) > 0$.

For all $\mathcal{T}' = \{\mathcal{C}', P\}$ compatible with A, $\mathcal{T}'$ must share the same set of positions that do not generate any read with $\mathcal{T}$. As a consequence, the probabilities at positions generating no reads are the same for both the numerator and denominator of (17). Therefore, these probabilities cancel out and we obtain (18).

If we define

$$g(\mathcal{C}, \Lambda) = \prod_{i=1}^{M} \prod_{j=0}^{\ell_i - L} \frac{\lambda_i^{n_{ij}}}{n_{ij}!} e^{-\lambda_i},$$

then

$$\begin{aligned}
P(D|A, \Lambda) &= \sum_{\mathcal{C}} P(D|\mathcal{C}) P(\mathcal{C}|A, \Lambda), &(19)\\
&= \sum_{\mathcal{C}} P(D|\mathcal{C}) \sum_{\mathcal{P}, T, \Xi} P(\mathcal{C}, \mathcal{P}, T, \Xi|A, \Lambda), &(20)\\
&= \sum_{\mathcal{C}} P(D|\mathcal{C}) \sum_{\mathcal{P}, T, \Xi} P(\mathcal{P}, T, \Xi|A, \Lambda) P(\mathcal{C}|\mathcal{P}, A, \Lambda, T, \Xi), &(21)\\
&= \sum_{\mathcal{C}} P(D|\mathcal{C}) \frac{g(\mathcal{C}, \Lambda)}{\prod_{i=1}^{M} (1 - p_{\lambda_i}) f_{\lambda_i}(\ell_i)} \sum_{\mathcal{P}, T, \Xi} P(\mathcal{P}, T, \Xi|A, \Lambda), &(22)\\
&= \frac{\sum_{\mathcal{C}} P(D|\mathcal{C}) g(\mathcal{C}, \Lambda)}{\prod_{i=1}^{M} (1 - p_{\lambda_i}) f_{\lambda_i}(\ell_i)}. &(23)
\end{aligned}$$

(22) follows from (21) for the reason that $P(\mathcal{P}, T, \Xi|A, \Lambda) > 0 \Leftrightarrow P(\mathcal{P}, A, \Lambda|T, \Xi) > 0$. The denominator in (23) is the likelihood correction term.

Calculation of the numerator. The numerator in (23), $\sum_{\mathcal{C}} P(D|\mathcal{C})g(\mathcal{C}, \Lambda)$, assumes that reads can only be generated from transcript positions that are part of a contig. This is roughly equivalent to calculating the likelihood only from positions within a contig under the "natural" model. Suppose that the nth read comes from contig i. Although we cannot determine G_n and S_n for this read in the "natural" model, we still know that

$$P(G_n, S_n) = \frac{\lambda_i}{N}.$$

Therefore we can calculate the equivalent part of $\sum_{\mathcal{C}} P(D|\mathcal{C})g(\mathcal{C}, \Lambda)$ in the "natural" model.

Lastly, in RSEM-EVAL, we use λ'_i instead of λ_i in the calculation. The relationship between λ'_i and λ_i is

$$\lambda'_i = \frac{N}{\sum_{j=0}^{M} \lambda_j(\ell_j - L + 1)}\lambda_i.$$

5 Contig impact score

In this section, we will derive RSEM-EVAL's contig impact score.

5.1 Decomposition of the RSEM log likelihood term

First, let us look at $\log P_{RSEM}(D|T = A, \Theta^c_{MLE})$, the log transform of the numerator of (15). For simplicity, we assume that this term follows the basic RSEM model [1] and denote by Z_{nijk} the indicator random variable summarizing the hidden information for read n, where $Z_{nijk} = 1$ if $(G_n, S_n, O_n) = (i, j, k)$. We use Z to summarize all indicator variables, $Z = \{Z_{nijk}\}$. We reorganize $\log P_{RSEM}(D|T = A, \Theta^c_{MLE})$ as

$$\begin{aligned}
\log P_{RSEM}(D|T = A, \Theta^c_{MLE}) &= \sum_{n=1}^{N} \log P_{RSEM}(r_n|\Theta^c_{MLE}), \\
&= \sum_{n=1}^{N}\sum_{i,j,k} P_{RSEM}(Z_{nijk} = 1|\Theta^c_{MLE}, r_n) \log \frac{P_{RSEM}(Z_{nijk} = 1, r_n|\Theta^c_{MLE})}{P_{RSEM}(Z_{nijk} = 1|\Theta^c_{MLE}, r_n)}, \\
&= \sum_{n=1}^{N}\sum_{i,j,k} P_{RSEM}(Z_{nijk} = 1|\Theta^c_{MLE}, r_n) \log P_{RSEM}(Z_{nijk} = 1, r_n|\Theta^c_{MLE}) \\
&\quad -\sum_{n=1}^{N}\sum_{i,j,k} P_{RSEM}(Z_{nijk} = 1|\Theta^c_{MLE}, r_n) \log P_{RSEM}(Z_{nijk} = 1|\Theta^c_{MLE}, r_n), \\
&= \sum_{i=0}^{M} lik_i - H(Z|D, \Theta^c_{MLE}),
\end{aligned}$$

where lik_i is the expected complete likelihood for contig i,

$$lik_i = \sum_{n,j,k} P_{RSEM}(Z_{nijk} = 1 | \Theta^c_{MLE}, r_n) \log P_{RSEM}(Z_{nijk} = 1, r_n | \Theta^c_{MLE}),$$

and $H(Z|D, \Theta^c_{MLE})$ is the posterior entropy of Z.

5.2 Decomposition of the RSEM-EVAL score

Because the assembly prior, BIC, and likelihood correction terms can also be decomposed into contig components, we can rewrite $\log P(A, D)$ as

$$\log P(A, D) = (lik_0 - \frac{1}{2} \log N) + \sum_{i=1}^{M} score_i - H(Z|D, \Theta^c_{MLE}),$$

where $score_i$ is defined as

$$score_i = \log P(\ell_i, s_i | \lambda_{MLE,i}) + lik_i - \log P(C_i = 1 | \ell_i, s_i, \lambda^{'}_i) - \frac{1}{2} \log N.$$

5.3 RSEM-EVAL's contig impact score

We denote the contig impact score for contig i as b_i. It is defined as the log of the ratio between two hypotheses. The first hypothesis is that contig i is real. The second (null) hypothesis is that the reads composing contig i are actually from the background noise (i.e., contig i is not real). In order to avoid the expected reads from contig i being assigned to other isoforms, in the null hypothesis, we fix the posterior probability $P(Z|D, \Theta^c_{MLE})$ and only replace lik_i in $\log P(A, D)$ by $lik^{'}_i$,

$$lik^{'}_i = \sum_{n,j,k} P_{RSEM}(Z_{nijk} = 1 | \Theta^c_{MLE}, r_n) \log P_{RSEM}(Z_{n0} = 1, r_n | \Theta^c_{MLE}),$$

where $Z_{n0} = 1$ means that read n is from the background noise.

Thus the contig impact score, b_i, for contig i, becomes

$$\begin{aligned} b_i \quad = \quad & \log P(\ell_i, s_i | \lambda_{MLE,i}) - \log P(C_i = 1 | \ell_i, s_i, \lambda^{'}_i) - \frac{1}{2} \log N \\ & + \sum_{n,j,k} P_{RSEM}(Z_{nijk} = 1 | \Theta^c_{MLE}, r_n) \log \frac{P_{RSEM}(Z_{nijk} = 1, r_n | \Theta^c_{MLE})}{P_{RSEM}(Z_{n0} = 1, r_n | \Theta^c_{MLE})}. \end{aligned}$$

6 Definition of contig (or scaffold) precision, recall, and F_1

In this subsection, we provide detailed definitions of the contig precision, recall, and F_1. The same definitions apply in the case of scaffolds, yielding scaffold precision, recall, and F_1, assuming

that each scaffold is represented as one or more contigs joined by an appropriate number of `N`'s. Throughout, A denotes the assembly, and B denotes the reference. Both A and B are thought of as sets of sequences. As discussed in the main text, the reference can be either an estimate of the "true" assembly or a collection of full-length reference transcripts. In this paper, we have used an estimate of the "true" assembly as the reference.

First, the contig (scaffold) recall is defined as follows:

- Align the assembly A to the reference B, using Blat. We use default settings for Blat, except that we require 80% identity (under Blat's definition of percent identity), instead of the default 90% identity, in order to generate more candidate alignments.
- Throw out alignments that are to the reverse strand, if in strand-specific mode.
- Throw out alignments whose fraction identity (defined below) is less than a parameter, the minimum fraction identity (0.99 in this paper).
- Throw out alignments whose fraction indel (defined below) is greater than a parameter, the maximum fraction indel (0.01 in this paper).
- Construct a bipartite graph from the remaining alignments, in which there is an edge between $a \in A$ and $b \in B$ iff there is a remaining alignment l of a to b.
- The contig (scaffold) recall is the number of edges in the maximum cardinality matching of this graph, divided by the number of sequences in the reference B.

The contig (scaffold) precision is defined as follows: interchange the assembly and the reference, and compute the contig (scaffold) recall. The contig (scaffold) F_1 is the harmonic mean of the precision and recall.

The fraction identity of an alignment l from a to b is defined as $\min(x/y, x/z)$, where

- x is the number of non-`N` bases in a that are aligned to an identical base in b, according to l,
- y is the number of non-`N` bases in a, and
- z is the number of non-`N` bases in b.

The fraction indel of an alignment l from a to b is defined as $\max(w/y, x/z)$, where

- w is the number of bases that are inserted in a, according to l (Blat's "Q gap bases"),
- x is the number of bases that are inserted in b, according to l (Blat's "T gap bases"),
- y is the number of non-`N` bases in a, and
- z is the number of non-`N` bases in b.

7 Definition of nucleotide precision, recall, and F_1

In this subsection, we provide detailed definitions of the nucleotide precision, recall, and F_1. The assembly A and reference B are as described in the previous subsection.

First, the nucleotide recall is defined as follows:

- Align the assembly A to the reference B, using Blat. We use default settings for Blat, except that we require 80% identity (under Blat's definition of percent identity), instead of the default 90% identity, in order to generate more candidate alignments.
- Throw out alignments that are to the reverse strand, if in strand-specific mode.
- Throw out alignments that are shorter than a parameter, the minimum fragment length. (In this paper, we have used the read length as the minimum fragment length.)
- Add each remaining alignment to a priority queue, with priority equal to the number of identical non-`N` bases in the alignment.
- Let numer = 0.
- While the priority queue is not empty:
  - Pop the alignment l with highest priority.
  - Add the number of identical non-`N` bases in the alignment to numer.
  - Subtract l from all the other alignments in the queue and update their priorities (see below).
- Let denom be the total number of non-`N` bases in the reference B.
- The unweighted nucleotide recall is numer/denom.

The actual implementation uses a more complicated and efficient algorithm than the one above.

The nucleotide precision is defined as follows: interchange the assembly and the reference, and compute the nucleotide recall. The nucleotide F_1 is the harmonic mean of the precision and recall.

Alignment subtraction, used in the definition of the nucleotide recall, is defined as follows:

- An alignment l from a to b can be thought of as a set of pairs of disjoint intervals

  $$\{([s_1(a), e_1(a)], [s_1(b), e_1(b)]), \ldots, ([s_n(a), e_n(a)], [s_n(b), e_n(b)])\},$$

  where each pair $([s_i(a), e_i(a)], [s_i(b), e_i(b)])$ corresponds to an ungapped segment of the alignment: $s_i(a)$ and $e_i(a)$ are the segment's start and end positions within a, and $s_i(b)$ and $e_i(b)$ are the segment's start and end positions within b. In the case of non-strand-specific alignments, $s_i(b)$ might be greater than $e_i(b)$.
- If l is an alignment from a to b, l' is an alignment from a' to b', $a \neq a'$, and $b \neq b'$, then the difference $l - l' = l$.

- If l is an alignment from a to b, l' is an alignment from a' to b', $a = a'$, and $b \neq b'$, then the difference $l - l' = l''$, defined as follows. Each alignment segment of l is compared to the alignment segments of l'. If a segment of l overlaps one of the segments of l' wrt a, it is truncated so as to avoid the overlap. This truncation may result in zero, one, or two replacement alignment segments. (If the overlapping alignment segment of l' is contained strictly within the segment of l, wrt a, two segments will result.)
- If l is an alignment from a to b, l' is an alignment from a' to b', $a \neq a'$, and $b = b'$, then the difference $l - l' = l''$, defined similarly as in the previous item, except overlaps are examined and resolved wrt b.

8 The relationship between the KC and RSEM-EVAL scores

In this section we explain the mathematical relationship between the KC and RSEM-EVAL scores. To do so, we first present another reference-based measure, the *expected description length*, which has an even closer relationship to the RSEM-EVAL score. We then show how maximizing the KC score is equivalent to maximizing a simplified version of the expected description length measure.

8.1 Expected description length as a referenced-based measure

A transcriptome is defined as the set of transcripts and their abundances. Therefore it is natural to define the ground truth as a set of true transcripts and their relative abundances. We denote the ground truth as (T, τ), where T is the set of transcripts, τ represents each transcript's relative abundance. Given a k-mer size k, the ground truth induces a distribution over all possible k-mers. We denote this distribution as $p(x)$.

Having $p(x)$, we can generate any amount of k-mers from the ground truth. Let D denote the set of k-mers generated. For simplicity, we assume that the throughput (number of bases generated) is fixed (e.g., $|D|$ is fixed). Then the number of reads $N = \frac{|D|}{k}$.

Suppose we want to select a model from a family of models having the form $(A, \hat{\tau})$, where A is a set of sequences and $\hat{\tau}$ is a set of associated relative abundances. Because A is an assembly, we require that every sequence's relative abundance is positive. In addition, we also introduce a $\hat{\tau}_0$ in $\hat{\tau}$, which represents the probability of generating k-mers from the background noise. The probability of generating any k-mer is the same under the background noise. Thus $|\hat{\tau}| = M + 1$, where M is the number of sequences in A. Each $(A, \hat{\tau})$ also induces a distribution over k-mers and we denote it as $q(x)$.

Given any instance of D, we can evaluate each candidate model $(A, \hat{\tau})$ by its description length [3]. The description length of a model is the number of bits required to compress both the data D and the model itself $(A, \hat{\tau})$. We assume the number of sequences, M, follows distribution $P(M)$ and the length of a sequence l follows a distribution of $P(l)$.

To encode a model, we need to encode the number of sequences, M, with $-\log_2 P(M)$ bits. Then we need to encode the length of each sequence with a total of $-\sum_{i=1}^{M} \log_2 P(l_i)$ bits. After that, we need $-\log_2 \frac{1}{4}|A| = 2|A|$ bits to encode all of the nucleotides in A, where $|A|$ refers to the total

number of bases in A. Lastly, we need at least $\frac{M}{2}\log_2 N$ bits to encode $\hat{\tau}$ [4] since we have M free parameters. Given the model, we use $-\log_2 q(x)$ bits to encode a k-mer x. Thus the description length of $(A, \hat{\tau})$ is

$$f_{DL}(A,\hat{\tau}) = \underbrace{\left(\sum_{x\in D} -\log_2 q(x)\right)}_{\text{bits to encode the data}} + \underbrace{\left(-\log_2 P(M) - \sum_{i=1}^{M}\log_2 P(l_i) + 2|A| + \frac{M}{2}\log_2 N\right)}_{\text{bits to encode the model}}. \tag{24}$$

Finally, our new reference-based measure is defined by taking expectation over D:

$$f_{EDL}(A,\hat{\tau}) = E[f_{DL}(A,\hat{\tau})] = N\cdot H(p,q) + 2|A| + g(A,\hat{\tau}), \tag{25}$$

where $H(p,q)$ is the cross entropy and defined as

$$H(p,q) = \sum_x -p(x)\log_2 q(x),$$

and $g(A,\hat{\tau})$ is defined as

$$g(A,\hat{\tau}) = -\log_2 P(M) - \sum_{i=1}^{M}\log_2 P(l_i) + \frac{M}{2}\log_2 N.$$

8.2 Interpretation of the RSEM-EVAL score as a description length

We can write $-\log P(A,D)$ as (see equation 2 in the main text)

$$-\log P(A,D) \approx -\underbrace{\log P(A|\Lambda_{MLE})}_{\text{assembly prior}} - \underbrace{\log P(D|A,\Lambda_{MLE})}_{\text{likelihood}} + \underbrace{\frac{1}{2}(M+1)\log N}_{\text{BIC term}}.$$

In the above equation, the assembly prior term encodes the assembly A, the BIC term encodes the parameters Λ_{MLE} and the likelihood term encode the data given the model. Thus we can interpret the RSEM-EVAL score as a description length in a better designed system (e.g., we require that the data covers the assembly).

8.3 From expected description length to KC score

Let us focus on $N\cdot H(p,q)$:

$$\begin{aligned}
N\cdot H(p,q) &= \frac{|D|}{k}\sum_{x\in T\wedge x\notin A} p(x)\left(-\log_2\left(\frac{1}{4}\right)^k\right) - N\sum_{x\notin T\vee x\in A} p(x)\log_2 q(x)\\
&= \frac{|D|}{k}\sum_{x\in T\wedge x\notin A} p(x)2k - N\sum_{x\notin T\vee x\in A} p(x)\log_2 q(x)\\
&= 2|D|\sum_{x\in T\wedge x\notin A} p(x) - N\sum_{x\notin T\vee x\in A} p(x)\log_2 q(x)\\
&= 2|D|(1-\sum_{x\in T\wedge x\in A} p(x)) - N\sum_{x\notin T\vee x\in A} p(x)\log_2 q(x),
\end{aligned}$$

where $x \in A$ means that the k-mer x is present in the sequences of A (not from background noise).

In the real size RNA-Seq data sets we tested, it appears that $2|D|(1 - \sum_{x \in T \wedge x \in A} p(x))$ dominates $N \cdot H(p, q)$ and $2|A|$ dominates the bits used to encode the model in the expected description length. Therefore we have

$$\begin{aligned} f_{EDL}(A, \hat{\tau}) &\approx 2|D|(1 - \sum_{x \in T \wedge x \in A} p(x)) + 2|A| \\ &= 2|D|(1 - (\sum_{x \in T \wedge x \in A} p(x) - \frac{|A|}{|D|})) \\ &= 2|D|(1 - \text{score}_{\text{KC}}(A)). \end{aligned}$$

Thus maximizing KC score is roughly equivalent to minimizing the expected description length.

9 Experimental details for the axolotl assembly

To generate Figure 6 in the main text, we used the following procedure to build a one-to-one mapping between the axolotl contigs and frog protein sequences. First, we aligned the axolotl contigs to the frog protein sequences using BLASTX and kept only those alignments with e-value < 1e-5. Then a one-to-one mapping was determined using reciprocal alignments. Reciprocal alignments were defined as those alignments that are best with respect to both the axolotl contig and frog protein sequence. Here, "best" means that an alignment has the largest number of axolotl bases aligned to a frog protein and vice versa.

References

[1] Li, B., Ruotti, V., Stewart, R.M., Thomson, J.A., Dewey, C.N.: RNA-Seq gene expression estimation with read mapping uncertainty. Bioinformatics **26**(4), 493–500 (2010)

[2] Li, B., Dewey, C.N.: RSEM: Accurate transcript quantification from RNA-Seq data with or without a reference genome. BMC Bioinformatics **12**, 323 (2011)

[3] Rissanen, J.: Modeling by shortest data description. Automatica **14**, 465–471 (1978)

[4] Rissanen, J.: A universal prior for integers and estimation by minimum description length. Annals of Statistics **11**(2), 416–431 (1983)

[5] Stewart, R., Rascon, C.A., Tian, S., Nie, J., Barry, C., Chu, L.F., Ardalani, H., Wagner, R.J., Probasco, M.D., Bolin, J.M., Leng, N., Sengupta, S., Volkmer, M., Habermann, B., Tanaka, E.M., Thomson, J.A., Dewey, C.N.: Comparative RNA-seq analysis in the unsequenced axolotl: The oncogene burst highlights early gene expression in the blastema. PLoS Computational Biology **9**(3), 1002936 (2013)

[6] Grabherr, M.G., Haas, B.J., Yassour, M., Levin, J.Z., Thompson, D.A., Amit, I., Adiconis, X., Fan, L., Raychowdhury, R., Zeng, Q., Chen, Z., Mauceli, E., Hacohen, N., Gnirke, A., Rhind,

N., di Palma, F., Birren, B.W., Nusbaum, C., Lindblad-Toh, K., Friedman, N., Regev, A.: Full-length transcriptome assembly from RNA-Seq data without a reference genome. Nature Biotechnology **29**(7), 644–652 (2011)

Supplementary tables

	Nucleotide-level F_1	Contig-level F_1	Novel measure
Before	0.92	0.70	0.98
After	0.92	0.70	0.98

Table S1: Spearman's rank correlation coefficients between the RSEM-EVAL score w/o the transcript length distribution related term and reference-based measures. No difference is observed.

		Median difference in			
	Number of contigs	Nucleotide-level F_1	Contig-level F_1	KC score	RSEM-EVAL score
Trinity	**142**	**2.76e-4**	**2.81e-5**	**1.13e-4**	**291234.82**
Oases	**22043**	**1.19e-1**	**2.80e-3**	**1.53e-2**	**38941405.71**
SOAPdenovo-Trans	**703**	**-3.59e-4**	**4.06e-5**	**6.83e-7**	**39309.76**
Trans-ABySS	142491	7.10e-2	6.95e-3	8.31e-3	21906624.47

Table S2: Effect of trimming contigs with negative contig score for assemblies. Median difference is the median of the difference of the trimmed measure and the untrimmed measure. Bold values indicate that the estimates are significantly ($P < 0.05$) more accurate, as assessed by a paired Wilcoxon signed rank test. The median values for Trans-ABySS are not significant because there is only one Trans-ABySS assembly. The median numbers of contigs that are trimmed is largest for Oases and Trans-ABySS assemblies. The median improvement of the trimmed assemblies over untrimmed assemblies is also largest, for all evaluation measures, for these two assemblers.

	[5] assembly	RSEM-EVAL-guided assembly
Number of contigs	113,925	173,130
Total length of assembly	71,027,573	121,949,539
Minimum contig length	40	201
Maximum contig length	7,943	18,756
Mean contig length	623	704
First quartile	423	254
Second quartile (median)	500	366
Third quartile	700	720
95th Percentile	1,356	2,456
Standard Deviation	414	887
N50	650	1,200

Table S3: Comparison of the assembly statistics between the published axolotl assembly and the new RSEM-EVAL-guided assembly.

Supplementary figures

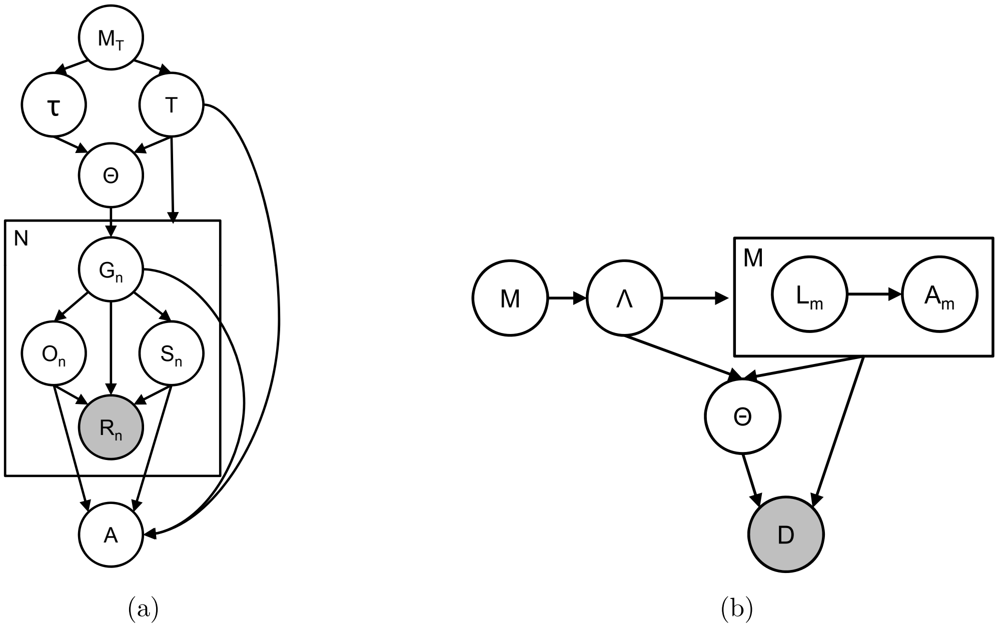

Figure S1: Generative probabilistic models for RSEM-EVAL. (a) The "natural" model, which represents a natural definition of the process of generating both the RNA-Seq reads and their "true" assembly. In this model, we first generate the true transcript sequences and their relative expression levels. Then we use the RSEM model [1, 2] to generate an RNA-Seq data set. Lastly, the "true" assembly is defined based on the generated transcript sequences and hidden information from RNA-Seq reads. (b) The model used by RSEM-EVAL, which approximates the "natural" model to enable more efficient inference. In this model, we first generate an assembly and then generate a set of RNA-Seq reads given the assembly. To generate an assembly, each contig is assumed to be generated independently. To generate a contig m, its sequence length L_m is first picked and then its sequence A_m is generated given its length.

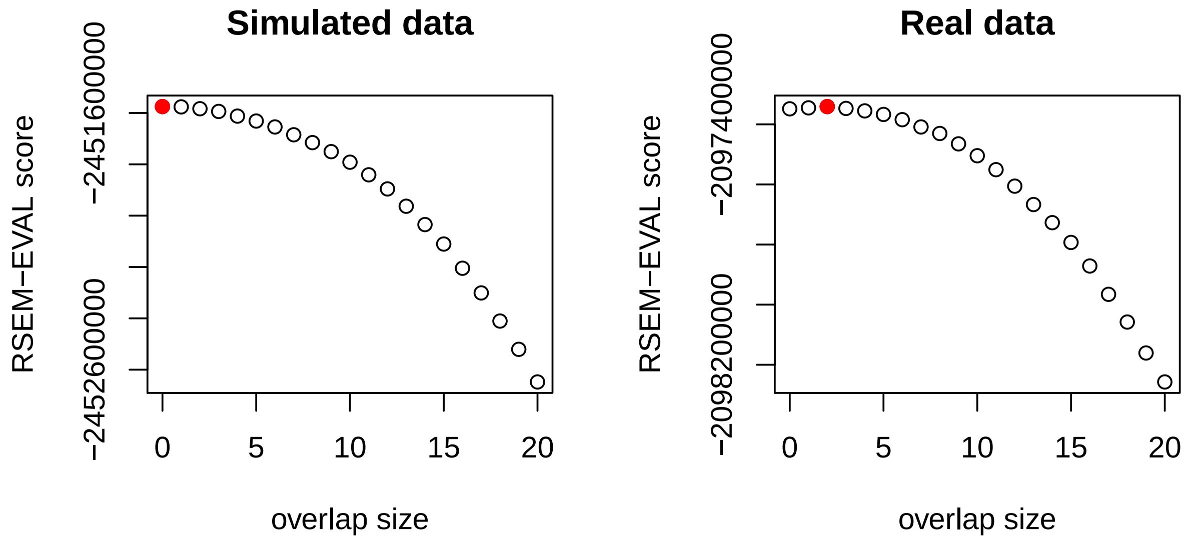

Figure S2: RSEM-EVAL scores of "true" assemblies for the local regions around the maximal values on both simulated and real data sets. The maximum values (red circles) are achieved at $w = 0$ and $w = 2$ in a left-right order.

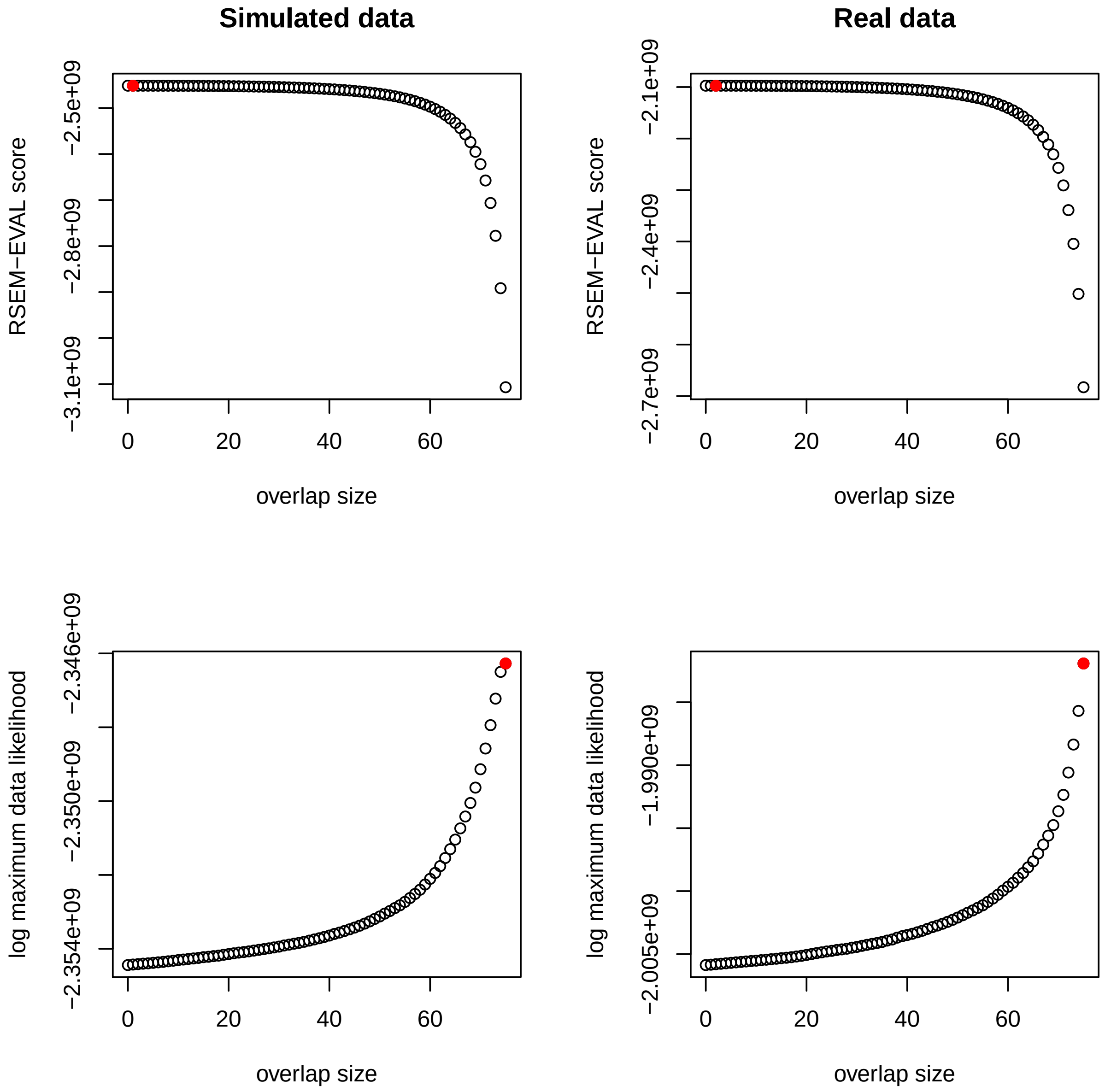

Figure S3: RSEM-EVAL scores with overlap length parameter set as 50 (top row) and ML (bottom row) scores of "true" assemblies for different values of the minimum overlap length on both simulated (left column) and real (right column) data sets. The maximum values (red circles) are achieved at $w = 1$, $w = 2$, $w = 75$ and $w = 75$ in a top-down, left-right order. For better visualization of the maximizing values of w, RSEM-EVAL scores for the local regions around the maximal values are shown in Figure S4.

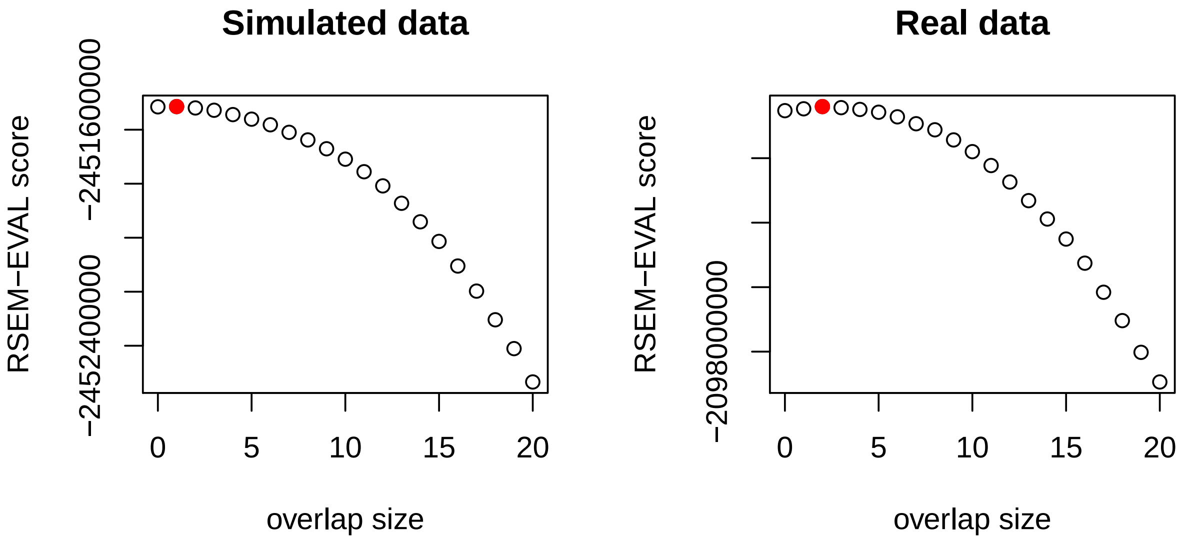

Figure S4: RSEM-EVAL scores (with overlap length parameter set as 50) of “true” assemblies for the local regions around the maximal values on both simulated and real data sets. The maximum values (red circles) are achieved at $w = 1$ and $w = 2$ in a left-right order.

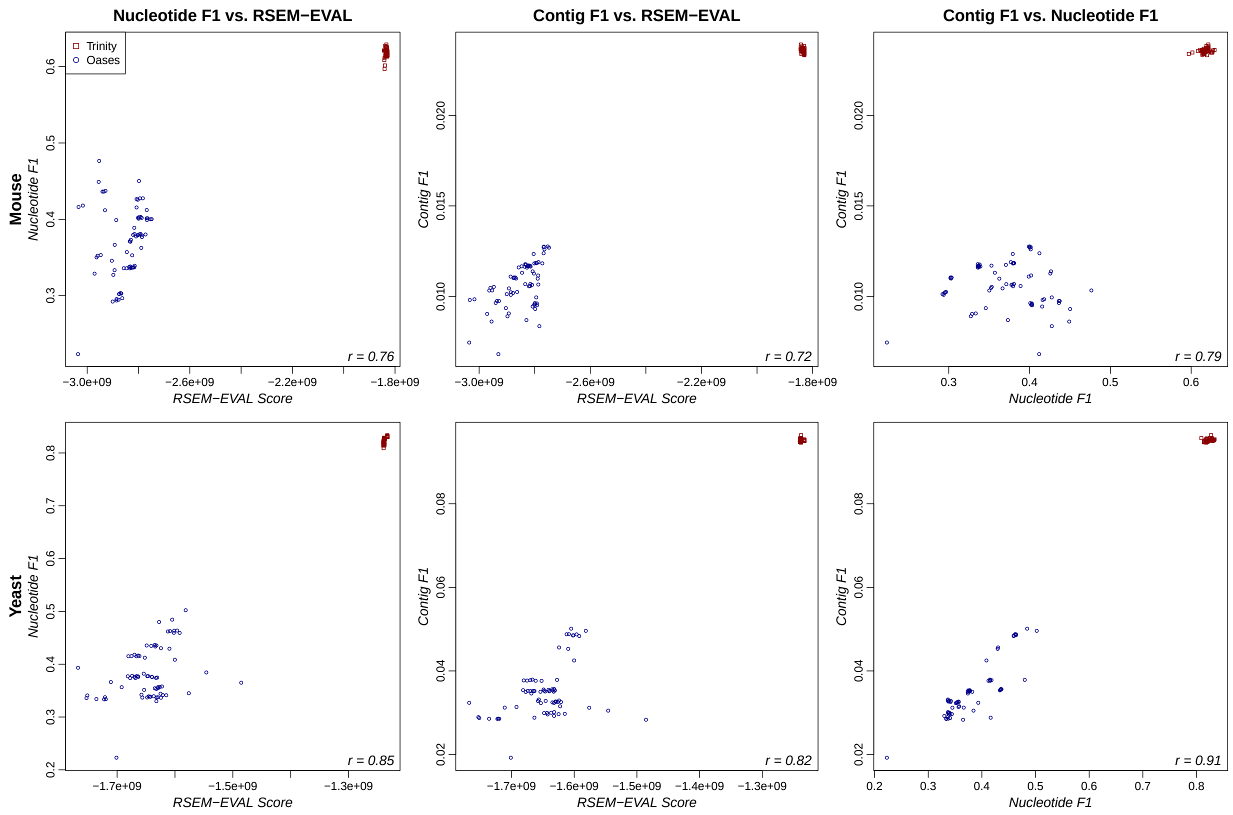

Figure S5: Correlation of the RSEM-EVAL score with reference-based measures on the strand-specific data sets [6]. Scatterplots are shown for the real mouse (top row) and real yeast (bottom row) data sets and for both the nucleotide-level F_1 (left column) and contig-level F_1 (center column) measures. For comparison, scatterplots of the nucleotide-level F_1 against the contig-level F_1 are shown (right column). Because the versions of SOAPdenovo-Trans and Trans-ABySS we used did not take into account strand-specificity, they were not run on the two strand-specific data sets. The Spearman rank correlation coefficient (bottom-right corner of each plot) was computed for each combination of data set and reference-based measure.

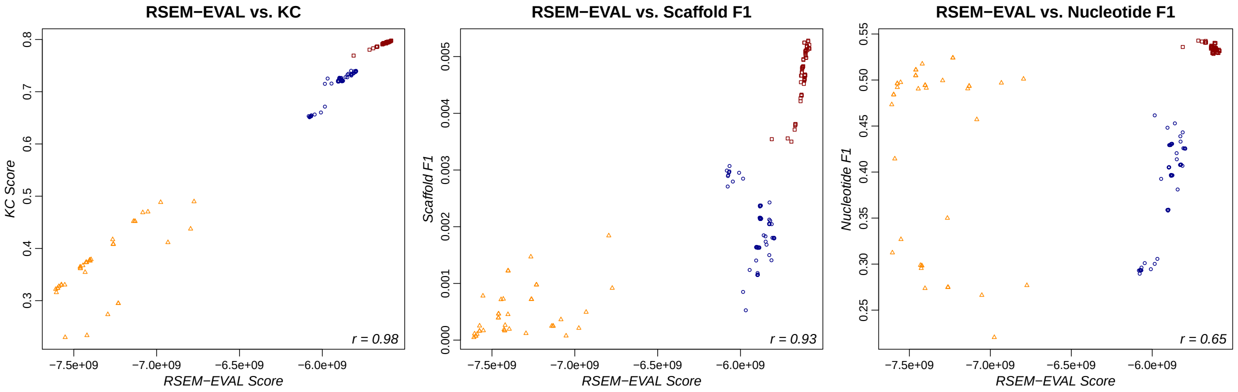

Figure S6: Correlation results of the RSEM-EVAL score for the paired-end strand non-specific data set. The Spearman rank correlation coefficient (bottom-right corner of each plot) was computed for each data set.

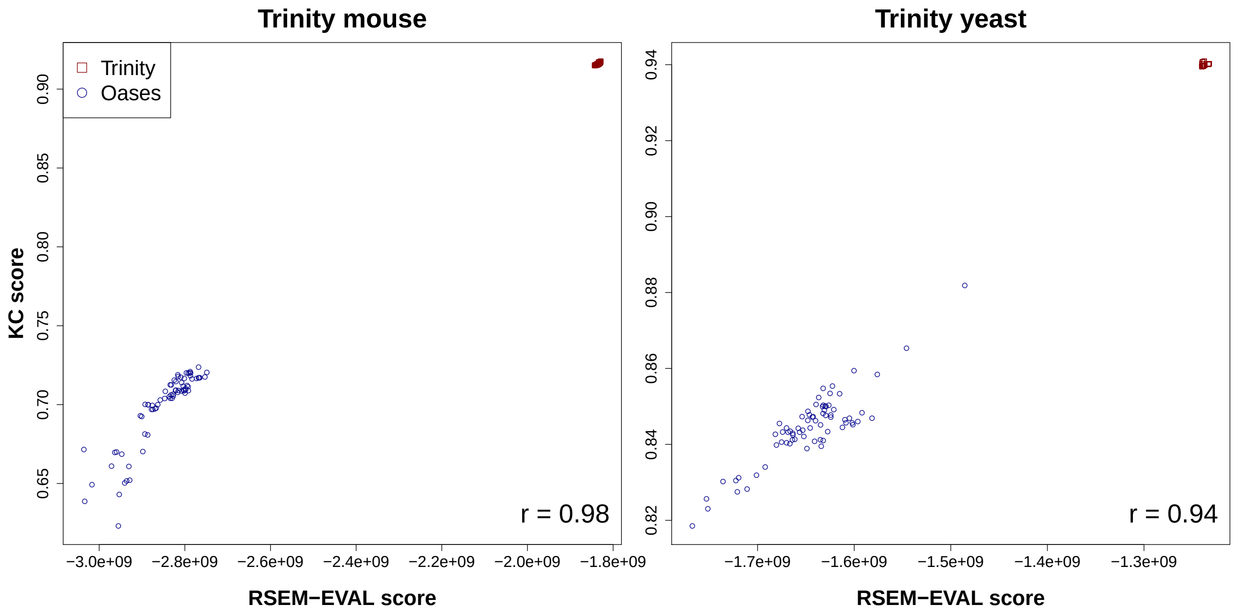

Figure S7: Correlation of the RSEM-EVAL and KC scores on the strand-specific data sets. The Spearman rank correlation coefficient (bottom-right corner of each plot) was computed for each data set.

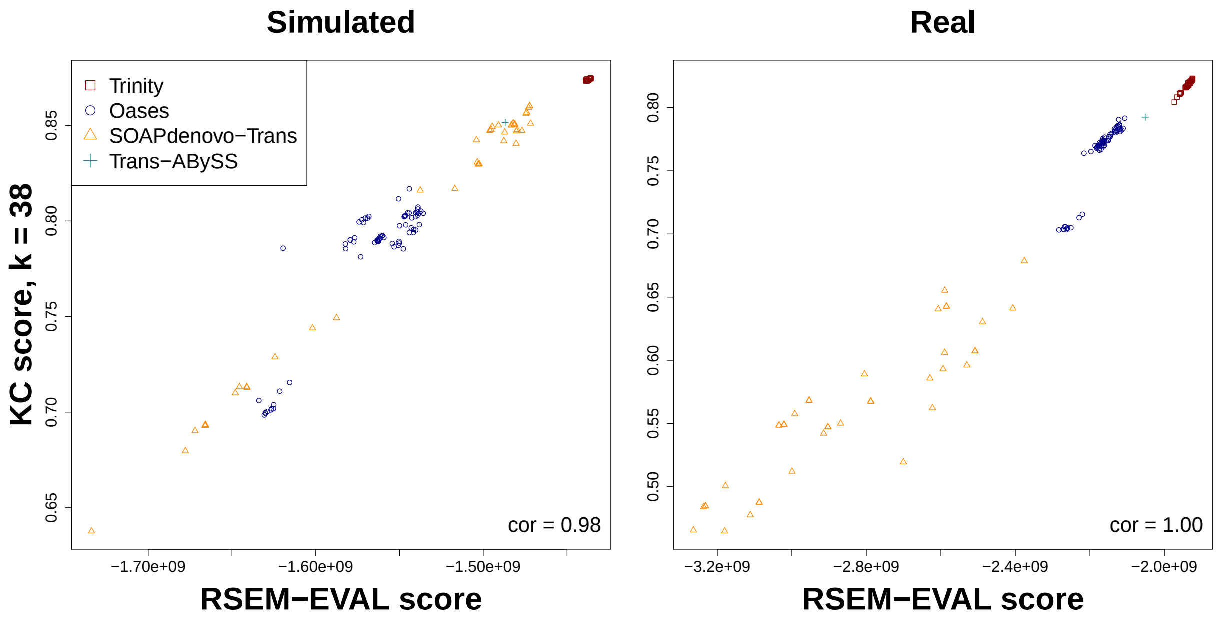

Figure S8: Correlation of the RSEM-EVAL score and KC score with $k = 38$ on the strand non-specific data sets. The Spearman rank correlation coefficient (bottom-right corner of each plot) was computed for each data set.

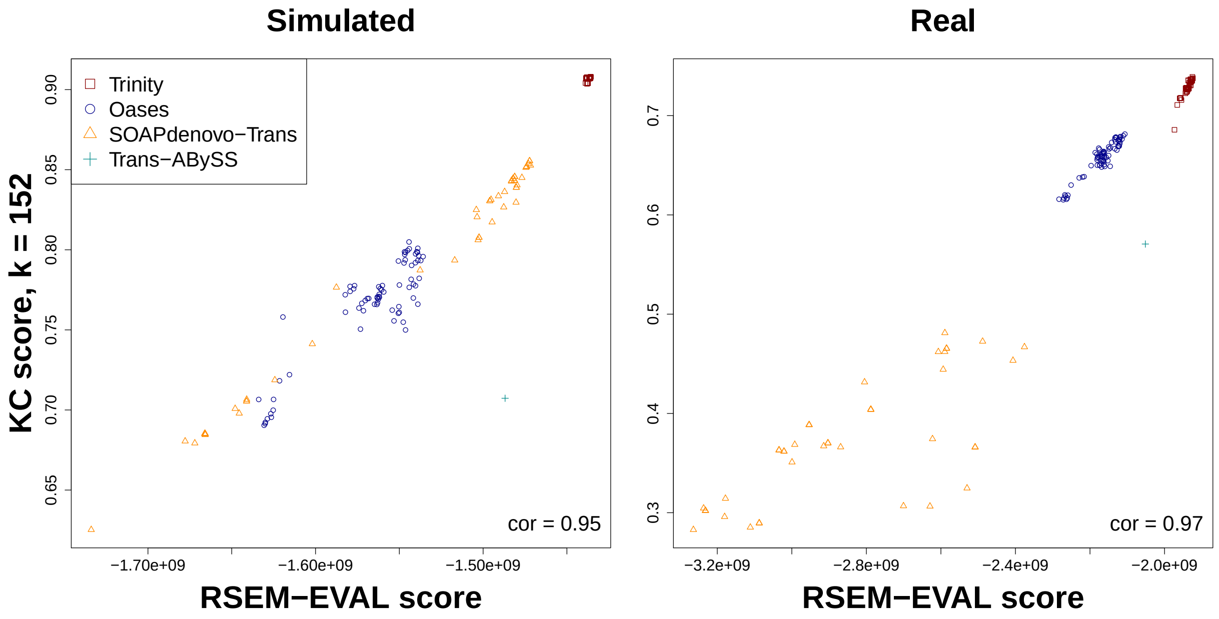

Figure S9: Correlation of the RSEM-EVAL score and KC score with $k = 152$ on the strand non-specific data sets. The Spearman rank correlation coefficient (bottom-right corner of each plot) was computed for each data set.

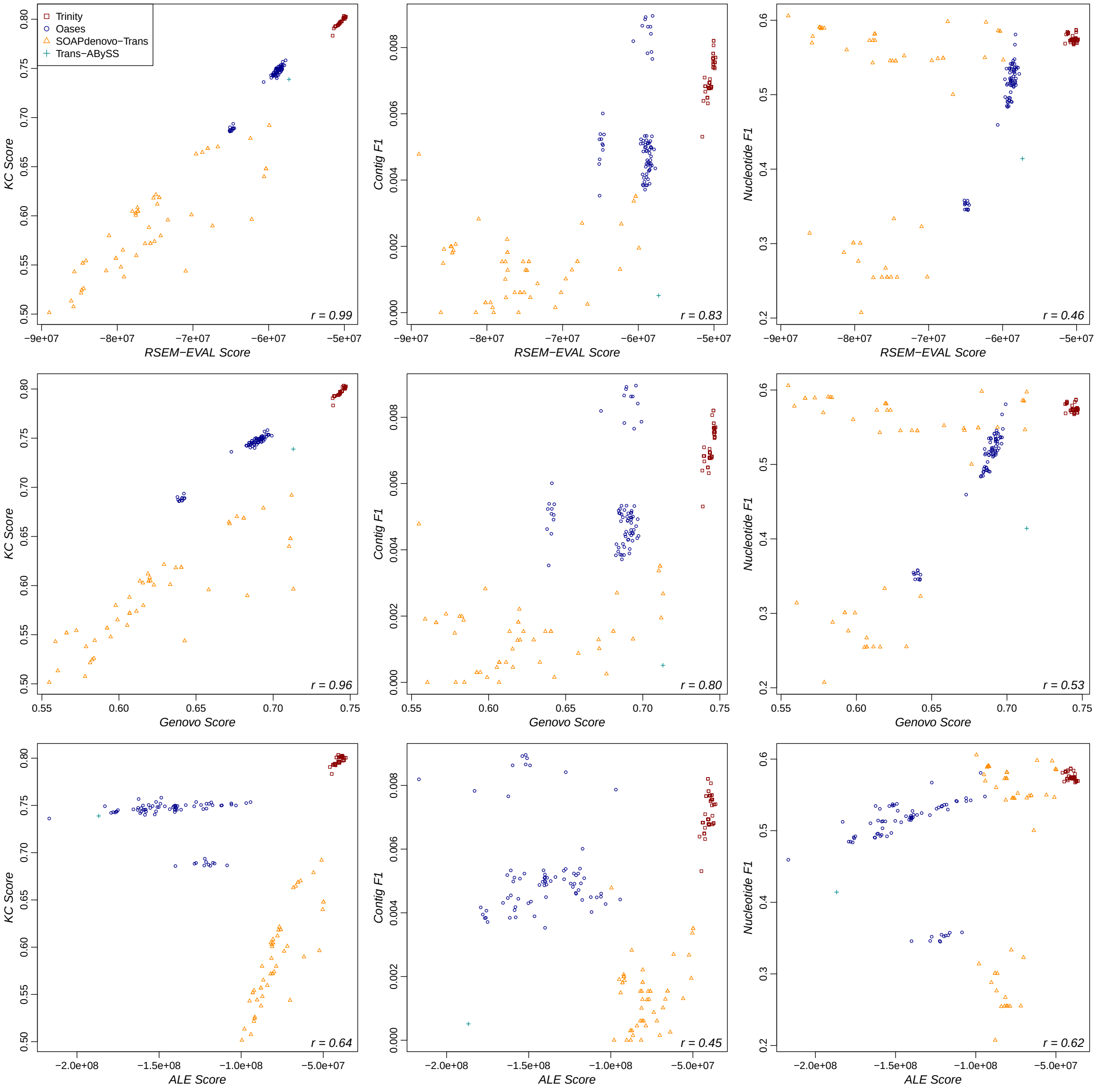

Figure S10: Correlation of the RSEM-EVAL score and alternative model-based measures with the REF-EVAL reference-based measures on the mouse chromosome 1 strand non-specific single-end data set. The Spearman rank correlation coefficient (bottom-right corner of each plot) was computed for each data set.

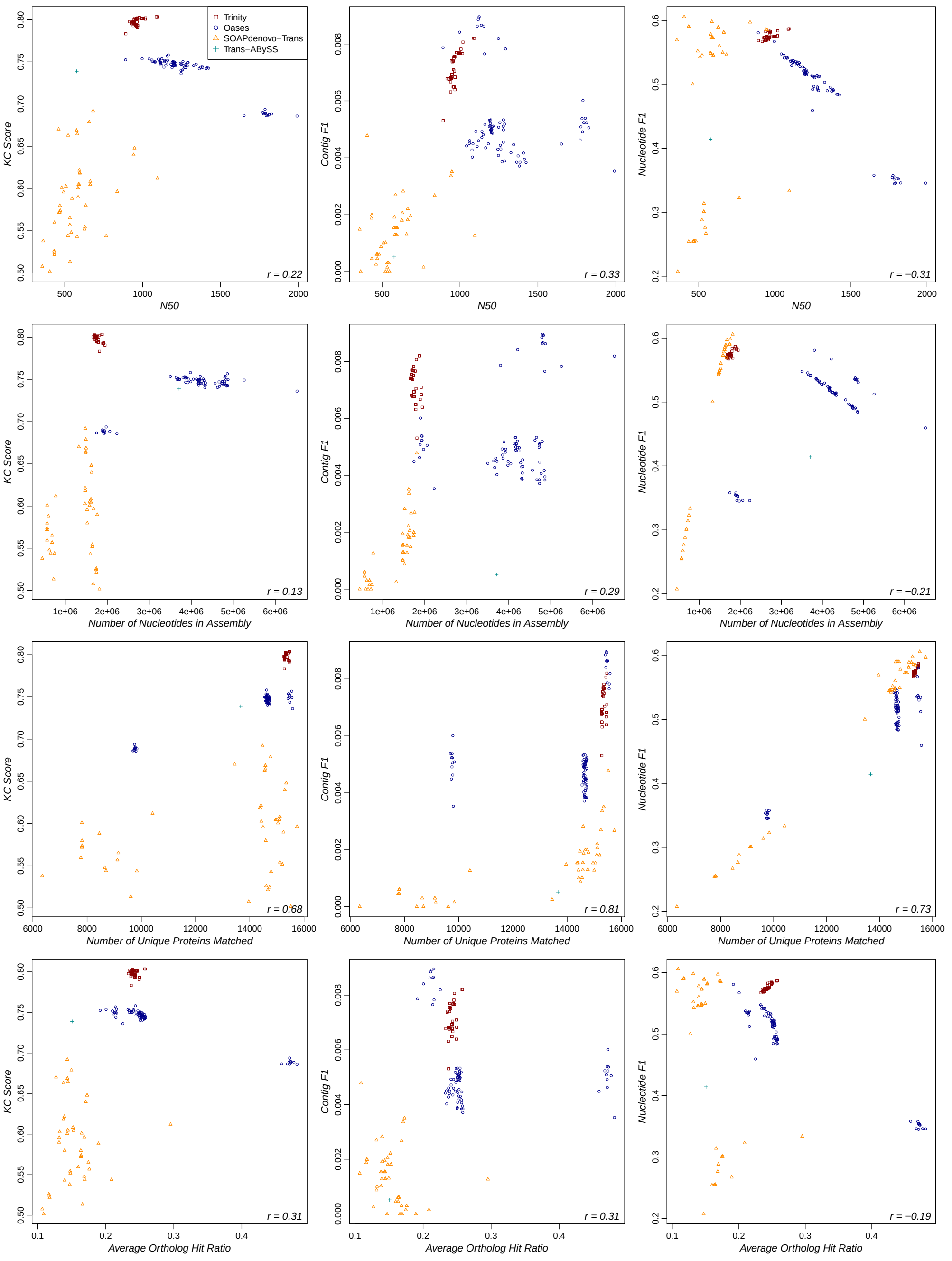

Figure S11: Correlation of the RSEM-EVAL score and alternative reference-free and comparative-reference-based measures with the REF-EVAL reference-based measures on the mouse chromosome 1 strand non-specific single-end data set. The Spearman rank correlation coefficient (bottom-right corner of each plot) was computed for each data set.

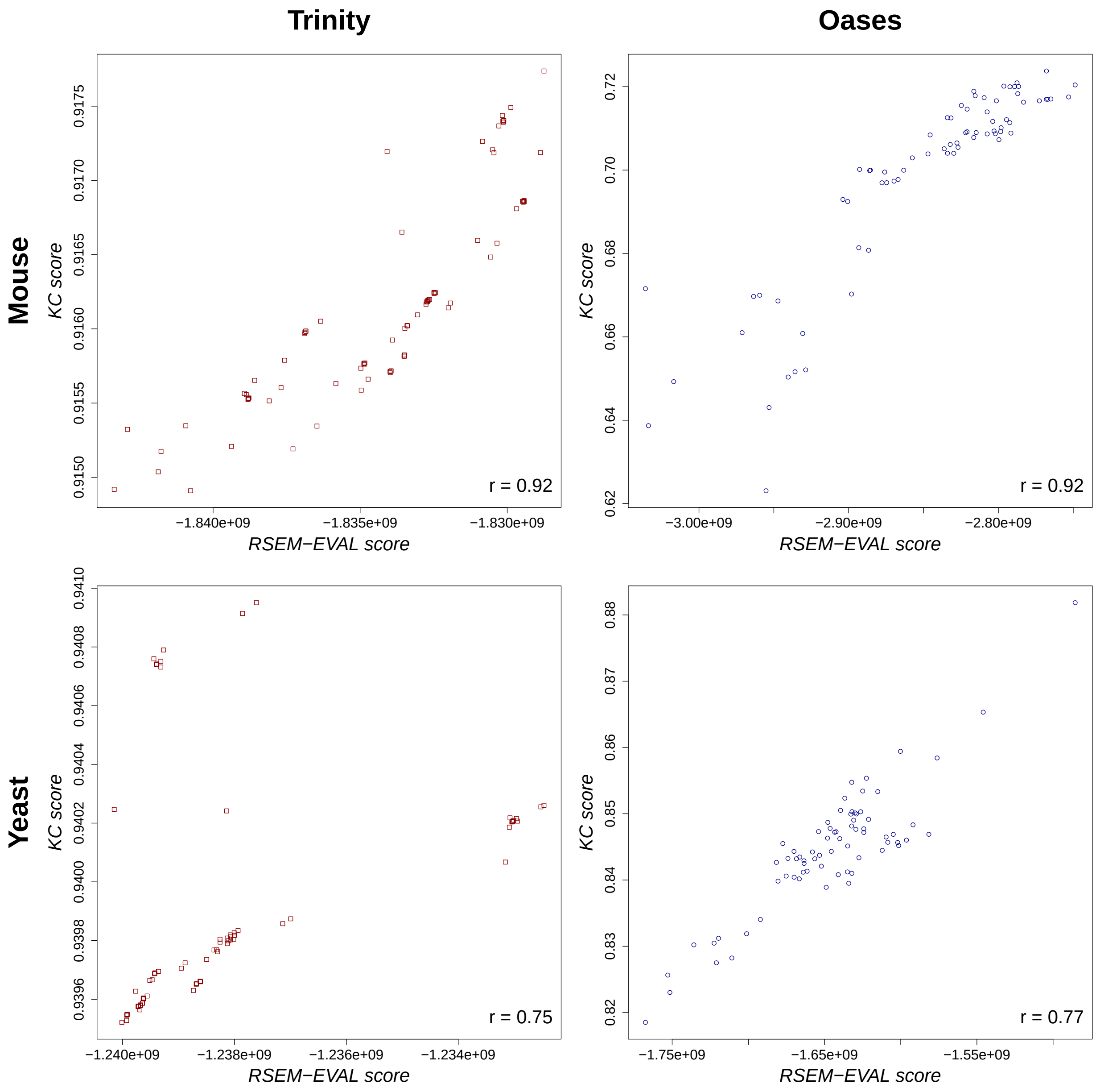

Figure S12: Within-assembler correlation of the RSEM-EVAL and KC scores on the strand-specific data sets. Scatterplots are shown for the real mouse (top row) and real yeast (bottom row) data sets and for the Trinity (left column) and Oases (right column) assemblers. The Spearman rank correlation coefficient (bottom-right corner of each plot) was computed for each combination of data set and assembler.

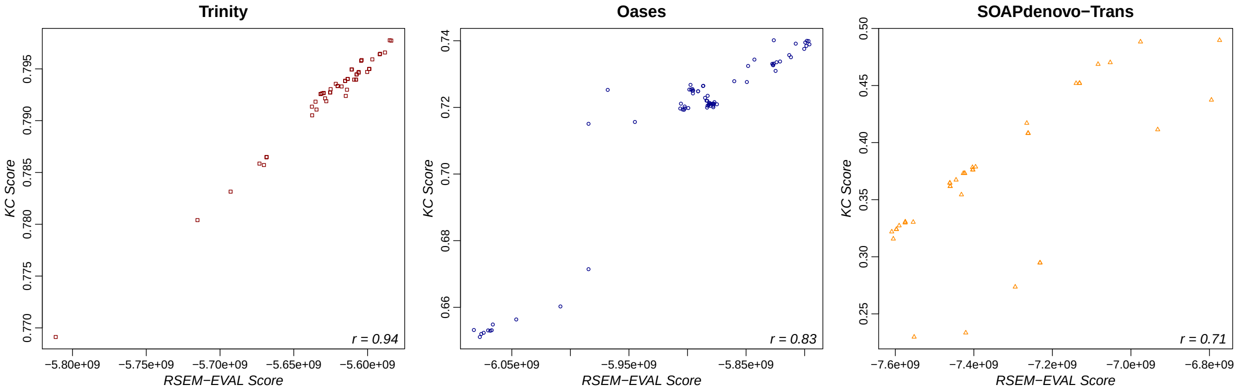

Figure S13: Within assembler correlation results for the RSEM-EVAL score and KC score on the paired-end strand non-specific data set. Scatterplots are shown for the Trinity (left column), Oases (middle column), and SOAPdenovo-Trans (right column) assemblers. The Spearman rank correlation coefficient (bottom-right corner of each plot) was computed for each assembler.

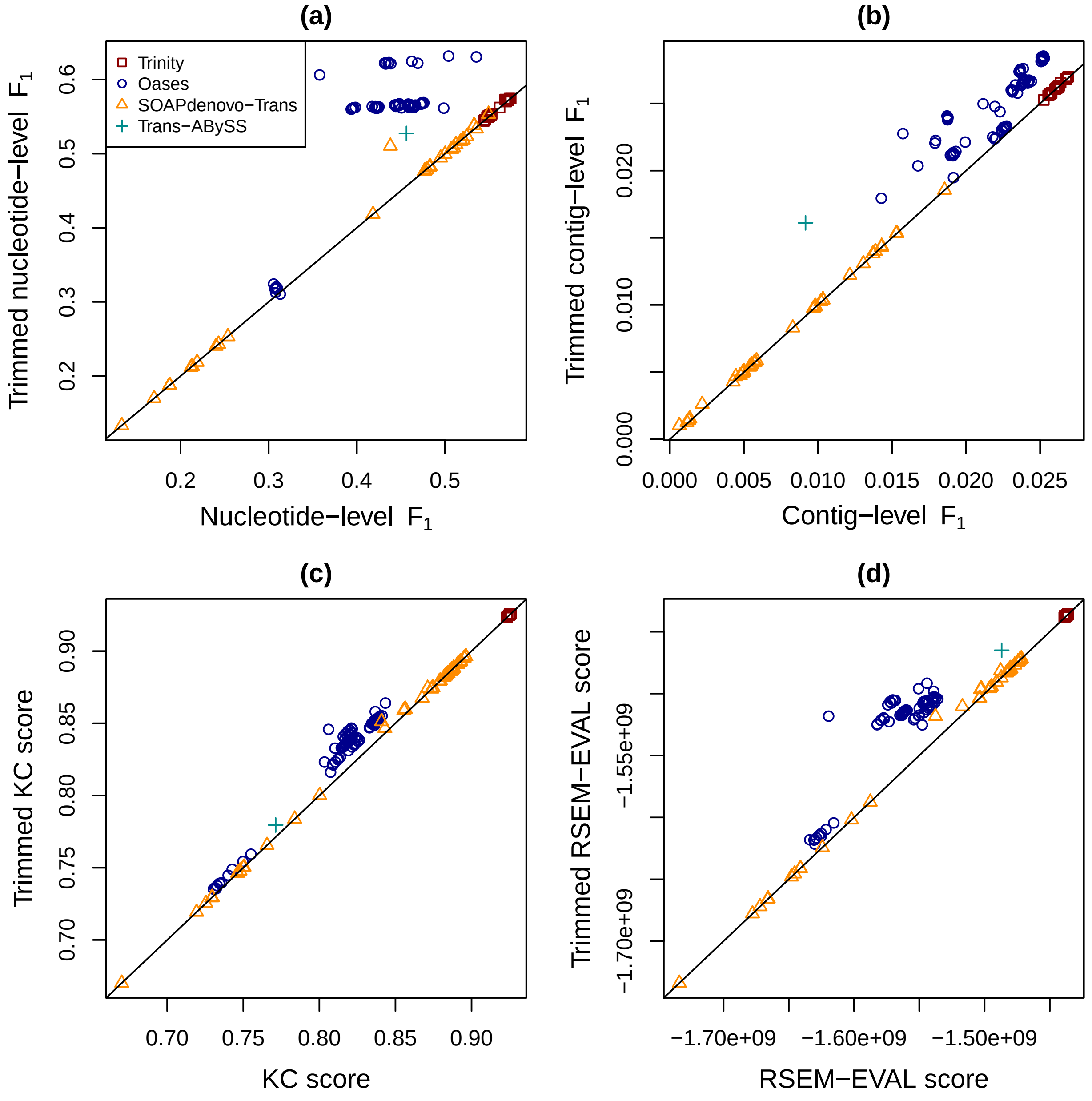

Figure S14: Scatterplots of evaluation measures between the untrimmed assemblies and trimmed assemblies. Measures of the trimmed assemblies were plotted against those for the untrimmed assemblies. The diagonal lines, $y = x$, are shown for easy visualization. The largest improvements were seen for assemblies produced by Oases and Trans-ABySS. For both the nucleotide- and contig-level F_1 scores, the trimmed Oases assemblies were the most accurate of all assemblies (both trimmed and untrimmed).

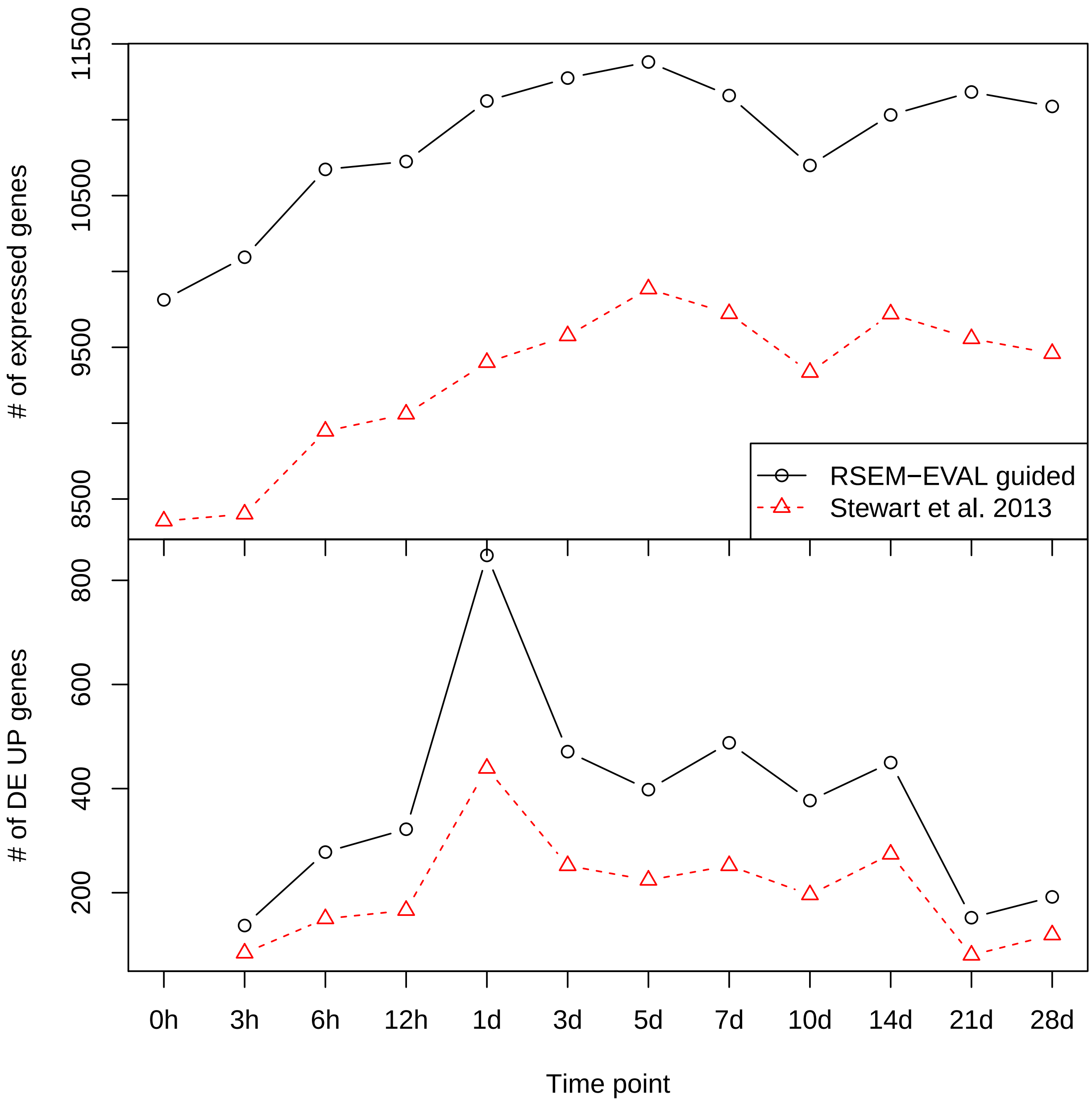

Figure S15: The number of genes expressed (TPM $\geq$ 1) (top) and the number of DE UP genes (bottom) at each time point in the RSEM-EVAL guided (black) and [5] (red) assemblies.
