# Supplementary material for: Evaluation of de novo transcriptome assemblies from RNA-Seq data
Source: Genome Biol. 2014 Dec 21;15(12):553. doi: 10.1186/s13059-014-0553-5 (PMC4298084; doi:10.1186/s13059-014-0553-5)
Supplement: Additional file 3 — Version of the DETONATE source code used for the experiments in this paper. [file 13059_2014_553_MOESM3_ESM.zip › 13059_2014_553_MOESM3_ESM/detonate-1.8.1/html/index.html]

DETONATE: DE novo TranscriptOme rNa-seq Assembly with or without the Truth Evaluation


# DETONATE: DE novo TranscriptOme rNa-seq Assembly with or without the Truth Evaluation

## Overview

DETONATE (DE novo TranscriptOme rNa-seq Assembly with or without the Truth
Evaluation) consists of two component packages, RSEM-EVAL and REF-EVAL. Both
packages are mainly intended to be used to evaluate *de novo*
transcriptome assemblies, although REF-EVAL can be used to compare sets of any
kinds of genomic sequences.

RSEM-EVAL is a reference-free evaluation method based on a novel
probabilistic model that depends only on an assembly and the RNA-Seq reads used
for its construction. Unlike N50, RSEM-EVAL combines multiple factors,
including the compactness of an assembly and the support of the assembly from
the RNA-Seq data, into a single, statistically-principled evaluation score.
This score can be used to select a best assembler, optimize an assembler's
parameters, and guide new assembler design as an objective function. In
addition, for each contig within an assembly, RSEM-EVAL provides a score that
assesses how well that contig is supported by the RNA-Seq data and can be used
to filter unnecessary contigs.

REF-EVAL is a toolkit of reference-based measures, including contig,
nucleotide, and pair precision, recall, and F1 scores, a novel kmer compression
score, and several scores that compare induced kmer distributions between the
assembly and the reference. REF-EVAL also includes a program to estimate the
“true” assembly of a set of RNA-Seq reads, relative to a collection of
full-length reference transcripts. See here, or
ref-eval/README in the distribution, for detailed information.

DETONATE is motivated and described in detail in the following paper:

Bo Li\*, Nathanael Fillmore\*, Yongsheng Bai, Mike Collins, James A. Thompson,
Ron Stewart, Colin N. Dewey. Evaluation of *de novo* transcriptome
assemblies from RNA-Seq data.

\* = equal contributions

## Downloading

The current version of DETONATE (1.8.1) is available here:

- detonate-1.8.1.tar.gz

## Building/installation

To build RSEM-EVAL and REF-EVAL, simply type “make” in the top-level
detonate-1.8.1 directory, after you've upacked the tarball (tar
xvf detonate-1.8.1.tar.gz). If you have trouble with any of this, please
contact us.

## Vignette

A vignette with an extended example of the basic usage of DETONATE is
available here, or in VIGNETTE in the
distribution.

## Usage

For information about using RSEM-EVAL, see here, or rsem-eval/README.md in the distribution.

For information about using REF-EVAL, see here,
or ref-eval/README in the distribution.

For information about using REF-EVAL-ESTIMATE-TRUE-ASSEMBLY, see here, or
ref-eval/README.REF-EVAL-ESTIMATE-TRUE-ASSEMBLY in the distribution.

## Authors

RSEM-EVAL was coded by Bo Li, and REF-EVAL was coded by Nathanael Fillmore.
Bo, Nate, and Colin Dewey jointly developed the RSEM-EVAL and REF-EVAL
methodology, with feedback on the methodology from the other coauthors.

## Mailing list

|  |
| --- |
| **Subscribe to DETONATE Users** |
| Email: |
| Visit this group |

## Development

A repository for the DETONATE source code is available at Github, here.

## Axolotl assembly

The RSEM-EVAL-guided Axolotl assembly described in the DETONATE paper (see
above) is available here.

## Changelog

In DETONATE 1.8.1 (the current version),
released on Oct 2, 2014, the following changes were made:

- Updated the toy example to match that in the paper.

In DETONATE 1.8, released on Sep 26, 2014,
and comprised of RSEM-EVAL 1.8 and REF-EVAL 1.8, the following changes were
made:

- Added support for paired-end data.
- Improved the interface.

In DETONATE 20140123, comprised of
RSEM-EVAL 1.6 and REF-EVAL 20140123, the following changes were made:

- Fixed UI bug in REF-EVAL related to --alignment-policy.
- Incorporated changes from RSEM 1.2.9 into RSEM-EVAL.

DETONATE 20140108, comprised of
RSEM-EVAL 1.5 and REF-EVAL 20140108 was the initial release.
