# Supplementary material for: Evaluation of de novo transcriptome assemblies from RNA-Seq data
Source: Genome Biol. 2014 Dec 21;15(12):553. doi: 10.1186/s13059-014-0553-5 (PMC4298084; doi:10.1186/s13059-014-0553-5)
Supplement: Additional file 3 — Version of the DETONATE source code used for the experiments in this paper. [file 13059_2014_553_MOESM3_ESM.zip › 13059_2014_553_MOESM3_ESM/detonate-1.8.1/html/ref-eval-estimate-true-assembly.html]

REF-EVAL-ESTIMATE-TRUE-ASSEMBLY: A program to estimate the “true”
assembly of a set of reads, relative to a set of reference sequences


# REF-EVAL-ESTIMATE-TRUE-ASSEMBLY: A program to estimate the “true” assembly of a set of reads, relative to a set of reference sequences

## Overview

This program constructs an estimate of the “true” assembly of a set of
reads, relative to a set of reference sequences, based on alignment information
produced by RSEM.

As defined by DETONATE [1], the “true” assembly of a set of reads,
relative to the true transcript sequences that the reads were generated from,
is the set of contiguous subsequences of the transcript sequences that are
covered by reads, with the reads overlapping by at least --min-overlap
bases, when each read is aligned to its true location of origin.

In practice, we do not know the reads' true locations of origin; in fact, we
do not even know (precisely) the true transcript sequences that the reads were
generated from. Instead, we start with a set of reference sequences (e.g., an
Ensembl reference), and we align each read to these reference sequences using
RSEM/Bowtie. The current program chooses a subset of these alignments,
according to a policy specified by --alignment-policy and
--min-alignment-prob, and then builds our best guess as to the
“true” assembly based on these alignments.

[1] Bo Li\*, Nathanael Fillmore\*, Yongsheng Bai, Mike Collins, James A.
Thompson, Ron Stewart, Colin N. Dewey. Evaluation of *de novo*
transcriptome assemblies from RNA-Seq data.

## Example usage

First, use a recent version of RSEM to quantify the expression of the
full-length transcripts relative to the reads. For example:

```
$ rsem-prepare-reference --gtf mm9.ensembl63.filtered.gtf mm9.fa ref
$ rsem-calculate-expression --num-threads 24 reads.fq ref expr
```

Second, use this program to estimate the “true” assembly:

```
$ ./ref-eval-estimate-true-assembly --reference ref --expression expr --assembly cc
```

This will output a file, cc\_0.fa, that contains the “true” assembly.

## General options

-? [ --help ]
:   Display this information.

## Options that specify input and output

--reference arg
:   The prefix of the reference built by
    rsem-prepare-reference. Required.

--expression arg
:   The prefix of the expression built by
    rsem-calculate-expression. Required.

--paired-end
:   If you have paired-end data, and you want to estimate the “true”
    scaffolded assembly, then include the --paired-end flag. In this case,
    rsem-calculate-expression needs to have been run with the --paired-end
    flag. (However, even if rsem-calculate-expression was run with the
    --paired-end flag, you can omit it here in order to generate an
    unscaffolded assembly. In this case, each mate will be treated as an
    independent read.)

--assembly arg
:   A prefix to write the “true” assembly or sequence of assemblies
    to. The suffix \_*x*.fa will be appended to this prefix,
    where *x* is the minimum overlap size. Required.

## Options that change the output

--min-overlap arg
:   Either:

    - An integer that specifies how much overlap between two reads is
      required to merge two reads. For example, if
      --min-overlap=3, then only reads whose chosen alignments
      overlap by at least 3 bases will be joined into contigs. If
      --min-overlap=0, then only reads whose chosen alignments
      are contiguous (or overlap by a positive amount) will be joined
      into contigs.

    Or:

    - A pair of integers, separated by commas, specifying a range of
      overlap sizes, as described above. For example, if
      --min-overlap=2,4 is given, then three assemblies will be
      produced, corresponding to --min-overlap=2,
      --min-overlap=3, and --min-overlap=4 You might
      use this option to compute ideal assemblies at all overlap sizes,
      e.g., --min-overlap=0,76 for 76-length reads.

    Default: 0.

--min-alignment-prob arg
:   A number between 0 and 1 (inclusive). Any alignment (of a read to a
    reference transcript) with posterior probability, as calculated by
    RSEM, strictly less than this value will be discarded. Noise reads,
    with posterior probability exactly 0, are always discarded. Default:
    0.

--alignment-policy arg
:   The policy used to choose which alignment(s) of each read to use in
    constructing the “true” assembly. Options:

    - sample:
      For each read, sample a single alignment (to some reference
      transcript) according to the posterior probability that the
      read follows each alignment, as calculated by RSEM.
    - best:
      For each read, choose the alignment that maximizes the
      posterior probability mentioned above. Ties are broken
      arbitrarily but deterministically (the first alignment in the
      BAM file is used).
    - all:
      For each read, use all its alignments. Some reads might end
      up with more than one alignment. In that case, contigs will
      be made assuming that the read aligns to each place. (In
      other words, the read is effectively duplicated, with one
      copy per alignment.)

    This policy is applied after the thresholding implied by
    --min-alignment-prob. For example, if
    “--min-alignment-prob=0.10 --alignment-policy=sample” is
    given, then (first) all alignments with posterior probability less than
    0.10 will be discarded, and (second), for each read, an alignment will
    be sampled from among the remaining alignments, with the posterior
    distribution renormalized as appropriate. As another example, if
    “--min-alignment-prob=0.90 --alignment-policy=all” is given,
    then all alignments with posterior probability at least 0.90 will be
    used.

    Default: sample.
