# Supplementary material for: Evaluation of de novo transcriptome assemblies from RNA-Seq data
Source: Genome Biol. 2014 Dec 21;15(12):553. doi: 10.1186/s13059-014-0553-5 (PMC4298084; doi:10.1186/s13059-014-0553-5)
Supplement: Additional file 3 — Version of the DETONATE source code used for the experiments in this paper. [file 13059_2014_553_MOESM3_ESM.zip › 13059_2014_553_MOESM3_ESM/detonate-1.8.1/html/rsem-eval.html]

RSEM-EVAL: A novel reference-free transcriptome assembly evaluation measure


# RSEM-EVAL: A novel reference-free transcriptome assembly evaluation measure

## Introduction

RSEM-EVAL is built off of RSEM. It is a reference-free de novo transcriptome assembly evaluator. This document will only cover the RSEM-EVAL only features. For the shared feature with RSEM, please refer to 'README\_RSEM.md'.

## Compilation & Installation

To compile RSEM-EVAL, simply run

```
make
```

To install, simply put the rsem directory in your environment's PATH
variable.

### Prerequisites

C++, Perl and R are required to be installed.

To take advantage of RSEM-EVAL's built-in support for the Bowtie alignment
program, you must have Bowtie installed.

## Usage

### I. Build an assembly from the RNA-Seq data using an assembler

Please note that the RNA-Seq data set used to build the assembly
should be exactly the same as the RNA-Seq data set for evaluating this
assembly. RSEM-EVAL supports both single-end and paired-end reads.

### II. Estimate Transcript Length Distribution Parameters

RSEM-EVAL provides a script
'rsem-eval-estimate-transcript-length-distribution' to estimate
transcript length distribution from a set of transcript
sequences. Transcripts can be from a closely related species to the
orgaism whose transcriptome is sequenced. Its usage is:

```
rsem-eval-estimate-transcript-length-distribution input.fasta parameter_file
```

\_\_input.fasta\_\_ is a multi-FASTA file contains all transcript sequences used to learn the true transcript length distribution's parameters.   
\_\_parameter\_file\_\_ records the learned parameters--the estimated mean and standard deviation (separated by a tab character).

We have some learned paramter files stored at folder
'true\_transcript\_length\_distribution'. Please refer to
'true\_length\_dist\_mean\_sd.txt' in the folder for more details.

### III. Calculate the RSEM-EVAL score

To calculate the RSEM-EVAL score, you should use 'rsem-eval-calculate-score'. Run

```
rsem-eval-calculate-score --help
```

to get usage information.

### IV. Outputs related to the evaluation score

RSEM-EVAL produces the following three score related files:
'sample\_name.score', 'sample\_name.isoforms.results' and
'sample\_name.genes.results'.

'sample\_name.score' stores the evaluation score for the evaluated
assembly. It contains 14 lines and each line contains a name and a
value separated by a tab.

The first 6 lines provide: 'Score', the
RSEM-EVAL score; 'BIC\_penalty', the BIC penalty term;
'Prior\_score\_on\_contig\_lengths', the log score of priors of contig
lengths; 'Prior\_score\_on\_contig\_sequences', the log score of priors of
contig sequence bases; 'Data\_likelihood\_in\_log\_space\_without\_correction', the RSEM log data
likelihood calculated with contig-level read generating probabilities
mentioned in the supplement text of our DETONATE manuscript;
'Correction\_term', the correction term. Score = BIC\_penalty +
Prior\_score\_on\_contig\_lengths + Prior\_score\_on\_contig\_sequences +
Data\_likelihood\_in\_log\_space\_without\_correction - Correction\_term.

The next 8 lines provide statistics that may help users to understand
the RSEM-EVAL score better. They are: 'Number\_of\_contigs', the number
of contigs contained in the assembly; 'Expected\_number\_of\_ali
gned\_reads\_given\_the\_data', the expected number of reads assigned to
each contig estimated using the contig-level read generating
probabilities mentioned in the supplement text of our DETONATE
manuscri pt;
'Number\_of\_contigs\_smaller\_than\_expected\_read/fragment\_length', the
number of contigs whose length is smaller than the expected
read/fragment length; 'Number\_of\_contigs\_with\_no\_read\_aligned\_to', the
number of contigs whose expected number of aligned reads is smaller
than 0.005; 'Maximum\_data\_likelihood\_in\_log\_space', the maximum data
likelihood in log space calculated from RSEM by treating the assembly
as "true" transcripts; 'Number\_of\_alignable\_reads', the number of
reads that have at least one alignment found by the aligner (Because
'rsem-calculate-expression' tries to use a very loose criteria to find
alignments, reads with only low quality alignments may also be counted
as alignable reads here); 'Number\_of\_alignments\_in\_total', the number
of total alignments found by the aligner;
'Transcript\_length\_distribution\_related\_factors', the term related to
transcript length distribution in 'Prior\_score\_on\_contig\_lengths' (it
is the summation of log c\_{\lambda}(\ell) terms mentioned in
'Calculation of the contig length distribution' subsection of the
supplementary text of our DETONATE manuscript.

'sample\_name.score.isoforms.results' and
'sample\_name.score.genes.results' output "corrected" expression levels
based on contig-level read generating probabilities mentioned in the
supplement of the DETONATE manuscript. Unlike
'sample\_name.isoforms.results' and 'sample\_name.genes.results', which
are calculated by treating the contigs as true transcripts,
calculating 'sample\_name.score.isoforms.results' and
'sample\_name.score.genes.results' involves first estimating expected
read coverage for each contig and then convert the expected read
coverage into contig-level read generating probabilities. This
procedure is aware of that provided sequences are contigs and gives
better expression estimates for very short contigs. In addtion, the
'TPM' field is changed to 'CPM' field, which stands for contig per
million.

For 'sample\_name.score.isoforms.results', one additional
column is added. The additional column is named as
'contig\_impact\_score' and gives the contig impact score for each
contig as described in the DETONATE manuscript.

## Example

We have a toy example in the 'examples' folder of the detonate distribution. A
single true transcript is stored at file 'toy\_ref.fa'. The single-end, 76bp
reads generated from this transcript are stored in file 'toy\_SE.fq'. In
addition, we have three different assemblies based on the data:
'toy\_assembly\_1.fa', 'toy\_assembly\_2.fa' and 'toy\_assembly\_3.fa'. We also know
the true transcript is from mouse and thus use 'mouse.txt' under
'true\_transcript\_length\_distribution' as our transcript length parameter file.

We run (assuming that we are in the rsem-eval directory)

```
./rsem-eval-calculate-score -p 8 \
              --transcript-length-parameters true_transcript_length_distribution/mouse.txt \
              ../examples/toy_SE.fq \
              ../examples/toy_assembly_1.fa \
              toy_assembly_1
              76
```

to obtain the RSEM-EVAL score.

The RSEM-EVAL score can be found in 'toy\_assembly\_1.score'. The
contig impact scores can be found in
'toy\_assembly\_1.score.isoforms.results'.

## Authors

RSEM-EVAL is developed by Bo Li, with substaintial technical input from Colin Dewey and Nate Fillmore.

## Acknowledgements

Please refer to the acknowledgements section in 'README\_RSEM.md'.

## License

RSEM-EVAL is licensed under the GNU General Public License
v3.
