# Supplementary material for: Evaluation of de novo transcriptome assemblies from RNA-Seq data
Source: Genome Biol. 2014 Dec 21;15(12):553. doi: 10.1186/s13059-014-0553-5 (PMC4298084; doi:10.1186/s13059-014-0553-5)
Supplement: Additional file 3 — Version of the DETONATE source code used for the experiments in this paper. [file 13059_2014_553_MOESM3_ESM.zip › 13059_2014_553_MOESM3_ESM/detonate-1.8.1/html/vignette.html]

Vignette for DETONATE


# Vignette for DETONATE

This document provides a brief tutorial on how to run DETONATE.
We focus on the basic commands, inputs, and outputs involved.
For information on the definitions, motivations, and validation of DETONATE's methods, see our paper [1] and the rest of the DETONATE website.

[1] Bo Li\*, Nathanael Fillmore\*, Yongsheng Bai, Mike Collins, James A. Thompson,
Ron Stewart, Colin N. Dewey. Evaluation of *de novo* transcriptome
assemblies from RNA-Seq data.

\* = equal contributions

## Step 1: Acquire RNA-Seq data and build de novo assemblies

Before we can evaluate assemblies, we need to acquire RNA-Seq data and construct the assemblies, using our favorite transcriptome assembly software (Trinity, Oases, SOAPdenovo-Trans, Trans-ABySS, etc.).
For the purposes of this vignette, we will use a tiny example dataset, available in examples/toy\_SE.fq, and three assemblies of this data, examples/toy\_assembly\_1.fa, examples/toy\_assembly\_2.fa, and examples/toy\_assembly\_3.fa.

(All paths in this vignette are relative to the root directory of the DETONATE distribution.)

## Step 2: Build the DETONATE software

Another preliminary step is to build the DETONATE software.
To do so, simply run make in the root directory of the DETONATE distribution.
This will build both RSEM-EVAL and REF-EVAL, plus several dependencies.

## Step 3: Run RSEM-EVAL on each assembly

Now we are ready to run either RSEM-EVAL or REF-EVAL to evaluate our assemblies.
Let's start with RSEM-EVAL.
We run RSEM-EVAL on the three assemblies of our reads as follows.

```
$ ./rsem-eval/rsem-eval-calculate-score examples/toy_SE.fq examples/toy_assembly_1.fa examples/rsem_eval_1 76 --transcript-length-parameters rsem-eval/true_transcript_length_distribution/mouse.txt -p 16
$ ./rsem-eval/rsem-eval-calculate-score examples/toy_SE.fq examples/toy_assembly_2.fa examples/rsem_eval_2 76 --transcript-length-parameters rsem-eval/true_transcript_length_distribution/mouse.txt -p 16
$ ./rsem-eval/rsem-eval-calculate-score examples/toy_SE.fq examples/toy_assembly_3.fa examples/rsem_eval_3 76 --transcript-length-parameters rsem-eval/true_transcript_length_distribution/mouse.txt -p 16
```

Above, the first argument to RSEM-EVAL specifies the reads, the second argument specifies the assembly, and the third argument specifies the prefix of RSEM-EVAL's output.

The fourth argument, 76, is the read length in our data.
If the reads were of varying lengths, the fourth argument would be the average read length.
For paired-end data, the fourth argument would be the (average) fragment length.

The --transcript-length-parameters option instructs RSEM-EVAL to parameterize its prior distribution using the mean and standard deviation of the transcript lengths in the Ensembl mouse annotation.
These parameters can also be estimated from a species more closely related to the one you are interested in, using ./rsem-eval/rsem-eval-estimate-transcript-length-distribution.
If --transcript-length-parameters is not provided, default transcript-length parameters, estimated from the human Ensembl annotation, will be used.

The -p option tells RSEM-EVAL how many threads to use, 16 in this example.

After running RSEM-EVAL as above, the RSEM-EVAL scores for our assemblies will be in the files examples/rsem\_eval\_1.score, examples/rsem\_eval\_2.score, and examples/rsem\_eval\_3.score.
Let's look at one of these score files:

```
$ cat examples/rsem_eval_1.score
Score   -87426.14
BIC_penalty     -8.25
Prior_score_on_contig_lengths   -7.91
Prior_score_on_contig_sequences -867.82
Data_likelihood_in_log_space_without_correction -86542.17
Correction_term -0.00
Number_of_contigs       1
Expected_number_of_aligned_reads_given_the_data 3812.00
Number_of_contigs_smaller_than_expected_read/fragment_length    0
Number_of_contigs_with_no_read_aligned_to       0
Maximum_data_likelihood_in_log_space    -86541.97
Number_of_alignable_reads       3812
Number_of_alignments_in_total   3812
Transcript_length_distribution_related_factors  -7.91
```

The first line contains the RSEM-EVAL score.
The remaining lines break down this score and provide other related information.

Now let's compare the three assemblies' RSEM-EVAL scores:

```
$ cat examples/rsem_eval_1.score | awk '$1 == "Score"'
Score   -87426.14
$ cat examples/rsem_eval_2.score | awk '$1 == "Score"'
Score   -201465.39
$ cat examples/rsem_eval_3.score | awk '$1 == "Score"'
Score   -254935.35
```

The first assembly has a substantially better RSEM-EVAL score than the other two assemblies, and the second assembly has a slightly better RSEM-EVAL score than the third.

## Step 4: Estimate the “true” assembly.

We now proceed to a reference-based comparison of the assemblies.
A reference for our toy example is available in examples/toy\_ref.fa.
It contains a single transcript.

We will compare our assemblies to an estimated “true” assembly.
To do so, we first need to construct this estimate, and this can be done using ref-eval/ref-eval-estimate-true-assembly.

A preliminary step, though, is to run RSEM relative to the set of full-length reference transcripts.
(The point here is not to compute the expression levels of each transcript, which is RSEM's primary purpose, but rather to compute the posterior probability of each read's alignment to the reference.)
To do so, download RSEM from its website, unpack it, and build it by typing make in its root directory.
Let's say that you have installed RSEM in the directory /path/to/rsem.
(This example uses version 1.2.17.)
Then we run RSEM as follows:

```
$ /path/to/rsem/rsem-prepare-reference --bowtie examples/toy_ref.fa examples/toy_rsem_ref
$ /path/to/rsem/rsem-calculate-expression -p 12 examples/toy_SE.fq examples/toy_rsem_ref examples/toy_rsem_expr
```

Now we are ready to estimate the “true” assembly:

```
$ ./ref-eval/ref-eval-estimate-true-assembly --reference examples/toy_rsem_ref --expression examples/toy_rsem_expr --assembly examples/ta --alignment-policy best
```

The first two options (--reference and --expression) tell REF-EVAL where to find the alignment information output by RSEM.

The third option (--assembly) tells REF-EVAL to output the estimated “true” assembly in a file with prefix examples/ta.
Specifially, the estimated “true” assembly will be output to examples/ta\_0.fa.

The fourth option (--alignment-policy best) tells REF-EVAL to use the highest-probability alignment of each read in constructing the estimated “true” assembly.
In the paper, we used --alignment-policy sample, but we use best here so that our results are deterministic.

The estimated “true” assembly contains one contig in this case; is a bit shorter than the full-length transcript.

## Step 5: Compute the kmer-compression score for each assembly.

Next, we will compare each assembly to the estimated “true” assembly using the kmer-compression (KC) score.

To do so, a preliminary step is to run RSEM again.
This time, we will run RSEM to estimate the expression levels of each sequence in the estimated “true” assembly, as follows:

```
$ /path/to/rsem/rsem-prepare-reference --bowtie examples/ta_0.fa examples/ta_0_ref
$ /path/to/rsem/rsem-calculate-expression -p 12 examples/toy_SE.fq examples/ta_0_ref examples/ta_0_expr
```

We now compute the KC score of each assembly as follows:

```
$ ./ref-eval/ref-eval --scores kc --A-seqs examples/toy_assembly_1.fa --B-seqs examples/ta_0.fa --B-expr examples/ta_0_expr.isoforms.results --kmerlen 76 --readlen 76 --num-reads 46988 | tee examples/kc_1.txt
$ ./ref-eval/ref-eval --scores kc --A-seqs examples/toy_assembly_2.fa --B-seqs examples/ta_0.fa --B-expr examples/ta_0_expr.isoforms.results --kmerlen 76 --readlen 76 --num-reads 46988 | tee examples/kc_2.txt
$ ./ref-eval/ref-eval --scores kc --A-seqs examples/toy_assembly_3.fa --B-seqs examples/ta_0.fa --B-expr examples/ta_0_expr.isoforms.results --kmerlen 76 --readlen 76 --num-reads 46988 | tee examples/kc_3.txt
```

The above commands instruct REF-EVAL to compute the KC score (--scores kc) of the assembly (e.g., --A-seqs examples/toy\_assembly\_1.fa) versus the estimated “true” assembly (--B-seqs examples/ta\_0.fa).
The expression profile of the estimated “true” assembly is given by --B-expr examples/ta\_0\_expr.isoforms.results.
We also provide REF-EVAL with the kmer length (--kmerlen 76) to use in computing the KC; this will typically be the read length or average read length.
Finally, we provide REF-EVAL with the number of reads (--num-reads 46988) and the read length (--readlen 76); here, what is important is that the number of reads times the read length equals the total number of nucleotides in the read set.

Each of these the output files, e.g., examples/kc\_1.txt, contains the KC score and its two constitutive terms:

```
$ cat examples/kc_1.txt 
weighted_kmer_recall    0.862069
inverse_compression_rate        0.000175297
kmer_compression_score  0.861894
```

Now let's compare the three assemblies' KC scores:

```
$ cat examples/kc_1.txt | awk '$1 == "kmer_compression_score"'
kmer_compression_score  0.861894
$ cat examples/kc_2.txt | awk '$1 == "kmer_compression_score"'
kmer_compression_score  0.520331
$ cat examples/kc_3.txt | awk '$1 == "kmer_compression_score"'
kmer_compression_score  0.509861
```

Like for the RSEM-EVAL score, the first assembly has a substantially better KC score than the other two assemblies, and the second assembly has a slightly better KC score than the third.

## Step 6: Compute the alignment-based scores for each assembly.

Finally, we compute the contig and nucleotide F1 scores.

To do so, we need to align each assembly to the estimated "true" assembly, and vice versa, using Blat.
Download Blat from its website (here, we use version 3.4), unpack it, and build it.
Let's say that you have installed Blat at /path/to/blat.
Then we run Blat as follows:

```
$ /path/to/blat -minIdentity=80 examples/ta_0.fa examples/toy_assembly_1.fa examples/toy_assembly_1_to_ta_0.psl
$ /path/to/blat -minIdentity=80 examples/ta_0.fa examples/toy_assembly_2.fa examples/toy_assembly_2_to_ta_0.psl
$ /path/to/blat -minIdentity=80 examples/ta_0.fa examples/toy_assembly_3.fa examples/toy_assembly_3_to_ta_0.psl
$ /path/to/blat -minIdentity=80 examples/toy_assembly_1.fa examples/ta_0.fa examples/ta_0_to_toy_assembly_1.psl
$ /path/to/blat -minIdentity=80 examples/toy_assembly_2.fa examples/ta_0.fa examples/ta_0_to_toy_assembly_2.psl
$ /path/to/blat -minIdentity=80 examples/toy_assembly_3.fa examples/ta_0.fa examples/ta_0_to_toy_assembly_3.psl
```

We can now compute the contig and nucleotide scores as follows:

```
$ ./ref-eval/ref-eval --scores contig,nucl --weighted no --A-seqs examples/toy_assembly_1.fa --B-seqs examples/ta_0.fa --A-to-B examples/toy_assembly_1_to_ta_0.psl --B-to-A examples/ta_0_to_toy_assembly_1.psl --min-frac-identity 0.90 | tee examples/contig_nucl_1.txt
$ ./ref-eval/ref-eval --scores contig,nucl --weighted no --A-seqs examples/toy_assembly_2.fa --B-seqs examples/ta_0.fa --A-to-B examples/toy_assembly_2_to_ta_0.psl --B-to-A examples/ta_0_to_toy_assembly_2.psl --min-frac-identity 0.90 | tee examples/contig_nucl_2.txt
$ ./ref-eval/ref-eval --scores contig,nucl --weighted no --A-seqs examples/toy_assembly_3.fa --B-seqs examples/ta_0.fa --A-to-B examples/toy_assembly_3_to_ta_0.psl --B-to-A examples/ta_0_to_toy_assembly_3.psl --min-frac-identity 0.90 | tee examples/contig_nucl_3.txt
```

Each of these the output files, e.g., examples/contig\_nucl\_1.txt, contains the (unweighted) contig and nucleotide precision, recall, and F1 scores:

```
$ cat examples/contig_nucl_1.txt
unweighted_nucl_precision       0.998403
unweighted_nucl_recall  0.998403
unweighted_nucl_F1      0.998403
unweighted_contig_recall        1
unweighted_contig_precision     1
unweighted_contig_F1    1
```

Now let's compare the three assemblies' contig F1 scores:

```
$ cat examples/contig_nucl_1.txt | awk '$1 == "unweighted_contig_F1"'
unweighted_contig_F1    1
$ cat examples/contig_nucl_2.txt | awk '$1 == "unweighted_contig_F1"'
unweighted_contig_F1    0
$ cat examples/contig_nucl_3.txt | awk '$1 == "unweighted_contig_F1"'
unweighted_contig_F1    0
```

The first assembly recovered the single contig in the estimated “true” assembly, but the other two assemblies did not recover it, at least not to > 90 percent identity. This fact is also obvious from looking at the assemblies.

Now let's compare the three assemblies' nucleotide F1 scores:

```
$ cat examples/contig_nucl_1.txt | awk '$1 == "unweighted_nucl_F1"'
unweighted_nucl_F1      0.998403
$ cat examples/contig_nucl_2.txt | awk '$1 == "unweighted_nucl_F1"'
unweighted_nucl_F1      0.419405
$ cat examples/contig_nucl_3.txt | awk '$1 == "unweighted_nucl_F1"'
unweighted_nucl_F1      0.815516
```
