# Supplementary material for: Evaluation of de novo transcriptome assemblies from RNA-Seq data
Source: Genome Biol. 2014 Dec 21;15(12):553. doi: 10.1186/s13059-014-0553-5 (PMC4298084; doi:10.1186/s13059-014-0553-5)
Supplement: Additional file 3 — Version of the DETONATE source code used for the experiments in this paper. [file 13059_2014_553_MOESM3_ESM.zip › 13059_2014_553_MOESM3_ESM/detonate-1.8.1/ref-eval/boost/tools/build/v2/doc/development_plan.html]

Boost Build System V2

 

# Boost Build Development Plan

- **Milestone 2** (October 25, 2002)

  Boost buildable with gcc and also usable as part of another
  project.
- **Milestone 3** (May 21, 2003)

  Two gcc versions and two other compilers are supported. Some other
  tools (e.g. lex and BoostBook) are implemented.
- **Milestone 4** (June 18, 2003)

  More tools implemented.
- **Milestone 5** (Jule 2, 2003)

  Improvements and bugfixes with dependency scanning. Support for
  i18n tools. Testing framework for Boost.
- **Milestone 6** (Jule 22, 2003)

  Bugfixes.
- **Milestone 7** (Aug 1, 2003)

  Optimizations.
- **Milestone 8** (Oct 15, 2003)

  Implementation of regression testing framework was
  implemented. Handling of dependencies to generated headers was
  optimized. Algorithms for link-compatibility checking and selecting
  main target alternatives were improved.
- **Milestone 9.1** (Nov 06, 2003)

  Performance was considerably improved. The syntax for linking a
  library into a library was simplified. The 'stage' rule can traverse
  dependencies of the targets it installs. Regression testing support
  now works on Windows. Some toolsets were improved. Bugs in project
  loading and suffix determination were fixed.
- **Milestone 10** (TBD)

  Remaining features for C++ Boost:
  improved installation, Python support. Uniform toolset initialization
  scheme. Many bugfixes
- **Alpha** (TBD)

  Optimization. Using buildable tools: consider using bison, which
  is itself built with Boost.Build.
- **Beta** (TBD)

  Feature-complete milestone.
- **2.0 Release** (TBD)

  Bugfixes

---

© Copyright Vladimir Prus 2002-2003. Permission to copy, use,
modify, sell and distribute this document is granted provided this
copyright notice appears in all copies. This document is provided "as is"
without express or implied warranty, and with no claim as to its
suitability for any purpose.

Revised
23 Aug, 2004
