# Supplementary material for: Evaluation of de novo transcriptome assemblies from RNA-Seq data
Source: Genome Biol. 2014 Dec 21;15(12):553. doi: 10.1186/s13059-014-0553-5 (PMC4298084; doi:10.1186/s13059-014-0553-5)
Supplement: Additional file 3 — Version of the DETONATE source code used for the experiments in this paper. [file 13059_2014_553_MOESM3_ESM.zip › 13059_2014_553_MOESM3_ESM/detonate-1.8.1/ref-eval/boost/tools/build/v2/doc/tools.html]

Boost Build System V2 — supported tools

 

# Boost Build System V2 — supported tools

## Compilers

The following compilers are supported:

- GNU gcc (various versions), on Linux, NT, Cygwin and Darwin.
- Microsoft VC++ compiler (various version), on NT and Cygwin.
- Borland's compiler, on NT and Cygwin.
- Metrowerks Code Warrior, on NT.
- Intel C++ Compiler, on Linux and NT.
- Sun's C++ Compiler.

## Libraries

Some important libraries have special support in Boost.Build:

- STLport — on gcc and
  msvc.
- QT,
  including the moc and uic tools — on gcc and msvc.

## Other tools

- Parser/lexer generators bison and lex.
- XSLT processor xsltproc.
- The Apache FOP
  tool.
- BoostBook documentation format.
- GNU gettext
  internationalization tools.

---

© Copyright Vladimir Prus 2003. Permission to copy, use, modify,
sell and distribute this document is granted provided this copyright
notice appears in all copies. This document is provided "as is" without
express or implied warranty, and with no claim as to its suitability for
any purpose.

Revised
Jule 3, 2003
