# Supplementary material for: Evaluation of de novo transcriptome assemblies from RNA-Seq data
Source: Genome Biol. 2014 Dec 21;15(12):553. doi: 10.1186/s13059-014-0553-5 (PMC4298084; doi:10.1186/s13059-014-0553-5)
Supplement: Additional file 3 — Version of the DETONATE source code used for the experiments in this paper. [file 13059_2014_553_MOESM3_ESM.zip › 13059_2014_553_MOESM3_ESM/detonate-1.8.1/ref-eval/boost/tools/build/v2/engine/boehm_gc/doc/debugging.html]

Debugging Garbage Collector Related Problems


# Debugging Garbage Collector Related Problems

This page contains some hints on
debugging issues specific to
the Boehm-Demers-Weiser conservative garbage collector.
It applies both to debugging issues in client code that manifest themselves
as collector misbehavior, and to debugging the collector itself.

If you suspect a bug in the collector itself, it is strongly recommended
that you try the latest collector release, even if it is labelled as "alpha",
before proceeding.

## Bus Errors and Segmentation Violations

If the fault occurred in GC\_find\_limit, or with incremental collection enabled,
this is probably normal. The collector installs handlers to take care of
these. You will not see these unless you are using a debugger.
Your debugger *should* allow you to continue.
It's often preferable to tell the debugger to ignore SIGBUS and SIGSEGV
("handle SIGSEGV SIGBUS nostop noprint" in gdb,
"ignore SIGSEGV SIGBUS" in most versions of dbx)
and set a breakpoint in abort.
The collector will call abort if the signal had another cause,
and there was not other handler previously installed.

We recommend debugging without incremental collection if possible.
(This applies directly to UNIX systems.
Debugging with incremental collection under win32 is worse. See README.win32.)

If the application generates an unhandled SIGSEGV or equivalent, it may
often be easiest to set the environment variable GC\_LOOP\_ON\_ABORT. On many
platforms, this will cause the collector to loop in a handler when the
SIGSEGV is encountered (or when the collector aborts for some other reason),
and a debugger can then be attached to the looping
process. This sidesteps common operating system problems related
to incomplete core files for multithreaded applications, etc.

## Other Signals

On most platforms, the multithreaded version of the collector needs one or
two other signals for internal use by the collector in stopping threads.
It is normally wise to tell the debugger to ignore these. On Linux,
the collector currently uses SIGPWR and SIGXCPU by default.

## Warning Messages About Needing to Allocate Blacklisted Blocks

The garbage collector generates warning messages of the form

```
Needed to allocate blacklisted block at 0x...
```

or

```
Repeated allocation of very large block ...
```

when it needs to allocate a block at a location that it knows to be
referenced by a false pointer. These false pointers can be either permanent
(*e.g.* a static integer variable that never changes) or temporary.
In the latter case, the warning is largely spurious, and the block will
eventually be reclaimed normally.
In the former case, the program will still run correctly, but the block
will never be reclaimed. Unless the block is intended to be
permanent, the warning indicates a memory leak.

1. Ignore these warnings while you are using GC\_DEBUG. Some of the routines
   mentioned below don't have debugging equivalents. (Alternatively, write
   the missing routines and send them to me.)- Replace allocator calls that request large blocks with calls to
     GC\_malloc\_ignore\_off\_page or
     GC\_malloc\_atomic\_ignore\_off\_page. You may want to set a
     breakpoint in GC\_default\_warn\_proc to help you identify such calls.
     Make sure that a pointer to somewhere near the beginning of the resulting block
     is maintained in a (preferably volatile) variable as long as
     the block is needed.- If the large blocks are allocated with realloc, we suggest instead allocating
       them with something like the following. Note that the realloc size increment
       should be fairly large (e.g. a factor of 3/2) for this to exhibit reasonable
       performance. But we all know we should do that anyway.

       ```
       void * big_realloc(void *p, size_t new_size)
       {
           size_t old_size = GC_size(p);
           void * result;
        
           if (new_size <= 10000) return(GC_realloc(p, new_size));
           if (new_size <= old_size) return(p);
           result = GC_malloc_ignore_off_page(new_size);
           if (result == 0) return(0);
           memcpy(result,p,old_size);
           GC_free(p);
           return(result);
       }
       ```

       - In the unlikely case that even relatively small object
         (<20KB) allocations are triggering these warnings, then your address
         space contains lots of "bogus pointers", i.e. values that appear to
         be pointers but aren't. Usually this can be solved by using GC\_malloc\_atomic
         or the routines in gc\_typed.h to allocate large pointer-free regions of bitmaps, etc. Sometimes the problem can be solved with trivial changes of encoding
         in certain values. It is possible, to identify the source of the bogus
         pointers by building the collector with -DPRINT\_BLACK\_LIST,
         which will cause it to print the "bogus pointers", along with their location.- If you get only a fixed number of these warnings, you are probably only
           introducing a bounded leak by ignoring them. If the data structures being
           allocated are intended to be permanent, then it is also safe to ignore them.
           The warnings can be turned off by calling GC\_set\_warn\_proc with a procedure
           that ignores these warnings (e.g. by doing absolutely nothing).

## The Collector References a Bad Address in GC\_malloc

This typically happens while the collector is trying to remove an entry from
its free list, and the free list pointer is bad because the free list link
in the last allocated object was bad.

With > 99% probability, you wrote past the end of an allocated object.
Try setting GC\_DEBUG before including gc.h and
allocating with GC\_MALLOC. This will try to detect such
overwrite errors.

## Unexpectedly Large Heap

Unexpected heap growth can be due to one of the following:

1. Data structures that are being unintentionally retained. This
   is commonly caused by data structures that are no longer being used,
   but were not cleared, or by caches growing without bounds.- Pointer misidentification. The garbage collector is interpreting
     integers or other data as pointers and retaining the "referenced"
     objects. A common symptom is that GC\_dump() shows much of the heap
     as black-listed.- Heap fragmentation. This should never result in unbounded growth,
       but it may account for larger heaps. This is most commonly caused
       by allocation of large objects. On some platforms it can be reduced
       by building with -DUSE\_MUNMAP, which will cause the collector to unmap
       memory corresponding to pages that have not been recently used.- Per object overhead. This is usually a relatively minor effect, but
         it may be worth considering. If the collector recognizes interior
         pointers, object sizes are increased, so that one-past-the-end pointers
         are correctly recognized. The collector can be configured not to do this
         (-DDONT\_ADD\_BYTE\_AT\_END).

         The collector rounds up object sizes so the result fits well into the
         chunk size (HBLKSIZE, normally 4K on 32 bit machines, 8K
         on 64 bit machines) used by the collector. Thus it may be worth avoiding
         objects of size 2K + 1 (or 2K if a byte is being added at the end.)

The last two cases can often be identified by looking at the output
of a call to GC\_dump(). Among other things, it will print the
list of free heap blocks, and a very brief description of all chunks in
the heap, the object sizes they correspond to, and how many live objects
were found in the chunk at the last collection.

Growing data structures can usually be identified by

1. Building the collector with -DKEEP\_BACK\_PTRS,- Preferably using debugging allocation (defining GC\_DEBUG
     before including gc.h and allocating with GC\_MALLOC),
     so that objects will be identified by their allocation site,- Running the application long enough so
       that most of the heap is composed of "leaked" memory, and- Then calling GC\_generate\_random\_backtrace() from backptr.h
         a few times to determine why some randomly sampled objects in the heap are
         being retained.

The same technique can often be used to identify problems with false
pointers, by noting whether the reference chains printed by
GC\_generate\_random\_backtrace() involve any misidentified pointers.
An alternate technique is to build the collector with
-DPRINT\_BLACK\_LIST which will cause it to report values that
are almost, but not quite, look like heap pointers. It is very likely that
actual false pointers will come from similar sources.

In the unlikely case that false pointers are an issue, it can usually
be resolved using one or more of the following techniques:

1. Use GC\_malloc\_atomic for objects containing no pointers.
   This is especially important for large arrays containing compressed data,
   pseudo-random numbers, and the like. It is also likely to improve GC
   performance, perhaps drastically so if the application is paging.- If you allocate large objects containing only
     one or two pointers at the beginning, either try the typed allocation
     primitives is gc\_typed.h, or separate out the pointerfree component.- Consider using GC\_malloc\_ignore\_off\_page()
       to allocate large objects. (See gc.h and above for details.
       Large means > 100K in most environments.)- If your heap size is larger than 100MB or so, build the collector with
         -DLARGE\_CONFIG. This allows the collector to keep more precise black-list
         information.- If you are using heaps close to, or larger than, a gigabyte on a 32-bit
           machine, you may want to consider moving to a platform with 64-bit pointers.
           This is very likely to resolve any false pointer issues.

## Prematurely Reclaimed Objects

The usual symptom of this is a segmentation fault, or an obviously overwritten
value in a heap object. This should, of course, be impossible. In practice,
it may happen for reasons like the following:

1. The collector did not intercept the creation of threads correctly in
   a multithreaded application, *e.g.* because the client called
   pthread\_create without including gc.h, which redefines it.- The last pointer to an object in the garbage collected heap was stored
     somewhere were the collector couldn't see it, *e.g.* in an
     object allocated with system malloc, in certain types of
     mmaped files,
     or in some data structure visible only to the OS. (On some platforms,
     thread-local storage is one of these.)- The last pointer to an object was somehow disguised, *e.g.* by
       XORing it with another pointer.- Incorrect use of GC\_malloc\_atomic or typed allocation.- An incorrect GC\_free call.- The client program overwrote an internal garbage collector data structure.- A garbage collector bug.- (Empirically less likely than any of the above.) A compiler optimization
                 that disguised the last pointer.

The following relatively simple techniques should be tried first to narrow
down the problem:

1. If you are using the incremental collector try turning it off for
   debugging.- If you are using shared libraries, try linking statically. If that works,
     ensure that DYNAMIC\_LOADING is defined on your platform.- Try to reproduce the problem with fully debuggable unoptimized code.
       This will eliminate the last possibility, as well as making debugging easier.- Try replacing any suspect typed allocation and GC\_malloc\_atomic
         calls with calls to GC\_malloc.- Try removing any GC\_free calls (*e.g.* with a suitable
           #define).- Rebuild the collector with -DGC\_ASSERTIONS.- If the following works on your platform (i.e. if gctest still works
               if you do this), try building the collector with
               -DREDIRECT\_MALLOC=GC\_malloc\_uncollectable. This will cause
               the collector to scan memory allocated with malloc.

If all else fails, you will have to attack this with a debugger.
Suggested steps:

1. Call GC\_dump() from the debugger around the time of the failure. Verify
   that the collectors idea of the root set (i.e. static data regions which
   it should scan for pointers) looks plausible. If not, i.e. if it doesn't
   include some static variables, report this as
   a collector bug. Be sure to describe your platform precisely, since this sort
   of problem is nearly always very platform dependent.- Especially if the failure is not deterministic, try to isolate it to
     a relatively small test case.- Set a break point in GC\_finish\_collection. This is a good
       point to examine what has been marked, i.e. found reachable, by the
       collector.- If the failure is deterministic, run the process
         up to the last collection before the failure.
         Note that the variable GC\_gc\_no counts collections and can be used
         to set a conditional breakpoint in the right one. It is incremented just
         before the call to GC\_finish\_collection.
         If object p was prematurely recycled, it may be helpful to
         look at \*GC\_find\_header(p) at the failure point.
         The hb\_last\_reclaimed field will identify the collection number
         during which its block was last swept.- Verify that the offending object still has its correct contents at
           this point.
           Then call GC\_is\_marked(p) from the debugger to verify that the
           object has not been marked, and is about to be reclaimed. Note that
           GC\_is\_marked(p) expects the real address of an object (the
           address of the debug header if there is one), and thus it may
           be more appropriate to call GC\_is\_marked(GC\_base(p))
           instead.- Determine a path from a root, i.e. static variable, stack, or
             register variable,
             to the reclaimed object. Call GC\_is\_marked(q) for each object
             q along the path, trying to locate the first unmarked object, say
             r.- If r is pointed to by a static root,
               verify that the location
               pointing to it is part of the root set printed by GC\_dump(). If it
               is on the stack in the main (or only) thread, verify that
               GC\_stackbottom is set correctly to the base of the stack. If it is
               in another thread stack, check the collector's thread data structure
               (GC\_thread[] on several platforms) to make sure that stack bounds
               are set correctly.- If r is pointed to by heap object s, check that the
                 collector's layout description for s is such that the pointer field
                 will be scanned. Call \*GC\_find\_header(s) to look at the descriptor
                 for the heap chunk. The hb\_descr field specifies the layout
                 of objects in that chunk. See gc\_mark.h for the meaning of the descriptor.
                 (If it's low order 2 bits are zero, then it is just the length of the
                 object prefix to be scanned. This form is always used for objects allocated
                 with GC\_malloc or GC\_malloc\_atomic.)- If the failure is not deterministic, you may still be able to apply some
                   of the above technique at the point of failure. But remember that objects
                   allocated since the last collection will not have been marked, even if the
                   collector is functioning properly. On some platforms, the collector
                   can be configured to save call chains in objects for debugging.
                   Enabling this feature will also cause it to save the call stack at the
                   point of the last GC in GC\_arrays.\_last\_stack.- When looking at GC internal data structures remember that a number
                     of GC\_*xxx* variables are really macro defined to
                     GC\_arrays.\_*xxx*, so that
                     the collector can avoid scanning them.
