# Supplementary material for: Evaluation of de novo transcriptome assemblies from RNA-Seq data
Source: Genome Biol. 2014 Dec 21;15(12):553. doi: 10.1186/s13059-014-0553-5 (PMC4298084; doi:10.1186/s13059-014-0553-5)
Supplement: Additional file 3 — Version of the DETONATE source code used for the experiments in this paper. [file 13059_2014_553_MOESM3_ESM.zip › 13059_2014_553_MOESM3_ESM/detonate-1.8.1/ref-eval/boost/tools/build/v2/engine/boehm_gc/doc/gcdescr.html]

 Conservative GC Algorithmic Overview 
 Hans-J. Boehm, HP Labs (Some of this was written at SGI)


# *This is under construction, and may always be.*

# Conservative GC Algorithmic Overview

This is a description of the algorithms and data structures used in our
conservative garbage collector. I expect the level of detail to increase
with time. For a survey of GC algorithms, see for example
 Paul Wilson's
excellent paper. For an overview of the collector interface,
see here.

This description is targeted primarily at someone trying to understand the
source code. It specifically refers to variable and function names.
It may also be useful for understanding the algorithms at a higher level.

The description here assumes that the collector is used in default mode.
In particular, we assume that it used as a garbage collector, and not just
a leak detector. We initially assume that it is used in stop-the-world,
non-incremental mode, though the presence of the incremental collector
will be apparent in the design.
We assume the default finalization model, but the code affected by that
is very localized.

## Introduction

The garbage collector uses a modified mark-sweep algorithm. Conceptually
it operates roughly in four phases, which are performed occasionally
as part of a memory allocation:

1. *Preparation* Each object has an associated mark bit.
   Clear all mark bits, indicating that all objects
   are potentially unreachable.- *Mark phase* Marks all objects that can be reachable via chains of
     pointers from variables. Often the collector has no real information
     about the location of pointer variables in the heap, so it
     views all static data areas, stacks and registers as potentially containing
     pointers. Any bit patterns that represent addresses inside
     heap objects managed by the collector are viewed as pointers.
     Unless the client program has made heap object layout information
     available to the collector, any heap objects found to be reachable from
     variables are again scanned similarly.- *Sweep phase* Scans the heap for inaccessible, and hence unmarked,
       objects, and returns them to an appropriate free list for reuse. This is
       not really a separate phase; even in non incremental mode this is operation
       is usually performed on demand during an allocation that discovers an empty
       free list. Thus the sweep phase is very unlikely to touch a page that
       would not have been touched shortly thereafter anyway.- *Finalization phase* Unreachable objects which had been registered
         for finalization are enqueued for finalization outside the collector.

The remaining sections describe the memory allocation data structures,
and then the last 3 collection phases in more detail. We conclude by
outlining some of the additional features implemented in the collector.

## Allocation

The collector includes its own memory allocator. The allocator obtains
memory from the system in a platform-dependent way. Under UNIX, it
uses either malloc, sbrk, or mmap.

Most static data used by the allocator, as well as that needed by the
rest of the garbage collector is stored inside the
\_GC\_arrays structure.
This allows the garbage collector to easily ignore the collectors own
data structures when it searches for root pointers. Other allocator
and collector internal data structures are allocated dynamically
with GC\_scratch\_alloc. GC\_scratch\_alloc does not
allow for deallocation, and is therefore used only for permanent data
structures.

The allocator allocates objects of different *kinds*.
Different kinds are handled somewhat differently by certain parts
of the garbage collector. Certain kinds are scanned for pointers,
others are not. Some may have per-object type descriptors that
determine pointer locations. Or a specific kind may correspond
to one specific object layout. Two built-in kinds are uncollectable.
One (STUBBORN) is immutable without special precautions.
In spite of that, it is very likely that most C clients of the
collector currently
use at most two kinds: NORMAL and PTRFREE objects.
The gcj runtime also makes
heavy use of a kind (allocated with GC\_gcj\_malloc) that stores
type information at a known offset in method tables.

The collector uses a two level allocator. A large block is defined to
be one larger than half of HBLKSIZE, which is a power of 2,
typically on the order of the page size.

Large block sizes are rounded up to
the next multiple of HBLKSIZE and then allocated by
GC\_allochblk. Recent versions of the collector
use an approximate best fit algorithm by keeping free lists for
several large block sizes.
The actual
implementation of GC\_allochblk
is significantly complicated by black-listing issues
(see below).

Small blocks are allocated in chunks of size HBLKSIZE.
Each chunk is
dedicated to only one object size and kind. The allocator maintains
separate free lists for each size and kind of object.

Once a large block is split for use in smaller objects, it can only
be used for objects of that size, unless the collector discovers a completely
empty chunk. Completely empty chunks are restored to the appropriate
large block free list.

In order to avoid allocating blocks for too many distinct object sizes,
the collector normally does not directly allocate objects of every possible
request size. Instead request are rounded up to one of a smaller number
of allocated sizes, for which free lists are maintained. The exact
allocated sizes are computed on demand, but subject to the constraint
that they increase roughly in geometric progression. Thus objects
requested early in the execution are likely to be allocated with exactly
the requested size, subject to alignment constraints.
See GC\_init\_size\_map for details.

The actual size rounding operation during small object allocation is
implemented as a table lookup in GC\_size\_map.

Both collector initialization and computation of allocated sizes are
handled carefully so that they do not slow down the small object fast
allocation path. An attempt to allocate before the collector is initialized,
or before the appropriate GC\_size\_map entry is computed,
will take the same path as an allocation attempt with an empty free list.
This results in a call to the slow path code (GC\_generic\_malloc\_inner)
which performs the appropriate initialization checks.

In non-incremental mode, we make a decision about whether to garbage collect
whenever an allocation would otherwise have failed with the current heap size.
If the total amount of allocation since the last collection is less than
the heap size divided by GC\_free\_space\_divisor, we try to
expand the heap. Otherwise, we initiate a garbage collection. This ensures
that the amount of garbage collection work per allocated byte remains
constant.

The above is in fact an oversimplification of the real heap expansion
and GC triggering heuristic, which adjusts slightly for root size
and certain kinds of
fragmentation. In particular:

- Programs with a large root set size and
  little live heap memory will expand the heap to amortize the cost of
  scanning the roots.- Versions 5.x of the collector actually collect more frequently in
    nonincremental mode. The large block allocator usually refuses to split
    large heap blocks once the garbage collection threshold is
    reached. This often has the effect of collecting well before the
    heap fills up, thus reducing fragmentation and working set size at the
    expense of GC time. Versions 6.x choose an intermediate strategy depending
    on how much large object allocation has taken place in the past.
    (If the collector is configured to unmap unused pages, versions 6.x
    use the 5.x strategy.)- In calculating the amount of allocation since the last collection we
      give partial credit for objects we expect to be explicitly deallocated.
      Even if all objects are explicitly managed, it is often desirable to collect
      on rare occasion, since that is our only mechanism for coalescing completely
      empty chunks.

It has been suggested that this should be adjusted so that we favor
expansion if the resulting heap still fits into physical memory.
In many cases, that would no doubt help. But it is tricky to do this
in a way that remains robust if multiple application are contending
for a single pool of physical memory.

## Mark phase

At each collection, the collector marks all objects that are
possibly reachable from pointer variables. Since it cannot generally
tell where pointer variables are located, it scans the following
*root segments* for pointers:

- The registers. Depending on the architecture, this may be done using
  assembly code, or by calling a setjmp-like function which saves
  register contents on the stack.- The stack(s). In the case of a single-threaded application,
    on most platforms this
    is done by scanning the memory between (an approximation of) the current
    stack pointer and GC\_stackbottom. (For Itanium, the register stack
    scanned separately.) The GC\_stackbottom variable is set in
    a highly platform-specific way depending on the appropriate configuration
    information in gcconfig.h. Note that the currently active
    stack needs to be scanned carefully, since callee-save registers of
    client code may appear inside collector stack frames, which may
    change during the mark process. This is addressed by scanning
    some sections of the stack "eagerly", effectively capturing a snapshot
    at one point in time.- Static data region(s). In the simplest case, this is the region
      between DATASTART and DATAEND, as defined in
      gcconfig.h. However, in most cases, this will also involve
      static data regions associated with dynamic libraries. These are
      identified by the mostly platform-specific code in dyn\_load.c.

The marker maintains an explicit stack of memory regions that are known
to be accessible, but that have not yet been searched for contained pointers.
Each stack entry contains the starting address of the block to be scanned,
as well as a descriptor of the block. If no layout information is
available for the block, then the descriptor is simply a length.
(For other possibilities, see gc\_mark.h.)

At the beginning of the mark phase, all root segments
(as described above) are pushed on the
stack by GC\_push\_roots. (Registers and eagerly processed
stack sections are processed by pushing the referenced objects instead
of the stack section itself.) If ALL\_INTERIOR\_PTRS is not
defined, then stack roots require special treatment. In this case, the
normal marking code ignores interior pointers, but GC\_push\_all\_stack
explicitly checks for interior pointers and pushes descriptors for target
objects.

The marker is structured to allow incremental marking.
Each call to GC\_mark\_some performs a small amount of
work towards marking the heap.
It maintains
explicit state in the form of GC\_mark\_state, which
identifies a particular sub-phase. Some other pieces of state, most
notably the mark stack, identify how much work remains to be done
in each sub-phase. The normal progression of mark states for
a stop-the-world collection is:

1. MS\_INVALID indicating that there may be accessible unmarked
   objects. In this case GC\_objects\_are\_marked will simultaneously
   be false, so the mark state is advanced to- MS\_PUSH\_UNCOLLECTABLE indicating that it suffices to push
     uncollectable objects, roots, and then mark everything reachable from them.
     Scan\_ptr is advanced through the heap until all uncollectable
     objects are pushed, and objects reachable from them are marked.
     At that point, the next call to GC\_mark\_some calls
     GC\_push\_roots to push the roots. It the advances the
     mark state to- MS\_ROOTS\_PUSHED asserting that once the mark stack is
       empty, all reachable objects are marked. Once in this state, we work
       only on emptying the mark stack. Once this is completed, the state
       changes to- MS\_NONE indicating that reachable objects are marked.

The core mark routine GC\_mark\_from, is called
repeatedly by several of the sub-phases when the mark stack starts to fill
up. It is also called repeatedly in MS\_ROOTS\_PUSHED state
to empty the mark stack.
The routine is designed to only perform a limited amount of marking at
each call, so that it can also be used by the incremental collector.
It is fairly carefully tuned, since it usually consumes a large majority
of the garbage collection time.

The fact that it perform a only a small amount of work per call also
allows it to be used as the core routine of the parallel marker. In that
case it is normally invoked on thread-private mark stacks instead of the
global mark stack. More details can be found in
scale.html

The marker correctly handles mark stack overflows. Whenever the mark stack
overflows, the mark state is reset to MS\_INVALID.
Since there are already marked objects in the heap,
this eventually forces a complete
scan of the heap, searching for pointers, during which any unmarked objects
referenced by marked objects are again pushed on the mark stack. This
process is repeated until the mark phase completes without a stack overflow.
Each time the stack overflows, an attempt is made to grow the mark stack.
All pieces of the collector that push regions onto the mark stack have to be
careful to ensure forward progress, even in case of repeated mark stack
overflows. Every mark attempt results in additional marked objects.

Each mark stack entry is processed by examining all candidate pointers
in the range described by the entry. If the region has no associated
type information, then this typically requires that each 4-byte aligned
quantity (8-byte aligned with 64-bit pointers) be considered a candidate
pointer.

We determine whether a candidate pointer is actually the address of
a heap block. This is done in the following steps:
- The candidate pointer is checked against rough heap bounds.
  These heap bounds are maintained such that all actual heap objects
  fall between them. In order to facilitate black-listing (see below)
  we also include address regions that the heap is likely to expand into.
  Most non-pointers fail this initial test.- The candidate pointer is divided into two pieces; the most significant
    bits identify a HBLKSIZE-sized page in the address space, and
    the least significant bits specify an offset within that page.
    (A hardware page may actually consist of multiple such pages.
    HBLKSIZE is usually the page size divided by a small power of two.)- The page address part of the candidate pointer is looked up in a
      table.
      Each table entry contains either 0, indicating that the page is not part
      of the garbage collected heap, a small integer *n*, indicating
      that the page is part of large object, starting at least *n* pages
      back, or a pointer to a descriptor for the page. In the first case,
      the candidate pointer i not a true pointer and can be safely ignored.
      In the last two cases, we can obtain a descriptor for the page containing
      the beginning of the object.- The starting address of the referenced object is computed.
        The page descriptor contains the size of the object(s)
        in that page, the object kind, and the necessary mark bits for those
        objects. The size information can be used to map the candidate pointer
        to the object starting address. To accelerate this process, the page header
        also contains a pointer to a precomputed map of page offsets to displacements
        from the beginning of an object. The use of this map avoids a
        potentially slow integer remainder operation in computing the object
        start address.- The mark bit for the target object is checked and set. If the object
          was previously unmarked, the object is pushed on the mark stack.
          The descriptor is read from the page descriptor. (This is computed
          from information GC\_obj\_kinds when the page is first allocated.)

At the end of the mark phase, mark bits for left-over free lists are cleared,
in case a free list was accidentally marked due to a stray pointer.

## Sweep phase

At the end of the mark phase, all blocks in the heap are examined.
Unmarked large objects are immediately returned to the large object free list.
Each small object page is checked to see if all mark bits are clear.
If so, the entire page is returned to the large object free list.
Small object pages containing some reachable object are queued for later
sweeping, unless we determine that the page contains very little free
space, in which case it is not examined further.

This initial sweep pass touches only block headers, not
the blocks themselves. Thus it does not require significant paging, even
if large sections of the heap are not in physical memory.

Nonempty small object pages are swept when an allocation attempt
encounters an empty free list for that object size and kind.
Pages for the correct size and kind are repeatedly swept until at
least one empty block is found. Sweeping such a page involves
scanning the mark bit array in the page header, and building a free
list linked through the first words in the objects themselves.
This does involve touching the appropriate data page, but in most cases
it will be touched only just before it is used for allocation.
Hence any paging is essentially unavoidable.

Except in the case of pointer-free objects, we maintain the invariant
that any object in a small object free list is cleared (except possibly
for the link field). Thus it becomes the burden of the small object
sweep routine to clear objects. This has the advantage that we can
easily recover from accidentally marking a free list, though that could
also be handled by other means. The collector currently spends a fair
amount of time clearing objects, and this approach should probably be
revisited.

In most configurations, we use specialized sweep routines to handle common
small object sizes. Since we allocate one mark bit per word, it becomes
easier to examine the relevant mark bits if the object size divides
the word length evenly. We also suitably unroll the inner sweep loop
in each case. (It is conceivable that profile-based procedure cloning
in the compiler could make this unnecessary and counterproductive. I
know of no existing compiler to which this applies.)

The sweeping of small object pages could be avoided completely at the expense
of examining mark bits directly in the allocator. This would probably
be more expensive, since each allocation call would have to reload
a large amount of state (e.g. next object address to be swept, position
in mark bit table) before it could do its work. The current scheme
keeps the allocator simple and allows useful optimizations in the sweeper.

## Finalization

Both GC\_register\_disappearing\_link and
GC\_register\_finalizer add the request to a corresponding hash
table. The hash table is allocated out of collected memory, but
the reference to the finalizable object is hidden from the collector.
Currently finalization requests are processed non-incrementally at the
end of a mark cycle.

The collector makes an initial pass over the table of finalizable objects,
pushing the contents of unmarked objects onto the mark stack.
After pushing each object, the marker is invoked to mark all objects
reachable from it. The object itself is not explicitly marked.
This assures that objects on which a finalizer depends are neither
collected nor finalized.

If in the process of marking from an object the
object itself becomes marked, we have uncovered
a cycle involving the object. This usually results in a warning from the
collector. Such objects are not finalized, since it may be
unsafe to do so. See the more detailed
 discussion of finalization semantics.

Any objects remaining unmarked at the end of this process are added to
a queue of objects whose finalizers can be run. Depending on collector
configuration, finalizers are dequeued and run either implicitly during
allocation calls, or explicitly in response to a user request.
(Note that the former is unfortunately both the default and not generally safe.
If finalizers perform synchronization, it may result in deadlocks.
Nontrivial finalizers generally need to perform synchronization, and
thus require a different collector configuration.)

The collector provides a mechanism for replacing the procedure that is
used to mark through objects. This is used both to provide support for
Java-style unordered finalization, and to ignore certain kinds of cycles,
*e.g.* those arising from C++ implementations of virtual inheritance.

## Generational Collection and Dirty Bits

We basically use the concurrent and generational GC algorithm described in
"Mostly Parallel Garbage Collection",
by Boehm, Demers, and Shenker.

The most significant modification is that
the collector always starts running in the allocating thread.
There is no separate garbage collector thread. (If parallel GC is
enabled, helper threads may also be woken up.)
If an allocation attempt either requests a large object, or encounters
an empty small object free list, and notices that there is a collection
in progress, it immediately performs a small amount of marking work
as described above.

This change was made both because we wanted to easily accommodate
single-threaded environments, and because a separate GC thread requires
very careful control over the scheduler to prevent the mutator from
out-running the collector, and hence provoking unneeded heap growth.

In incremental mode, the heap is always expanded when we encounter
insufficient space for an allocation. Garbage collection is triggered
whenever we notice that more than
GC\_heap\_size/2 \* GC\_free\_space\_divisor
bytes of allocation have taken place.
After GC\_full\_freq minor collections a major collection
is started.

All collections initially run interrupted until a predetermined
amount of time (50 msecs by default) has expired. If this allows
the collection to complete entirely, we can avoid correcting
for data structure modifications during the collection. If it does
not complete, we return control to the mutator, and perform small
amounts of additional GC work during those later allocations that
cannot be satisfied from small object free lists. When marking completes,
the set of modified pages is retrieved, and we mark once again from
marked objects on those pages, this time with the mutator stopped.

We keep track of modified pages using one of several distinct mechanisms:

1. Through explicit mutator cooperation. Currently this requires
   the use of GC\_malloc\_stubborn, and is rarely used.- (MPROTECT\_VDB) By write-protecting physical pages and
     catching write faults. This is
     implemented for many Unix-like systems and for win32. It is not possible
     in a few environments.- (PROC\_VDB) By retrieving dirty bit information from /proc.
       (Currently only Sun's
       Solaris supports this. Though this is considerably cleaner, performance
       may actually be better with mprotect and signals.)- (PCR\_VDB) By relying on an external dirty bit implementation, in this
         case the one in Xerox PCR.- (DEFAULT\_VDB) By treating all pages as dirty. This is the default if
           none of the other techniques is known to be usable, and
           GC\_malloc\_stubborn is not used. Practical only for testing, or if
           the vast majority of objects use GC\_malloc\_stubborn.

## Black-listing

The collector implements *black-listing* of pages, as described
in
Boehm, ``Space Efficient Conservative Collection'', PLDI '93, also available
here.

During the mark phase, the collector tracks ``near misses'', i.e. attempts
to follow a ``pointer'' to just outside the garbage-collected heap, or
to a currently unallocated page inside the heap. Pages that have been
the targets of such near misses are likely to be the targets of
misidentified ``pointers'' in the future. To minimize the future
damage caused by such misidentifications they will be allocated only to
small pointerfree objects.

The collector understands two different kinds of black-listing. A
page may be black listed for interior pointer references
(GC\_add\_to\_black\_list\_stack), if it was the target of a near
miss from a location that requires interior pointer recognition,
*e.g.* the stack, or the heap if GC\_all\_interior\_pointers
is set. In this case, we also avoid allocating large blocks that include
this page.

If the near miss came from a source that did not require interior
pointer recognition, it is black-listed with
GC\_add\_to\_black\_list\_normal.
A page black-listed in this way may appear inside a large object,
so long as it is not the first page of a large object.

The GC\_allochblk routine respects black-listing when assigning
a block to a particular object kind and size. It occasionally
drops (i.e. allocates and forgets) blocks that are completely black-listed
in order to avoid excessively long large block free lists containing
only unusable blocks. This would otherwise become an issue
if there is low demand for small pointerfree objects.

## Thread support

We support several different threading models. Unfortunately Pthreads,
the only reasonably well standardized thread model, supports too narrow
an interface for conservative garbage collection. There appears to be
no completely portable way to allow the collector
to coexist with various Pthreads
implementations. Hence we currently support only the more
common Pthreads implementations.

In particular, it is very difficult for the collector to stop all other
threads in the system and examine the register contents. This is currently
accomplished with very different mechanisms for some Pthreads
implementations. The Solaris implementation temporarily disables much
of the user-level threads implementation by stopping kernel-level threads
("lwp"s). The Linux/HPUX/OSF1 and Irix implementations sends signals to
individual Pthreads and has them wait in the signal handler.

The Linux and Irix implementations use
only documented Pthreads calls, but rely on extensions to their semantics.
The Linux implementation linux\_threads.c relies on only very
mild extensions to the pthreads semantics, and already supports a large number
of other Unix-like pthreads implementations. Our goal is to make this the
only pthread support in the collector.

(The Irix implementation is separate only for historical reasons and should
clearly be merged. The current Solaris implementation probably performs
better in the uniprocessor case, but does not support thread operations in the
collector. Hence it cannot support the parallel marker.)

All implementations must
intercept thread creation and a few other thread-specific calls to allow
enumeration of threads and location of thread stacks. This is current
accomplished with # define's in gc.h
(really gc\_pthread\_redirects.h), or optionally
by using ld's function call wrapping mechanism under Linux.

Recent versions of the collector support several facilites to enhance
the processor-scalability and thread performance of the collector.
These are discussed in more detail here.
We briefly outline the data approach to thread-local allocation in the
next section.

## Thread-local allocation

If thread-local allocation is enabled, the collector keeps separate
arrays of free lists for each thread. Thread-local allocation
is currently only supported on a few platforms.

The free list arrays associated
with each thread are only used to satisfy requests for objects that
are both very small, and belong to one of a small number of well-known
kinds. These currently include "normal" and pointer-free objects.
Depending onthe configuration, "gcj" objects may also be included.

Thread-local free list entries contain either a pointer to the first
element of a free list, or they contain a counter of the number of
allocation "granules" allocated so far. Initially they contain the
value one, i.e. a small counter value.

Thread-local allocation allocates directly through the global
allocator, if the object is of a size or kind not covered by the
local free lists.

If there is an appropriate local free list, the allocator checks whether it
contains a sufficiently small counter value. If so, the counter is simply
incremented by the counter value, and the global allocator is used.
In this way, the initial few allocations of a given size bypass the local
allocator. A thread that only allocates a handful of objects of a given
size will not build up its own free list for that size. This avoids
wasting space for unpopular objects sizes or kinds.

Once the counter passes a threshold, GC\_malloc\_many is called
to allocate roughly HBLKSIZE space and put it on the corresponding
local free list. Further allocations of that size and kind then use
this free list, and no longer need to acquire the allocation lock.
The allocation procedure is otherwise similar to the global free lists.
The local free lists are also linked using the first word in the object.
In most cases this means they require considerably less time.

Local free lists are treated buy most of the rest of the collector
as though they were in-use reachable data. This requires some care,
since pointer-free objects are not normally traced, and hence a special
tracing procedure is required to mark all objects on pointer-free and
gcj local free lists.

On thread exit, any remaining thread-local free list entries are
transferred back to the global free list.

Note that if the collector is configured for thread-local allocation,
GC versions before 7 do not invoke the thread-local allocator by default.
GC\_malloc only uses thread-local allocation in version 7 and later.
In earlier versions, GC\_MALLOC (all caps) may be directed
to use thread-local allocation by defining GC\_REDIRECT\_TO\_LOCAL
and then include gc\_local\_alloc.h.

For some more details see here, and the
technical report entitled
``Fast Multiprocessor Memory Allocation and Garbage Collection''

---

Comments are appreciated. Please send mail to
boehm@acm.org or
Hans.Boehm@hp.com

This is a modified copy of a page written while the author was at SGI.
The original was here.
