# Supplementary material for: Evaluation of de novo transcriptome assemblies from RNA-Seq data
Source: Genome Biol. 2014 Dec 21;15(12):553. doi: 10.1186/s13059-014-0553-5 (PMC4298084; doi:10.1186/s13059-014-0553-5)
Supplement: Additional file 3 — Version of the DETONATE source code used for the experiments in this paper. [file 13059_2014_553_MOESM3_ESM.zip › 13059_2014_553_MOESM3_ESM/detonate-1.8.1/ref-eval/boost/tools/build/v2/engine/boehm_gc/doc/gcinterface.html]

Garbage Collector Interface


# C Interface

On many platforms, a single-threaded garbage collector library can be built
to act as a plug-in malloc replacement.
(Build with -DREDIRECT\_MALLOC=GC\_malloc -DIGNORE\_FREE.)
This is often the best way to deal with third-party libraries
which leak or prematurely free objects. -DREDIRECT\_MALLOC is intended
primarily as an easy way to adapt old code, not for new development.

New code should use the interface discussed below.

Code must be linked against the GC library. On most UNIX platforms,
depending on how the collector is built, this will be gc.a
or libgc.{a,so}.

The following describes the standard C interface to the garbage collector.
It is not a complete definition of the interface. It describes only the
most commonly used functionality, approximately in decreasing order of
frequency of use.
The full interface is described in
gc.h
or gc.h in the distribution.

Clients should include gc.h.

In the case of multithreaded code,
gc.h should be included after the threads header file, and
after defining the appropriate GC\_*XXXX*\_THREADS macro.
(For 6.2alpha4 and later, simply defining GC\_THREADS should suffice.)
The header file gc.h must be included
in files that use either GC or threads primitives, since threads primitives
will be redefined to cooperate with the GC on many platforms.

Thread users should also be aware that on many platforms objects reachable
only from thread-local variables may be prematurely reclaimed.
Thus objects pointed to by thread-local variables should also be pointed to
by a globally visible data structure. (This is viewed as a bug, but as
one that is exceedingly hard to fix without some libc hooks.)

**void \* GC\_MALLOC(size\_t *nbytes*)**: Allocates and clears *nbytes* of storage. Requires (amortized) time proportional to *nbytes*. The resulting object will be automatically deallocated when unreferenced. References from objects allocated with the system malloc are usually not considered by the collector. (See GC\_MALLOC\_UNCOLLECTABLE, however.) GC\_MALLOC is a macro which invokes GC\_malloc by default or, if GC\_DEBUG is defined before gc.h is included, a debugging version that checks occasionally for overwrite errors, and the like. **void \* GC\_MALLOC\_ATOMIC(size\_t *nbytes*)**: Allocates *nbytes* of storage. Requires (amortized) time proportional to *nbytes*. The resulting object will be automatically deallocated when unreferenced. The client promises that the resulting object will never contain any pointers. The memory is not cleared. This is the preferred way to allocate strings, floating point arrays, bitmaps, etc. More precise information about pointer locations can be communicated to the collector using the interface in gc\_typed.h in the distribution. **void \* GC\_MALLOC\_UNCOLLECTABLE(size\_t *nbytes*)**: Identical to GC\_MALLOC, except that the resulting object is not automatically deallocated. Unlike the system-provided malloc, the collector does scan the object for pointers to garbage-collectable memory, even if the block itself does not appear to be reachable. (Objects allocated in this way are effectively treated as roots by the collector.) **void \* GC\_REALLOC(void \**old*, size\_t *new\_size*)** : Allocate a new object of the indicated size and copy (a prefix of) the old object into the new object. The old object is reused in place if convenient. If the original object was allocated with GC\_MALLOC\_ATOMIC, the new object is subject to the same constraints. If it was allocated as an uncollectable object, then the new object is uncollectable, and the old object (if different) is deallocated. **void GC\_FREE(void \**dead*)** : Explicitly deallocate an object. Typically not useful for small collectable objects. **void \* GC\_MALLOC\_IGNORE\_OFF\_PAGE(size\_t *nbytes*)** : **void \* GC\_MALLOC\_ATOMIC\_IGNORE\_OFF\_PAGE(size\_t *nbytes*)** : Analogous to GC\_MALLOC and GC\_MALLOC\_ATOMIC, except that the client guarantees that as long as the resulting object is of use, a pointer is maintained to someplace inside the first 512 bytes of the object. This pointer should be declared volatile to avoid interference from compiler optimizations. (Other nonvolatile pointers to the object may exist as well.) This is the preferred way to allocate objects that are likely to be > 100KBytes in size. It greatly reduces the risk that such objects will be accidentally retained when they are no longer needed. Thus space usage may be significantly reduced. **void GC\_INIT(void)** : On some platforms, it is necessary to invoke this *from the main executable, not from a dynamic library,* before the initial invocation of a GC routine. It is recommended that this be done in portable code, though we try to ensure that it expands to a no-op on as many platforms as possible. As of GC 7.0, it is required if thread-local allocation is enabled in the collector build, and malloc is not redirected to GC\_malloc. **void GC\_gcollect(void)** : Explicitly force a garbage collection. **void GC\_enable\_incremental(void)** : Cause the garbage collector to perform a small amount of work every few invocations of GC\_MALLOC or the like, instead of performing an entire collection at once. This is likely to increase total running time. It will improve response on a platform that either has suitable support in the garbage collector (Linux and most Unix versions, win32 if the collector was suitably built) or if "stubborn" allocation is used (see gc.h). On many platforms this interacts poorly with system calls that write to the garbage collected heap. **GC\_warn\_proc GC\_set\_warn\_proc(GC\_warn\_proc *p*)** : Replace the default procedure used by the collector to print warnings. The collector may otherwise write to sterr, most commonly because GC\_malloc was used in a situation in which GC\_malloc\_ignore\_off\_page would have been more appropriate. See gc.h for details. **void GC\_REGISTER\_FINALIZER(...)** : Register a function to be called when an object becomes inaccessible. This is often useful as a backup method for releasing system resources (*e.g.* closing files) when the object referencing them becomes inaccessible. It is not an acceptable method to perform actions that must be performed in a timely fashion. See gc.h for details of the interface. See here for a more detailed discussion of the design. Note that an object may become inaccessible before client code is done operating on objects referenced by its fields. Suitable synchronization is usually required. See here or here for details.

If you are concerned with multiprocessor performance and scalability,
you should consider enabling and using thread local allocation (*e.g.*
GC\_LOCAL\_MALLOC, see gc\_local\_alloc.h. If your platform
supports it, you should build the collector with parallel marking support
(-DPARALLEL\_MARK, or --enable-parallel-mark).

If the collector is used in an environment in which pointer location
information for heap objects is easily available, this can be passed on
to the collector using the interfaces in either gc\_typed.h
or gc\_gcj.h.

The collector distribution also includes a **string package** that takes
advantage of the collector. For details see
cord.h

# C++ Interface

The C++ interface is implemented as a thin layer on the C interface.
Unfortunately, this thin layer appears to be very sensitive to variations
in C++ implementations, particularly since it tries to replace the global
::new operator, something that appears to not be well-standardized.
Your platform may need minor adjustments in this layer (gc\_cpp.cc, gc\_cpp.h,
and possibly gc\_allocator.h). Such changes do not require understanding
of collector internals, though they may require a good understanding of
your platform. (Patches enhancing portability are welcome.
But it's easy to break one platform by fixing another.)

Usage of the collector from C++ is also complicated by the fact that there
are many "standard" ways to allocate memory in C++. The default ::new
operator, default malloc, and default STL allocators allocate memory
that is not garbage collected, and is not normally "traced" by the
collector. This means that any pointers in memory allocated by these
default allocators will not be seen by the collector. Garbage-collectable
memory referenced only by pointers stored in such default-allocated
objects is likely to be reclaimed prematurely by the collector.

It is the programmers responsibility to ensure that garbage-collectable
memory is referenced by pointers stored in one of

- Program variables- Garbage-collected objects- Uncollected but "traceable" objects

"Traceable" objects are not necessarily reclaimed by the collector,
but are scanned for pointers to collectable objects.
They are usually allocated by GC\_MALLOC\_UNCOLLECTABLE, as described
above, and through some interfaces described below.

(On most platforms, the collector may not trace correctly from in-flight
exception objects. Thus objects thrown as exceptions should only
point to otherwise reachable memory. This is another bug whose
proper repair requires platform hooks.)

The easiest way to ensure that collectable objects are properly referenced
is to allocate only collectable objects. This requires that every
allocation go through one of the following interfaces, each one of
which replaces a standard C++ allocation mechanism:

**STL allocators** : Recent versions of the collector also include a more standard-conforming allocator implementation in gc\_allocator.h. It defines - traceable\_allocator- gc\_allocator which may be used either directly to allocate memory or to instantiate container templates. The former allocates uncollectable but traced memory. The latter allocates garbage-collected memory. These should work with any fully standard-conforming C++ compiler. Users of the SGI extended STL or its derivatives (including most g++ versions) can alternatively include new\_gc\_alloc.h before including STL header files. (gc\_alloc.h corresponds to now obsolete versions of the SGI STL.) This interface is no longer recommended, but it has existed for much longer. This defines SGI-style allocators - alloc- single\_client\_alloc- gc\_alloc- single\_client\_gc\_alloc The first two allocate uncollectable but traced memory, while the second two allocate collectable memory. The single\_client versions are not safe for concurrent access by multiple threads, but are faster. For an example, click here. **Class inheritance based interface** : Users may include gc\_cpp.h and then cause members of classes to be allocated in garbage collectable memory by having those classes inherit from class gc. For details see gc\_cpp.h. Linking against libgccpp in addition to the gc library overrides ::new (and friends) to allocate traceable memory but uncollectable memory, making it safe to refer to collectable objects from the resulting memory. **C interface** : It is also possible to use the C interface from gc.h directly. On platforms which use malloc to implement ::new, it should usually be possible to use a version of the collector that has been compiled as a malloc replacement. It is also possible to replace ::new and other allocation functions suitably, as is done by libgccpp. Note that user-implemented small-block allocation often works poorly with an underlying garbage-collected large block allocator, since the collector has to view all objects accessible from the user's free list as reachable. This is likely to cause problems if GC\_MALLOC is used with something like the original HP version of STL. This approach works well with the SGI versions of the STL only if the malloc\_alloc allocator is used.
