# Supplementary material for: Evaluation of de novo transcriptome assemblies from RNA-Seq data
Source: Genome Biol. 2014 Dec 21;15(12):553. doi: 10.1186/s13059-014-0553-5 (PMC4298084; doi:10.1186/s13059-014-0553-5)
Supplement: Additional file 3 — Version of the DETONATE source code used for the experiments in this paper. [file 13059_2014_553_MOESM3_ESM.zip › 13059_2014_553_MOESM3_ESM/detonate-1.8.1/ref-eval/boost/tools/build/v2/engine/boehm_gc/doc/leak.html]

Using the Garbage Collector as Leak Detector


# Using the Garbage Collector as Leak Detector

The garbage collector may be used as a leak detector.
In this case, the primary function of the collector is to report
objects that were allocated (typically with GC\_MALLOC),
not deallocated (normally with GC\_FREE), but are
no longer accessible. Since the object is no longer accessible,
there in normally no way to deallocate the object at a later time;
thus it can safely be assumed that the object has been "leaked".

This is substantially different from counting leak detectors,
which simply verify that all allocated objects are eventually
deallocated. A garbage-collector based leak detector can provide
somewhat more precise information when an object was leaked.
More importantly, it does not report objects that are never
deallocated because they are part of "permanent" data structures.
Thus it does not require all objects to be deallocated at process
exit time, a potentially useless activity that often triggers
large amounts of paging.

All non-ancient versions of the garbage collector provide
leak detection support. Version 5.3 adds the following
features:

1. Leak detection mode can be initiated at run-time by
   setting GC\_find\_leak instead of building the collector with FIND\_LEAK
   defined. This variable should be set to a nonzero value
   at program startup.- Leaked objects should be reported and then correctly garbage collected.
     Prior versions either reported leaks or functioned as a garbage collector.

For the rest of this description we will give instructions that work
with any reasonable version of the collector.

To use the collector as a leak detector, follow the following steps:

1. Build the collector with -DFIND\_LEAK. Otherwise use default
   build options.- Change the program so that all allocation and deallocation goes
     through the garbage collector.- Arrange to call GC\_gcollect at appropriate points to check
       for leaks.
       (For sufficiently long running programs, this will happen implicitly,
       but probably not with sufficient frequency.)

The second step can usually be accomplished with the
-DREDIRECT\_MALLOC=GC\_malloc option when the collector is built,
or by defining malloc, calloc,
realloc and free
to call the corresponding garbage collector functions.
But this, by itself, will not yield very informative diagnostics,
since the collector does not keep track of information about
how objects were allocated. The error reports will include
only object addresses.

For more precise error reports, as much of the program as possible
should use the all uppercase variants of these functions, after
defining GC\_DEBUG, and then including gc.h.
In this environment GC\_MALLOC is a macro which causes
at least the file name and line number at the allocation point to
be saved as part of the object. Leak reports will then also include
this information.

Many collector features (*e.g* stubborn objects, finalization,
and disappearing links) are less useful in this context, and are not
fully supported. Their use will usually generate additional bogus
leak reports, since the collector itself drops some associated objects.

The same is generally true of thread support. However, as of 6.0alpha4,
correct leak reports should be generated with linuxthreads.

On a few platforms (currently Solaris/SPARC, Irix, and, with -DSAVE\_CALL\_CHAIN,
Linux/X86), GC\_MALLOC
also causes some more information about its call stack to be saved
in the object. Such information is reproduced in the error
reports in very non-symbolic form, but it can be very useful with the
aid of a debugger.

## An Example

The following header file leak\_detector.h is included in the
"include" subdirectory of the distribution:

```
#define GC_DEBUG
#include "gc.h"
#define malloc(n) GC_MALLOC(n)
#define calloc(m,n) GC_MALLOC((m)*(n))
#define free(p) GC_FREE(p)
#define realloc(p,n) GC_REALLOC((p),(n))
#define CHECK_LEAKS() GC_gcollect()
```

Assume the collector has been built with -DFIND\_LEAK. (For very
new versions of the collector, we could instead add the statement
GC\_find\_leak = 1 as the first statement in main.

The program to be tested for leaks can then look like:

```
#include "leak_detector.h"

main() {
    int *p[10];
    int i;
    /* GC_find_leak = 1; for new collector versions not 	*/
    /* compiled with -DFIND_LEAK.				*/
    for (i = 0; i < 10; ++i) {
	p[i] = malloc(sizeof(int)+i);
    }
    for (i = 1; i < 10; ++i) {
	free(p[i]);
    }
    for (i = 0; i < 9; ++i) {
	p[i] = malloc(sizeof(int)+i);
    }
    CHECK_LEAKS();
}	
```

On an Intel X86 Linux system this produces on the stderr stream:

```
Leaked composite object at 0x806dff0 (leak_test.c:8, sz=4)
```

(On most unmentioned operating systems, the output is similar to this.
If the collector had been built on Linux/X86 with -DSAVE\_CALL\_CHAIN,
the output would be closer to the Solaris example. For this to work,
the program should not be compiled with -fomit\_frame\_pointer.)

On Irix it reports

```
Leaked composite object at 0x10040fe0 (leak_test.c:8, sz=4)
        Caller at allocation:
                ##PC##= 0x10004910
```

and on Solaris the error report is

```
Leaked composite object at 0xef621fc8 (leak_test.c:8, sz=4)
        Call chain at allocation:
                args: 4 (0x4), 200656 (0x30FD0)
                ##PC##= 0x14ADC
                args: 1 (0x1), -268436012 (0xEFFFFDD4)
                ##PC##= 0x14A64
```

In the latter two cases some additional information is given about
how malloc was called when the leaked object was allocated. For
Solaris, the first line specifies the arguments to GC\_debug\_malloc
(the actual allocation routine), The second the program counter inside
main, the third the arguments to main, and finally the program
counter inside the caller to main (i.e. in the C startup code).

In the Irix case, only the address inside the caller to main is given.

In many cases, a debugger is needed to interpret the additional information.
On systems supporting the "adb" debugger, the callprocs script
can be used to replace program counter values with symbolic names.
As of version 6.1, the collector tries to generate symbolic names for
call stacks if it knows how to do so on the platform. This is true on
Linux/X86, but not on most other platforms.

## Simplified leak detection under Linux

Since version 6.1, it should be possible to run the collector in leak
detection mode on a program a.out under Linux/X86 as follows:

1. *Ensure that a.out is a single-threaded executable, or you are using
   a very recent (7.0alpha7+) collector version on Linux.*
   On most platforms this does not
   work at all for multithreaded programs.- If possible, ensure that the addr2line program is installed in
     /usr/bin. (It comes with RedHat Linux.)- If possible, compile a.out with full debug information.
       This will improve the quality of the leak reports. With this approach, it is
       no longer necessary to call GC\_ routines explicitly, though that can also
       improve the quality of the leak reports.- Build the collector and install it in directory *foo* as follows:
         - configure --prefix=*foo* --enable-full-debug --enable-redirect-malloc
           --disable-threads- make- make install
         With a very recent collector on Linux, it may be safe to omit the --disable-threads.- Set environment variables as follows:
           - LD\_PRELOAD=*foo*/lib/libgc.so- GC\_FIND\_LEAK- You may also want to set GC\_PRINT\_STATS (to confirm that the collector
                 is running) and/or GC\_LOOP\_ON\_ABORT (to facilitate debugging from another
                 window if something goes wrong).
           Simply run a.out as you normally would. Note that if you run anything
           else (*e.g.* your editor) with those environment variables set,
           it will also be leak tested. This may or may not be useful and/or
           embarrassing. It can generate
           mountains of leak reports if the application wasn't designed to avoid leaks,
           *e.g.* because it's always short-lived.

This has not yet been thoroughly tested on large applications, but it's known
to do the right thing on at least some small ones.
