# Supplementary material for: Evaluation of de novo transcriptome assemblies from RNA-Seq data
Source: Genome Biol. 2014 Dec 21;15(12):553. doi: 10.1186/s13059-014-0553-5 (PMC4298084; doi:10.1186/s13059-014-0553-5)
Supplement: Additional file 3 — Version of the DETONATE source code used for the experiments in this paper. [file 13059_2014_553_MOESM3_ESM.zip › 13059_2014_553_MOESM3_ESM/detonate-1.8.1/ref-eval/boost/tools/build/v2/engine/boehm_gc/doc/overview.html]

A garbage collector for C and C++


|  |  |  |  |  |  |
| --- | --- | --- | --- | --- | --- |
| Interface Overview | Tutorial Slides | FAQ | Example | Download | License |

# A garbage collector for C and C++

- Platforms
- Scalable multiprocessor versions
- Some collector details
- Further reading
- Current users
- Local Links for this collector
- Local Background Links
- Contacts and Mailing List

[ This is an updated version of the page formerly at
http://reality.sgi.com/boehm/gc.html
and before that at
ftp://parcftp.xerox.com/pub/gc/gc.html.]

The Boehm-Demers-Weiser
conservative garbage collector can
be used as a garbage collecting
replacement for C malloc or C++ new.
It allows you to allocate memory basically as you normally would,
without explicitly deallocating memory that is no longer useful.
The collector automatically recycles memory when it determines
that it can no longer be otherwise accessed.
A simple example of such a use is given
here.

The collector is also used by a number of programming language
implementations that either use C as intermediate code, want
to facilitate easier interoperation with C libraries, or
just prefer the simple collector interface.
For a more detailed description of the interface, see
here.

Alternatively, the garbage collector may be used as
a leak detector
for C or C++ programs, though that is not its primary goal.

Typically several versions will be available.
Usually you should first try to use
gc\_source/gc.tar.gz,
which is normally an older, more stable version.

If that fails, try the latest explicitly numbered version
in 
gc\_source/.
Later versions may contain additional features, platform support,
or bug fixes, but are likely to be less well tested.
Note that versions containing the letters alpha are even less
well tested than others, especially on non-HP platforms.

A slightly older version of the garbage collector is now also
included as part of the
GNU compiler
distribution. The source
code for that version is available for browsing
here.

The arguments for and against conservative garbage collection
in C and C++ are briefly
discussed in
issues.html. The beginnings of
a frequently-asked-questions list are here.

The garbage collector code is copyrighted by
Hans-J. Boehm,
Alan J. Demers,
Xerox Corporation,
Silicon Graphics,
and
Hewlett-Packard Company.
It may be used and copied without payment of a fee under minimal restrictions.
See the README file in the distribution or the
license for more details.
**IT IS PROVIDED AS IS,
WITH ABSOLUTELY NO WARRANTY EXPRESSED OR IMPLIED. ANY USE IS AT YOUR OWN RISK**.

Empirically, this collector works with most unmodified C programs,
simply by replacing
malloc with GC\_malloc calls,
replacing realloc with GC\_realloc calls, and removing
free calls. Exceptions are discussed
in issues.html.

## Platforms

The collector is not completely portable, but the distribution
includes ports to most standard PC and UNIX/Linux platforms.
The collector should work on Linux, \*BSD, recent Windows versions,
MacOS X, HP/UX, Solaris,
Tru64, Irix and a few other operating systems.
Some ports are more polished than others.

Irix pthreads, Linux threads, Win32 threads, Solaris threads
(old style and pthreads),
HP/UX 11 pthreads, Tru64 pthreads, and MacOS X threads are supported
in recent versions.

### Separately distributed ports

For MacOS 9/Classic use, Patrick Beard's latest port is available from
http://homepage.mac.com/pcbeard/gc/.
(Unfortunately, that's now quite dated.
I'm not in a position to test under MacOS. Although I try to
incorporate changes, it is impossible for
me to update the project file.)

Precompiled versions of the collector for NetBSD are available
here
or
here.

Debian Linux includes prepackaged
versions of the collector.

## Scalable multiprocessor versions

Kenjiro Taura, Toshio Endo, and Akinori Yonezawa have made available
a parallel collector
based on this one. Their collector takes advantage of multiple processors
during a collection. Starting with collector version 6.0alpha1
we also do this, though with more modest processor scalability goals.
Our approach is discussed briefly in
scale.html.

## Some Collector Details

The collector uses a mark-sweep algorithm.
It provides incremental and generational
collection under operating systems which provide the right kind of
virtual memory support. (Currently this includes SunOS[45], IRIX,
OSF/1, Linux, and Windows, with varying restrictions.)
It allows *finalization* code
to be invoked when an object is collected.
It can take advantage of type information to locate pointers if such
information is provided, but it is usually used without such information.
ee the README and
gc.h files in the distribution for more details.

For an overview of the implementation, see here.

The garbage collector distribution includes a C string
(*cord*) package that provides
for fast concatenation and substring operations on long strings.
A simple curses- and win32-based editor that represents the entire file
as a cord is included as a
sample application.

Performance of the nonincremental collector is typically competitive
with malloc/free implementations. Both space and time overhead are
likely to be only slightly higher
for programs written for malloc/free
(see Detlefs, Dosser and Zorn's
Memory Allocation Costs in Large C and C++ Programs.)
For programs allocating primarily very small objects, the collector
may be faster; for programs allocating primarily large objects it will
be slower. If the collector is used in a multithreaded environment
and configured for thread-local allocation, it may in some cases
significantly outperform malloc/free allocation in time.

We also expect that in many cases any additional overhead
will be more than compensated for by decreased copying etc.
if programs are written
and tuned for garbage collection.

# Further Reading:

**The beginnings of a frequently asked questions list for this
collector are here**.

**The following provide information on garbage collection in general**:

Paul Wilson's garbage collection ftp archive and GC survey.

The Ravenbrook 
Memory Management Reference.

David Chase's
GC FAQ.

Richard Jones'
GC page and
his book.

**The following papers describe the collector algorithms we use
and the underlying design decisions at
a higher level.**

(Some of the lower level details can be found
here.)

The first one is not available
electronically due to copyright considerations. Most of the others are
subject to ACM copyright.

Boehm, H., "Dynamic Memory Allocation and Garbage Collection", *Computers in Physics
9*, 3, May/June 1995, pp. 297-303. This is directed at an otherwise sophisticated
audience unfamiliar with memory allocation issues. The algorithmic details differ
from those in the implementation. There is a related letter to the editor and a minor
correction in the next issue.

Boehm, H., and M. Weiser,
"Garbage Collection in an Uncooperative Environment",
*Software Practice & Experience*, September 1988, pp. 807-820.

Boehm, H., A. Demers, and S. Shenker, "Mostly Parallel Garbage Collection", Proceedings
of the ACM SIGPLAN '91 Conference on Programming Language Design and Implementation,
*SIGPLAN Notices 26*, 6 (June 1991), pp. 157-164.

Boehm, H., "Space Efficient Conservative Garbage Collection", Proceedings of the ACM
SIGPLAN '93 Conference on Programming Language Design and Implementation, *SIGPLAN
Notices 28*, 6 (June 1993), pp. 197-206.

Boehm, H., "Reducing Garbage Collector Cache Misses",
 *Proceedings of the 2000 International Symposium on Memory Management* .
Official version.

Technical report version. Describes the prefetch strategy
incorporated into the collector for some platforms. Explains why
the sweep phase of a "mark-sweep" collector should not really be
a distinct phase.

M. Serrano, H. Boehm,
"Understanding Memory Allocation of Scheme Programs",
*Proceedings of the Fifth ACM SIGPLAN International Conference on
Functional Programming*, 2000, Montreal, Canada, pp. 245-256.
Official version.

Earlier Technical Report version. Includes some discussion of the
collector debugging facilities for identifying causes of memory retention.

Boehm, H.,
"Fast Multiprocessor Memory Allocation and Garbage Collection",
HP Labs Technical Report HPL 2000-165. Discusses the parallel
collection algorithms, and presents some performance results.

Boehm, H., "Bounding Space Usage of Conservative Garbage Collectors",
*Proceeedings of the 2002 ACM SIGPLAN-SIGACT Symposium on Principles of
Programming Languages*, Jan. 2002, pp. 93-100.
Official version.

Technical report version.
Includes a discussion of a collector facility to much more reliably test for
the potential of unbounded heap growth.

**The following papers discuss language and compiler restrictions necessary to guaranteed
safety of conservative garbage collection.**

We thank John Levine and JCLT for allowing
us to make the second paper available electronically, and providing PostScript for the final
version.

Boehm, H., ``Simple
Garbage-Collector-Safety'', Proceedings
of the ACM SIGPLAN '96 Conference on Programming Language Design
and Implementation.

Boehm, H., and D. Chase, 
``A Proposal for Garbage-Collector-Safe C Compilation'',
*Journal of C Language Translation 4*, 2 (Decemeber 1992), pp. 126-141.

**Other related information:**

The Detlefs, Dosser and Zorn's Memory Allocation Costs in Large C and C++ Programs.
This is a performance comparison of the Boehm-Demers-Weiser collector to malloc/free,
using programs written for malloc/free.

Joel Bartlett's mostly copying conservative garbage collector for C++.

John Ellis and David Detlef's Safe Efficient Garbage Collection for C++ proposal.

Henry Baker's paper collection.

Slides for Hans Boehm's Allocation and GC Myths talk.

# Current users:

Known current users of some variant of this collector include:

The runtime system for GCJ,
the static GNU java compiler.

W3m, a text-based web browser.

Some versions of the Xerox DocuPrint printer software.

The Mozilla project, as leak
detector.

The Mono project,
an open source implementation of the .NET development framework.

The DotGNU Portable.NET
project, another open source .NET implementation.

The Irssi IRC client.

The Berkeley Titanium project.

The NAGWare f90 Fortran 90 compiler.

Elwood Corporation's 
Eclipse Common Lisp system, C library, and translator.

The Bigloo
Scheme
and Camloo ML
compilers
written by Manuel Serrano and others.

Brent Benson's libscheme.

The MzScheme scheme implementation.

The University of Washington Cecil Implementation.

The Berkeley Sather implementation.

The Berkeley Harmonia Project.

The Toba Java Virtual
Machine to C translator.

The Gwydion Dylan compiler.

The 
GNU Objective C runtime.

Macaulay 2, a system to support
research in algebraic geometry and commutative algebra.

The Vesta configuration management
system.

Visual Prolog 6.

Asymptote LaTeX-compatible
vector graphics language.

# More collector information at this site

A simple illustration of how to build and
use the collector..

Description of alternate interfaces to the
garbage collector.

Slides from an ISMM 2004 tutorial about the GC.

A FAQ (frequently asked questions) list.

How to use the garbage collector as a leak detector.

Some hints on debugging garbage collected
applications.

An overview of the implementation of the
garbage collector.

The data structure used for fast pointer lookups.

Scalability of the collector to multiprocessors.

Directory containing garbage collector source.

# More background information at this site

An attempt to establish a bound on space usage of
conservative garbage collectors.

Mark-sweep versus copying garbage collectors
and their complexity.

Pros and cons of conservative garbage collectors,
in comparison to other collectors.

Issues related to garbage collection vs.
manual memory management in C/C++.

An example of a case in which garbage collection
results in a much faster implementation as a result of reduced
synchronization.

Slide set discussing performance of nonmoving
garbage collectors.

Slide set discussing *Destructors, Finalizers, and Synchronization*
(POPL 2003).

Paper corresponding to above slide set.
(
Technical Report version.)

A Java/Scheme/C/C++ garbage collection benchmark.

Slides for talk on memory allocation myths.

Slides for OOPSLA 98 garbage collection talk.

Related papers.

# Contacts and Mailing List

We have recently set up two mailing list for collector announcements
and discussions:

- gc-announce@linux.hpl.hp.com
  is used for announcements of new versions. Postings are restricted.
  We expect this to always remain a very low volume list.
- gc@linux.hpl.hp.com is used for
  discussions, bug reports, and the like. Subscribers may post.
  On-topic posts by nonsubscribers will usually also be accepted, but
  it may take some time to review them.

To subscribe to these lists, send a mail message containing the
word "subscribe" to
gc-announce-request@linux.hpl.hp.com
or to
gc-request@linux.hpl.hp.com.
(Please ignore the instructions about web-based subscription.
The listed web site is behind the HP firewall.)

The archives for these lists appear
here.
The gc list archive may also be read at
gmane.org.

Some prior discussion of the collector has taken place on the gcc
java mailing list, whose archives appear
here, and also on
gclist@iecc.com.

Comments and bug reports may also be sent to
(Hans.Boehm@hp.com) or
(boehm@acm.org), but the gc
mailing list is usually preferred.
