# Supplementary material for: Evaluation of de novo transcriptome assemblies from RNA-Seq data
Source: Genome Biol. 2014 Dec 21;15(12):553. doi: 10.1186/s13059-014-0553-5 (PMC4298084; doi:10.1186/s13059-014-0553-5)
Supplement: Additional file 3 — Version of the DETONATE source code used for the experiments in this paper. [file 13059_2014_553_MOESM3_ESM.zip › 13059_2014_553_MOESM3_ESM/detonate-1.8.1/ref-eval/boost/tools/build/v2/engine/boehm_gc/doc/porting.html]

Conservative GC Porting Directions


# Conservative GC Porting Directions

The collector is designed to be relatively easy to port, but is not
portable code per se. The collector inherently has to perform operations,
such as scanning the stack(s), that are not possible in portable C code.

All of the following assumes that the collector is being ported to a
byte-addressable 32- or 64-bit machine. Currently all successful ports
to 64-bit machines involve LP64 targets. The code base includes some
provisions for P64 targets (notably win64), but that has not been tested.
You are hereby discouraged from attempting a port to non-byte-addressable,
or 8-bit, or 16-bit machines.

The difficulty of porting the collector varies greatly depending on the needed
functionality. In the simplest case, only some small additions are needed
for the include/private/gcconfig.h file. This is described in the
following section. Later sections discuss some of the optional features,
which typically involve more porting effort.

Note that the collector makes heavy use of ifdefs. Unlike
some other software projects, we have concluded repeatedly that this is preferable
to system dependent files, with code duplicated between the files.
However, to keep this manageable, we do strongly believe in indenting
ifdefs correctly (for historical reasons usually without the leading
sharp sign). (Separate source files are of course fine if they don't result in
code duplication.)

## Adding Platforms to gcconfig.h

If neither thread support, nor tracing of dynamic library data is required,
these are often the only changes you will need to make.

The gcconfig.h file consists of three sections:

1. A section that defines GC-internal macros
   that identify the architecture (e.g. IA64 or I386)
   and operating system (e.g. LINUX or MSWIN32).
   This is usually done by testing predefined macros. By defining
   our own macros instead of using the predefined ones directly, we can
   impose a bit more consistency, and somewhat isolate ourselves from
   compiler differences.

   It is relatively straightforward to add a new entry here. But please try
   to be consistent with the existing code. In particular, 64-bit variants
   of 32-bit architectures general are *not* treated as a new architecture.
   Instead we explicitly test for 64-bit-ness in the few places in which it
   matters. (The notable exception here is I386 and X86\_64.
   This is partially historical, and partially justified by the fact that there
   are arguably more substantial architecture and ABI differences here than
   for RISC variants.)

   on GNU-based systems, cpp -dM empty\_source\_file.c seems to generate
   a set of predefined macros. On some other systems, the "verbose"
   compiler option may do so, or the manual page may list them.- A section that defines a small number of platform-specific macros, which are
     then used directly by the collector. For simple ports, this is where most of
     the effort is required. We describe the macros below.

     This section contains a subsection for each architecture (enclosed in a
     suitable ifdef. Each subsection usually contains some
     architecture-dependent defines, followed by several sets of OS-dependent
     defines, again enclosed in ifdefs.- A section that fills in defaults for some macros left undefined in the preceding
       section, and defines some other macros that rarely need adjustment for
       new platforms. You will typically not have to touch these.
       If you are porting to an OS that
       was previously completely unsupported, it is likely that you will
       need to add another clause to the definition of GET\_MEM.

The following macros must be defined correctly for each architecture and operating
system:

MACH\_TYPE: Defined to a string that represents the machine architecture. Usually just the macro name used to identify the architecture, but enclosed in quotes. OS\_TYPE: Defined to a string that represents the operating system name. Usually just the macro name used to identify the operating system, but enclosed in quotes. CPP\_WORDSZ: The word size in bits as a constant suitable for preprocessor tests, i.e. without casts or sizeof expressions. Currently always defined as either 64 or 32. For platforms supporting both 32- and 64-bit ABIs, this should be conditionally defined depending on the current ABI. There is a default of 32. ALIGNMENT: Defined to be the largest N, such that all pointer are guaranteed to be aligned on N-byte boundaries. defining it to be 1 will always work, but perform poorly. For all modern 32-bit platforms, this is 4. For all modern 64-bit platforms, this is 8. Whether or not X86 qualifies as a modern architecture here is compiler- and OS-dependent. DATASTART: The beginning of the main data segment. The collector will trace all memory between DATASTART and DATAEND for root pointers. On some platforms,this can be defined to a constant address, though experience has shown that to be risky. Ideally the linker will define a symbol (e.g. \_data whose address is the beginning of the data segment. Sometimes the value can be computed using the GC\_SysVGetDataStart function. Not used if either the next macro is defined, or if dynamic loading is supported, and the dynamic loading support defines a function GC\_register\_main\_static\_data() which returns false. SEARCH\_FOR\_DATA\_START: If this is defined DATASTART will be defined to a dynamically computed value which is obtained by starting with the address of \_end and walking backwards until non-addressable memory is found. This often works on Posix-like platforms. It makes it harder to debug client programs, since startup involves generating and catching a segmentation fault, which tends to confuse users. DATAEND: Set to the end of the main data segment. Defaults to end, where that is declared as an array. This works in some cases, since the linker introduces a suitable symbol. DATASTART2, DATAEND2: Some platforms have two discontiguous main data segments, e.g. for initialized and uninitialized data. If so, these two macros should be defined to the limits of the second main data segment. STACK\_GROWS\_UP: Should be defined if the stack (or thread stacks) grow towards higher addresses. (This appears to be true only on PA-RISC. If your architecture has more than one stack per thread, and is not already supported, you will need to do more work. Grep for "IA64" in the source for an example.) STACKBOTTOM: Defined to be the cool end of the stack, which is usually the highest address in the stack. It must bound the region of the stack that contains pointers into the GC heap. With thread support, this must be the cold end of the main stack, which typically cannot be found in the same way as the other thread stacks. If this is not defined and none of the following three macros is defined, client code must explicitly set GC\_stackbottom to an appropriate value before calling GC\_INIT() or any other GC\_ routine. LINUX\_STACKBOTTOM: May be defined instead of STACKBOTTOM. If defined, then the cold end of the stack will be determined Currently we usually read it from /proc. HEURISTIC1: May be defined instead of STACKBOTTOM. STACK\_GRAN should generally also be undefined and defined. The cold end of the stack is determined by taking an address inside GC\_init's frame, and rounding it up to the next multiple of STACK\_GRAN. This works well if the stack base is always aligned to a large power of two. (STACK\_GRAN is predefined to 0x1000000, which is rarely optimal.) HEURISTIC2: May be defined instead of STACKBOTTOM. The cold end of the stack is determined by taking an address inside GC\_init's frame, incrementing it repeatedly in small steps (decrement if STACK\_GROWS\_UP), and reading the value at each location. We remember the value when the first Segmentation violation or Bus error is signalled, round that to the nearest plausible page boundary, and use that as the stack base. DYNAMIC\_LOADING: Should be defined if dyn\_load.c has been updated for this platform and tracing of dynamic library roots is supported. MPROTECT\_VDB, PROC\_VDB: May be defined if the corresponding "virtual dirty bit" implementation in os\_dep.c is usable on this platform. This allows incremental/generational garbage collection. MPROTECT\_VDB identifies modified pages by write protecting the heap and catching faults. PROC\_VDB uses the /proc primitives to read dirty bits. PREFETCH, PREFETCH\_FOR\_WRITE: The collector uses PREFETCH(*x*) to preload the cache with \**x*. This defaults to a no-op. CLEAR\_DOUBLE: If CLEAR\_DOUBLE is defined, then CLEAR\_DOUBLE(x) is used as a fast way to clear the two words at GC\_malloc-aligned address x. By default, word stores of 0 are used instead. HEAP\_START: HEAP\_START may be defined as the initial address hint for mmap-based allocation. ALIGN\_DOUBLE: Should be defined if the architecture requires double-word alignment of GC\_malloced memory, e.g. 8-byte alignment with a 32-bit ABI. Most modern machines are likely to require this. This is no longer needed for GC7 and later.

## Additional requirements for a basic port

In some cases, you may have to add additional platform-specific code
to other files. A likely candidate is the implementation of
GC\_with\_callee\_saves\_pushed in mach\_dep.c.
This ensure that register contents that the collector must trace
from are copied to the stack. Typically this can be done portably,
but on some platforms it may require assembly code, or just
tweaking of conditional compilation tests.

For GC7, if your platform supports getcontext(), then definining
the macro UNIX\_LIKE for your OS in gcconfig.h
(if it isn't defined there already) is likely to solve the problem.
otherwise, if you are using gcc, \_builtin\_unwind\_init()
will be used, and should work fine. If that is not applicable either,
the implementation will try to use setjmp(). This will work if your
setjmp implementation saves all possibly pointer-valued registers
into the buffer, as opposed to trying to unwind the stack at
longjmp time. The setjmp\_test test tries to determine this,
but often doesn't get it right.

In GC6.x versions of the collector, tracing of registers
was more commonly handled
with assembly code. In GC7, this is generally to be avoided.

Most commonly os\_dep.c will not require attention, but see below.

## Thread support

Supporting threads requires that the collector be able to find and suspend
all threads potentially accessing the garbage-collected heap, and locate
any state associated with each thread that must be traced.

The functionality needed for thread support is generally implemented
in one or more files specific to the particular thread interface.
For example, somewhat portable pthread support is implemented
in pthread\_support.c and pthread\_stop\_world.c.
The essential functionality consists of

GC\_stop\_world(): Stops all threads which may access the garbage collected heap, other than the caller. GC\_start\_world(): Restart other threads. GC\_push\_all\_stacks(): Push the contents of all thread stacks (or at least of pointer-containing regions in the thread stacks) onto the mark stack.

These very often require that the garbage collector maintain its
own data structures to track active threads.

In addition, LOCK and UNLOCK must be implemented
in gc\_locks.h

The easiest case is probably a new pthreads platform
on which threads can be stopped
with signals. In this case, the changes involve:

1. Introducing a suitable GC\_*X*\_THREADS macro, which should
   be automatically defined by gc\_config\_macros.h in the right cases.
   It should also result in a definition of GC\_PTHREADS, as for the
   existing cases.- For GC7+, ensuring that the atomic\_ops package at least
     minimally supports the platform.
     If incremental GC is needed, or if pthread locks don't
     perform adequately as the allocation lock, you will probably need to
     ensure that a sufficient atomic\_ops port
     exists for the platform to provided an atomic test and set
     operation. (Current GC7 versions require moreatomic\_ops
     asupport than necessary. This is a bug.) For earlier versions define
     GC\_test\_and\_set in gc\_locks.h.- Making any needed adjustments to pthread\_stop\_world.c and
       pthread\_support.c. Ideally none should be needed. In fact,
       not all of this is as well standardized as one would like, and outright
       bugs requiring workarounds are common.

Non-preemptive threads packages will probably require further work. Similarly
thread-local allocation and parallel marking requires further work
in pthread\_support.c, and may require better atomic\_ops
support.

## Dynamic library support

So long as DATASTART and DATAEND are defined correctly,
the collector will trace memory reachable from file scope or static
variables defined as part of the main executable. This is sufficient
if either the program is statically linked, or if pointers to the
garbage-collected heap are never stored in non-stack variables
defined in dynamic libraries.

If dynamic library data sections must also be traced, then

- DYNAMIC\_LOADING must be defined in the appropriate section
  of gcconfig.h.- An appropriate versions of the functions
    GC\_register\_dynamic\_libraries() should be defined in
    dyn\_load.c. This function should invoke
    GC\_cond\_add\_roots(*region\_start, region\_end*, TRUE)
    on each dynamic library data section.

Implementations that scan for writable data segments are error prone, particularly
in the presence of threads. They frequently result in race conditions
when threads exit and stacks disappear. They may also accidentally trace
large regions of graphics memory, or mapped files. On at least
one occasion they have been known to try to trace device memory that
could not safely be read in the manner the GC wanted to read it.

It is usually safer to walk the dynamic linker data structure, especially
if the linker exports an interface to do so. But beware of poorly documented
locking behavior in this case.

## Incremental GC support

For incremental and generational collection to work, os\_dep.c
must contain a suitable "virtual dirty bit" implementation, which
allows the collector to track which heap pages (assumed to be
a multiple of the collectors block size) have been written during
a certain time interval. The collector provides several
implementations, which might be adapted. The default
(DEFAULT\_VDB) is a placeholder which treats all pages
as having been written. This ensures correctness, but renders
incremental and generational collection essentially useless.

## Stack traces for debug support

If stack traces in objects are need for debug support,
GC\_dave\_callers and GC\_print\_callers must be
implemented.

## Disclaimer

This is an initial pass at porting guidelines. Some things
have no doubt been overlooked.
