# Supplementary material for: Evaluation of de novo transcriptome assemblies from RNA-Seq data
Source: Genome Biol. 2014 Dec 21;15(12):553. doi: 10.1186/s13059-014-0553-5 (PMC4298084; doi:10.1186/s13059-014-0553-5)
Supplement: Additional file 3 — Version of the DETONATE source code used for the experiments in this paper. [file 13059_2014_553_MOESM3_ESM.zip › 13059_2014_553_MOESM3_ESM/detonate-1.8.1/ref-eval/boost/tools/build/v2/engine/boehm_gc/doc/scale.html]

Garbage collector scalability


# Garbage collector scalability

In its default configuration, the Boehm-Demers-Weiser garbage collector
is not thread-safe. It can be made thread-safe for a number of environments
by building the collector with the appropriate
-D*XXX*-THREADS compilation
flag. This has primarily two effects:

1. It causes the garbage collector to stop all other threads when
   it needs to see a consistent memory state.- It causes the collector to acquire a lock around essentially all
     allocation and garbage collection activity.

Since a single lock is used for all allocation-related activity, only one
thread can be allocating or collecting at one point. This inherently
limits performance of multi-threaded applications on multiprocessors.

On most platforms, the allocator/collector lock is implemented as a
spin lock with exponential back-off. Longer wait times are implemented
by yielding and/or sleeping. If a collection is in progress, the pure
spinning stage is skipped. This has the advantage that uncontested and
thus most uniprocessor lock acquisitions are very cheap. It has the
disadvantage that the application may sleep for small periods of time
even when there is work to be done. And threads may be unnecessarily
woken up for short periods. Nonetheless, this scheme empirically
outperforms native queue-based mutual exclusion implementations in most
cases, sometimes drastically so.

## Options for enhanced scalability

Version 6.0 of the collector adds two facilities to enhance collector
scalability on multiprocessors. As of 6.0alpha1, these are supported
only under Linux on X86 and IA64 processors, though ports to other
otherwise supported Pthreads platforms should be straightforward.
They are intended to be used together.

- Building the collector with -DPARALLEL\_MARK allows the collector to
  run the mark phase in parallel in multiple threads, and thus on multiple
  processors. The mark phase typically consumes the large majority of the
  collection time. Thus this largely parallelizes the garbage collector
  itself, though not the allocation process. Currently the marking is
  performed by the thread that triggered the collection, together with
  *N*-1 dedicated
  threads, where *N* is the number of processors detected by the collector.
  The dedicated threads are created once at initialization time.

  A second effect of this flag is to switch to a more concurrent
  implementation of GC\_malloc\_many, so that free lists can be
  built, and memory can be cleared, by more than one thread concurrently.- Building the collector with -DTHREAD\_LOCAL\_ALLOC adds support for thread
    local allocation. It does not, by itself, cause thread local allocation
    to be used. It simply allows the use of the interface in
    gc\_local\_alloc.h.

    Memory returned from thread-local allocators is completely interchangeable
    with that returned by the standard allocators. It may be used by other
    threads. The only difference is that, if the thread allocates enough
    memory of a certain kind, it will build a thread-local free list for
    objects of that kind, and allocate from that. This greatly reduces
    locking. The thread-local free lists are refilled using
    GC\_malloc\_many.

    An important side effect of this flag is to replace the default
    spin-then-sleep lock to be replace by a spin-then-queue based implementation.
    This *reduces performance* for the standard allocation functions,
    though it usually improves performance when thread-local allocation is
    used heavily, and thus the number of short-duration lock acquisitions
    is greatly reduced.

The easiest way to switch an application to thread-local allocation is to

1. Define the macro GC\_REDIRECT\_TO\_LOCAL,
   and then include the gc.h
   header in each client source file.- Invoke GC\_thr\_init() before any allocation.- Allocate using GC\_MALLOC, GC\_MALLOC\_ATOMIC,
       and/or GC\_GCJ\_MALLOC.

## The Parallel Marking Algorithm

We use an algorithm similar to
that developed by
Endo, Taura, and Yonezawa at the University of Tokyo.
However, the data structures and implementation are different,
and represent a smaller change to the original collector source,
probably at the expense of extreme scalability. Some of
the refinements they suggest, *e.g.* splitting large
objects, were also incorporated into out approach.

The global mark stack is transformed into a global work queue.
Unlike the usual case, it never shrinks during a mark phase.
The mark threads remove objects from the queue by copying them to a
local mark stack and changing the global descriptor to zero, indicating
that there is no more work to be done for this entry.
This removal
is done with no synchronization. Thus it is possible for more than
one worker to remove the same entry, resulting in some work duplication.

The global work queue grows only if a marker thread decides to
return some of its local mark stack to the global one. This
is done if the global queue appears to be running low, or if
the local stack is in danger of overflowing. It does require
synchronization, but should be relatively rare.

The sequential marking code is reused to process local mark stacks.
Hence the amount of additional code required for parallel marking
is minimal.

It should be possible to use generational collection in the presence of the
parallel collector, by calling GC\_enable\_incremental().
This does not result in fully incremental collection, since parallel mark
phases cannot currently be interrupted, and doing so may be too
expensive.

Gcj-style mark descriptors do not currently mix with the combination
of local allocation and incremental collection. They should work correctly
with one or the other, but not both.

The number of marker threads is set on startup to the number of
available processors (or to the value of the GC\_NPROCS
environment variable). If only a single processor is detected,
parallel marking is disabled.

Note that setting GC\_NPROCS to 1 also causes some lock acquisitions inside
the collector to immediately yield the processor instead of busy waiting
first. In the case of a multiprocessor and a client with multiple
simultaneously runnable threads, this may have disastrous performance
consequences (e.g. a factor of 10 slowdown).

## Performance

We conducted some simple experiments with a version of
our GC benchmark that was slightly modified to
run multiple concurrent client threads in the same address space.
Each client thread does the same work as the original benchmark, but they share
a heap.
This benchmark involves very little work outside of memory allocation.
This was run with GC 6.0alpha3 on a dual processor Pentium III/500 machine
under Linux 2.2.12.

Running with a thread-unsafe collector, the benchmark ran in 9
seconds. With the simple thread-safe collector,
built with -DLINUX\_THREADS, the execution time
increased to 10.3 seconds, or 23.5 elapsed seconds with two clients.
(The times for the malloc/ifree version
with glibc malloc
are 10.51 (standard library, pthreads not linked),
20.90 (one thread, pthreads linked),
and 24.55 seconds respectively. The benchmark favors a
garbage collector, since most objects are small.)

The following table gives execution times for the collector built
with parallel marking and thread-local allocation support
(-DGC\_LINUX\_THREADS -DPARALLEL\_MARK -DTHREAD\_LOCAL\_ALLOC). We tested
the client using either one or two marker threads, and running
one or two client threads. Note that the client uses thread local
allocation exclusively. With -DTHREAD\_LOCAL\_ALLOC the collector
switches to a locking strategy that is better tuned to less frequent
lock acquisition. The standard allocation primitives thus peform
slightly worse than without -DTHREAD\_LOCAL\_ALLOC, and should be
avoided in time-critical code.

(The results using pthread\_mutex\_lock
directly for allocation locking would have been worse still, at
least for older versions of linuxthreads.
With THREAD\_LOCAL\_ALLOC, we first repeatedly try to acquire the
lock with pthread\_mutex\_try\_lock(), busy\_waiting between attempts.
After a fixed number of attempts, we use pthread\_mutex\_lock().)

These measurements do not use incremental collection, nor was prefetching
enabled in the marker. We used the C version of the benchmark.
All measurements are in elapsed seconds on an unloaded machine.

| Number of threads | 1 marker thread (secs.) | 2 marker threads (secs.) |
| --- | --- | --- |
| 1 client | 10.45 | 7.85 || 2 clients | 19.95 | 12.3 |

The execution time for the single threaded case is slightly worse than with
simple locking. However, even the single-threaded benchmark runs faster than
even the thread-unsafe version if a second processor is available.
The execution time for two clients with thread local allocation time is
only 1.4 times the sequential execution time for a single thread in a
thread-unsafe environment, even though it involves twice the client work.
That represents close to a
factor of 2 improvement over the 2 client case with the old collector.
The old collector clearly
still suffered from some contention overhead, in spite of the fact that the
locking scheme had been fairly well tuned.

Full linear speedup (i.e. the same execution time for 1 client on one
processor as 2 clients on 2 processors)
is probably not achievable on this kind of
hardware even with such a small number of processors,
since the memory system is
a major constraint for the garbage collector,
the processors usually share a single memory bus, and thus
the aggregate memory bandwidth does not increase in
proportion to the number of processors.

These results are likely to be very sensitive to both hardware and OS
issues. Preliminary experiments with an older Pentium Pro machine running
an older kernel were far less encouraging.
