# Supplementary material for: Evaluation of de novo transcriptome assemblies from RNA-Seq data
Source: Genome Biol. 2014 Dec 21;15(12):553. doi: 10.1186/s13059-014-0553-5 (PMC4298084; doi:10.1186/s13059-014-0553-5)
Supplement: Additional file 3 — Version of the DETONATE source code used for the experiments in this paper. [file 13059_2014_553_MOESM3_ESM.zip › 13059_2014_553_MOESM3_ESM/detonate-1.8.1/ref-eval/boost/tools/build/v2/engine/boehm_gc/doc/simple_example.html]

Using the Garbage Collector: A simple example


# Using the Garbage Collector: A simple example

The following consists of step-by-step instructions for building and
using the collector. We'll assume a Linux/gcc platform and
a single-threaded application. The green
text contains information about other platforms or scenarios.
It can be skipped, especially on first reading.

## Building the collector

If you haven't already so, unpack the collector and enter
the newly created directory with

```
tar xvfz gc.tar.gz
cd gc
```

You can configure, build, and install the collector in a private
directory, say /home/xyz/gc, with the following commands:

```
./configure --prefix=/home/xyz/gc --disable-threads
make
make check
make install
```

Here the "make check" command is optional, but highly recommended.
It runs a basic correctness test which usually takes well under a minute.

### Other platforms

On non-Unix, non-Linux platforms, the collector is usually built by copying
the appropriate makefile (see the platform-specific README in doc/README.xxx
in the distribution) to the file "Makefile" (overwriting the copy of
Makefile.direct that was originally there), and then typing "make"
(or "nmake" or ...). This builds the library in the source tree. You may
want to move it and the files in the include directory to a more convenient
place.

If you use a makefile that does not require running a configure script,
you should first look at the makefile, and adjust any options that are
documented there.

If your platform provides a "make" utility, that is generally preferred
to platform- and compiler- dependent "project" files. (At least that is the
strong preference of the would-be maintainer of those project files.)

### Threads

If you need thread support, configure the collector with

```
--enable-threads=posix --enable-thread-local-alloc --enable-parallel-mark
```

instead of
--disable-threads
If your target is a real old-fashioned uniprocessor (no "hyperthreading",
etc.) you will want to omit --enable-parallel-mark.

### C++

You will need to include the C++ support, which unfortunately tends to
be among the least portable parts of the collector, since it seems
to rely on some corner cases of the language. On Linux, it
suffices to add --enable-cplusplus to the configure options.

## Writing the program

You will need a

```
#include "gc.h"
```

at the beginning of every file that allocates memory through the
garbage collector. Call GC\_MALLOC wherever you would
have call malloc. This initializes memory to zero like
calloc; there is no need to explicitly clear the
result.

If you know that an object will not contain pointers to the
garbage-collected heap, and you don't need it to be initialized,
call GC\_MALLOC\_ATOMIC instead.

A function GC\_FREE is provided but need not be called.
For very small objects, your program will probably perform better if
you do not call it, and let the collector do its job.

A GC\_REALLOC function behaves like the C library realloc.
It allocates uninitialized pointer-free memory if the original
object was allocated that way.

The following program loop.c is a trivial example:

```
#include "gc.h"
#include <assert.h>
#include <stdio.h>

int main()
{
  int i;

  GC_INIT();	/* Optional on Linux/X86; see below.  */
  for (i = 0; i < 10000000; ++i)
   {
     int **p = (int **) GC_MALLOC(sizeof(int *));
     int *q = (int *) GC_MALLOC_ATOMIC(sizeof(int));
     assert(*p == 0);
     *p = (int *) GC_REALLOC(q, 2 * sizeof(int));
     if (i % 100000 == 0)
       printf("Heap size = %d\n", GC_get_heap_size());
   }
  return 0;
}
```

### Interaction with the system malloc

It is usually best not to mix garbage-collected allocation with the system
malloc-free. If you do, you need to be careful not to store
pointers to the garbage-collected heap in memory allocated with the system
malloc.

### Other Platforms

On some other platforms it is necessary to call GC\_INIT() from the main program,
which is presumed to be part of the main executable, not a dynamic library.
This can never hurt, and is thus generally good practice.

### Threads

For a multithreaded program some more rules apply:

- Files that either allocate through the GC *or make thread-related calls*
  should first define the macro GC\_THREADS, and then
  include "gc.h". On some platforms this will redefine some
  threads primitives, e.g. to let the collector keep track of thread creation.- To take advantage of fast thread-local allocation, use the following instead
    of including gc.h:

    ```
    #define GC_REDIRECT_TO_LOCAL
    #include "gc_local_alloc.h"
    ```

    This will cause GC\_MALLOC and GC\_MALLOC\_ATOMIC to keep per-thread allocation
    caches, and greatly reduce the number of lock acquisitions during allocation.

### C++

In the case of C++, you need to be especially careful not to store pointers
to the garbage-collected heap in areas that are not traced by the collector.
The collector includes some alternate interfaces
to make that easier.

### Debugging

Additional debug checks can be performed by defining GC\_DEBUG before
including gc.h. Additional options are available if the collector
is also built with --enable-full\_debug and all allocations are
performed with GC\_DEBUG defined.

### What if I can't rewrite/recompile my program?

You may be able to build the collector with --enable-redirect-malloc
and set the LD\_PRELOAD environment variable to point to the resulting
library, thus replacing the standard malloc with its garbage-collected
counterpart. This is rather platform dependent. See the
leak detection documentation for some more details.

## Compiling and linking

The above application loop.c test program can be compiled and linked
with

```
cc -I/home/xyz/gc/include loop.c /home/xyz/gc/lib/libgc.a -o loop
```

The -I option directs the compiler to the right include
directory. In this case, we list the static library
directly on the compile line; the dynamic library could have been
used instead, provided we arranged for the dynamic loader to find
it, e.g. by setting LD\_LIBRARY\_PATH.

### Threads

On pthread platforms, you will of course also have to link with
-lpthread,
and compile with any thread-safety options required by your compiler.
On some platforms, you may also need to link with -ldl
or -lrt.
Looking at threadlibs.c in the GC build directory
should give you the appropriate
list if a plain -lpthread doesn't work.

## Running the executable

The executable can of course be run normally, e.g. by typing

```
./loop
```

The operation of the collector is affected by a number of environment variables.
For example, setting GC\_PRINT\_STATS produces some
GC statistics on stdout.
See README.environment in the distribution for details.
