# Supplementary material for: Evaluation of de novo transcriptome assemblies from RNA-Seq data
Source: Genome Biol. 2014 Dec 21;15(12):553. doi: 10.1186/s13059-014-0553-5 (PMC4298084; doi:10.1186/s13059-014-0553-5)
Supplement: Additional file 3 — Version of the DETONATE source code used for the experiments in this paper. [file 13059_2014_553_MOESM3_ESM.zip › 13059_2014_553_MOESM3_ESM/detonate-1.8.1/ref-eval/boost/tools/build/v2/engine/boehm_gc/doc/tree.html]

 Two-Level Tree Structure for Fast Pointer Lookup
 Hans-J. Boehm, Silicon Graphics (now at HP)


# Two-Level Tree Structure for Fast Pointer Lookup

The conservative garbage collector described
here
uses a 2-level tree
data structure to aid in fast pointer identification.
This data structure is described in a bit more detail here, since

1. Variations of the data structure are more generally useful.- It appears to be hard to understand by reading the code.- Some other collectors appear to use inferior data structures to
       solve the same problem.- It is central to fast collector operation.

A candidate pointer is divided into three sections, the *high*,
*middle*, and *low* bits. The exact division between these
three groups of bits is dependent on the detailed collector configuration.

The high and middle bits are used to look up an entry in the table described
here. The resulting table entry consists of either a block descriptor
(struct hblkhdr \* or hdr \*)
identifying the layout of objects in the block, or an indication that this
address range corresponds to the middle of a large block, together with a
hint for locating the actual block descriptor. Such a hint consist
of a displacement that can be subtracted from the middle bits of the candidate
pointer without leaving the object.

In either case, the block descriptor (struct hblkhdr)
refers to a table of object starting addresses (the hb\_map field).
The starting address table is indexed by the low bits if the candidate pointer.
The resulting entry contains a displacement to the beginning of the object,
or an indication that this cannot be a valid object pointer.
(If all interior pointer are recognized, pointers into large objects
are handled specially, as appropriate.)

## The Tree

The rest of this discussion focuses on the two level data structure
used to map the high and middle bits to the block descriptor.

The high bits are used as an index into the GC\_top\_index (really
GC\_arrays.\_top\_index) array. Each entry points to a
bottom\_index data structure. This structure in turn consists
mostly of an array index indexed by the middle bits of
the candidate pointer. The index array contains the actual
hdr pointers.

Thus a pointer lookup consists primarily of a handful of memory references,
and can be quite fast:

1. The appropriate bottom\_index pointer is looked up in
   GC\_top\_index, based on the high bits of the candidate pointer.- The appropriate hdr pointer is looked up in the
     bottom\_index structure, based on the middle bits.- The block layout map pointer is retrieved from the hdr
       structure. (This memory reference is necessary since we try to share
       block layout maps.)- The displacement to the beginning of the object is retrieved from the
         above map.

In order to conserve space, not all GC\_top\_index entries in fact
point to distinct bottom\_index structures. If no address with
the corresponding high bits is part of the heap, then the entry points
to GC\_all\_nils, a single bottom\_index structure consisting
only of NULL hdr pointers.

Bottom\_index structures contain slightly more information than
just hdr pointers. The asc\_link field is used to link
all bottom\_index structures in ascending order for fast traversal.
This list is pointed to be GC\_all\_bottom\_indices.
It is maintained with the aid of key field that contains the
high bits corresponding to the bottom\_index.

## 64 bit addresses

In the case of 64 bit addresses, this picture is complicated slightly
by the fact that one of the index structures would have to be huge to
cover the entire address space with a two level tree. We deal with this
by turning GC\_top\_index into a chained hash table, instead of
a simple array. This adds a hash\_link field to the
bottom\_index structure.

The "hash function" consists of dropping the high bits. This is cheap to
compute, and guarantees that there will be no collisions if the heap
is contiguous and not excessively large.

## A picture

The following is an ASCII diagram of the data structure.
This was contributed by Dave Barrett several years ago.

```
		Data Structure used by GC_base in gc3.7:
			      21-Apr-94
			 
			


    63                  LOG_TOP_SZ[11]  LOG_BOTTOM_SZ[10]   LOG_HBLKSIZE[13]
   +------------------+----------------+------------------+------------------+
 p:|                  |   TL_HASH(hi)  |                  |   HBLKDISPL(p)   |
   +------------------+----------------+------------------+------------------+
    \-----------------------HBLKPTR(p)-------------------/
    \------------hi-------------------/ 
                      \______ ________/ \________ _______/ \________ _______/
                             V                   V                  V
                             |                   |                  |
           GC_top_index[]    |                   |                  | 
 ---      +--------------+   |                   |                  |  
  ^       |              |   |                   |                  |   
  |       |              |   |                   |                  |   
 TOP      +--------------+<--+                   |                  |      
 _SZ   +-<|      []      | *                     |                  |     
(items)|  +--------------+  if 0 < bi< HBLKSIZE  |                  |    
  |    |  |              | then large object     |                  |    
  |    |  |              | starts at the bi'th   |                  |    
  v    |  |              | HBLK before p.        |             i    |    
 ---   |  +--------------+                       |          (word-  |    
       v                                         |         aligned) |    
   bi= |GET_BI(p){->hash_link}->key==hi          |                  |   
       v                                         |                  |    
       |   (bottom_index)  \ scratch_alloc'd     |                  |    
       |   ( struct  bi )  / by get_index()      |                  |    
 ---   +->+--------------+                       |                  |    
  ^       |              |                       |                  |
  ^       |              |                       |                  |
 BOTTOM   |              |   ha=GET_HDR_ADDR(p)  |                  |
_SZ(items)+--------------+<----------------------+          +-------+
  |   +--<|   index[]    |                                  |         
  |   |   +--------------+                      GC_obj_map: v              
  |   |   |              |              from      / +-+-+-----+-+-+-+-+  --- 
  v   |   |              |              GC_add   < 0| | |     | | | | |   ^  
 ---  |   +--------------+             _map_entry \ +-+-+-----+-+-+-+-+   |  
      |   |   asc_link   |                          +-+-+-----+-+-+-+-+ MAXOBJSZ
      |   +--------------+                      +-->| | |  j  | | | | |  +1   
      |   |     key      |                      |   +-+-+-----+-+-+-+-+   |  
      |   +--------------+                      |   +-+-+-----+-+-+-+-+   | 
      |   |  hash_link   |                      |   | | |     | | | | |   v 
      |   +--------------+                      |   +-+-+-----+-+-+-+-+  ---
      |                                         |   |<--MAX_OFFSET--->|   
      |                                         |         (bytes)
HDR(p)| GC_find_header(p)                       |   |<--MAP_ENTRIES-->| 
      |                           \ from        |    =HBLKSIZE/WORDSZ   
      |    (hdr) (struct hblkhdr) / alloc_hdr() |    (1024 on Alpha)
      +-->+----------------------+              |    (8/16 bits each)
GET_HDR(p)| word   hb_sz (words) |              |          
          +----------------------+              |     
          | struct hblk *hb_next |              |
          +----------------------+              |       
          |mark_proc hb_mark_proc|              |
          +----------------------+              |
          | char * hb_map        |>-------------+
          +----------------------+           
          | ushort hb_obj_kind   |           
          +----------------------+           
          |   hb_last_reclaimed  |           
 ---      +----------------------+                
  ^       |                      |
 MARK_BITS|       hb_marks[]     | *if hdr is free, hb_sz + DISCARD_WORDS
_SZ(words)|                      |  is the size of a heap chunk (struct hblk)
  v       |                      |  of at least MININCR*HBLKSIZE bytes (below),
 ---      +----------------------+  otherwise, size of each object in chunk.

Dynamic data structures above are interleaved throughout the heap in blocks of 
size MININCR * HBLKSIZE bytes as done by gc_scratch_alloc which cannot be
freed; free lists are used (e.g. alloc_hdr).  HBLK's below are collected.

	      (struct hblk)      
 ---      +----------------------+ < HBLKSIZE ---         ---          DISCARD_
  ^       |garbage[DISCARD_WORDS]|   aligned   ^           ^ HDR_BYTES WORDS
  |       |                      |             |           v (bytes)   (words)
  |       +-----hb_body----------+ < WORDSZ    |          ---   ---   
  |       |                      |   aligned   |           ^     ^
  |       |      Object 0        |             |           hb_sz |
  |       |                      |           i |(word-    (words)|
  |       |                      |      (bytes)|aligned)   v     |
  |       + - - - - - - - - - - -+ ---         |          ---    |
  |       |                      |  ^          |           ^     |
  n *     |                      |  j (words)  |          hb_sz BODY_SZ 
 HBLKSIZE |      Object 1        |  v          v           |   (words)
 (bytes)  |                      |---------------          v   MAX_OFFSET
  |       + - - - - - - - - - - -+                        ---  (bytes)
  |       |                      | !All_INTERIOR_PTRS      ^     |
  |       |                      | sets j only for       hb_sz   |
  |       |      Object N        | valid object offsets.   |     |
  v       |                      | All objects WORDSZ      v     v
 ---      +----------------------+ aligned.               ---   ---

DISCARD_WORDS is normally zero.  Indeed the collector has not been tested
with another value in ages.
```
