# Supplementary material for: Evaluation of de novo transcriptome assemblies from RNA-Seq data
Source: Genome Biol. 2014 Dec 21;15(12):553. doi: 10.1186/s13059-014-0553-5 (PMC4298084; doi:10.1186/s13059-014-0553-5)
Supplement: Additional file 3 — Version of the DETONATE source code used for the experiments in this paper. [file 13059_2014_553_MOESM3_ESM.zip › 13059_2014_553_MOESM3_ESM/detonate-1.8.1/ref-eval/boost/tools/build/v2/index.html]

Boost.Build V2


**Quick access**

- Download: [zip]
  , [tar.bz2]
  - Nightly build: [zip],
    [tar.bz2]- Documentation
      (PDF)- Wiki
        (User-contibuted documentation)- Feedback: [mailing list],
          [newsgroup]
          - Before posting, subscribe
          - Bug tracker

## Overview

Boost.Build is an easy way to build C++ projects, everywhere. You
name you executables and libraries and list their sources. Boost.Build
takes care about compiling your sources with right options, creating
static and shared libraries, making executables, and other chores --
whether you're using gcc, msvc, or a dozen more supported C++
compilers -- on Windows, OSX, Linux and commercial UNIX systems.

Some of the most important features:

- **Simple and high level build description**. In most
  cases a name of target and list of sources is all you need.
- **Portability**. Most important build properties have symbolic
  names that work everywhere. Why memorize compiler flags necessary
  for multi-threaded 64-bit shared library, if Boost.Build can do it for you?- **Variant builds**. When you build the same project
    twice with different properties, all produced files are placed
    in different directories, so you can build with 2 versions of
    gcc, or both debug and release variants in one invocation.
  - **Global dependencies**. No matter what directory you build
    in, Boost.Build will always check all dependencies in your entire
    project, preventing inconsistent binaries. And it's easy to
    use one Boost.Build project in other, again with full dependency
    tracking.- **Usage requirements**. A target can specify properties,
      like include paths and preprocessor defines, that are necessary to use
      it. Those properties will be automatically applied whenever the target
      is used.
    - **Standalone**. Boost.Build's only dependency is a C compiler,
      so it's easy to setup. You can even include all of Boost.Build in your
      project. Boost.Build does not depend on C++ Boost in any way.

## Status and future

Boost.Build is ready to use today, and new features are being actively
developed.

The current version of 2.0 Milestone 12, which added support for
precompiled headers on gcc, and added 3 new C++ compilers
(full changelog).

Milestone 13 is planned as bugfix release. Milestone 14 will
focus on improving user documentation. Milestone 15 will see most
of Boost.Build reimplemented in Python, to make extending
Boost.Build even easier for end users (see PythonPort).
The specific issues planned for each release can be found on the
roadmap.

## Feedback and contributing

Should you have any questions or comments, we'd be glad to hear them.
Post everything to the mailing list.

Bugs and feature requests can be entered at our
bug tracker.

If you'd like to help with development, just pick a bug
in the tracker that you'd like to fix, or feel free to implement
any feature you like. There's a separate
guidelines document for working on code.

---

© Copyright David Abrahams and Vladimir Prus 2002-2007.
Permission to copy, use, modify, sell and distribute this document is
granted provided this copyright notice appears in all copies. This
document is provided "as is" without express or implied warranty, and
with no claim as to its suitability for any purpose.

Revised
Oct 4, 2007
