# Supplementary material for: Evaluation of de novo transcriptome assemblies from RNA-Seq data
Source: Genome Biol. 2014 Dec 21;15(12):553. doi: 10.1186/s13059-014-0553-5 (PMC4298084; doi:10.1186/s13059-014-0553-5)
Supplement: Additional file 3 — Version of the DETONATE source code used for the experiments in this paper. [file 13059_2014_553_MOESM3_ESM.zip › 13059_2014_553_MOESM3_ESM/detonate-1.8.1/ref-eval/boost/tools/build/v2/test/test_system.html]

A testing system for Boost.Build


# A testing system for Boost.Build

---

Introduction for users
:   Command line options

Introduction for developers
:   Changing the working directory

    Examining the working directory and changing it

    Test result

Reference documentation
:   Method \_\_init\_\_

    Method set\_tree

    Method write

    Method copy

    Method touch

    Method run\_build\_system

    Method read

    Method read\_and\_strip

    Methods for declaring expectations

    Methods for ignoring changes

    Methods for explicitly specifying results

    Helper class List

---

## Introduction for users

The testing system for Boost.Build is a small set of Python modules and
scripts for automatically testing user-obversable behaviour. It uses
components from testing systems of Scons
and Subversion, together with
some additional functionality.

To run the tests you need to:

1. Get the source tree of Boost.Build (located at tools/build
   in Boost)
2. Have Python installed. Version
   2.1 is known to work.
3. Build Boost.Jam. See $boost\_build\_root/engine/index.html for
   instructions.
4. Configure at least one toolset. You can edit site-config.jam
   or user-config.jam to add new toolsets. Or you can create file
   test-config.jam in $boost\_build\_root/test directory. In
   this case, site-config.jam and user-config.jam will be
   ignored for testing.

When all is set, you can run all the tests using the test\_all.py
script or you can run a specific test by starting its Python script
directly.

Examples:

```
python test_all.py
python generators_test.py
```

If everything is OK, you will see a list of passed tests. Otherwise, a
failure will be reported.

### Command line options

Test scripts will use the toolset you configured to be the default or
you can specify a specific one on the command line:

```
python test_all.py borland
python generators_test.py msvc-7.1
```

Other test script flags you can specify on the command line are:

- --default-bjam -- By default the test system will use the
  Boost Jam executable found built in its default development build
  location. This option makes it use the default one available on your
  system, i.e. the one found in the system path.
- --preserve -- In case of a failed test its working
  directory will be copied to the "failed\_test" directory under the
  current directory.
- --verbose -- Makes the test system and the run build system
  display additional output. Note though that this may cause tests that
  check the build system output to fail.

## Introduction for developers

It is suggested that every new functionality come together with tests,
and that bugfixes are accompanied by tests. There's no need to say that
tests are good, but two points are extremely important:

- For an interpreted language like Jam, without any static checks,
  testing is simply the only sefeguard we can have.
- Good tests allow us to change internal design much more safely, and we
  have not gotten everything nailed down yet.

Adding a new test is simple:

1. Go to $boost\_build\_root/test/test\_all.py and add new test
   name to the list at the end of the file. Suppose the test name is "hello".
2. Add a new python module, in this example "hello.py", to do the actual
   testing.

The module, in general will perform these basic actions:

1. Set up the initial working directory state
2. Run the build system and check the results:
   1. generated output,
   2. changes made to the working directory,
   3. new content of the working directory.
3. Add, remove or touch files or change their content and then repeat
   the previous step until satisfied.
4. Clean up

The "hello.py" module might contain:

```
from BoostBuild import List

# Create a temporary working directory
t = BoostBuild.Tester()

# Create the needed files
t.write("jamroot.jam", "")
t.write("jamfile.jam", """
exe hello : hello.cpp ;
""")
t.write("hello.cpp", """
int main()
{
    return 0;
}

""")

t.run_build_system()

# First, create a list of three pathnames.
file_list = List("bin/$toolset/debug/") * List("hello.exe hello.obj")
# Second, assert that those files were added as result of the last build system invocation.
t.expect_addition(file_list)

# Invoke the build system once again.
t.run_build_system("clean")
# Check if the files added previously were removed.
t.expect_removal(file_list)

# Remove temporary directories
t.cleanup()
```

The test directory contains a file "template.py" which can be
used as a start for your own tests.

Overview of the most important methods of class Tester follows.

### Changing the working directory

The class Tester creates a temporary directory in its
constructor and changes to that directory. It can be modified by calling
these methods:

- set\_tree -- sets the content of the working directory to be
  equal to the content of the specified directory. This method is
  preferrable when directory tree for testing is large.
- write -- sets the content of file in a working directory.
  This is optimal if you want to create a directory tree with 3-4 small
  files.
- touch -- changes the modification times of a file

### Examining the working directory and changing it

The method read, inherited from the TestCmd class, can
be used to read any file in the working directory and check its content.
Tester adds another method for tracking changes. Whenever the build
system is run (using run\_build\_system
), the working dir state before and after running is recorded. In
addition, difference between the two states -- i.e. lists of files that were
added, removed, modified or touched -- are stored in two member variables -
tree\_difference and unexpected\_difference.

After than, the test author may specify that some change is expected, for
example, by calling expect\_addition("foo"). This call will check if
the file was indeed added, and if so, will remove its name from the list of
added files in unexpected\_difference. Likewise, it is possible to
specify that some changes are not interesting, for example a call to
ignore("\*.obj") will just remove every file with the ".obj"
extension from unexpected\_difference.

When test has finished with expectations and ignoring, the member
unexpected\_difference will contain the list of all changes not yet
accounted for. It is possible to assure that this list is empty by calling
the expect\_nothing\_more member function.

### Test result

Any of the expect\* methods below will fail the test if the
expectation is not met. It is also possible to perform manually arbitrary
test and explicitly cause the test to either pass or fail. Ordinary
filesystem functions can be used to work with the directory tree. Methods
pass\_test and fail\_test are used to explicitly give the
test outcome.

Typically, after test termination, the working directory is erased. See
the "--preserve" command line option
for information on how to preserve the working directory content for failed
tests for debugging purposes.

## Reference documentation

The test system is composed of class Tester, derived form
TestCmd.TestCmd, and helper class List. Tester
and List methods are described below.

The documentation frequently refers to filename. In all cases,
files are specified in unix style: a sequence of components, separated by
"/". This is true on all platforms. In some contexts a list of files is
allowed. In those cases any object with a sequence interface is allowed.

### Method \_\_init\_\_(self, arguments="", executable="bjam", match=TestCmd.match\_exact, boost\_build\_path=None, translate\_suffixes=True, pass\_toolset=True, use\_test\_config=True, ignore\_toolset\_requirements=True, workdir="", \*\*keywords)

**Optional arguments:**

- arguments
  -- Arguments passed to the run executable.
- executable
  -- Name of the executable to invoke.
- match
  -- Function to use for compating actual and expected file contents.
- boost\_build\_path
  -- Boost build path to be passed to the run executable.
- translate\_suffixes
  -- Whether to update suffixes on the the file names passed from the
  test script so they match those actually created by the current
  toolset. For example, static library files are specified by using
  the .lib suffix but when the 'gcc' toolset is used it actually
  creates them using the .a suffix.
- pass\_toolset
  -- Whether the test system should pass the specified toolset to the
  run executable.
- use\_test\_config
  -- Whether the test system should tell the run executable to read in
  the test\_config.jam configuration file.
- ignore\_toolset\_requirements
  -- Whether the test system should tell the run executable to ignore
  toolset requirements.
- workdir
  -- Indicates an absolute directory where the test will be run from.

**Optional arguments inherited from the base class:**

- description
  -- Test description string displayed in case of a failed test.
- subdir
  -- List of subdirectories to automatically create under the working
  directory. Each subdirectory needs to be specified separately
  parent coming before its child.
- verbose
  -- Flag that may be used to enable more verbose test system output.
  Note that it does not also enable more verbose build system output
  like the "--verbose" command
  line option does.

**Effects:**

1. Remembers the current working directory in member
   original\_workdir.
2. Determines the location of the executable (`bjam` by
   default) and build system files, assuming that the current directory is
   tools/build/test. Formulates jam invocation command, which
   will include explicit setting for the BOOST\_BUILD\_PATH variable
   and arguments passed to this methods, if any. This command will be used
   by subsequent invocation of 
   run\_build\_system. Finally, initializes the base class.
3. Changes the current working directory to the temporary working
   directory created by the base constructor.
4. If you want to run a test in an existing directory, pass it as
   workdir.
5. Most parameters passed to this constructor function may be overruled
   for each specific test system run using run\_build\_system parameters.

### Method set\_tree(self, tree\_location)

**Effects:**

Replaces the content of the current working directory with the content
of directory at tree\_location. If tree\_location is not
absolute pathname, it will be treated as relative to
self.original\_workdir. This methods also explicitly makes the
copied files writeable.

### Method write(self, name, content)

**Effects:**

Writes the specified content to the file given by name under
the temporary working directory. If the file already exists, it is
overwritten. Any required directories are automatically created.

### Method copy(self, src, dst)

**Effects:**

Equvivalent to self.write(self.read(src), dst).

### Method touch(self, names)

**Effects:**

Sets the access and modification times for all files in names to
the current time. All the elements in names should be relative
paths.

### Method run\_build\_system(self, extra\_args="", subdir="", stdout=None, stderr="", status=0, match=None, pass\_toolset=None, use\_test\_config=None, ignore\_toolset\_requirements=None, expected\_duration=None, \*\*kw)

**Effects:**

1. Stores the state of the working directory in
   self.previous\_tree.
2. Changes to subdir, if it is specified. It is relative to
   the original\_workdir or the workdir specified in
   \_\_init.
3. Invokes the bjam executable, passing extra\_args
   to it. The binary should be located under
   <test\_invocation\_dir>/../jam/src/bin.<platform>.
   This is to make sure tests use the version of jam build from CVS.
4. Compares the stdout, stderr and exit status of build system
   invocation with values to appropriate parameters, if they are not
   None. If any difference is found, the test fails.
5. If the expected\_duration parameter is specified then it
   represents the maximal allowed time in seconds for the test to run. The
   test will be marked as failed if its duration is greater than the given
   expected\_duration parameter value.
6. Stores the new state of the working directory in self.tree.
   Computes the difference between previous and current trees and stores them
   in variables self.tree\_difference and
   self.unexpected\_difference. Both variables are instances of class
   tree.Trees\_different, which have four attributes:
   added\_files, removed\_files, modified\_files and
   touched\_files. Each is a list of strings.

### Method read(self, name)

**Effects:**

Read the specified file and returns it content. Raises an exception is
the file is absent.

### Method read\_and\_strip(self, name)

**Effects:**

Read the specified file and returns it content, after removing trailing
whitespace from every line. Raises an exception is the file is absent.

**Rationale:**

Althought this method is questionable, there are a lot of cases when jam
or shells it uses insert spaces. It seems that introducing this method is
much simpler than dealing with all those cases.

### Methods for declaring expectations

Accordingly to the number of changes kinds that are detected, there are
four methods that specify that test author expects a specific change to
occur. They check self.unexpected\_difference, and if the change is
present there, it is removed. Otherwise, test fails.

Each method accepts a list of names. Those names use / path
separator on all systems. Additionaly, the test system translates suffixes
appropriately. For the test to be portable, suffixes should use Windows
convention: exe for executables, dll for dynamic libraries
and lib for static libraries. Lastly, the string "$toolset" in file
names is replaced by the name of tested toolset.

**Note:** The List helper class might be useful to create
lists of names.

**Note:** The file content can be examined using the
TestCmd.read function.

The members are:

- expect\_addition
- expect\_removal
- expect\_modification
- expect\_nothing

Note that expect\_modification is used to check that a either
file content or timestamp has changed. The rationale is that some compilers
change content even if sources does not change, and it's easier to have a
method which checks for both content and time changes.

There's also a member expect\_nothing\_more, which checks that all
the changes are either expected or ignored, in other words that
unexpected\_difference is empty by now.

Lastly, there's a method to compare file content with expected content:

expect\_content(self, name, content, exact=0)

The method fails the test if the content of file identified by 'name' is
different from 'content'. If 'exact' is true, the file content is used
as-is, otherwise, two transformations are applied:

- The read\_and\_strip method is used to read the file, which
  removes trailing whitespace
- Each backslash in the file content is converted to forward slash.

### Methods for ignoring changes

There are five methods which ignore changes made to the working tree.
They silently remove elements from self.unexpected\_difference, and
don't generate error if element is not found. They accept shell style
wildcard.

The following methods correspond to four kinds of changes:

- ignore\_addition(self, wildcard)
- ignore\_removal(self, wildcard)
- ignore\_modification(self, wildcard)
- ignore\_touch(self, wilcard)

The method ignore(self, wildcard) ignores all the changes made
to files that match a wildcard.

### Methods for explicitly specifying results

#### Method pass\_test(self, condition=1)

At this moment, the method should not be used.

#### Method fail\_test(self, condition=1)

**Effects:** Cause the test to fail if condition is true.

### Helper class List

The class has sequence interface and two additional methods.

#### Method \_\_init\_\_(self, string)

**Effects:** Splits the string on unescaped spaces and tabs. The split
components can further be retrieved using standard sequence access.

#### Method \_\_mul\_\_(self, other)

**Effects:** Returns an List instance, which elements are all
possible concatenations of two string, first of which is from self,
and second of which is from other.

The class also defines \_\_str\_\_ and \_\_repr\_\_ methods.
Finally, there's \_\_coerce\_\_ method which allows to convert strings
to instances of List.

**Example:**

```
    l = "a b" * List("c d")
    for e in l:
        print e
```

will output:

```
    ac
    ad
    bc
    bd
```

---

Last modified: May 02, 2008

© Copyright Vladimir Prus 2002, 2003, 2004, 2005.  
© Copyright Jurko Gospodnetic 2008.  
Distributed under the Boost Software License, Version 1.0.
(See accompanying file LICENSE\_1\_0.txt or http://www.boost.org/LICENSE\_1\_0.txt)
