# Supplementary material for: Evaluation of de novo transcriptome assemblies from RNA-Seq data
Source: Genome Biol. 2014 Dec 21;15(12):553. doi: 10.1186/s13059-014-0553-5 (PMC4298084; doi:10.1186/s13059-014-0553-5)
Supplement: Additional file 3 — Version of the DETONATE source code used for the experiments in this paper. [file 13059_2014_553_MOESM3_ESM.zip › 13059_2014_553_MOESM3_ESM/detonate-1.8.1/ref-eval/cmake/cmake-2.8.12.1.tar.gz!/cmake-2.8.12.1/Tests/CPackComponents/Issue 7470.html]

The install rule for this file demonstrates the problem described in  
CMake issue #7470:  
  

http://public.kitware.com/Bug/view.php?id=7470
