# Supplementary material for: Evaluation of de novo transcriptome assemblies from RNA-Seq data
Source: Genome Biol. 2014 Dec 21;15(12):553. doi: 10.1186/s13059-014-0553-5 (PMC4298084; doi:10.1186/s13059-014-0553-5)
Supplement: Additional file 3 — Version of the DETONATE source code used for the experiments in this paper. [file 13059_2014_553_MOESM3_ESM.zip › 13059_2014_553_MOESM3_ESM/detonate-1.8.1/ref-eval/cmake/cmake-2.8.12.1.tar.gz!/cmake-2.8.12.1/Utilities/cmbzip2/manual.html]

bzip2 and libbzip2, version 1.0.5


# bzip2 and libbzip2, version 1.0.5

## A program and library for data compression

### Julian Seward

http://www.bzip.org

Version 1.0.5 of 10 December 2007

Copyright © 1996-2007 Julian Seward

This program, `bzip2`, the
associated library `libbzip2`, and
all documentation, are copyright © 1996-2007 Julian Seward.
All rights reserved.

Redistribution and use in source and binary forms, with
or without modification, are permitted provided that the
following conditions are met:

- Redistributions of source code must retain the
  above copyright notice, this list of conditions and the
  following disclaimer.
- The origin of this software must not be
  misrepresented; you must not claim that you wrote the original
  software. If you use this software in a product, an
  acknowledgment in the product documentation would be
  appreciated but is not required.
- Altered source versions must be plainly marked
  as such, and must not be misrepresented as being the original
  software.
- The name of the author may not be used to
  endorse or promote products derived from this software without
  specific prior written permission.

THIS SOFTWARE IS PROVIDED BY THE AUTHOR "AS IS" AND ANY
EXPRESS OR IMPLIED WARRANTIES, INCLUDING, BUT NOT LIMITED TO,
THE IMPLIED WARRANTIES OF MERCHANTABILITY AND FITNESS FOR A
PARTICULAR PURPOSE ARE DISCLAIMED. IN NO EVENT SHALL THE
AUTHOR BE LIABLE FOR ANY DIRECT, INDIRECT, INCIDENTAL, SPECIAL,
EXEMPLARY, OR CONSEQUENTIAL DAMAGES (INCLUDING, BUT NOT LIMITED
TO, PROCUREMENT OF SUBSTITUTE GOODS OR SERVICES; LOSS OF USE,
DATA, OR PROFITS; OR BUSINESS INTERRUPTION) HOWEVER CAUSED AND
ON ANY THEORY OF LIABILITY, WHETHER IN CONTRACT, STRICT
LIABILITY, OR TORT (INCLUDING NEGLIGENCE OR OTHERWISE) ARISING
IN ANY WAY OUT OF THE USE OF THIS SOFTWARE, EVEN IF ADVISED OF
THE POSSIBILITY OF SUCH DAMAGE.

PATENTS: To the best of my knowledge,
`bzip2` and
`libbzip2` do not use any patented
algorithms. However, I do not have the resources to carry
out a patent search. Therefore I cannot give any guarantee of
the above statement.

---

**Table of Contents**

1. Introduction

2. How to use bzip2
:   2.1. NAME

    2.2. SYNOPSIS

    2.3. DESCRIPTION

    2.4. OPTIONS

    2.5. MEMORY MANAGEMENT

    2.6. RECOVERING DATA FROM DAMAGED FILES

    2.7. PERFORMANCE NOTES

    2.8. CAVEATS

    2.9. AUTHOR

3. Programming with `libbzip2`
:   3.1. Top-level structure
    :   3.1.1. Low-level summary

        3.1.2. High-level summary

        3.1.3. Utility functions summary

    3.2. Error handling

    3.3. Low-level interface
    :   3.3.1. `BZ2_bzCompressInit`

        3.3.2. `BZ2_bzCompress`

        3.3.3. `BZ2_bzCompressEnd`

        3.3.4. `BZ2_bzDecompressInit`

        3.3.5. `BZ2_bzDecompress`

        3.3.6. `BZ2_bzDecompressEnd`

    3.4. High-level interface
    :   3.4.1. `BZ2_bzReadOpen`

        3.4.2. `BZ2_bzRead`

        3.4.3. `BZ2_bzReadGetUnused`

        3.4.4. `BZ2_bzReadClose`

        3.4.5. `BZ2_bzWriteOpen`

        3.4.6. `BZ2_bzWrite`

        3.4.7. `BZ2_bzWriteClose`

        3.4.8. Handling embedded compressed data streams

        3.4.9. Standard file-reading/writing code

    3.5. Utility functions
    :   3.5.1. `BZ2_bzBuffToBuffCompress`

        3.5.2. `BZ2_bzBuffToBuffDecompress`

    3.6. `zlib` compatibility functions

    3.7. Using the library in a `stdio`-free environment
    :   3.7.1. Getting rid of `stdio`

        3.7.2. Critical error handling

    3.8. Making a Windows DLL

4. Miscellanea
:   4.1. Limitations of the compressed file format

    4.2. Portability issues

    4.3. Reporting bugs

    4.4. Did you get the right package?

    4.5. Further Reading

## 1. Introduction

`bzip2` compresses files
using the Burrows-Wheeler block-sorting text compression
algorithm, and Huffman coding. Compression is generally
considerably better than that achieved by more conventional
LZ77/LZ78-based compressors, and approaches the performance of
the PPM family of statistical compressors.

`bzip2` is built on top of
`libbzip2`, a flexible library for
handling compressed data in the
`bzip2` format. This manual
describes both how to use the program and how to work with the
library interface. Most of the manual is devoted to this
library, not the program, which is good news if your interest is
only in the program.

- How to use bzip2 describes how to use
  `bzip2`; this is the only part
  you need to read if you just want to know how to operate the
  program.
- Programming with libbzip2 describes the
  programming interfaces in detail, and
- Miscellanea records some
  miscellaneous notes which I thought ought to be recorded
  somewhere.

## 2. How to use bzip2

**Table of Contents**

2.1. NAME

2.2. SYNOPSIS

2.3. DESCRIPTION

2.4. OPTIONS

2.5. MEMORY MANAGEMENT

2.6. RECOVERING DATA FROM DAMAGED FILES

2.7. PERFORMANCE NOTES

2.8. CAVEATS

2.9. AUTHOR

This chapter contains a copy of the
`bzip2` man page, and nothing
else.

## 2.1. NAME

- `bzip2`,
  `bunzip2` - a block-sorting file
  compressor, v1.0.4
- `bzcat` -
  decompresses files to stdout
- `bzip2recover` -
  recovers data from damaged bzip2 files

## 2.2. SYNOPSIS

- `bzip2` [
  -cdfkqstvzVL123456789 ] [ filenames ... ]
- `bunzip2` [
  -fkvsVL ] [ filenames ... ]
- `bzcat` [ -s ] [
  filenames ... ]
- `bzip2recover`
  filename

## 2.3. DESCRIPTION

`bzip2` compresses files
using the Burrows-Wheeler block sorting text compression
algorithm, and Huffman coding. Compression is generally
considerably better than that achieved by more conventional
LZ77/LZ78-based compressors, and approaches the performance of
the PPM family of statistical compressors.

The command-line options are deliberately very similar to
those of GNU `gzip`, but they are
not identical.

`bzip2` expects a list of
file names to accompany the command-line flags. Each file is
replaced by a compressed version of itself, with the name
`original_name.bz2`. Each
compressed file has the same modification date, permissions, and,
when possible, ownership as the corresponding original, so that
these properties can be correctly restored at decompression time.
File name handling is naive in the sense that there is no
mechanism for preserving original file names, permissions,
ownerships or dates in filesystems which lack these concepts, or
have serious file name length restrictions, such as
MS-DOS.

`bzip2` and
`bunzip2` will by default not
overwrite existing files. If you want this to happen, specify
the `-f` flag.

If no file names are specified,
`bzip2` compresses from standard
input to standard output. In this case,
`bzip2` will decline to write
compressed output to a terminal, as this would be entirely
incomprehensible and therefore pointless.

`bunzip2` (or
`bzip2 -d`) decompresses all
specified files. Files which were not created by
`bzip2` will be detected and
ignored, and a warning issued.
`bzip2` attempts to guess the
filename for the decompressed file from that of the compressed
file as follows:

- `filename.bz2` 
  becomes
  `filename`
- `filename.bz` 
  becomes
  `filename`
- `filename.tbz2`
  becomes
  `filename.tar`
- `filename.tbz` 
  becomes
  `filename.tar`
- `anyothername` 
  becomes
  `anyothername.out`

If the file does not end in one of the recognised endings,
`.bz2`,
`.bz`,
`.tbz2` or
`.tbz`,
`bzip2` complains that it cannot
guess the name of the original file, and uses the original name
with `.out` appended.

As with compression, supplying no filenames causes
decompression from standard input to standard output.

`bunzip2` will correctly
decompress a file which is the concatenation of two or more
compressed files. The result is the concatenation of the
corresponding uncompressed files. Integrity testing
(`-t`) of concatenated compressed
files is also supported.

You can also compress or decompress files to the standard
output by giving the `-c` flag.
Multiple files may be compressed and decompressed like this. The
resulting outputs are fed sequentially to stdout. Compression of
multiple files in this manner generates a stream containing
multiple compressed file representations. Such a stream can be
decompressed correctly only by
`bzip2` version 0.9.0 or later.
Earlier versions of `bzip2` will
stop after decompressing the first file in the stream.

`bzcat` (or
`bzip2 -dc`) decompresses all
specified files to the standard output.

`bzip2` will read arguments
from the environment variables
`BZIP2` and
`BZIP`, in that order, and will
process them before any arguments read from the command line.
This gives a convenient way to supply default arguments.

Compression is always performed, even if the compressed
file is slightly larger than the original. Files of less than
about one hundred bytes tend to get larger, since the compression
mechanism has a constant overhead in the region of 50 bytes.
Random data (including the output of most file compressors) is
coded at about 8.05 bits per byte, giving an expansion of around
0.5%.

As a self-check for your protection,
`bzip2` uses 32-bit CRCs to make
sure that the decompressed version of a file is identical to the
original. This guards against corruption of the compressed data,
and against undetected bugs in
`bzip2` (hopefully very unlikely).
The chances of data corruption going undetected is microscopic,
about one chance in four billion for each file processed. Be
aware, though, that the check occurs upon decompression, so it
can only tell you that something is wrong. It can't help you
recover the original uncompressed data. You can use
`bzip2recover` to try to recover
data from damaged files.

Return values: 0 for a normal exit, 1 for environmental
problems (file not found, invalid flags, I/O errors, etc.), 2
to indicate a corrupt compressed file, 3 for an internal
consistency error (eg, bug) which caused
`bzip2` to panic.

## 2.4. OPTIONS

`-c --stdout`
:   Compress or decompress to standard
    output.

`-d --decompress`
:   Force decompression.
    `bzip2`,
    `bunzip2` and
    `bzcat` are really the same
    program, and the decision about what actions to take is done on
    the basis of which name is used. This flag overrides that
    mechanism, and forces bzip2 to decompress.

`-z --compress`
:   The complement to
    `-d`: forces compression,
    regardless of the invokation name.

`-t --test`
:   Check integrity of the specified file(s), but
    don't decompress them. This really performs a trial
    decompression and throws away the result.

`-f --force`
:   Force overwrite of output files. Normally,
    `bzip2` will not overwrite
    existing output files. Also forces
    `bzip2` to break hard links to
    files, which it otherwise wouldn't do.

    `bzip2` normally declines
    to decompress files which don't have the correct magic header
    bytes. If forced (`-f`),
    however, it will pass such files through unmodified. This is
    how GNU `gzip` behaves.

`-k --keep`
:   Keep (don't delete) input files during
    compression or decompression.

`-s --small`
:   Reduce memory usage, for compression,
    decompression and testing. Files are decompressed and tested
    using a modified algorithm which only requires 2.5 bytes per
    block byte. This means any file can be decompressed in 2300k
    of memory, albeit at about half the normal speed.

    During compression, `-s`
    selects a block size of 200k, which limits memory use to around
    the same figure, at the expense of your compression ratio. In
    short, if your machine is low on memory (8 megabytes or less),
    use `-s` for everything. See
    MEMORY MANAGEMENT below.

`-q --quiet`
:   Suppress non-essential warning messages.
    Messages pertaining to I/O errors and other critical events
    will not be suppressed.

`-v --verbose`
:   Verbose mode -- show the compression ratio for
    each file processed. Further
    `-v`'s increase the verbosity
    level, spewing out lots of information which is primarily of
    interest for diagnostic purposes.

`-L --license -V --version`
:   Display the software version, license terms and
    conditions.

`-1` (or `--fast`) to `-9` (or `-best`)
:   Set the block size to 100 k, 200 k ... 900 k
    when compressing. Has no effect when decompressing. See MEMORY MANAGEMENT below. The
    `--fast` and
    `--best` aliases are primarily
    for GNU `gzip` compatibility.
    In particular, `--fast` doesn't
    make things significantly faster. And
    `--best` merely selects the
    default behaviour.

`--`
:   Treats all subsequent arguments as file names,
    even if they start with a dash. This is so you can handle
    files with names beginning with a dash, for example:
    `bzip2 --
    -myfilename`.

`--repetitive-fast`, `--repetitive-best`
:   These flags are redundant in versions 0.9.5 and
    above. They provided some coarse control over the behaviour of
    the sorting algorithm in earlier versions, which was sometimes
    useful. 0.9.5 and above have an improved algorithm which
    renders these flags irrelevant.

## 2.5. MEMORY MANAGEMENT

`bzip2` compresses large
files in blocks. The block size affects both the compression
ratio achieved, and the amount of memory needed for compression
and decompression. The flags `-1`
through `-9` specify the block
size to be 100,000 bytes through 900,000 bytes (the default)
respectively. At decompression time, the block size used for
compression is read from the header of the compressed file, and
`bunzip2` then allocates itself
just enough memory to decompress the file. Since block sizes are
stored in compressed files, it follows that the flags
`-1` to
`-9` are irrelevant to and so
ignored during decompression.

Compression and decompression requirements, in bytes, can be
estimated as:

```
Compression:   400k + ( 8 x block size )

Decompression: 100k + ( 4 x block size ), or
               100k + ( 2.5 x block size )
```

Larger block sizes give rapidly diminishing marginal
returns. Most of the compression comes from the first two or
three hundred k of block size, a fact worth bearing in mind when
using `bzip2` on small machines.
It is also important to appreciate that the decompression memory
requirement is set at compression time by the choice of block
size.

For files compressed with the default 900k block size,
`bunzip2` will require about 3700
kbytes to decompress. To support decompression of any file on a
4 megabyte machine, `bunzip2` has
an option to decompress using approximately half this amount of
memory, about 2300 kbytes. Decompression speed is also halved,
so you should use this option only where necessary. The relevant
flag is `-s`.

In general, try and use the largest block size memory
constraints allow, since that maximises the compression achieved.
Compression and decompression speed are virtually unaffected by
block size.

Another significant point applies to files which fit in a
single block -- that means most files you'd encounter using a
large block size. The amount of real memory touched is
proportional to the size of the file, since the file is smaller
than a block. For example, compressing a file 20,000 bytes long
with the flag `-9` will cause the
compressor to allocate around 7600k of memory, but only touch
400k + 20000 \* 8 = 560 kbytes of it. Similarly, the decompressor
will allocate 3700k but only touch 100k + 20000 \* 4 = 180
kbytes.

Here is a table which summarises the maximum memory usage
for different block sizes. Also recorded is the total compressed
size for 14 files of the Calgary Text Compression Corpus
totalling 3,141,622 bytes. This column gives some feel for how
compression varies with block size. These figures tend to
understate the advantage of larger block sizes for larger files,
since the Corpus is dominated by smaller files.

```
        Compress   Decompress   Decompress   Corpus
Flag     usage      usage       -s usage     Size

 -1      1200k       500k         350k      914704
 -2      2000k       900k         600k      877703
 -3      2800k      1300k         850k      860338
 -4      3600k      1700k        1100k      846899
 -5      4400k      2100k        1350k      845160
 -6      5200k      2500k        1600k      838626
 -7      6100k      2900k        1850k      834096
 -8      6800k      3300k        2100k      828642
 -9      7600k      3700k        2350k      828642
```

## 2.6. RECOVERING DATA FROM DAMAGED FILES

`bzip2` compresses files in
blocks, usually 900kbytes long. Each block is handled
independently. If a media or transmission error causes a
multi-block `.bz2` file to become
damaged, it may be possible to recover data from the undamaged
blocks in the file.

The compressed representation of each block is delimited by
a 48-bit pattern, which makes it possible to find the block
boundaries with reasonable certainty. Each block also carries
its own 32-bit CRC, so damaged blocks can be distinguished from
undamaged ones.

`bzip2recover` is a simple
program whose purpose is to search for blocks in
`.bz2` files, and write each block
out into its own `.bz2` file. You
can then use `bzip2 -t` to test
the integrity of the resulting files, and decompress those which
are undamaged.

`bzip2recover` takes a
single argument, the name of the damaged file, and writes a
number of files `rec0001file.bz2`,
`rec0002file.bz2`, etc, containing
the extracted blocks. The output filenames are designed so that
the use of wildcards in subsequent processing -- for example,
`bzip2 -dc rec*file.bz2 >
recovered_data` -- lists the files in the correct
order.

`bzip2recover` should be of
most use dealing with large `.bz2`
files, as these will contain many blocks. It is clearly futile
to use it on damaged single-block files, since a damaged block
cannot be recovered. If you wish to minimise any potential data
loss through media or transmission errors, you might consider
compressing with a smaller block size.

## 2.7. PERFORMANCE NOTES

The sorting phase of compression gathers together similar
strings in the file. Because of this, files containing very long
runs of repeated symbols, like "aabaabaabaab ..." (repeated
several hundred times) may compress more slowly than normal.
Versions 0.9.5 and above fare much better than previous versions
in this respect. The ratio between worst-case and average-case
compression time is in the region of 10:1. For previous
versions, this figure was more like 100:1. You can use the
`-vvvv` option to monitor progress
in great detail, if you want.

Decompression speed is unaffected by these
phenomena.

`bzip2` usually allocates
several megabytes of memory to operate in, and then charges all
over it in a fairly random fashion. This means that performance,
both for compressing and decompressing, is largely determined by
the speed at which your machine can service cache misses.
Because of this, small changes to the code to reduce the miss
rate have been observed to give disproportionately large
performance improvements. I imagine
`bzip2` will perform best on
machines with very large caches.

## 2.8. CAVEATS

I/O error messages are not as helpful as they could be.
`bzip2` tries hard to detect I/O
errors and exit cleanly, but the details of what the problem is
sometimes seem rather misleading.

This manual page pertains to version 1.0.5 of
`bzip2`. Compressed data created by
this version is entirely forwards and backwards compatible with the
previous public releases, versions 0.1pl2, 0.9.0 and 0.9.5, 1.0.0,
1.0.1, 1.0.2 and 1.0.3, but with the following exception: 0.9.0 and
above can correctly decompress multiple concatenated compressed files.
0.1pl2 cannot do this; it will stop after decompressing just the first
file in the stream.

`bzip2recover` versions
prior to 1.0.2 used 32-bit integers to represent bit positions in
compressed files, so it could not handle compressed files more
than 512 megabytes long. Versions 1.0.2 and above use 64-bit ints
on some platforms which support them (GNU supported targets, and
Windows). To establish whether or not
`bzip2recover` was built with such
a limitation, run it without arguments. In any event you can
build yourself an unlimited version if you can recompile it with
`MaybeUInt64` set to be an
unsigned 64-bit integer.

## 2.9. AUTHOR

Julian Seward,
`jseward@bzip.org`

The ideas embodied in
`bzip2` are due to (at least) the
following people: Michael Burrows and David Wheeler (for the
block sorting transformation), David Wheeler (again, for the
Huffman coder), Peter Fenwick (for the structured coding model in
the original `bzip`, and many
refinements), and Alistair Moffat, Radford Neal and Ian Witten
(for the arithmetic coder in the original
`bzip`). I am much indebted for
their help, support and advice. See the manual in the source
distribution for pointers to sources of documentation. Christian
von Roques encouraged me to look for faster sorting algorithms,
so as to speed up compression. Bela Lubkin encouraged me to
improve the worst-case compression performance.
Donna Robinson XMLised the documentation.
Many people sent
patches, helped with portability problems, lent machines, gave
advice and were generally helpful.

## 3.  Programming with `libbzip2`

**Table of Contents**

3.1. Top-level structure
:   3.1.1. Low-level summary

    3.1.2. High-level summary

    3.1.3. Utility functions summary

3.2. Error handling

3.3. Low-level interface
:   3.3.1. `BZ2_bzCompressInit`

    3.3.2. `BZ2_bzCompress`

    3.3.3. `BZ2_bzCompressEnd`

    3.3.4. `BZ2_bzDecompressInit`

    3.3.5. `BZ2_bzDecompress`

    3.3.6. `BZ2_bzDecompressEnd`

3.4. High-level interface
:   3.4.1. `BZ2_bzReadOpen`

    3.4.2. `BZ2_bzRead`

    3.4.3. `BZ2_bzReadGetUnused`

    3.4.4. `BZ2_bzReadClose`

    3.4.5. `BZ2_bzWriteOpen`

    3.4.6. `BZ2_bzWrite`

    3.4.7. `BZ2_bzWriteClose`

    3.4.8. Handling embedded compressed data streams

    3.4.9. Standard file-reading/writing code

3.5. Utility functions
:   3.5.1. `BZ2_bzBuffToBuffCompress`

    3.5.2. `BZ2_bzBuffToBuffDecompress`

3.6. `zlib` compatibility functions

3.7. Using the library in a `stdio`-free environment
:   3.7.1. Getting rid of `stdio`

    3.7.2. Critical error handling

3.8. Making a Windows DLL

This chapter describes the programming interface to
`libbzip2`.

For general background information, particularly about
memory use and performance aspects, you'd be well advised to read
How to use bzip2 as well.

## 3.1. Top-level structure

`libbzip2` is a flexible
library for compressing and decompressing data in the
`bzip2` data format. Although
packaged as a single entity, it helps to regard the library as
three separate parts: the low level interface, and the high level
interface, and some utility functions.

The structure of
`libbzip2`'s interfaces is similar
to that of Jean-loup Gailly's and Mark Adler's excellent
`zlib` library.

All externally visible symbols have names beginning
`BZ2_`. This is new in version
1.0. The intention is to minimise pollution of the namespaces of
library clients.

To use any part of the library, you need to
`#include <bzlib.h>`
into your sources.

### 3.1.1. Low-level summary

This interface provides services for compressing and
decompressing data in memory. There's no provision for dealing
with files, streams or any other I/O mechanisms, just straight
memory-to-memory work. In fact, this part of the library can be
compiled without inclusion of
`stdio.h`, which may be helpful
for embedded applications.

The low-level part of the library has no global variables
and is therefore thread-safe.

Six routines make up the low level interface:
`BZ2_bzCompressInit`,
`BZ2_bzCompress`, and
`BZ2_bzCompressEnd` for
compression, and a corresponding trio
`BZ2_bzDecompressInit`,
`BZ2_bzDecompress` and
`BZ2_bzDecompressEnd` for
decompression. The `*Init`
functions allocate memory for compression/decompression and do
other initialisations, whilst the
`*End` functions close down
operations and release memory.

The real work is done by
`BZ2_bzCompress` and
`BZ2_bzDecompress`. These
compress and decompress data from a user-supplied input buffer to
a user-supplied output buffer. These buffers can be any size;
arbitrary quantities of data are handled by making repeated calls
to these functions. This is a flexible mechanism allowing a
consumer-pull style of activity, or producer-push, or a mixture
of both.

### 3.1.2. High-level summary

This interface provides some handy wrappers around the
low-level interface to facilitate reading and writing
`bzip2` format files
(`.bz2` files). The routines
provide hooks to facilitate reading files in which the
`bzip2` data stream is embedded
within some larger-scale file structure, or where there are
multiple `bzip2` data streams
concatenated end-to-end.

For reading files,
`BZ2_bzReadOpen`,
`BZ2_bzRead`,
`BZ2_bzReadClose` and
`BZ2_bzReadGetUnused` are
supplied. For writing files,
`BZ2_bzWriteOpen`,
`BZ2_bzWrite` and
`BZ2_bzWriteFinish` are
available.

As with the low-level library, no global variables are used
so the library is per se thread-safe. However, if I/O errors
occur whilst reading or writing the underlying compressed files,
you may have to consult `errno` to
determine the cause of the error. In that case, you'd need a C
library which correctly supports
`errno` in a multithreaded
environment.

To make the library a little simpler and more portable,
`BZ2_bzReadOpen` and
`BZ2_bzWriteOpen` require you to
pass them file handles (`FILE*`s)
which have previously been opened for reading or writing
respectively. That avoids portability problems associated with
file operations and file attributes, whilst not being much of an
imposition on the programmer.

### 3.1.3. Utility functions summary

For very simple needs,
`BZ2_bzBuffToBuffCompress` and
`BZ2_bzBuffToBuffDecompress` are
provided. These compress data in memory from one buffer to
another buffer in a single function call. You should assess
whether these functions fulfill your memory-to-memory
compression/decompression requirements before investing effort in
understanding the more general but more complex low-level
interface.

Yoshioka Tsuneo
(`tsuneo@rr.iij4u.or.jp`) has
contributed some functions to give better
`zlib` compatibility. These
functions are `BZ2_bzopen`,
`BZ2_bzread`,
`BZ2_bzwrite`,
`BZ2_bzflush`,
`BZ2_bzclose`,
`BZ2_bzerror` and
`BZ2_bzlibVersion`. You may find
these functions more convenient for simple file reading and
writing, than those in the high-level interface. These functions
are not (yet) officially part of the library, and are minimally
documented here. If they break, you get to keep all the pieces.
I hope to document them properly when time permits.

Yoshioka also contributed modifications to allow the
library to be built as a Windows DLL.

## 3.2. Error handling

The library is designed to recover cleanly in all
situations, including the worst-case situation of decompressing
random data. I'm not 100% sure that it can always do this, so
you might want to add a signal handler to catch segmentation
violations during decompression if you are feeling especially
paranoid. I would be interested in hearing more about the
robustness of the library to corrupted compressed data.

Version 1.0.3 more robust in this respect than any
previous version. Investigations with Valgrind (a tool for detecting
problems with memory management) indicate
that, at least for the few files I tested, all single-bit errors
in the decompressed data are caught properly, with no
segmentation faults, no uses of uninitialised data, no out of
range reads or writes, and no infinite looping in the decompressor.
So it's certainly pretty robust, although
I wouldn't claim it to be totally bombproof.

The file `bzlib.h` contains
all definitions needed to use the library. In particular, you
should definitely not include
`bzlib_private.h`.

In `bzlib.h`, the various
return values are defined. The following list is not intended as
an exhaustive description of the circumstances in which a given
value may be returned -- those descriptions are given later.
Rather, it is intended to convey the rough meaning of each return
value. The first five actions are normal and not intended to
denote an error situation.

`BZ_OK`
:   The requested action was completed
    successfully.

`BZ_RUN_OK, BZ_FLUSH_OK, BZ_FINISH_OK`
:   In
    `BZ2_bzCompress`, the requested
    flush/finish/nothing-special action was completed
    successfully.

`BZ_STREAM_END`
:   Compression of data was completed, or the
    logical stream end was detected during
    decompression.

The following return values indicate an error of some
kind.

`BZ_CONFIG_ERROR`
:   Indicates that the library has been improperly
    compiled on your platform -- a major configuration error.
    Specifically, it means that
    `sizeof(char)`,
    `sizeof(short)` and
    `sizeof(int)` are not 1, 2 and
    4 respectively, as they should be. Note that the library
    should still work properly on 64-bit platforms which follow
    the LP64 programming model -- that is, where
    `sizeof(long)` and
    `sizeof(void*)` are 8. Under
    LP64, `sizeof(int)` is still 4,
    so `libbzip2`, which doesn't
    use the `long` type, is
    OK.

`BZ_SEQUENCE_ERROR`
:   When using the library, it is important to call
    the functions in the correct sequence and with data structures
    (buffers etc) in the correct states.
    `libbzip2` checks as much as it
    can to ensure this is happening, and returns
    `BZ_SEQUENCE_ERROR` if not.
    Code which complies precisely with the function semantics, as
    detailed below, should never receive this value; such an event
    denotes buggy code which you should
    investigate.

`BZ_PARAM_ERROR`
:   Returned when a parameter to a function call is
    out of range or otherwise manifestly incorrect. As with
    `BZ_SEQUENCE_ERROR`, this
    denotes a bug in the client code. The distinction between
    `BZ_PARAM_ERROR` and
    `BZ_SEQUENCE_ERROR` is a bit
    hazy, but still worth making.

`BZ_MEM_ERROR`
:   Returned when a request to allocate memory
    failed. Note that the quantity of memory needed to decompress
    a stream cannot be determined until the stream's header has
    been read. So
    `BZ2_bzDecompress` and
    `BZ2_bzRead` may return
    `BZ_MEM_ERROR` even though some
    of the compressed data has been read. The same is not true
    for compression; once
    `BZ2_bzCompressInit` or
    `BZ2_bzWriteOpen` have
    successfully completed,
    `BZ_MEM_ERROR` cannot
    occur.

`BZ_DATA_ERROR`
:   Returned when a data integrity error is
    detected during decompression. Most importantly, this means
    when stored and computed CRCs for the data do not match. This
    value is also returned upon detection of any other anomaly in
    the compressed data.

`BZ_DATA_ERROR_MAGIC`
:   As a special case of
    `BZ_DATA_ERROR`, it is
    sometimes useful to know when the compressed stream does not
    start with the correct magic bytes (`'B' 'Z'
    'h'`).

`BZ_IO_ERROR`
:   Returned by
    `BZ2_bzRead` and
    `BZ2_bzWrite` when there is an
    error reading or writing in the compressed file, and by
    `BZ2_bzReadOpen` and
    `BZ2_bzWriteOpen` for attempts
    to use a file for which the error indicator (viz,
    `ferror(f)`) is set. On
    receipt of `BZ_IO_ERROR`, the
    caller should consult `errno`
    and/or `perror` to acquire
    operating-system specific information about the
    problem.

`BZ_UNEXPECTED_EOF`
:   Returned by
    `BZ2_bzRead` when the
    compressed file finishes before the logical end of stream is
    detected.

`BZ_OUTBUFF_FULL`
:   Returned by
    `BZ2_bzBuffToBuffCompress` and
    `BZ2_bzBuffToBuffDecompress` to
    indicate that the output data will not fit into the output
    buffer provided.

## 3.3. Low-level interface

### 3.3.1. `BZ2_bzCompressInit`

```
typedef struct {
  char *next_in;
  unsigned int avail_in;
  unsigned int total_in_lo32;
  unsigned int total_in_hi32;

  char *next_out;
  unsigned int avail_out;
  unsigned int total_out_lo32;
  unsigned int total_out_hi32;

  void *state;

  void *(*bzalloc)(void *,int,int);
  void (*bzfree)(void *,void *);
  void *opaque;
} bz_stream;

int BZ2_bzCompressInit ( bz_stream *strm, 
                         int blockSize100k, 
                         int verbosity,
                         int workFactor );
```

Prepares for compression. The
`bz_stream` structure holds all
data pertaining to the compression activity. A
`bz_stream` structure should be
allocated and initialised prior to the call. The fields of
`bz_stream` comprise the entirety
of the user-visible data. `state`
is a pointer to the private data structures required for
compression.

Custom memory allocators are supported, via fields
`bzalloc`,
`bzfree`, and
`opaque`. The value
`opaque` is passed to as the first
argument to all calls to `bzalloc`
and `bzfree`, but is otherwise
ignored by the library. The call `bzalloc (
opaque, n, m )` is expected to return a pointer
`p` to `n *
m` bytes of memory, and `bzfree (
opaque, p )` should free that memory.

If you don't want to use a custom memory allocator, set
`bzalloc`,
`bzfree` and
`opaque` to
`NULL`, and the library will then
use the standard `malloc` /
`free` routines.

Before calling
`BZ2_bzCompressInit`, fields
`bzalloc`,
`bzfree` and
`opaque` should be filled
appropriately, as just described. Upon return, the internal
state will have been allocated and initialised, and
`total_in_lo32`,
`total_in_hi32`,
`total_out_lo32` and
`total_out_hi32` will have been
set to zero. These four fields are used by the library to inform
the caller of the total amount of data passed into and out of the
library, respectively. You should not try to change them. As of
version 1.0, 64-bit counts are maintained, even on 32-bit
platforms, using the `_hi32`
fields to store the upper 32 bits of the count. So, for example,
the total amount of data in is `(total_in_hi32
<< 32) + total_in_lo32`.

Parameter `blockSize100k`
specifies the block size to be used for compression. It should
be a value between 1 and 9 inclusive, and the actual block size
used is 100000 x this figure. 9 gives the best compression but
takes most memory.

Parameter `verbosity` should
be set to a number between 0 and 4 inclusive. 0 is silent, and
greater numbers give increasingly verbose monitoring/debugging
output. If the library has been compiled with
`-DBZ_NO_STDIO`, no such output
will appear for any verbosity setting.

Parameter `workFactor`
controls how the compression phase behaves when presented with
worst case, highly repetitive, input data. If compression runs
into difficulties caused by repetitive data, the library switches
from the standard sorting algorithm to a fallback algorithm. The
fallback is slower than the standard algorithm by perhaps a
factor of three, but always behaves reasonably, no matter how bad
the input.

Lower values of `workFactor`
reduce the amount of effort the standard algorithm will expend
before resorting to the fallback. You should set this parameter
carefully; too low, and many inputs will be handled by the
fallback algorithm and so compress rather slowly, too high, and
your average-to-worst case compression times can become very
large. The default value of 30 gives reasonable behaviour over a
wide range of circumstances.

Allowable values range from 0 to 250 inclusive. 0 is a
special case, equivalent to using the default value of 30.

Note that the compressed output generated is the same
regardless of whether or not the fallback algorithm is
used.

Be aware also that this parameter may disappear entirely in
future versions of the library. In principle it should be
possible to devise a good way to automatically choose which
algorithm to use. Such a mechanism would render the parameter
obsolete.

Possible return values:

```
BZ_CONFIG_ERROR
  if the library has been mis-compiled
BZ_PARAM_ERROR
  if strm is NULL 
  or blockSize < 1 or blockSize > 9
  or verbosity < 0 or verbosity > 4
  or workFactor < 0 or workFactor > 250
BZ_MEM_ERROR 
  if not enough memory is available
BZ_OK 
  otherwise
```

Allowable next actions:

```
BZ2_bzCompress
  if BZ_OK is returned
  no specific action needed in case of error
```

### 3.3.2. `BZ2_bzCompress`

```
int BZ2_bzCompress ( bz_stream *strm, int action );
```

Provides more input and/or output buffer space for the
library. The caller maintains input and output buffers, and
calls `BZ2_bzCompress` to transfer
data between them.

Before each call to
`BZ2_bzCompress`,
`next_in` should point at the data
to be compressed, and `avail_in`
should indicate how many bytes the library may read.
`BZ2_bzCompress` updates
`next_in`,
`avail_in` and
`total_in` to reflect the number
of bytes it has read.

Similarly, `next_out` should
point to a buffer in which the compressed data is to be placed,
with `avail_out` indicating how
much output space is available.
`BZ2_bzCompress` updates
`next_out`,
`avail_out` and
`total_out` to reflect the number
of bytes output.

You may provide and remove as little or as much data as you
like on each call of
`BZ2_bzCompress`. In the limit,
it is acceptable to supply and remove data one byte at a time,
although this would be terribly inefficient. You should always
ensure that at least one byte of output space is available at
each call.

A second purpose of
`BZ2_bzCompress` is to request a
change of mode of the compressed stream.

Conceptually, a compressed stream can be in one of four
states: IDLE, RUNNING, FLUSHING and FINISHING. Before
initialisation
(`BZ2_bzCompressInit`) and after
termination (`BZ2_bzCompressEnd`),
a stream is regarded as IDLE.

Upon initialisation
(`BZ2_bzCompressInit`), the stream
is placed in the RUNNING state. Subsequent calls to
`BZ2_bzCompress` should pass
`BZ_RUN` as the requested action;
other actions are illegal and will result in
`BZ_SEQUENCE_ERROR`.

At some point, the calling program will have provided all
the input data it wants to. It will then want to finish up -- in
effect, asking the library to process any data it might have
buffered internally. In this state,
`BZ2_bzCompress` will no longer
attempt to read data from
`next_in`, but it will want to
write data to `next_out`. Because
the output buffer supplied by the user can be arbitrarily small,
the finishing-up operation cannot necessarily be done with a
single call of
`BZ2_bzCompress`.

Instead, the calling program passes
`BZ_FINISH` as an action to
`BZ2_bzCompress`. This changes
the stream's state to FINISHING. Any remaining input (ie,
`next_in[0 .. avail_in-1]`) is
compressed and transferred to the output buffer. To do this,
`BZ2_bzCompress` must be called
repeatedly until all the output has been consumed. At that
point, `BZ2_bzCompress` returns
`BZ_STREAM_END`, and the stream's
state is set back to IDLE.
`BZ2_bzCompressEnd` should then be
called.

Just to make sure the calling program does not cheat, the
library makes a note of `avail_in`
at the time of the first call to
`BZ2_bzCompress` which has
`BZ_FINISH` as an action (ie, at
the time the program has announced its intention to not supply
any more input). By comparing this value with that of
`avail_in` over subsequent calls
to `BZ2_bzCompress`, the library
can detect any attempts to slip in more data to compress. Any
calls for which this is detected will return
`BZ_SEQUENCE_ERROR`. This
indicates a programming mistake which should be corrected.

Instead of asking to finish, the calling program may ask
`BZ2_bzCompress` to take all the
remaining input, compress it and terminate the current
(Burrows-Wheeler) compression block. This could be useful for
error control purposes. The mechanism is analogous to that for
finishing: call `BZ2_bzCompress`
with an action of `BZ_FLUSH`,
remove output data, and persist with the
`BZ_FLUSH` action until the value
`BZ_RUN` is returned. As with
finishing, `BZ2_bzCompress`
detects any attempt to provide more input data once the flush has
begun.

Once the flush is complete, the stream returns to the
normal RUNNING state.

This all sounds pretty complex, but isn't really. Here's a
table which shows which actions are allowable in each state, what
action will be taken, what the next state is, and what the
non-error return values are. Note that you can't explicitly ask
what state the stream is in, but nor do you need to -- it can be
inferred from the values returned by
`BZ2_bzCompress`.

```
IDLE/any
  Illegal.  IDLE state only exists after BZ2_bzCompressEnd or
  before BZ2_bzCompressInit.
  Return value = BZ_SEQUENCE_ERROR

RUNNING/BZ_RUN
  Compress from next_in to next_out as much as possible.
  Next state = RUNNING
  Return value = BZ_RUN_OK

RUNNING/BZ_FLUSH
  Remember current value of next_in. Compress from next_in
  to next_out as much as possible, but do not accept any more input.
  Next state = FLUSHING
  Return value = BZ_FLUSH_OK

RUNNING/BZ_FINISH
  Remember current value of next_in. Compress from next_in
  to next_out as much as possible, but do not accept any more input.
  Next state = FINISHING
  Return value = BZ_FINISH_OK

FLUSHING/BZ_FLUSH
  Compress from next_in to next_out as much as possible, 
  but do not accept any more input.
  If all the existing input has been used up and all compressed
  output has been removed
    Next state = RUNNING; Return value = BZ_RUN_OK
  else
    Next state = FLUSHING; Return value = BZ_FLUSH_OK

FLUSHING/other     
  Illegal.
  Return value = BZ_SEQUENCE_ERROR

FINISHING/BZ_FINISH
  Compress from next_in to next_out as much as possible,
  but to not accept any more input.  
  If all the existing input has been used up and all compressed
  output has been removed
    Next state = IDLE; Return value = BZ_STREAM_END
  else
    Next state = FINISHING; Return value = BZ_FINISH_OK

FINISHING/other
  Illegal.
  Return value = BZ_SEQUENCE_ERROR
```

That still looks complicated? Well, fair enough. The
usual sequence of calls for compressing a load of data is:

1. Get started with
   `BZ2_bzCompressInit`.
2. Shovel data in and shlurp out its compressed form
   using zero or more calls of
   `BZ2_bzCompress` with action =
   `BZ_RUN`.
3. Finish up. Repeatedly call
   `BZ2_bzCompress` with action =
   `BZ_FINISH`, copying out the
   compressed output, until
   `BZ_STREAM_END` is
   returned.
4. Close up and go home. Call
   `BZ2_bzCompressEnd`.

If the data you want to compress fits into your input
buffer all at once, you can skip the calls of
`BZ2_bzCompress ( ..., BZ_RUN )`
and just do the `BZ2_bzCompress ( ..., BZ_FINISH
)` calls.

All required memory is allocated by
`BZ2_bzCompressInit`. The
compression library can accept any data at all (obviously). So
you shouldn't get any error return values from the
`BZ2_bzCompress` calls. If you
do, they will be
`BZ_SEQUENCE_ERROR`, and indicate
a bug in your programming.

Trivial other possible return values:

```
BZ_PARAM_ERROR
  if strm is NULL, or strm->s is NULL
```

### 3.3.3. `BZ2_bzCompressEnd`

```
int BZ2_bzCompressEnd ( bz_stream *strm );
```

Releases all memory associated with a compression
stream.

Possible return values:

```
BZ_PARAM_ERROR  if strm is NULL or strm->s is NULL
BZ_OK           otherwise
```

### 3.3.4. `BZ2_bzDecompressInit`

```
int BZ2_bzDecompressInit ( bz_stream *strm, int verbosity, int small );
```

Prepares for decompression. As with
`BZ2_bzCompressInit`, a
`bz_stream` record should be
allocated and initialised before the call. Fields
`bzalloc`,
`bzfree` and
`opaque` should be set if a custom
memory allocator is required, or made
`NULL` for the normal
`malloc` /
`free` routines. Upon return, the
internal state will have been initialised, and
`total_in` and
`total_out` will be zero.

For the meaning of parameter
`verbosity`, see
`BZ2_bzCompressInit`.

If `small` is nonzero, the
library will use an alternative decompression algorithm which
uses less memory but at the cost of decompressing more slowly
(roughly speaking, half the speed, but the maximum memory
requirement drops to around 2300k). See How to use bzip2
for more information on memory management.

Note that the amount of memory needed to decompress a
stream cannot be determined until the stream's header has been
read, so even if
`BZ2_bzDecompressInit` succeeds, a
subsequent `BZ2_bzDecompress`
could fail with
`BZ_MEM_ERROR`.

Possible return values:

```
BZ_CONFIG_ERROR
  if the library has been mis-compiled
BZ_PARAM_ERROR
  if ( small != 0 && small != 1 )
  or (verbosity <; 0 || verbosity > 4)
BZ_MEM_ERROR
  if insufficient memory is available
```

Allowable next actions:

```
BZ2_bzDecompress
  if BZ_OK was returned
  no specific action required in case of error
```

### 3.3.5. `BZ2_bzDecompress`

```
int BZ2_bzDecompress ( bz_stream *strm );
```

Provides more input and/out output buffer space for the
library. The caller maintains input and output buffers, and uses
`BZ2_bzDecompress` to transfer
data between them.

Before each call to
`BZ2_bzDecompress`,
`next_in` should point at the
compressed data, and `avail_in`
should indicate how many bytes the library may read.
`BZ2_bzDecompress` updates
`next_in`,
`avail_in` and
`total_in` to reflect the number
of bytes it has read.

Similarly, `next_out` should
point to a buffer in which the uncompressed output is to be
placed, with `avail_out`
indicating how much output space is available.
`BZ2_bzCompress` updates
`next_out`,
`avail_out` and
`total_out` to reflect the number
of bytes output.

You may provide and remove as little or as much data as you
like on each call of
`BZ2_bzDecompress`. In the limit,
it is acceptable to supply and remove data one byte at a time,
although this would be terribly inefficient. You should always
ensure that at least one byte of output space is available at
each call.

Use of `BZ2_bzDecompress` is
simpler than
`BZ2_bzCompress`.

You should provide input and remove output as described
above, and repeatedly call
`BZ2_bzDecompress` until
`BZ_STREAM_END` is returned.
Appearance of `BZ_STREAM_END`
denotes that `BZ2_bzDecompress`
has detected the logical end of the compressed stream.
`BZ2_bzDecompress` will not
produce `BZ_STREAM_END` until all
output data has been placed into the output buffer, so once
`BZ_STREAM_END` appears, you are
guaranteed to have available all the decompressed output, and
`BZ2_bzDecompressEnd` can safely
be called.

If case of an error return value, you should call
`BZ2_bzDecompressEnd` to clean up
and release memory.

Possible return values:

```
BZ_PARAM_ERROR
  if strm is NULL or strm->s is NULL
  or strm->avail_out < 1
BZ_DATA_ERROR
  if a data integrity error is detected in the compressed stream
BZ_DATA_ERROR_MAGIC
  if the compressed stream doesn't begin with the right magic bytes
BZ_MEM_ERROR
  if there wasn't enough memory available
BZ_STREAM_END
  if the logical end of the data stream was detected and all
  output in has been consumed, eg s-->avail_out > 0
BZ_OK
  otherwise
```

Allowable next actions:

```
BZ2_bzDecompress
  if BZ_OK was returned
BZ2_bzDecompressEnd
  otherwise
```

### 3.3.6. `BZ2_bzDecompressEnd`

```
int BZ2_bzDecompressEnd ( bz_stream *strm );
```

Releases all memory associated with a decompression
stream.

Possible return values:

```
BZ_PARAM_ERROR
  if strm is NULL or strm->s is NULL
BZ_OK
  otherwise
```

Allowable next actions:

```
  None.
```

## 3.4. High-level interface

This interface provides functions for reading and writing
`bzip2` format files. First, some
general points.

- All of the functions take an
  `int*` first argument,
  `bzerror`. After each call,
  `bzerror` should be consulted
  first to determine the outcome of the call. If
  `bzerror` is
  `BZ_OK`, the call completed
  successfully, and only then should the return value of the
  function (if any) be consulted. If
  `bzerror` is
  `BZ_IO_ERROR`, there was an
  error reading/writing the underlying compressed file, and you
  should then consult `errno` /
  `perror` to determine the cause
  of the difficulty. `bzerror`
  may also be set to various other values; precise details are
  given on a per-function basis below.
- If `bzerror` indicates
  an error (ie, anything except
  `BZ_OK` and
  `BZ_STREAM_END`), you should
  immediately call
  `BZ2_bzReadClose` (or
  `BZ2_bzWriteClose`, depending on
  whether you are attempting to read or to write) to free up all
  resources associated with the stream. Once an error has been
  indicated, behaviour of all calls except
  `BZ2_bzReadClose`
  (`BZ2_bzWriteClose`) is
  undefined. The implication is that (1)
  `bzerror` should be checked
  after each call, and (2) if
  `bzerror` indicates an error,
  `BZ2_bzReadClose`
  (`BZ2_bzWriteClose`) should then
  be called to clean up.
- The `FILE*` arguments
  passed to `BZ2_bzReadOpen` /
  `BZ2_bzWriteOpen` should be set
  to binary mode. Most Unix systems will do this by default, but
  other platforms, including Windows and Mac, will not. If you
  omit this, you may encounter problems when moving code to new
  platforms.
- Memory allocation requests are handled by
  `malloc` /
  `free`. At present there is no
  facility for user-defined memory allocators in the file I/O
  functions (could easily be added, though).

### 3.4.1. `BZ2_bzReadOpen`

```
typedef void BZFILE;

BZFILE *BZ2_bzReadOpen( int *bzerror, FILE *f, 
                        int verbosity, int small,
                        void *unused, int nUnused );
```

Prepare to read compressed data from file handle
`f`.
`f` should refer to a file which
has been opened for reading, and for which the error indicator
(`ferror(f)`)is not set. If
`small` is 1, the library will try
to decompress using less memory, at the expense of speed.

For reasons explained below,
`BZ2_bzRead` will decompress the
`nUnused` bytes starting at
`unused`, before starting to read
from the file `f`. At most
`BZ_MAX_UNUSED` bytes may be
supplied like this. If this facility is not required, you should
pass `NULL` and
`0` for
`unused` and
n`Unused` respectively.

For the meaning of parameters
`small` and
`verbosity`, see
`BZ2_bzDecompressInit`.

The amount of memory needed to decompress a file cannot be
determined until the file's header has been read. So it is
possible that `BZ2_bzReadOpen`
returns `BZ_OK` but a subsequent
call of `BZ2_bzRead` will return
`BZ_MEM_ERROR`.

Possible assignments to
`bzerror`:

```
BZ_CONFIG_ERROR
  if the library has been mis-compiled
BZ_PARAM_ERROR
  if f is NULL
  or small is neither 0 nor 1
  or ( unused == NULL && nUnused != 0 )
  or ( unused != NULL && !(0 <= nUnused <= BZ_MAX_UNUSED) )
BZ_IO_ERROR
  if ferror(f) is nonzero
BZ_MEM_ERROR
  if insufficient memory is available
BZ_OK
  otherwise.
```

Possible return values:

```
Pointer to an abstract BZFILE
  if bzerror is BZ_OK
NULL
  otherwise
```

Allowable next actions:

```
BZ2_bzRead
  if bzerror is BZ_OK
BZ2_bzClose
  otherwise
```

### 3.4.2. `BZ2_bzRead`

```
int BZ2_bzRead ( int *bzerror, BZFILE *b, void *buf, int len );
```

Reads up to `len`
(uncompressed) bytes from the compressed file
`b` into the buffer
`buf`. If the read was
successful, `bzerror` is set to
`BZ_OK` and the number of bytes
read is returned. If the logical end-of-stream was detected,
`bzerror` will be set to
`BZ_STREAM_END`, and the number of
bytes read is returned. All other
`bzerror` values denote an
error.

`BZ2_bzRead` will supply
`len` bytes, unless the logical
stream end is detected or an error occurs. Because of this, it
is possible to detect the stream end by observing when the number
of bytes returned is less than the number requested.
Nevertheless, this is regarded as inadvisable; you should instead
check `bzerror` after every call
and watch out for
`BZ_STREAM_END`.

Internally, `BZ2_bzRead`
copies data from the compressed file in chunks of size
`BZ_MAX_UNUSED` bytes before
decompressing it. If the file contains more bytes than strictly
needed to reach the logical end-of-stream,
`BZ2_bzRead` will almost certainly
read some of the trailing data before signalling
`BZ_SEQUENCE_END`. To collect the
read but unused data once
`BZ_SEQUENCE_END` has appeared,
call `BZ2_bzReadGetUnused`
immediately before
`BZ2_bzReadClose`.

Possible assignments to
`bzerror`:

```
BZ_PARAM_ERROR
  if b is NULL or buf is NULL or len < 0
BZ_SEQUENCE_ERROR
  if b was opened with BZ2_bzWriteOpen
BZ_IO_ERROR
  if there is an error reading from the compressed file
BZ_UNEXPECTED_EOF
  if the compressed file ended before 
  the logical end-of-stream was detected
BZ_DATA_ERROR
  if a data integrity error was detected in the compressed stream
BZ_DATA_ERROR_MAGIC
  if the stream does not begin with the requisite header bytes 
  (ie, is not a bzip2 data file).  This is really 
  a special case of BZ_DATA_ERROR.
BZ_MEM_ERROR
  if insufficient memory was available
BZ_STREAM_END
  if the logical end of stream was detected.
BZ_OK
  otherwise.
```

Possible return values:

```
number of bytes read
  if bzerror is BZ_OK or BZ_STREAM_END
undefined
  otherwise
```

Allowable next actions:

```
collect data from buf, then BZ2_bzRead or BZ2_bzReadClose
  if bzerror is BZ_OK
collect data from buf, then BZ2_bzReadClose or BZ2_bzReadGetUnused
  if bzerror is BZ_SEQUENCE_END
BZ2_bzReadClose
  otherwise
```

### 3.4.3. `BZ2_bzReadGetUnused`

```
void BZ2_bzReadGetUnused( int* bzerror, BZFILE *b, 
                          void** unused, int* nUnused );
```

Returns data which was read from the compressed file but
was not needed to get to the logical end-of-stream.
`*unused` is set to the address of
the data, and `*nUnused` to the
number of bytes. `*nUnused` will
be set to a value between `0` and
`BZ_MAX_UNUSED` inclusive.

This function may only be called once
`BZ2_bzRead` has signalled
`BZ_STREAM_END` but before
`BZ2_bzReadClose`.

Possible assignments to
`bzerror`:

```
BZ_PARAM_ERROR
  if b is NULL
  or unused is NULL or nUnused is NULL
BZ_SEQUENCE_ERROR
  if BZ_STREAM_END has not been signalled
  or if b was opened with BZ2_bzWriteOpen
BZ_OK
  otherwise
```

Allowable next actions:

```
BZ2_bzReadClose
```

### 3.4.4. `BZ2_bzReadClose`

```
void BZ2_bzReadClose ( int *bzerror, BZFILE *b );
```

Releases all memory pertaining to the compressed file
`b`.
`BZ2_bzReadClose` does not call
`fclose` on the underlying file
handle, so you should do that yourself if appropriate.
`BZ2_bzReadClose` should be called
to clean up after all error situations.

Possible assignments to
`bzerror`:

```
BZ_SEQUENCE_ERROR
  if b was opened with BZ2_bzOpenWrite
BZ_OK
  otherwise
```

Allowable next actions:

```
none
```

### 3.4.5. `BZ2_bzWriteOpen`

```
BZFILE *BZ2_bzWriteOpen( int *bzerror, FILE *f, 
                         int blockSize100k, int verbosity,
                         int workFactor );
```

Prepare to write compressed data to file handle
`f`.
`f` should refer to a file which
has been opened for writing, and for which the error indicator
(`ferror(f)`)is not set.

For the meaning of parameters
`blockSize100k`,
`verbosity` and
`workFactor`, see
`BZ2_bzCompressInit`.

All required memory is allocated at this stage, so if the
call completes successfully,
`BZ_MEM_ERROR` cannot be signalled
by a subsequent call to
`BZ2_bzWrite`.

Possible assignments to
`bzerror`:

```
BZ_CONFIG_ERROR
  if the library has been mis-compiled
BZ_PARAM_ERROR
  if f is NULL
  or blockSize100k < 1 or blockSize100k > 9
BZ_IO_ERROR
  if ferror(f) is nonzero
BZ_MEM_ERROR
  if insufficient memory is available
BZ_OK
  otherwise
```

Possible return values:

```
Pointer to an abstract BZFILE
  if bzerror is BZ_OK
NULL
  otherwise
```

Allowable next actions:

```
BZ2_bzWrite
  if bzerror is BZ_OK
  (you could go directly to BZ2_bzWriteClose, but this would be pretty pointless)
BZ2_bzWriteClose
  otherwise
```

### 3.4.6. `BZ2_bzWrite`

```
void BZ2_bzWrite ( int *bzerror, BZFILE *b, void *buf, int len );
```

Absorbs `len` bytes from the
buffer `buf`, eventually to be
compressed and written to the file.

Possible assignments to
`bzerror`:

```
BZ_PARAM_ERROR
  if b is NULL or buf is NULL or len < 0
BZ_SEQUENCE_ERROR
  if b was opened with BZ2_bzReadOpen
BZ_IO_ERROR
  if there is an error writing the compressed file.
BZ_OK
  otherwise
```

### 3.4.7. `BZ2_bzWriteClose`

```
void BZ2_bzWriteClose( int *bzerror, BZFILE* f,
                       int abandon,
                       unsigned int* nbytes_in,
                       unsigned int* nbytes_out );

void BZ2_bzWriteClose64( int *bzerror, BZFILE* f,
                         int abandon,
                         unsigned int* nbytes_in_lo32,
                         unsigned int* nbytes_in_hi32,
                         unsigned int* nbytes_out_lo32,
                         unsigned int* nbytes_out_hi32 );
```

Compresses and flushes to the compressed file all data so
far supplied by `BZ2_bzWrite`.
The logical end-of-stream markers are also written, so subsequent
calls to `BZ2_bzWrite` are
illegal. All memory associated with the compressed file
`b` is released.
`fflush` is called on the
compressed file, but it is not
`fclose`'d.

If `BZ2_bzWriteClose` is
called to clean up after an error, the only action is to release
the memory. The library records the error codes issued by
previous calls, so this situation will be detected automatically.
There is no attempt to complete the compression operation, nor to
`fflush` the compressed file. You
can force this behaviour to happen even in the case of no error,
by passing a nonzero value to
`abandon`.

If `nbytes_in` is non-null,
`*nbytes_in` will be set to be the
total volume of uncompressed data handled. Similarly,
`nbytes_out` will be set to the
total volume of compressed data written. For compatibility with
older versions of the library,
`BZ2_bzWriteClose` only yields the
lower 32 bits of these counts. Use
`BZ2_bzWriteClose64` if you want
the full 64 bit counts. These two functions are otherwise
absolutely identical.

Possible assignments to
`bzerror`:

```
BZ_SEQUENCE_ERROR
  if b was opened with BZ2_bzReadOpen
BZ_IO_ERROR
  if there is an error writing the compressed file
BZ_OK
  otherwise
```

### 3.4.8. Handling embedded compressed data streams

The high-level library facilitates use of
`bzip2` data streams which form
some part of a surrounding, larger data stream.

- For writing, the library takes an open file handle,
  writes compressed data to it,
  `fflush`es it but does not
  `fclose` it. The calling
  application can write its own data before and after the
  compressed data stream, using that same file handle.
- Reading is more complex, and the facilities are not as
  general as they could be since generality is hard to reconcile
  with efficiency. `BZ2_bzRead`
  reads from the compressed file in blocks of size
  `BZ_MAX_UNUSED` bytes, and in
  doing so probably will overshoot the logical end of compressed
  stream. To recover this data once decompression has ended,
  call `BZ2_bzReadGetUnused` after
  the last call of `BZ2_bzRead`
  (the one returning
  `BZ_STREAM_END`) but before
  calling
  `BZ2_bzReadClose`.

This mechanism makes it easy to decompress multiple
`bzip2` streams placed end-to-end.
As the end of one stream, when
`BZ2_bzRead` returns
`BZ_STREAM_END`, call
`BZ2_bzReadGetUnused` to collect
the unused data (copy it into your own buffer somewhere). That
data forms the start of the next compressed stream. To start
uncompressing that next stream, call
`BZ2_bzReadOpen` again, feeding in
the unused data via the `unused` /
`nUnused` parameters. Keep doing
this until `BZ_STREAM_END` return
coincides with the physical end of file
(`feof(f)`). In this situation
`BZ2_bzReadGetUnused` will of
course return no data.

This should give some feel for how the high-level interface
can be used. If you require extra flexibility, you'll have to
bite the bullet and get to grips with the low-level
interface.

### 3.4.9. Standard file-reading/writing code

Here's how you'd write data to a compressed file:

```
FILE*   f;
BZFILE* b;
int     nBuf;
char    buf[ /* whatever size you like */ ];
int     bzerror;
int     nWritten;

f = fopen ( "myfile.bz2", "w" );
if ( !f ) {
 /* handle error */
}
b = BZ2_bzWriteOpen( &bzerror, f, 9 );
if (bzerror != BZ_OK) {
 BZ2_bzWriteClose ( b );
 /* handle error */
}

while ( /* condition */ ) {
 /* get data to write into buf, and set nBuf appropriately */
 nWritten = BZ2_bzWrite ( &bzerror, b, buf, nBuf );
 if (bzerror == BZ_IO_ERROR) { 
   BZ2_bzWriteClose ( &bzerror, b );
   /* handle error */
 }
}

BZ2_bzWriteClose( &bzerror, b );
if (bzerror == BZ_IO_ERROR) {
 /* handle error */
}
```

And to read from a compressed file:

```
FILE*   f;
BZFILE* b;
int     nBuf;
char    buf[ /* whatever size you like */ ];
int     bzerror;
int     nWritten;

f = fopen ( "myfile.bz2", "r" );
if ( !f ) {
  /* handle error */
}
b = BZ2_bzReadOpen ( &bzerror, f, 0, NULL, 0 );
if ( bzerror != BZ_OK ) {
  BZ2_bzReadClose ( &bzerror, b );
  /* handle error */
}

bzerror = BZ_OK;
while ( bzerror == BZ_OK && /* arbitrary other conditions */) {
  nBuf = BZ2_bzRead ( &bzerror, b, buf, /* size of buf */ );
  if ( bzerror == BZ_OK ) {
    /* do something with buf[0 .. nBuf-1] */
  }
}
if ( bzerror != BZ_STREAM_END ) {
   BZ2_bzReadClose ( &bzerror, b );
   /* handle error */
} else {
   BZ2_bzReadClose ( &bzerror, b );
}
```

## 3.5. Utility functions

### 3.5.1. `BZ2_bzBuffToBuffCompress`

```
int BZ2_bzBuffToBuffCompress( char*         dest,
                              unsigned int* destLen,
                              char*         source,
                              unsigned int  sourceLen,
                              int           blockSize100k,
                              int           verbosity,
                              int           workFactor );
```

Attempts to compress the data in `source[0
.. sourceLen-1]` into the destination buffer,
`dest[0 .. *destLen-1]`. If the
destination buffer is big enough,
`*destLen` is set to the size of
the compressed data, and `BZ_OK`
is returned. If the compressed data won't fit,
`*destLen` is unchanged, and
`BZ_OUTBUFF_FULL` is
returned.

Compression in this manner is a one-shot event, done with a
single call to this function. The resulting compressed data is a
complete `bzip2` format data
stream. There is no mechanism for making additional calls to
provide extra input data. If you want that kind of mechanism,
use the low-level interface.

For the meaning of parameters
`blockSize100k`,
`verbosity` and
`workFactor`, see
`BZ2_bzCompressInit`.

To guarantee that the compressed data will fit in its
buffer, allocate an output buffer of size 1% larger than the
uncompressed data, plus six hundred extra bytes.

`BZ2_bzBuffToBuffDecompress`
will not write data at or beyond
`dest[*destLen]`, even in case of
buffer overflow.

Possible return values:

```
BZ_CONFIG_ERROR
  if the library has been mis-compiled
BZ_PARAM_ERROR
  if dest is NULL or destLen is NULL
  or blockSize100k < 1 or blockSize100k > 9
  or verbosity < 0 or verbosity > 4
  or workFactor < 0 or workFactor > 250
BZ_MEM_ERROR
  if insufficient memory is available 
BZ_OUTBUFF_FULL
  if the size of the compressed data exceeds *destLen
BZ_OK
  otherwise
```

### 3.5.2. `BZ2_bzBuffToBuffDecompress`

```
int BZ2_bzBuffToBuffDecompress( char*         dest,
                                unsigned int* destLen,
                                char*         source,
                                unsigned int  sourceLen,
                                int           small,
                                int           verbosity );
```

Attempts to decompress the data in `source[0
.. sourceLen-1]` into the destination buffer,
`dest[0 .. *destLen-1]`. If the
destination buffer is big enough,
`*destLen` is set to the size of
the uncompressed data, and `BZ_OK`
is returned. If the compressed data won't fit,
`*destLen` is unchanged, and
`BZ_OUTBUFF_FULL` is
returned.

`source` is assumed to hold
a complete `bzip2` format data
stream.
`BZ2_bzBuffToBuffDecompress` tries
to decompress the entirety of the stream into the output
buffer.

For the meaning of parameters
`small` and
`verbosity`, see
`BZ2_bzDecompressInit`.

Because the compression ratio of the compressed data cannot
be known in advance, there is no easy way to guarantee that the
output buffer will be big enough. You may of course make
arrangements in your code to record the size of the uncompressed
data, but such a mechanism is beyond the scope of this
library.

`BZ2_bzBuffToBuffDecompress`
will not write data at or beyond
`dest[*destLen]`, even in case of
buffer overflow.

Possible return values:

```
BZ_CONFIG_ERROR
  if the library has been mis-compiled
BZ_PARAM_ERROR
  if dest is NULL or destLen is NULL
  or small != 0 && small != 1
  or verbosity < 0 or verbosity > 4
BZ_MEM_ERROR
  if insufficient memory is available 
BZ_OUTBUFF_FULL
  if the size of the compressed data exceeds *destLen
BZ_DATA_ERROR
  if a data integrity error was detected in the compressed data
BZ_DATA_ERROR_MAGIC
  if the compressed data doesn't begin with the right magic bytes
BZ_UNEXPECTED_EOF
  if the compressed data ends unexpectedly
BZ_OK
  otherwise
```

## 3.6. `zlib` compatibility functions

Yoshioka Tsuneo has contributed some functions to give
better `zlib` compatibility.
These functions are `BZ2_bzopen`,
`BZ2_bzread`,
`BZ2_bzwrite`,
`BZ2_bzflush`,
`BZ2_bzclose`,
`BZ2_bzerror` and
`BZ2_bzlibVersion`. These
functions are not (yet) officially part of the library. If they
break, you get to keep all the pieces. Nevertheless, I think
they work ok.

```
typedef void BZFILE;

const char * BZ2_bzlibVersion ( void );
```

Returns a string indicating the library version.

```
BZFILE * BZ2_bzopen  ( const char *path, const char *mode );
BZFILE * BZ2_bzdopen ( int        fd,    const char *mode );
```

Opens a `.bz2` file for
reading or writing, using either its name or a pre-existing file
descriptor. Analogous to `fopen`
and `fdopen`.

```
int BZ2_bzread  ( BZFILE* b, void* buf, int len );
int BZ2_bzwrite ( BZFILE* b, void* buf, int len );
```

Reads/writes data from/to a previously opened
`BZFILE`. Analogous to
`fread` and
`fwrite`.

```
int  BZ2_bzflush ( BZFILE* b );
void BZ2_bzclose ( BZFILE* b );
```

Flushes/closes a `BZFILE`.
`BZ2_bzflush` doesn't actually do
anything. Analogous to `fflush`
and `fclose`.

```
const char * BZ2_bzerror ( BZFILE *b, int *errnum )
```

Returns a string describing the more recent error status of
`b`, and also sets
`*errnum` to its numerical
value.

## 3.7. Using the library in a `stdio`-free environment

### 3.7.1. Getting rid of `stdio`

In a deeply embedded application, you might want to use
just the memory-to-memory functions. You can do this
conveniently by compiling the library with preprocessor symbol
`BZ_NO_STDIO` defined. Doing this
gives you a library containing only the following eight
functions:

`BZ2_bzCompressInit`,
`BZ2_bzCompress`,
`BZ2_bzCompressEnd`
`BZ2_bzDecompressInit`,
`BZ2_bzDecompress`,
`BZ2_bzDecompressEnd`
`BZ2_bzBuffToBuffCompress`,
`BZ2_bzBuffToBuffDecompress`

When compiled like this, all functions will ignore
`verbosity` settings.

### 3.7.2. Critical error handling

`libbzip2` contains a number
of internal assertion checks which should, needless to say, never
be activated. Nevertheless, if an assertion should fail,
behaviour depends on whether or not the library was compiled with
`BZ_NO_STDIO` set.

For a normal compile, an assertion failure yields the
message:

> bzip2/libbzip2: internal error number N.
>
> This is a bug in bzip2/libbzip2, 1.0.5 of 10 December 2007.
> Please report it to me at: jseward@bzip.org. If this happened
> when you were using some program which uses libbzip2 as a
> component, you should also report this bug to the author(s)
> of that program. Please make an effort to report this bug;
> timely and accurate bug reports eventually lead to higher
> quality software. Thanks. Julian Seward, 10 December 2007.

where `N` is some error code
number. If `N == 1007`, it also
prints some extra text advising the reader that unreliable memory
is often associated with internal error 1007. (This is a
frequently-observed-phenomenon with versions 1.0.0/1.0.1).

`exit(3)` is then
called.

For a `stdio`-free library,
assertion failures result in a call to a function declared
as:

```
extern void bz_internal_error ( int errcode );
```

The relevant code is passed as a parameter. You should
supply such a function.

In either case, once an assertion failure has occurred, any
`bz_stream` records involved can
be regarded as invalid. You should not attempt to resume normal
operation with them.

You may, of course, change critical error handling to suit
your needs. As I said above, critical errors indicate bugs in
the library and should not occur. All "normal" error situations
are indicated via error return codes from functions, and can be
recovered from.

## 3.8. Making a Windows DLL

Everything related to Windows has been contributed by
Yoshioka Tsuneo
(`tsuneo@rr.iij4u.or.jp`), so
you should send your queries to him (but perhaps Cc: me,
`jseward@bzip.org`).

My vague understanding of what to do is: using Visual C++
5.0, open the project file
`libbz2.dsp`, and build. That's
all.

If you can't open the project file for some reason, make a
new one, naming these files:
`blocksort.c`,
`bzlib.c`,
`compress.c`,
`crctable.c`,
`decompress.c`,
`huffman.c`,
`randtable.c` and
`libbz2.def`. You will also need
to name the header files `bzlib.h`
and `bzlib_private.h`.

If you don't use VC++, you may need to define the
proprocessor symbol
`_WIN32`.

Finally, `dlltest.c` is a
sample program using the DLL. It has a project file,
`dlltest.dsp`.

If you just want a makefile for Visual C, have a look at
`makefile.msc`.

Be aware that if you compile
`bzip2` itself on Win32, you must
set `BZ_UNIX` to 0 and
`BZ_LCCWIN32` to 1, in the file
`bzip2.c`, before compiling.
Otherwise the resulting binary won't work correctly.

I haven't tried any of this stuff myself, but it all looks
plausible.

## 4. Miscellanea

**Table of Contents**

4.1. Limitations of the compressed file format

4.2. Portability issues

4.3. Reporting bugs

4.4. Did you get the right package?

4.5. Further Reading

These are just some random thoughts of mine. Your mileage
may vary.

## 4.1. Limitations of the compressed file format

`bzip2-1.0.X`,
`0.9.5` and
`0.9.0` use exactly the same file
format as the original version,
`bzip2-0.1`. This decision was
made in the interests of stability. Creating yet another
incompatible compressed file format would create further
confusion and disruption for users.

Nevertheless, this is not a painless decision. Development
work since the release of
`bzip2-0.1` in August 1997 has
shown complexities in the file format which slow down
decompression and, in retrospect, are unnecessary. These
are:

- The run-length encoder, which is the first of the
  compression transformations, is entirely irrelevant. The
  original purpose was to protect the sorting algorithm from the
  very worst case input: a string of repeated symbols. But
  algorithm steps Q6a and Q6b in the original Burrows-Wheeler
  technical report (SRC-124) show how repeats can be handled
  without difficulty in block sorting.
- The randomisation mechanism doesn't really need to be
  there. Udi Manber and Gene Myers published a suffix array
  construction algorithm a few years back, which can be employed
  to sort any block, no matter how repetitive, in O(N log N)
  time. Subsequent work by Kunihiko Sadakane has produced a
  derivative O(N (log N)^2) algorithm which usually outperforms
  the Manber-Myers algorithm.

  I could have changed to Sadakane's algorithm, but I find
  it to be slower than `bzip2`'s
  existing algorithm for most inputs, and the randomisation
  mechanism protects adequately against bad cases. I didn't
  think it was a good tradeoff to make. Partly this is due to
  the fact that I was not flooded with email complaints about
  `bzip2-0.1`'s performance on
  repetitive data, so perhaps it isn't a problem for real
  inputs.

  Probably the best long-term solution, and the one I have
  incorporated into 0.9.5 and above, is to use the existing
  sorting algorithm initially, and fall back to a O(N (log N)^2)
  algorithm if the standard algorithm gets into
  difficulties.
- The compressed file format was never designed to be
  handled by a library, and I have had to jump though some hoops
  to produce an efficient implementation of decompression. It's
  a bit hairy. Try passing
  `decompress.c` through the C
  preprocessor and you'll see what I mean. Much of this
  complexity could have been avoided if the compressed size of
  each block of data was recorded in the data stream.
- An Adler-32 checksum, rather than a CRC32 checksum,
  would be faster to compute.

It would be fair to say that the
`bzip2` format was frozen before I
properly and fully understood the performance consequences of
doing so.

Improvements which I was able to incorporate into 0.9.0,
despite using the same file format, are:

- Single array implementation of the inverse BWT. This
  significantly speeds up decompression, presumably because it
  reduces the number of cache misses.
- Faster inverse MTF transform for large MTF values.
  The new implementation is based on the notion of sliding blocks
  of values.
- `bzip2-0.9.0` now reads
  and writes files with `fread`
  and `fwrite`; version 0.1 used
  `putc` and
  `getc`. Duh! Well, you live
  and learn.

Further ahead, it would be nice to be able to do random
access into files. This will require some careful design of
compressed file formats.

## 4.2. Portability issues

After some consideration, I have decided not to use GNU
`autoconf` to configure 0.9.5 or
1.0.

`autoconf`, admirable and
wonderful though it is, mainly assists with portability problems
between Unix-like platforms. But
`bzip2` doesn't have much in the
way of portability problems on Unix; most of the difficulties
appear when porting to the Mac, or to Microsoft's operating
systems. `autoconf` doesn't help
in those cases, and brings in a whole load of new
complexity.

Most people should be able to compile the library and
program under Unix straight out-of-the-box, so to speak,
especially if you have a version of GNU C available.

There are a couple of
`__inline__` directives in the
code. GNU C (`gcc`) should be
able to handle them. If you're not using GNU C, your C compiler
shouldn't see them at all. If your compiler does, for some
reason, see them and doesn't like them, just
`#define`
`__inline__` to be
`/* */`. One easy way to do this
is to compile with the flag
`-D__inline__=`, which should be
understood by most Unix compilers.

If you still have difficulties, try compiling with the
macro `BZ_STRICT_ANSI` defined.
This should enable you to build the library in a strictly ANSI
compliant environment. Building the program itself like this is
dangerous and not supported, since you remove
`bzip2`'s checks against
compressing directories, symbolic links, devices, and other
not-really-a-file entities. This could cause filesystem
corruption!

One other thing: if you create a
`bzip2` binary for public distribution,
please consider linking it statically (`gcc
-static`). This avoids all sorts of library-version
issues that others may encounter later on.

If you build `bzip2` on
Win32, you must set `BZ_UNIX` to 0
and `BZ_LCCWIN32` to 1, in the
file `bzip2.c`, before compiling.
Otherwise the resulting binary won't work correctly.

## 4.3. Reporting bugs

I tried pretty hard to make sure
`bzip2` is bug free, both by
design and by testing. Hopefully you'll never need to read this
section for real.

Nevertheless, if `bzip2` dies
with a segmentation fault, a bus error or an internal assertion
failure, it will ask you to email me a bug report. Experience from
years of feedback of bzip2 users indicates that almost all these
problems can be traced to either compiler bugs or hardware
problems.

- Recompile the program with no optimisation, and
  see if it works. And/or try a different compiler. I heard all
  sorts of stories about various flavours of GNU C (and other
  compilers) generating bad code for
  `bzip2`, and I've run across two
  such examples myself.

  2.7.X versions of GNU C are known to generate bad code
  from time to time, at high optimisation levels. If you get
  problems, try using the flags
  `-O2`
  `-fomit-frame-pointer`
  `-fno-strength-reduce`. You
  should specifically *not* use
  `-funroll-loops`.

  You may notice that the Makefile runs six tests as part
  of the build process. If the program passes all of these, it's
  a pretty good (but not 100%) indication that the compiler has
  done its job correctly.
- If `bzip2`
  crashes randomly, and the crashes are not repeatable, you may
  have a flaky memory subsystem.
  `bzip2` really hammers your
  memory hierarchy, and if it's a bit marginal, you may get these
  problems. Ditto if your disk or I/O subsystem is slowly
  failing. Yup, this really does happen.

  Try using a different machine of the same type, and see
  if you can repeat the problem.
- This isn't really a bug, but ... If
  `bzip2` tells you your file is
  corrupted on decompression, and you obtained the file via FTP,
  there is a possibility that you forgot to tell FTP to do a
  binary mode transfer. That absolutely will cause the file to
  be non-decompressible. You'll have to transfer it
  again.

If you've incorporated
`libbzip2` into your own program
and are getting problems, please, please, please, check that the
parameters you are passing in calls to the library, are correct,
and in accordance with what the documentation says is allowable.
I have tried to make the library robust against such problems,
but I'm sure I haven't succeeded.

Finally, if the above comments don't help, you'll have to
send me a bug report. Now, it's just amazing how many people
will send me a bug report saying something like:

```
bzip2 crashed with segmentation fault on my machine
```

and absolutely nothing else. Needless to say, a such a
report is *totally, utterly, completely and
comprehensively 100% useless; a waste of your time, my time, and
net bandwidth*. With no details at all, there's no way
I can possibly begin to figure out what the problem is.

The rules of the game are: facts, facts, facts. Don't omit
them because "oh, they won't be relevant". At the bare
minimum:

```
Machine type.  Operating system version.  
Exact version of bzip2 (do bzip2 -V).  
Exact version of the compiler used.  
Flags passed to the compiler.
```

However, the most important single thing that will help me
is the file that you were trying to compress or decompress at the
time the problem happened. Without that, my ability to do
anything more than speculate about the cause, is limited.

## 4.4. Did you get the right package?

`bzip2` is a resource hog.
It soaks up large amounts of CPU cycles and memory. Also, it
gives very large latencies. In the worst case, you can feed many
megabytes of uncompressed data into the library before getting
any compressed output, so this probably rules out applications
requiring interactive behaviour.

These aren't faults of my implementation, I hope, but more
an intrinsic property of the Burrows-Wheeler transform
(unfortunately). Maybe this isn't what you want.

If you want a compressor and/or library which is faster,
uses less memory but gets pretty good compression, and has
minimal latency, consider Jean-loup Gailly's and Mark Adler's
work, `zlib-1.2.1` and
`gzip-1.2.4`. Look for them at
http://www.zlib.org and
http://www.gzip.org
respectively.

For something faster and lighter still, you might try Markus F
X J Oberhumer's `LZO` real-time
compression/decompression library, at
http://www.oberhumer.com/opensource.

## 4.5. Further Reading

`bzip2` is not research
work, in the sense that it doesn't present any new ideas.
Rather, it's an engineering exercise based on existing
ideas.

Four documents describe essentially all the ideas behind
`bzip2`:

Michael Burrows and D. J. Wheeler:  
  "A block-sorting lossless data compression algorithm"  
   10th May 1994.   
   Digital SRC Research Report 124.  
   ftp://ftp.digital.com/pub/DEC/SRC/research-reports/SRC-124.ps.gz  
   If you have trouble finding it, try searching at the  
   New Zealand Digital Library, http://www.nzdl.org.  
  
Daniel S. Hirschberg and Debra A. LeLewer  
  "Efficient Decoding of Prefix Codes"  
   Communications of the ACM, April 1990, Vol 33, Number 4.  
   You might be able to get an electronic copy of this  
   from the ACM Digital Library.  
  
David J. Wheeler  
   Program bred3.c and accompanying document bred3.ps.  
   This contains the idea behind the multi-table Huffman coding scheme.  
   ftp://ftp.cl.cam.ac.uk/users/djw3/  
  
Jon L. Bentley and Robert Sedgewick  
  "Fast Algorithms for Sorting and Searching Strings"  
   Available from Sedgewick's web page,  
   www.cs.princeton.edu/~rs

The following paper gives valuable additional insights into
the algorithm, but is not immediately the basis of any code used
in bzip2.

Peter Fenwick:  
   Block Sorting Text Compression  
   Proceedings of the 19th Australasian Computer Science Conference,  
     Melbourne, Australia.  Jan 31 - Feb 2, 1996.  
   ftp://ftp.cs.auckland.ac.nz/pub/peter-f/ACSC96paper.ps

Kunihiko Sadakane's sorting algorithm, mentioned above, is
available from:

http://naomi.is.s.u-tokyo.ac.jp/~sada/papers/Sada98b.ps.gz

The Manber-Myers suffix array construction algorithm is
described in a paper available from:

http://www.cs.arizona.edu/people/gene/PAPERS/suffix.ps

Finally, the following papers document some
investigations I made into the performance of sorting
and decompression algorithms:

Julian Seward  
   On the Performance of BWT Sorting Algorithms  
   Proceedings of the IEEE Data Compression Conference 2000  
     Snowbird, Utah.  28-30 March 2000.  
  
Julian Seward  
   Space-time Tradeoffs in the Inverse B-W Transform  
   Proceedings of the IEEE Data Compression Conference 2001  
     Snowbird, Utah.  27-29 March 2001.
