# Supplementary material for: Evaluation of de novo transcriptome assemblies from RNA-Seq data
Source: Genome Biol. 2014 Dec 21;15(12):553. doi: 10.1186/s13059-014-0553-5 (PMC4298084; doi:10.1186/s13059-014-0553-5)
Supplement: Additional file 3 — Version of the DETONATE source code used for the experiments in this paper. [file 13059_2014_553_MOESM3_ESM.zip › 13059_2014_553_MOESM3_ESM/detonate-1.8.1/ref-eval/cmake/cmake-2.8.12.1.tar.gz!/cmake-2.8.12.1/Utilities/cmbzip2/manual.pdf]

bzip2 and libbzip2, version 1.0.5: A program and library for data compression

by Julian Seward

Copyright ©-489(data)]TJ0-.4lE9dompressi85799eTess3

Table of Contents

than a block. For example, compressing a file 20,000 bytes long with the flag

worst-case and average-case compression time is in the region of 10:1. For previous versions, this figure was more like 100:1. You can use the `-vvvv` option to monitor progress in great detail, if you want.

BZ_MEM_ERROR

Returned when a request to allocate memory failed. Note that the quantity of memory needed to decompress

2. Shovel data in and shlurp out its compressed form using zero or more calls of

- If `bzerror` indicates an error (ie, anything except

```
Pointer to an abstract BZFILE
  if bzerror is BZ_OK
NULL
  otherwise
```

Allowable next actions:

```
BZ2_bzRead
  if bzerror is BZ_OK
BZ2_bzClose
  otherwise
```

For the meaning of parameters blockSize100k, verbosity

- Recompile the program with no optimisation, and see if it works. And/or try a different compiler. I heard all sorts of stories about various flavours of GNU C (and other compilers) generating bad code for `bzip2`, and I've run across two such examples myself.

  2.7.X versions of GNU C are known to generate bad code from time to time, at high optimisation levels. If you get problems, try using the flags `-O2 -fomit-frame-pointer -fno-strength-reduce`. You should specifically *not*
