# Supplementary material for: Evaluation of de novo transcriptome assemblies from RNA-Seq data
Source: Genome Biol. 2014 Dec 21;15(12):553. doi: 10.1186/s13059-014-0553-5 (PMC4298084; doi:10.1186/s13059-014-0553-5)
Supplement: Additional file 3 — Version of the DETONATE source code used for the experiments in this paper. [file 13059_2014_553_MOESM3_ESM.zip › 13059_2014_553_MOESM3_ESM/detonate-1.8.1/ref-eval/ref-eval.template.html]

xml version="1.0"?


REF-EVAL: A toolkit of reference-based scores for de novo
transcriptome sequence assembly evaluation


# REF-EVAL: A toolkit of reference-based scores for *de novo* transcriptome sequence assembly evaluation

## Overview

REF-EVAL computes a number of reference-based scores. These scores measure
the quality of a transcriptome assembly relative to a collection of reference
sequences. For information about how to run REF-EVAL, see ``Usage'' and the
sections following it below. For information about the score definitions, see
``Score definitions'' and the sections following it below.

## Usage

As an optional first step, estimate the ``true'' assembly, using REF-EVAL-ESTIMATE-TRUE-ASSEMBLY.
Alternatively, you can use the full-length reference transcript sequences
directly as a reference.

From now on, we will call the estimated ``true'' assembly or the collection
of full-length reference sequences (whichever you choose to use) the reference.
Let's assume that the assembly of interest is in A.fa, and the
reference is in B.fa.

If you want to compute alignment-based scores (see --scores below
for more info), align the assembly to the reference and vice versa using Blat. We recommend fairly
unrestrictive settings, in order to generate many candidate alignments.

```
$ blat -minIdentity=80 B.fa A.fa A_to_B.psl
$ blat -minIdentity=80 A.fa B.fa B_to_A.psl
```

If you want to compute weighted variants of scores, use RSEM (or RSEM-EVAL)
to compute the expression of the assembly and reference relative to the given
reads. Let's assume that the reads are in reads.fq.

```
$ rsem-prepare-reference --no-polyA A.fa A_ref
$ rsem-prepare-reference --no-polyA B.fa B_ref
$ rsem-calculate-expression -p 24 --no-bam-output reads.fq A_ref A_expr
$ rsem-calculate-expression -p 24 --no-bam-output reads.fq B_ref B_expr
```

Finally, run REF-EVAL. To compute everything, run:

```
$ ./ref-eval --scores=nucl,pair,contig,kmer,kc \
             --weighted=both \
             --A-seqs A.fa \
             --B-seqs B.fa \
             --A-expr A_expr.isoforms.results \
             --B-expr B_expr.isoforms.results \
             --A-to-B A_to_B.psl \
             --B-to-A B_to_A.psl \
             --num-reads 5000000 \
             --readlen 76 \
             --kmerlen 76 \
             | tee scores.txt
```

To only compute the kmer compression score (and its dependencies), run:

```
$ ./ref-eval --scores=kc \
             --A-seqs A.fa \
             --B-seqs B.fa \
             --B-expr B_expr.isoforms.results \
             --num-reads 5000000 \
             --readlen 76 \
             --kmerlen 76 \
             | tee scores.txt
```

To only compute the unweighted reference-based scores, run:

```
$ ./ref-eval --scores=nucl,pair,contig \
             --weighted=no \
             --A-seqs A.fa \
             --B-seqs B.fa \
             --A-to-B A_to_B.psl \
             --B-to-A B_to_A.psl \
             | tee scores.txt
```

To only compute the scores discussed in the DETONATE paper, run:

```
$ ./ref-eval --paper \
             --A-seqs A.fa \
             --B-seqs B.fa \
             --B-expr B_expr.isoforms.results \
             --A-to-B A_to_B.psl \
             --B-to-A B_to_A.psl \
             --num-reads 5000000 \
             --readlen 76 \
             --kmerlen 76 \
             | tee scores.txt
```

The scores will be written to standard output (hence, above, to scores.txt).
Progress information is written to standard error. Further details about the
arguments to REF-EVAL are described below. Further details about the scores
themselves are given under ``Score definitions'' below.

## Usage: Score specification

--scores arg
:   The groups of scores to compute, separated by commas (e.g.,
    --scores=nucl,contig,kc). It is more efficient to compute all
    the scores you are interested in using one invocation of REF-EVAL
    instead of using multiple invocations that each compute one score. The
    available score groups are as follows:

    Alignment-based score groups:

    - nucl: nucleotide precision, recall, and F1.
    - contig: contig precision, recall, and F1.
    - pair: pair precision, recall, and F1.

    Alignment-free score groups:

    - kmer: kmer Kullback-Leibler divergence, Jensen-Shannon
      divergence, and Hellinger distance.
    - kc: kmer recall, number of nucleotides, and kmer
      compression score.

    Required unless --paper is given.

--weighted arg
:   A string indicating whether to compute weighted or unweighted
    variants of scores, or both (e.g., --weighted=yes):

    - yes: compute weighted variants of scores.
    - no: compute unweighted variants of scores.
    - both: compute both weighted and unweighted variants of scores.

    In weighted variants, the expression levels (TPM) of the assembly
    and reference sequences are taken into account, and hence need to be
    specified using --A-expr and --B-expr. Unweighted
    variants are equivalent to weighted variants with uniform
    expression.

    The distinction between weighted and unweighted variants doesn't
    make sense for the KC score, so this option is ignored by the KC score.

    Required unless --paper or only --score=kc is
    given.

--paper
:   As an alternative to the above, if you are only interested in
    computing the scores described in the main text of our paper [1], you
    can pass the --paper flag instead of the --scores and
    --weighted options. In that case, the following scores will be
    computed:

    Alignment-based scores:

    - unweighted nucleotide F1
    - unweighted contig F1

    Alignment-free score groups:

    - weighted kmer compression score

    For obvious reasons, the --scores and --weighted
    options are incompatible with this flag.

    [1] Bo Li\*, Nathanael Fillmore\*, Yongsheng Bai, Mike Collins, James A.
    Thompson, Ron Stewart, Colin N. Dewey. Evaluation of *de novo*
    transcriptome assemblies from RNA-Seq data.

## Usage: Input and output specification

--A-seqs arg
:   The assembly sequences, in FASTA format. Required.

--B-seqs arg
:   The reference sequences, in FASTA format. Required.

--A-expr arg
:   The assembly expression, for use in weighted scores, as produced by
    RSEM in a file called \*.isoforms.results. Required for
    weighted variants of scores.

--B-expr arg
:   The reference expression, for use in weighted scores, as produced by
    RSEM in a file called \*.isoforms.results. Required for
    weighted variants of scores.

--A-to-B arg
:   The alignments of the assembly to the reference. The file format is
    specified by --alignment-type. Required for alignment-based
    scores.

--B-to-A arg
:   The alignments of the reference to the assembly. The file format is
    specified by --alignment-type. Required for alignment-based
    scores.

--alignment-type arg
:   The type of alignments used, either blast or psl.
    Default: psl. Currently BLAST support is experimental, not
    well tested, and not recommended.

## Usage: Options that modify the score definitions (and hence output)

--strand-specific
:   If this flag is present, it is assumed that all the assembly and
    reference sequences have the same orientation. Thus, alignments or kmer
    matches that are to the reverse strand are ignored.

--readlen arg
:   This option only applies to the KC scores. The read length of the
    reads used to build the assembly, used in the denominator of the ICR.
    Required for KC scores.

--num-reads arg
:   This option only applies to the KC scores. The number of reads used
    to build the assembly, used in the denominator of the ICR. Required for
    KC scores.

--kmerlen arg
:   This option only applies to the kmer and KC scores. This is the
    length (``k'') of the kmers used in the definition of the KC and kmer
    scores. Required for KC and kmer scores.

--min-frac-identity arg
:   This option only applies to contig scores. Alignments with fraction
    identity less than this threshold are ignored. The fraction identity
    of an alignment is min(x/y, x/z), where

    - $x$ is the number of bases in the assembly sequence that are
      aligned to an identical base in the reference sequence,
      according to the alignment,
    - $y$ is the number of bases in the assembly sequence, and
    - $z$ is the number of bases in the reference sequence.

    Default: 0.99.

--max-frac-indel arg
:   This option only applies to contig scores. Alignments with fraction
    indel greater than this threshold are ignored. For psl alignments, the
    fraction indel of an alignment is $\max(w/y, x/z)$, where

    - $w$ is the number of bases that are inserted in the assembly
      sequence, according to the alignment (``Q gap bases''),
    - $x$ is the number of bases that are inserted in the reference
      sequence, according to the alignment (``T gap bases''),
    - $y$ is the number of bases in the assembly sequence, and
    - $z$ is the number of bases in the reference sequence.

    For blast alignments, the fraction indel of an alignment is
    $\max(x/y, x/z)$, where

    - $x$ is the number of gaps bases that are inserted in the
      reference sequence, according to the alignment (``gaps''),
    - $y$ is the number of bases in the assembly sequence, and
    - $z$ is the number of bases in the reference sequence.

    Default: 0.01.

--min-segment-len arg
:   This option only applies to nucleotide and pair scores. Alignment
    segments that contain fewer than this number of bases will be
    discarded. Default: 100. In the DETONATE paper, this was set to the
    read length.

## Usage: Options that modify the algorithm, but not the score definitions

--hash-table-type arg
:   The type of hash table to use, either ``sparse'' or ``dense''. This
    is only relevant for KC and kmer scores. The sparse table is slower but
    uses less memory. The dense table is faster but uses more memory.
    Default: ``sparse''.

--hash-table-fudge-factor arg
:   This is only relevant for KC and kmer scores. When the hash table is
    created, its initial capacity is set as the total worst-case number of
    possible kmers in the assembly and reference, based on each sequence's
    length, divided by the fudge factor. The default, 2.0, is often
    reasonable because (1) most kmers should be shared by the assembly and
    the reference, and (2) many kmers will be repeated several times.
    However, if you have a lot of memory or a really bad assembly, you
    could try a smaller number. Default: 2.0.

## Usage: Options to include additional output

--trace arg
:   If given, the prefix for additional output that provides details
    about the REF-EVAL scores; if not given, no such output is produced.
    Currently, the only such output is as follows.

    - (--trace).{weighted,unweighted}\_contig\_{precision,recall}\_matching
      is a TSV file that describes the matching used to compute the
      weighted or unweighted contig precision or recall. (Details about
      the matching are given in the section on score definitions below.)
      For recall, each row corresponds to a reference sequence $b$.
      Column 1 contains $b$'s name. If $b$ is matched to a contig $a$,
      then the remaining columns are as follows:
      - Column 2 contains $a$'s name.
      - Column 3 contains the weight of the edge between $b$ and
        $a$. (This is set to the uniform weights $1/|B|$ in the
        unweighted case, although the maximum cardinality matching
        algorithm does not actually use these weights.)
      - Column 4 contains the names of all the contigs $a'$ that
        are adjacent to $b$ in the bipartite graph that the
        matching is based on, separated by commas. Thus, this
        column lists all the contigs $a'$ that have a "good enough"
        match with the reference sequence $b$, according to the
        criteria used to build the bipartite graph. (See the
        section below on score definitions for details.)
      Otherwise, if $b$ is not matched to any contig, columns 2 and 3
      contain "NA". For precision, the file has the same format, but with
      the reference and the assembly interchanged. In other words, each
      row corresponds to a contig $a$ and contains information about its
      matching to a reference sequence $b$, or all "NA" values if $a$ was
      not matched.

## Usage: General options

-? [ --help ]
:   Display this information.

## Score definitions

In the next few sections, we define the scores computed by REF-EVAL.
Throughout, $A$ denotes the assembly, and $B$ denotes the reference. (As
discussed under ``Usage'' above, the reference can be either an estimate of the
``true'' assembly or a collection of full-length reference transcripts.) Both
$A$ and $B$ are thought of as sets of sequences. $A$ is a set of contigs, and
$B$ is a set of reference sequences.

## Score definitions: contig precision, recall, and F1

The contig recall is defined as follows:

- Align the assembly $A$ to the reference $B$. Notation: each alignment $l$
  is between a contig $a$ in $A$ and an reference sequence $b$ in $B$.
- Throw out alignments that are to the reverse strand, if
  --strand-specific is present.
- Throw out alignments whose fraction identity is less than
  --min-frac-identity (q.v.\ for the definition of ``fraction
  identity'').
- Throw out alignments whose fraction indel is greater than
  --max-frac-indel (q.v.\ for the definition of ``fraction indel'').
- Construct a bipartite graph from the remaining alignments, in which there
  is an edge between $a$ and $b$ iff there is a remaining alignment $l$ of
  $a$ to $b$.
- If --weighted=yes, specify a weight for each edge between $a$ and
  $b$, namely $\tau(b)$, the relative abundance of $b$ within the
  reference, as specified in --B-expr.
- The unweighted contig recall is the number of edges in the maximum
  cardinality matching of this graph, divided by the number of sequences in
  the reference $B$.
- The weighted contig recall is the weight of the maximum weight matching
  of this graph.

The contig precision is defined as follows: Interchange the assembly and the
reference, and compute the contig recall.

The contig F1 is the harmonic mean of the precision and recall.

## Score definitions: nucleotide precision, recall, and F1

The nucleotide recall is defined as follows:

- Align the assembly $A$ to the reference $B$. Notation: each alignment $l$
  is between a contig $a \in A$ and an reference element $b \in B$.
- Throw out alignments that are to the reverse strand, if
  --strand-specific is present.
- Throw out alignments that are shorter than --min-fragment-length.
- Add each remaining alignment to a priority queue, with priority equal to
  the number of identical bases in the alignment.
- Let numer = 0.
- While the priority queue is not empty:
  - Pop the alignment $l$ with highest priority.
  - Add the number of identical bases in the alignment to numer.
  - Subtract $l$ from all the other alignments in the queue and update their
    priorities (see below).
- Let denom be the total number of bases in the reference $B$.
- The unweighted nucleotide recall is numer/denom.

The actual implementation uses a more complicated and efficient algorithm
than the one above.

If --weighted=yes, then (i) ``the number of identical bases'' above
is replaced by ``the number of identical bases, times $\tau(b)$'', in the
definition of the priority and the numer, and (ii) ``total number of bases in
the reference $B$'' is replaced by ``$\sum\_{b \in B} \tau(b) length(b)$''. In
other words, each base (of a reference sequence), throughout the computation,
is weighted by the expression level of its parent sequence.

The nucleotide precision is defined as follows: Interchange the assembly and
the reference, and compute the nucleotide recall.

The nucleotide F1 is the harmonic mean of the precision and recall.

Alignment subtraction is defined as follows.

- An alignment $l$ from $a$ to $b$ can be thought of as a set of pairs of
  disjoint intervals

  $$
  \{ ([s\_1(a), e\_1(a)], [s\_1(b), e\_1(b)]), \dots, ([s\_n(a), e\_n(a)], [s\_n(b), e\_n(b)]) \},
  $$

  where each pair $([s\_i(a), e\_i(a)], [s\_i(b), e\_i(b)])$ corresponds to an
  ungapped segment of the alignment: $s\_i(a)$ and $e\_i(a)$ are the
  segment's start and end positions within a, and $s\_i(b)$ and $e\_i(b)$ are
  the segment's start and end positions within b. In the case of
  non-strand-specific alignments, $s\_i(b)$ might be greater than $e\_i(b)$.
- If $l$ is an alignment from $a$ to $b$, $l'$ is an alignment from $a'$ to
  $b'$, $a \neq a'$, and $b \neq b'$, then the difference $l - l' = l$.
- If $l$ is an alignment from $a$ to $b$, $l'$ is an alignment from $a'$ to
  $b'$, $a = a'$, and $b \neq b'$, then the difference $l - l' = l''$,
  defined as follows. Each alignment segment of $l$ is compared to the
  alignment segments of $l'$. If a segment of $l$ overlaps one of the
  segments of $l'$ wrt $a$, it is truncated so as to avoid the overlap.
  This truncation may result in zero, one, or two replacement alignment
  segments. (If the overlapping alignment segment of $l'$ is contained
  strictly within the segment of $l$, wrt $a$, two segments will result.)
- If $l$ is an alignment from $a$ to $b$, $l'$ is an alignment from $a'$ to
  $b'$, $a \neq a'$, and $b = b'$, then the difference $l - l' = l''$,
  defined similarly as in the previous item, except overlaps are examined
  and resolved wrt $b$.

A couple of examples of the above are as follows. The comments in test\_re\_matched.cpp
contain even more examples.

As a first example, consider alignments of an assembly $A = \{a\_0, a\_1,
a\_2\}$ to a reference $B = \{b\_0, b\_1\}$. In the pictures below, each alignment
segment is indicated by a pair diagonal or vertical lines, with its name
(initially $x$, $y$, $z$, $w$) in between the two lines.

```
      b0                    b1
   B  -----------------     -------------
           /    \   /  \   /  | / |
          /      \ /    \ /   |/  |
         /  x     /  y   \  z / w |
        /        / \    / \  /|   |
   A    ---------   --------- ---------
        a0          a1        a2
```

Assume:

- $x > y > z > w$, where $>$ compares alignment size measured by the number of identical bases.
- $y - x < z$.
- $y - x$ is contained in $z$, wrt $A$.

Step 1: Process alignment $x$, resulting in

```
      b0                    b1
   B  ------------------   --------------
           /        /\ \   /  | / |
          /        /  \ \ /   |/  |
         /  x     /    \*\  z / w |        * = y - x
        /        /      / \  /|   |
   A    ---------   --------- ---------
        a0          a1        a2
```

Step 2: Process alignment $z$, resulting in

```
      b0                    b1
   B  -----------------    --------------
           /        /      /    /||
          /        /      /    / ||
         /  x     /      /  z /  *|        * = w - z
        /        /      /    /   ||
   A    ---------   --------- ---------
        a0          a1        a2
```

Now we have a 1-1 mathing. The intervals of $B$ used to compute recall
are as follows:

```
      b0                    b1
   B  -----[--------]--    [----][]------
           /        /      /    /||
          ...
```

As a second example, we start with the same initial set of alignments:

```
      b0                    b1
   B  -----------------     -------------
           /    \   /  \   /  | / |
          /      \ /    \ /   |/  |
         /  x     /  y   \  z / w |
        /        / \    / \  /|   |
   A    ---------   --------- ---------
        a0          a1        a2
```

But we make a slightly different set of assumptions:

- $x > y > w > z$ ($w$ and $z$ are interchanged, compared to the first example).
- $y - x < w$.
- $y - x > z - w$.
- $y - x$ is contained in $z$, wrt $A$.

Step 1: Process alignment $x$, resulting in

```
      b0                    b1
   B  ------------------   --------------
           /        /\ \   /  | / |
          /        /  \ \ /   |/  |
         /  x     /    \*\  z / w |        * = y - x
        /        /      / \  /|   |
   A    ---------   --------- ---------
        a0          a1        a2
```

Step 2: Process alignment w, resulting in

```
      b0                    b1
   B  ------------------   --------------
           /        /\ \   /  |   |
          /        /  \ \ /  /|   |
         /  x     /    \*\ +/ | w |        * = y - x
        /        /      / \/  |   |        + = z - w
   A    ---------   --------- ---------
        a0          a1        a2
```

Step 3: Process alignment $y - x$, resulting in

```
      b0                    b1
   B  ------------------   --------------
           /        /\ \  +/  |   |
          /        /  \*\//   |   |
         /  x     /    \ \    | w |        * = y - x
        /        /     // \   |   |        + = (z - w) - (y - x)
   A    ---------   --------- ---------
        a0          a1        a2
```

Now we have a 1-1 mathing. The intervals of $B$ used to compute recall
are:

```
      b0                    b1
   B  -----[--------][-]   []-[---]------
           /        /\ \  //  |   |        * = y - x
            x         *   +     w          + = (z - w) - (y - x)
          ...
```

## Score definitions: pair precision, recall, and F1

The definitions for pair precision, recall, and F1 are exactly the same as
for nucleotide precision, recall, and F1, except that instead of bases, we
operate on pairs of bases.

For example, consider the reference $B$ (with one transcript) and assembly $A$
(with two contigs), where horizontal position indicates alignment.

```
      B: b   = AGCTCGACGT 
      A: a_1 = AGCT
         a_2 =     CGACGT
```

Here, the transcript recall is 0 (because neither $a\_1$ nor $a\_2$ covers $b$
to $\geq$ 99 percent), but the nucleotide recall is 1 (because $a\_1$ and $a\_2$
jointly cover $b$ completely). The pair recall is somewhere in between, because
the following pairs of $b$ are correctly predicted (represented by an upper
triangular indicator matrix):

```
          First base
      S   AGCTCGACGT
      e A 1111
      c G  111
      o C   11
      n T    1
      d C     111111
        G      11111
      b A       1111
      a C        111
      s G         11
      e T          1
```

## Score definitions: KC and related scores

The kmer compression score (KC score) is a combination of two measures,
weighted kmer recall (WKR) and inverse compression rate (ICR), and is
simply

$$
KC = WKR - ICR.
$$

The WKR measures the fidelity with which a particular assembly
represents the kmer content of the reference sequences. Balancing the WKR, the
ICR measures the degree to which the assembly compresses the
RNA-Seq data. The details of the WKR and ICR measures are provided below.

To compute the WKR, the relative abundances of the reference elements are
required, as specified by --B-expr. Given the reference sequences and
their abundances, a kmer occurrence frequency profile, $p$, is computed, with
individual kmer occurrences weighted by their parent sequences' abundances: for
each kmer $r$, we define

$$
p(r) = \frac{ \sum\_{b \in B} n(r,b) \tau(b) }
{ \sum\_{b \in B} n(b) \tau(b) }
$$

where $B$ is the set of reference sequences, and for each reference sequence
$b \in B$:

- $n(r,b)$ is the number of times the kmer $r$ occurs in $b$,
- $n(b) $ is the total number of kmers in $b$, and
- $\tau(b)$ is the relative abundance of $b$.

Letting $R(A)$ be the set of all kmers in the assembly $A$, the weighted
kmer recall (WKR) is defined as

$$
WKR = \sum\_{r \in R(A)} p(r).
$$

REF-EVAL currently uses --readlen as the kmer length.

Since recall measures only tell half of the story regarding accuracy, the KC
score includes a second term, the ICR, which serves to penalize large
assemblies. We define the inverse compression rate (ICR) of an assembly as

$$
ICR = n\_A/(N L),
$$

where

- $n\_A$ is the total number of bases in the assembly $A$,
- $N$ is the total number of reads, as specified by --num-reads, and
- $L$ is the read length, as specified by --readlen.

## Score definitions: kmer scores

If --weighted=yes, we construct a kmer occurrence frequency profile
$p\_B$ for $B$ exactly as described in the previous section (about the KC
score). We construct a kmer occurrence frequency profile $p\_A$ for $A$
similarly. The relative abundances are specified by --A-expr and
--B-expr.

If --weighted=no, we construct the kmer occurrence frequency
profiles $p\_A$ and $p\_B$ in the same way, except that uniform relative
abundances are used, i.e., $\tau(a) = 1/|A|$ for all $a$ in $A$, and $\tau(b) =
1/|B|$ for all $b$ in $B$, where $|A|$ is the number of contigs in $A$, and
$|B|$ is the number of reference sequences in $B$.

Let $m$ be the ``mean'' profile of $p\_A$ and $p\_B$:

$$
m(r) = (1/2) (p\_A(r) + p\_B(r)) \qquad\hbox{for every kmer $r$}.
$$

The Jensen-Shannon divergence between $p\_A$ and $p\_B$ is defined in terms of
the KL divergence between $p\_A$ and the mean, and $p\_B$ and the mean, as
follows:

- Let $KL(p\_A || m) = \sum\_r p\_A(r) (\log\_2(p\_A(r)) - \log\_2(m(r)))$.
- Let $KL(p\_B || m) = \sum\_r p\_B(r) (\log\_2(p\_B(r)) - \log\_2(m(r)))$.
- Let $JS(p\_A || p\_B) = (1/2) (KL(p\_A || m) + KL(p\_B || m))$.

In the output file, these three scores are denoted
(un)weighted\_kmer\_KL\_A\_to\_M, (un)weighted\_kmer\_KL\_B\_to\_M, and
(un)weighted\_kmer\_jensen\_shannon, respectively.

The Hellinger distance between $p\_A$ and $p\_B$ is defined as

$$
\sqrt{ (1/2) \sum\_r (\sqrt{p\_A(r)} - \sqrt{p\_B(r)})^2 }
$$

The total variation distance between $p\_A$ and $p\_B$ is defined as

$$
(1/2) \sum\_r |p\_A(r) - p\_B(r)|,
$$

where $|\cdot|$ denotes absolute value. Above, $\sum\_r$ denotes a sum over
all possible kmers $r$ (most of which will have $p\_A(r) = p\_B(r) = 0$).
