# Supplementary material for: Evaluation of de novo transcriptome assemblies from RNA-Seq data
Source: Genome Biol. 2014 Dec 21;15(12):553. doi: 10.1186/s13059-014-0553-5 (PMC4298084; doi:10.1186/s13059-014-0553-5)
Supplement: Additional file 3 — Version of the DETONATE source code used for the experiments in this paper. [file 13059_2014_553_MOESM3_ESM.zip › 13059_2014_553_MOESM3_ESM/detonate-1.8.1/ref-eval/sparsehash/sparsehash-2.0.2.tar.gz!/sparsehash-2.0.2/doc/sparse_hash_set.html]

sparse\_hash\_set<Key, HashFcn, EqualKey, Alloc>


*[Note: this document is formatted similarly to the SGI STL
implementation documentation pages, and refers to concepts and classes
defined there. However, neither this document nor the code it
describes is associated with SGI, nor is it necessary to have SGI's
STL implementation installed in order to use this class.]*

# sparse\_hash\_set<Key, HashFcn, EqualKey, Alloc>

sparse\_hash\_set is a Hashed
Associative Container that stores objects of type Key.
sparse\_hash\_set is a Simple
Associative Container, meaning that its value type, as well as its
key type, is key. It is also a
Unique
Associative Container, meaning that no two elements have keys that
compare equal using EqualKey.

Looking up an element in a sparse\_hash\_set by its key is
efficient, so sparse\_hash\_set is useful for "dictionaries"
where the order of elements is irrelevant. If it is important for the
elements to be in a particular order, however, then map is more appropriate.

sparse\_hash\_set is distinguished from other hash-set
implementations by its stingy use of memory and by the ability to save
and restore contents to disk. On the other hand, this hash-set
implementation, while still efficient, is slower than other hash-set
implementations, and it also has requirements -- for instance, for a
distinguished "deleted key" -- that may not be easy for all
applications to satisfy.

This class is appropriate for applications that need to store
large "dictionaries" in memory, or for applications that need these
dictionaries to be persistent.

### Example

(Note: this example uses SGI semantics for `hash<>`
-- the kind used by gcc and most Unix compiler suites -- and not
Dinkumware semantics -- the kind used by Microsoft Visual Studio. If
you are using MSVC, this example will not compile as-is: you'll need
to change `hash` to `hash_compare`, and you
won't use `eqstr` at all. See the MSVC documentation for
`hash_map` and `hash_compare`, for more
details.)

```
#include <iostream>
#include <sparsehash/sparse_hash_set>

using google::sparse_hash_set;      // namespace where class lives by default
using std::cout;
using std::endl;
using ext::hash;  // or __gnu_cxx::hash, or maybe tr1::hash, depending on your OS

struct eqstr
{
  bool operator()(const char* s1, const char* s2) const
  {
    return (s1 == s2) || (s1 && s2 && strcmp(s1, s2) == 0);
  }
};

void lookup(const hash_set<const char*, hash<const char*>, eqstr>& Set,
            const char* word)
{
  sparse_hash_set<const char*, hash<const char*>, eqstr>::const_iterator it
    = Set.find(word);
  cout << word << ": "
       << (it != Set.end() ? "present" : "not present")
       << endl;
}

int main()
{
  sparse_hash_set<const char*, hash<const char*>, eqstr> Set;
  Set.insert("kiwi");
  Set.insert("plum");
  Set.insert("apple");
  Set.insert("mango");
  Set.insert("apricot");
  Set.insert("banana");

  lookup(Set, "mango");
  lookup(Set, "apple");
  lookup(Set, "durian");
}
```

### Definition

Defined in the header sparse\_hash\_set.
This class is not part of the C++ standard, though it is mostly
compatible with the tr1 class `unordered_set`.

### Template parameters

| Parameter | Description | Default |
| --- | --- | --- |
| Key | The hash\_set's key and value type. This is also defined as sparse\_hash\_set::key\_type and sparse\_hash\_set::value\_type. |  |
| HashFcn | The hash function used by the hash\_set. This is also defined as sparse\_hash\_set::hasher.  **Note:** Hashtable performance depends heavily on the choice of hash function. See the performance page for more information. | hash<Key> |
| EqualKey | The hash\_set key equality function: a binary predicate that determines whether two keys are equal. This is also defined as sparse\_hash\_set::key\_equal. | equal\_to<Key> |
| Alloc | The STL allocator to use. By default, uses the provided allocator `libc_allocator_with_realloc`, which likely gives better performance than other STL allocators due to its built-in support for `realloc`, which this container takes advantage of. If you use an allocator other than the default, note that this container imposes an additional requirement on the STL allocator type beyond those in [lib.allocator.requirements]: it does not support allocators that define alternate memory models. That is, it assumes that `pointer`, `const_pointer`, `size_type`, and `difference_type` are just `T*`, `const T*`, `size_t`, and `ptrdiff_t`, respectively. This is also defined as sparse\_hash\_set::allocator\_type. |  |

### Model of

Unique Hashed Associative Container,
Simple Associative Container

### Type requirements

- Key is Assignable.- EqualKey is a Binary Predicate whose argument type is Key.- EqualKey is an equivalence relation.- Alloc is an Allocator.

### Public base classes

None.

### Members

| Member | Where defined | Description |
| --- | --- | --- |
| value\_type | Container | The type of object, T, stored in the hash\_set. |
| key\_type | Associative Container | The key type associated with value\_type. |
| hasher | Hashed Associative Container | The sparse\_hash\_set's hash function. |
| key\_equal | Hashed Associative Container | Function object that compares keys for equality. |
| allocator\_type | Unordered Associative Container (tr1) | The type of the Allocator given as a template parameter. |
| pointer | Container | Pointer to T. |
| reference | Container | Reference to T |
| const\_reference | Container | Const reference to T |
| size\_type | Container | An unsigned integral type. |
| difference\_type | Container | A signed integral type. |
| iterator | Container | Iterator used to iterate through a sparse\_hash\_set. |
| const\_iterator | Container | Const iterator used to iterate through a sparse\_hash\_set. (iterator and const\_iterator are the same type.) |
| local\_iterator | Unordered Associative Container (tr1) | Iterator used to iterate through a subset of sparse\_hash\_set. |
| const\_local\_iterator | Unordered Associative Container (tr1) | Const iterator used to iterate through a subset of sparse\_hash\_set. |
| iterator begin() const | Container | Returns an iterator pointing to the beginning of the sparse\_hash\_set. |
| iterator end() const | Container | Returns an iterator pointing to the end of the sparse\_hash\_set. |
| local\_iterator begin(size\_type i) | Unordered Associative Container (tr1) | Returns a local\_iterator pointing to the beginning of bucket i in the sparse\_hash\_set. |
| local\_iterator end(size\_type i) | Unordered Associative Container (tr1) | Returns a local\_iterator pointing to the end of bucket i in the sparse\_hash\_set. For sparse\_hash\_set, each bucket contains either 0 or 1 item. |
| const\_local\_iterator begin(size\_type i) const | Unordered Associative Container (tr1) | Returns a const\_local\_iterator pointing to the beginning of bucket i in the sparse\_hash\_set. |
| const\_local\_iterator end(size\_type i) const | Unordered Associative Container (tr1) | Returns a const\_local\_iterator pointing to the end of bucket i in the sparse\_hash\_set. For sparse\_hash\_set, each bucket contains either 0 or 1 item. |
| size\_type size() const | Container | Returns the size of the sparse\_hash\_set. |
| size\_type max\_size() const | Container | Returns the largest possible size of the sparse\_hash\_set. |
| bool empty() const | Container | true if the sparse\_hash\_set's size is 0. |
| size\_type bucket\_count() const | Hashed Associative Container | Returns the number of buckets used by the sparse\_hash\_set. |
| size\_type max\_bucket\_count() const | Hashed Associative Container | Returns the largest possible number of buckets used by the sparse\_hash\_set. |
| size\_type bucket\_size(size\_type i) const | Unordered Associative Container (tr1) | Returns the number of elements in bucket i. For sparse\_hash\_set, this will be either 0 or 1. |
| size\_type bucket(const key\_type& key) const | Unordered Associative Container (tr1) | If the key exists in the set, returns the index of the bucket containing the given key, otherwise, return the bucket the key would be inserted into. This value may be passed to begin(size\_type) and end(size\_type). |
| float load\_factor() const | Unordered Associative Container (tr1) | The number of elements in the sparse\_hash\_set divided by the number of buckets. |
| float max\_load\_factor() const | Unordered Associative Container (tr1) | The maximum load factor before increasing the number of buckets in the sparse\_hash\_set. |
| void max\_load\_factor(float new\_grow) | Unordered Associative Container (tr1) | Sets the maximum load factor before increasing the number of buckets in the sparse\_hash\_set. |
| float min\_load\_factor() const | sparse\_hash\_set | The minimum load factor before decreasing the number of buckets in the sparse\_hash\_set. |
| void min\_load\_factor(float new\_grow) | sparse\_hash\_set | Sets the minimum load factor before decreasing the number of buckets in the sparse\_hash\_set. |
| void set\_resizing\_parameters(float shrink, float grow) | sparse\_hash\_set | DEPRECATED. See below. |
| void resize(size\_type n) | Hashed Associative Container | Increases the bucket count to hold at least n items. [2] [3] |
| void rehash(size\_type n) | Unordered Associative Container (tr1) | Increases the bucket count to hold at least n items. This is identical to resize. [2] [3] |
| hasher hash\_funct() const | Hashed Associative Container | Returns the hasher object used by the sparse\_hash\_set. |
| hasher hash\_function() const | Unordered Associative Container (tr1) | Returns the hasher object used by the sparse\_hash\_set. This is idential to hash\_funct. |
| key\_equal key\_eq() const | Hashed Associative Container | Returns the key\_equal object used by the sparse\_hash\_set. |
| allocator\_type get\_allocator() const | Unordered Associative Container (tr1) | Returns the allocator\_type object used by the sparse\_hash\_set: either the one passed in to the constructor, or a default Alloc instance. |
| sparse\_hash\_set() | Container | Creates an empty sparse\_hash\_set. |
| sparse\_hash\_set(size\_type n) | Hashed Associative Container | Creates an empty sparse\_hash\_set that's optimized for holding up to n items. [3] |
| sparse\_hash\_set(size\_type n, const hasher& h) | Hashed Associative Container | Creates an empty sparse\_hash\_set that's optimized for up to n items, using h as the hash function. |
| sparse\_hash\_set(size\_type n, const hasher& h, const key\_equal& k) | Hashed Associative Container | Creates an empty sparse\_hash\_set that's optimized for up to n items, using h as the hash function and k as the key equal function. |
| sparse\_hash\_set(size\_type n, const hasher& h, const key\_equal& k, const allocator\_type& a) | Unordered Associative Container (tr1) | Creates an empty sparse\_hash\_set that's optimized for up to n items, using h as the hash function, k as the key equal function, and a as the allocator object. |
| ``` template <class InputIterator> sparse_hash_set(InputIterator f, InputIterator l) ```  [2] | Unique Hashed Associative Container | Creates a sparse\_hash\_set with a copy of a range. |
| ``` template <class InputIterator> sparse_hash_set(InputIterator f, InputIterator l, size_type n) ```  [2] | Unique Hashed Associative Container | Creates a hash\_set with a copy of a range that's optimized to hold up to n items. |
| ``` template <class InputIterator> sparse_hash_set(InputIterator f, InputIterator l, size_type n, const hasher& h) ```  [2] | Unique Hashed Associative Container | Creates a hash\_set with a copy of a range that's optimized to hold up to n items, using h as the hash function. |
| ``` template <class InputIterator> sparse_hash_set(InputIterator f, InputIterator l, size_type n, const hasher& h, const key_equal& k) ```  [2] | Unique Hashed Associative Container | Creates a hash\_set with a copy of a range that's optimized for holding up to n items, using h as the hash function and k as the key equal function. |
| ``` template <class InputIterator> sparse_hash_set(InputIterator f, InputIterator l, size_type n, const hasher& h, const key_equal& k, const allocator_type& a) ```  [2] | Unordered Associative Container (tr1) | Creates a hash\_set with a copy of a range that's optimized for holding up to n items, using h as the hash function, k as the key equal function, and a as the allocator object. |
| sparse\_hash\_set(const hash\_set&) | Container | The copy constructor. |
| sparse\_hash\_set& operator=(const hash\_set&) | Container | The assignment operator |
| void swap(hash\_set&) | Container | Swaps the contents of two hash\_sets. |
| ``` pair<iterator, bool> insert(const value_type& x) ``` | Unique Associative Container | Inserts x into the sparse\_hash\_set. |
| ``` template <class InputIterator> void insert(InputIterator f, InputIterator l) ```  [2] | Unique Associative Container | Inserts a range into the sparse\_hash\_set. |
| void set\_deleted\_key(const key\_type& key) [4] | sparse\_hash\_set | See below. |
| void clear\_deleted\_key() [4] | sparse\_hash\_set | See below. |
| void erase(iterator pos) | Associative Container | Erases the element pointed to by pos. [4] |
| size\_type erase(const key\_type& k) | Associative Container | Erases the element whose key is k. [4] |
| void erase(iterator first, iterator last) | Associative Container | Erases all elements in a range. [4] |
| void clear() | Associative Container | Erases all of the elements. |
| iterator find(const key\_type& k) const | Associative Container | Finds an element whose key is k. |
| size\_type count(const key\_type& k) const | Unique Associative Container | Counts the number of elements whose key is k. |
| ``` pair<iterator, iterator> equal_range(const key_type& k) const ``` | Associative Container | Finds a range containing all elements whose key is k. |
| template <ValueSerializer, OUTPUT> bool serialize(ValueSerializer serializer, OUTPUT \*fp) | sparse\_hash\_set | See below. |
| template <ValueSerializer, INPUT> bool unserialize(ValueSerializer serializer, INPUT \*fp) | sparse\_hash\_set | See below. |
| NopointerSerializer | sparse\_hash\_set | See below. |
| bool write\_metadata(FILE \*fp) | sparse\_hash\_set | DEPRECATED. See below. |
| bool read\_metadata(FILE \*fp) | sparse\_hash\_set | DEPRECATED. See below. |
| bool write\_nopointer\_data(FILE \*fp) | sparse\_hash\_set | DEPRECATED. See below. |
| bool read\_nopointer\_data(FILE \*fp) | sparse\_hash\_set | DEPRECATED. See below. |
| ``` bool operator==(const hash_set&, const hash_set&) ``` | Hashed Associative Container | Tests two hash\_sets for equality. This is a global function, not a member function. |

### New members

These members are not defined in the Unique
Hashed Associative Container, Simple
Associative Container, or tr1's Unordered Associative
Container requirements, but are specific to
sparse\_hash\_set.

| Member | Description |
| --- | --- |
| void set\_deleted\_key(const key\_type& key) | Sets the distinguished "deleted" key to key. This must be called before any calls to erase(). [4] |
| void clear\_deleted\_key() | Clears the distinguished "deleted" key. After this is called, calls to erase() are not valid on this object. [4] |
 void set\_resizing\_parameters(float shrink, float grow) | This function is DEPRECATED. It is equivalent to calling min\_load\_factor(shrink); max\_load\_factor(grow). || template <ValueSerializer, OUTPUT> bool serialize(ValueSerializer serializer, OUTPUT \*fp) | Emit a serialization of the hash\_set to a stream. See below. |
| template <ValueSerializer, INPUT> bool unserialize(ValueSerializer serializer, INPUT \*fp) | Read in a serialization of a hash\_set from a stream, replacing the existing hash\_set contents with the serialized contents. See below. |
| bool write\_metadata(FILE \*fp) | This function is DEPRECATED. See below. |
| bool read\_metadata(FILE \*fp) | This function is DEPRECATED. See below. |
| bool write\_nopointer\_data(FILE \*fp) | This function is DEPRECATED. See below. |
| bool read\_nopointer\_data(FILE \*fp) | This function is DEPRECATED. See below. |

### Notes

[1]
This member function relies on *member template* functions, which
may not be supported by all compilers. If your compiler supports
member templates, you can call this function with any type of input
iterator. If your compiler does not yet support member templates,
though, then the arguments must either be of type const
value\_type\* or of type sparse\_hash\_set::const\_iterator.

[2]
In order to preserve iterators, erasing hashtable elements does not
cause a hashtable to resize. This means that after a string of
erase() calls, the hashtable will use more space than is
required. At a cost of invalidating all current iterators, you can
call resize() to manually compact the hashtable. The
hashtable promotes too-small resize() arguments to the
smallest legal value, so to compact a hashtable, it's sufficient to
call resize(0).

[3]
Unlike some other hashtable implementations, the optional *n* in
the calls to the constructor, resize, and rehash
indicates not the desired number of buckets that
should be allocated, but instead the expected number of items to be
inserted. The class then sizes the hash-set appropriately for the
number of items specified. It's not an error to actually insert more
or fewer items into the hashtable, but the implementation is most
efficient -- does the fewest hashtable resizes -- if the number of
inserted items is *n* or slightly less.

[4]
sparse\_hash\_set **requires** you call
set\_deleted\_key() before calling erase(). (This is
the largest difference between the sparse\_hash\_set API and
other hash-set APIs. See implementation.html
for why this is necessary.)
The argument to set\_deleted\_key() should be a key-value that
is never used for legitimate hash-set entries. It is an error to call
erase() without first calling set\_deleted\_key(), and
it is also an error to call insert() with an item whose key
is the "deleted key."

There is no need to call set\_deleted\_key if you do not
wish to call erase() on the hash-set.

It is acceptable to change the deleted-key at any time by calling
set\_deleted\_key() with a new argument. You can also call
clear\_deleted\_key(), at which point all keys become valid for
insertion but no hashtable entries can be deleted until
set\_deleted\_key() is called again.

### Input/Output

It is possible to save and restore sparse\_hash\_set objects
to an arbitrary stream (such as a disk file) using the
serialize() and unserialize() methods.

Each of these methods takes two arguments: a *serializer*,
which says how to write hashtable items to disk, and a *stream*,
which can be a C++ stream (istream or its subclasses for
input, ostream or its subclasses for output), a
FILE\*, or a user-defined type (as described below).

The serializer is a functor that takes a stream and a
single hashtable element (a value\_type) and copies the
hashtable element to the stream (for serialize()) or fills
the hashtable element contents from the stream (for
unserialize()), and returns true on success or false on
error. The copy-in and copy-out functions can be provided in a single
functor. Here is a sample serializer that read/writes a hashtable
element for a string hash\_set to a FILE\*:

```
struct StringSerializer {
  bool operator()(FILE* fp, const std::string& value) const {
    assert(value.length() <= 255);   // we only support writing small strings
    const unsigned char size = value.length();
    if (fwrite(&size, 1, 1, fp) != 1)
      return false;
    if (fwrite(value.data(), size, 1, fp) != 1)
      return false;
    return true;
  }
  bool operator()(FILE* fp, std::string* value) const {
    unsigned char size;    // all strings are <= 255 chars long
    if (fread(&size, 1, 1, fp) != 1)
      return false;
    char* buf = new char[size];
    if (fread(buf, size, 1, fp) != 1) {
      delete[] buf;
      return false;
    }
    new(value) string(buf, size);
    delete[] buf;
    return true;
  }
};
```

Here is the functor being used in code (error checking omitted):

```
   sparse_hash_set<string> myset = CreateSet();
   FILE* fp = fopen("hashtable.data", "w");
   myset.serialize(StringSerializer(), fp);
   fclose(fp);

   sparse_hash_set<string> myset2;
   FILE* fp_in = fopen("hashtable.data", "r");
   myset2.unserialize(StringSerializer(), fp_in);
   fclose(fp_in);
   assert(myset == myset2);
```

**Important note:** the code above uses placement-new to
instantiate the string. This is *required* for any
non-POD type. The value\_type passed in to the unserializer
points to garbage memory, so it is not safe to assign to it directly
if doing so causes a destructor to be called.

Also note that this example serializer can only serialize to a
FILE\*. If you want to also be able to use this serializer with C++
streams, you will need to write two more overloads of
operator()'s, one that reads from an istream, and
one that writes to an ostream. Likewise if you want to
support serializing to a custom class.

If the key is "simple" enough, you can use the pre-supplied functor
NopointerSerializer. This copies the hashtable data using
the equivalent of a memcpy<>. Native C data types can be
serialized this way, as can structs of native C data types. Pointers
and STL objects cannot.

Note that NopointerSerializer() does not do any endian
conversion. Thus, it is only appropriate when you intend to read the
data on the same endian architecture as you write the data.

If you wish to serialize to your own stream type, you can do so by
creating an object which supports two methods:

```
   bool Write(const void* data, size_t length);
   bool Read(void* data, size_t length);
```

Write() writes length bytes of data to a
stream (presumably a stream owned by the object), while
Read() reads data bytes from the stream into
data. Both return true on success or false on error.

To unserialize a hashtable from a stream, you wil typically create
a new sparse\_hash\_set object, then call unserialize()
on it. unserialize() destroys the old contents of the
object. You must pass in the appropriate ValueSerializer for
the data being read in.

Both serialize() and unserialize() return
true on success, or false if there was an error
streaming the data.

Note that serialize() is not a const method, since it
purges deleted elements before serializing. It is not safe to
serialize from two threads at once, without synchronization.

NOTE: older versions of sparse\_hash\_set provided a
different API, consisting of read\_metadata(),
read\_nopointer\_data(), write\_metadata(),
write\_nopointer\_data(). Writing to disk consisted of a call
to write\_metadata() followed by
write\_nopointer\_data() (if the hash data was POD) or a custom
loop over the hashtable buckets to write the data (otherwise).
Reading from disk was similar. Prefer the new API for new code.

### Validity of Iterators

erase() is guaranteed not to invalidate any iterators --
except for any iterators pointing to the item being erased, of course.
insert() invalidates all iterators, as does
resize().

This is implemented by making erase() not resize the
hashtable. If you desire maximum space efficiency, you can call
resize(0) after a string of erase() calls, to force
the hashtable to resize to the smallest possible size.

In addition to invalidating iterators, insert()
and resize() invalidate all pointers into the hashtable. If
you want to store a pointer to an object held in a sparse\_hash\_set,
either do so after finishing hashtable inserts, or store the object on
the heap and a pointer to it in the sparse\_hash\_set.

### See also

The following are SGI STL, and some Google STL, concepts and
classes related to sparse\_hash\_set.

hash\_set,
Associative Container,
Hashed Associative Container,
Simple Associative Container,
Unique Hashed Associative Container,
set,
map
multiset,
multimap,
hash\_map,
hash\_multiset,
hash\_multimap,
sparsetable,
sparse\_hash\_map,
dense\_hash\_set,
dense\_hash\_map
