# Supplementary material for: Evaluation of de novo transcriptome assemblies from RNA-Seq data
Source: Genome Biol. 2014 Dec 21;15(12):553. doi: 10.1186/s13059-014-0553-5 (PMC4298084; doi:10.1186/s13059-014-0553-5)
Supplement: Additional file 3 — Version of the DETONATE source code used for the experiments in this paper. [file 13059_2014_553_MOESM3_ESM.zip › 13059_2014_553_MOESM3_ESM/detonate-1.8.1/ref-eval/sparsehash/sparsehash-2.0.2.tar.gz!/sparsehash-2.0.2/doc/dense_hash_map.html]

dense\_hash\_map<Key, Data, HashFcn, EqualKey, Alloc>


*[Note: this document is formatted similarly to the SGI STL
implementation documentation pages, and refers to concepts and classes
defined there. However, neither this document nor the code it
describes is associated with SGI, nor is it necessary to have SGI's
STL implementation installed in order to use this class.]*

# dense\_hash\_map<Key, Data, HashFcn, EqualKey, Alloc>

dense\_hash\_map is a Hashed
Associative Container that associates objects of type Key
with objects of type Data. dense\_hash\_map is a Pair
Associative Container, meaning that its value type is pair<const Key, Data>. It is also a
Unique
Associative Container, meaning that no two elements have keys that
compare equal using EqualKey.

Looking up an element in a dense\_hash\_map by its key is
efficient, so dense\_hash\_map is useful for "dictionaries"
where the order of elements is irrelevant. If it is important for the
elements to be in a particular order, however, then map is more appropriate.

dense\_hash\_map is distinguished from other hash-map
implementations by its speed and by the ability to save
and restore contents to disk. On the other hand, this hash-map
implementation can use significantly more space than other hash-map
implementations, and it also has requirements -- for instance, for a
distinguished "empty key" -- that may not be easy for all
applications to satisfy.

This class is appropriate for applications that need speedy access
to relatively small "dictionaries" stored in memory, or for
applications that need these dictionaries to be persistent. [implementation note])

### Example

(Note: this example uses SGI semantics for `hash<>`
-- the kind used by gcc and most Unix compiler suites -- and not
Dinkumware semantics -- the kind used by Microsoft Visual Studio. If
you are using MSVC, this example will not compile as-is: you'll need
to change `hash` to `hash_compare`, and you
won't use `eqstr` at all. See the MSVC documentation for
`hash_map` and `hash_compare`, for more
details.)

```
#include <iostream>
#include <sparsehash/dense_hash_map>

using google::dense_hash_map;      // namespace where class lives by default
using std::cout;
using std::endl;
using ext::hash;  // or __gnu_cxx::hash, or maybe tr1::hash, depending on your OS

struct eqstr
{
  bool operator()(const char* s1, const char* s2) const
  {
    return (s1 == s2) || (s1 && s2 && strcmp(s1, s2) == 0);
  }
};

int main()
{
  dense_hash_map<const char*, int, hash<const char*>, eqstr> months;
  
  months.set_empty_key(NULL);
  months["january"] = 31;
  months["february"] = 28;
  months["march"] = 31;
  months["april"] = 30;
  months["may"] = 31;
  months["june"] = 30;
  months["july"] = 31;
  months["august"] = 31;
  months["september"] = 30;
  months["october"] = 31;
  months["november"] = 30;
  months["december"] = 31;
  
  cout << "september -> " << months["september"] << endl;
  cout << "april     -> " << months["april"] << endl;
  cout << "june      -> " << months["june"] << endl;
  cout << "november  -> " << months["november"] << endl;
}
```

### Definition

Defined in the header dense\_hash\_map.
This class is not part of the C++ standard, though it is mostly
compatible with the tr1 class `unordered_map`.

### Template parameters

| Parameter | Description | Default |
| --- | --- | --- |
| Key | The hash\_map's key type. This is also defined as dense\_hash\_map::key\_type. |  |
| Data | The hash\_map's data type. This is also defined as dense\_hash\_map::data\_type. [7] |  |
| HashFcn | The hash function used by the hash\_map. This is also defined as dense\_hash\_map::hasher.  **Note:** Hashtable performance depends heavily on the choice of hash function. See the performance page for more information. | hash<Key> |
| EqualKey | The hash\_map key equality function: a binary predicate that determines whether two keys are equal. This is also defined as dense\_hash\_map::key\_equal. | equal\_to<Key> |
| Alloc | The STL allocator to use. By default, uses the provided allocator `libc_allocator_with_realloc`, which likely gives better performance than other STL allocators due to its built-in support for `realloc`, which this container takes advantage of. If you use an allocator other than the default, note that this container imposes an additional requirement on the STL allocator type beyond those in [lib.allocator.requirements]: it does not support allocators that define alternate memory models. That is, it assumes that `pointer`, `const_pointer`, `size_type`, and `difference_type` are just `T*`, `const T*`, `size_t`, and `ptrdiff_t`, respectively. This is also defined as dense\_hash\_map::allocator\_type. |  |

### Model of

Unique Hashed Associative Container,
Pair Associative Container

### Type requirements

- Key is Assignable.- EqualKey is a Binary Predicate whose argument type is Key.- EqualKey is an equivalence relation.- Alloc is an Allocator.

### Public base classes

None.

### Members

| Member | Where defined | Description |
| --- | --- | --- |
| key\_type | Associative Container | The dense\_hash\_map's key type, Key. |
| data\_type | Pair Associative Container | The type of object associated with the keys. |
| value\_type | Pair Associative Container | The type of object, pair<const key\_type, data\_type>, stored in the hash\_map. |
| hasher | Hashed Associative Container | The dense\_hash\_map's hash function. |
| key\_equal | Hashed Associative Container | Function object that compares keys for equality. |
| allocator\_type | Unordered Associative Container (tr1) | The type of the Allocator given as a template parameter. |
| pointer | Container | Pointer to T. |
| reference | Container | Reference to T |
| const\_reference | Container | Const reference to T |
| size\_type | Container | An unsigned integral type. |
| difference\_type | Container | A signed integral type. |
| iterator | Container | Iterator used to iterate through a dense\_hash\_map. [1] |
| const\_iterator | Container | Const iterator used to iterate through a dense\_hash\_map. |
| local\_iterator | Unordered Associative Container (tr1) | Iterator used to iterate through a subset of dense\_hash\_map. [1] |
| const\_local\_iterator | Unordered Associative Container (tr1) | Const iterator used to iterate through a subset of dense\_hash\_map. |
| iterator begin() | Container | Returns an iterator pointing to the beginning of the dense\_hash\_map. |
| iterator end() | Container | Returns an iterator pointing to the end of the dense\_hash\_map. |
| const\_iterator begin() const | Container | Returns an const\_iterator pointing to the beginning of the dense\_hash\_map. |
| const\_iterator end() const | Container | Returns an const\_iterator pointing to the end of the dense\_hash\_map. |
| local\_iterator begin(size\_type i) | Unordered Associative Container (tr1) | Returns a local\_iterator pointing to the beginning of bucket i in the dense\_hash\_map. |
| local\_iterator end(size\_type i) | Unordered Associative Container (tr1) | Returns a local\_iterator pointing to the end of bucket i in the dense\_hash\_map. For dense\_hash\_map, each bucket contains either 0 or 1 item. |
| const\_local\_iterator begin(size\_type i) const | Unordered Associative Container (tr1) | Returns a const\_local\_iterator pointing to the beginning of bucket i in the dense\_hash\_map. |
| const\_local\_iterator end(size\_type i) const | Unordered Associative Container (tr1) | Returns a const\_local\_iterator pointing to the end of bucket i in the dense\_hash\_map. For dense\_hash\_map, each bucket contains either 0 or 1 item. |
| size\_type size() const | Container | Returns the size of the dense\_hash\_map. |
| size\_type max\_size() const | Container | Returns the largest possible size of the dense\_hash\_map. |
| bool empty() const | Container | true if the dense\_hash\_map's size is 0. |
| size\_type bucket\_count() const | Hashed Associative Container | Returns the number of buckets used by the dense\_hash\_map. |
| size\_type max\_bucket\_count() const | Hashed Associative Container | Returns the largest possible number of buckets used by the dense\_hash\_map. |
| size\_type bucket\_size(size\_type i) const | Unordered Associative Container (tr1) | Returns the number of elements in bucket i. For dense\_hash\_map, this will be either 0 or 1. |
| size\_type bucket(const key\_type& key) const | Unordered Associative Container (tr1) | If the key exists in the map, returns the index of the bucket containing the given key, otherwise, return the bucket the key would be inserted into. This value may be passed to begin(size\_type) and end(size\_type). |
| float load\_factor() const | Unordered Associative Container (tr1) | The number of elements in the dense\_hash\_map divided by the number of buckets. |
| float max\_load\_factor() const | Unordered Associative Container (tr1) | The maximum load factor before increasing the number of buckets in the dense\_hash\_map. |
| void max\_load\_factor(float new\_grow) | Unordered Associative Container (tr1) | Sets the maximum load factor before increasing the number of buckets in the dense\_hash\_map. |
| float min\_load\_factor() const | dense\_hash\_map | The minimum load factor before decreasing the number of buckets in the dense\_hash\_map. |
| void min\_load\_factor(float new\_grow) | dense\_hash\_map | Sets the minimum load factor before decreasing the number of buckets in the dense\_hash\_map. |
| void set\_resizing\_parameters(float shrink, float grow) | dense\_hash\_map | DEPRECATED. See below. |
| void resize(size\_type n) | Hashed Associative Container | Increases the bucket count to hold at least n items. [4] [5] |
| void rehash(size\_type n) | Unordered Associative Container (tr1) | Increases the bucket count to hold at least n items. This is identical to resize. [4] [5] |
| hasher hash\_funct() const | Hashed Associative Container | Returns the hasher object used by the dense\_hash\_map. |
| hasher hash\_function() const | Unordered Associative Container (tr1) | Returns the hasher object used by the dense\_hash\_map. This is idential to hash\_funct. |
| key\_equal key\_eq() const | Hashed Associative Container | Returns the key\_equal object used by the dense\_hash\_map. |
| allocator\_type get\_allocator() const | Unordered Associative Container (tr1) | Returns the allocator\_type object used by the dense\_hash\_map: either the one passed in to the constructor, or a default Alloc instance. |
| dense\_hash\_map() | Container | Creates an empty dense\_hash\_map. |
| dense\_hash\_map(size\_type n) | Hashed Associative Container | Creates an empty dense\_hash\_map that's optimized for holding up to n items. [5] |
| dense\_hash\_map(size\_type n, const hasher& h) | Hashed Associative Container | Creates an empty dense\_hash\_map that's optimized for up to n items, using h as the hash function. |
| dense\_hash\_map(size\_type n, const hasher& h, const key\_equal& k) | Hashed Associative Container | Creates an empty dense\_hash\_map that's optimized for up to n items, using h as the hash function and k as the key equal function. |
| dense\_hash\_map(size\_type n, const hasher& h, const key\_equal& k, const allocator\_type& a) | Unordered Associative Container (tr1) | Creates an empty dense\_hash\_map that's optimized for up to n items, using h as the hash function, k as the key equal function, and a as the allocator object. |
| ``` template <class InputIterator> dense_hash_map(InputIterator f, InputIterator l) ```  [2] | Unique Hashed Associative Container | Creates a dense\_hash\_map with a copy of a range. |
| ``` template <class InputIterator> dense_hash_map(InputIterator f, InputIterator l, size_type n) ```  [2] | Unique Hashed Associative Container | Creates a hash\_map with a copy of a range that's optimized to hold up to n items. |
| ``` template <class InputIterator> dense_hash_map(InputIterator f, InputIterator l, size_type n, const hasher& h) ```  [2] | Unique Hashed Associative Container | Creates a hash\_map with a copy of a range that's optimized to hold up to n items, using h as the hash function. |
| ``` template <class InputIterator> dense_hash_map(InputIterator f, InputIterator l, size_type n, const hasher& h, const key_equal& k) ```  [2] | Unique Hashed Associative Container | Creates a hash\_map with a copy of a range that's optimized for holding up to n items, using h as the hash function and k as the key equal function. |
| ``` template <class InputIterator> dense_hash_map(InputIterator f, InputIterator l, size_type n, const hasher& h, const key_equal& k, const allocator_type& a) ```  [2] | Unordered Associative Container (tr1) | Creates a hash\_map with a copy of a range that's optimized for holding up to n items, using h as the hash function, k as the key equal function, and a as the allocator object. |
| dense\_hash\_map(const hash\_map&) | Container | The copy constructor. |
| dense\_hash\_map& operator=(const hash\_map&) | Container | The assignment operator |
| void swap(hash\_map&) | Container | Swaps the contents of two hash\_maps. |
| ``` pair<iterator, bool> insert(const value_type& x) ``` | Unique Associative Container | Inserts x into the dense\_hash\_map. |
| ``` template <class InputIterator> void insert(InputIterator f, InputIterator l) ```  [2] | Unique Associative Container | Inserts a range into the dense\_hash\_map. |
| void set\_empty\_key(const key\_type& key) [6] | dense\_hash\_map | See below. |
| void set\_deleted\_key(const key\_type& key) [6] | dense\_hash\_map | See below. |
| void clear\_deleted\_key() [6] | dense\_hash\_map | See below. |
| void erase(iterator pos) | Associative Container | Erases the element pointed to by pos. [6] |
| size\_type erase(const key\_type& k) | Associative Container | Erases the element whose key is k. [6] |
| void erase(iterator first, iterator last) | Associative Container | Erases all elements in a range. [6] |
| void clear() | Associative Container | Erases all of the elements. |
| void clear\_no\_resize() | dense\_hash\_map | See below. |
| const\_iterator find(const key\_type& k) const | Associative Container | Finds an element whose key is k. |
| iterator find(const key\_type& k) | Associative Container | Finds an element whose key is k. |
| size\_type count(const key\_type& k) const | Unique Associative Container | Counts the number of elements whose key is k. |
| ``` pair<const_iterator, const_iterator> equal_range(const key_type& k) const ``` | Associative Container | Finds a range containing all elements whose key is k. |
| ``` pair<iterator, iterator> equal_range(const key_type& k) ``` | Associative Container | Finds a range containing all elements whose key is k. |
| ``` data_type& operator[](const key_type& k) [3] ``` | dense\_hash\_map | See below. |
| template <ValueSerializer, OUTPUT> bool serialize(ValueSerializer serializer, OUTPUT \*fp) | dense\_hash\_map | See below. |
| template <ValueSerializer, INPUT> bool unserialize(ValueSerializer serializer, INPUT \*fp) | dense\_hash\_map | See below. |
| NopointerSerializer | dense\_hash\_map | See below. |
| bool write\_metadata(FILE \*fp) | dense\_hash\_map | DEPRECATED. See below. |
| bool read\_metadata(FILE \*fp) | dense\_hash\_map | DEPRECATED. See below. |
| bool write\_nopointer\_data(FILE \*fp) | dense\_hash\_map | DEPRECATED. See below. |
| bool read\_nopointer\_data(FILE \*fp) | dense\_hash\_map | DEPRECATED. See below. |
| ``` bool operator==(const hash_map&, const hash_map&) ``` | Hashed Associative Container | Tests two hash\_maps for equality. This is a global function, not a member function. |

### New members

These members are not defined in the Unique
Hashed Associative Container, Pair
Associative Container, or tr1's +Unordered Associative
Container requirements, but are specific to
dense\_hash\_map.

| Member | Description |
| --- | --- |
| void set\_empty\_key(const key\_type& key) | Sets the distinguished "empty" key to key. This must be called immediately after construct time, before calls to another other dense\_hash\_map operation. [6] |
| void set\_deleted\_key(const key\_type& key) | Sets the distinguished "deleted" key to key. This must be called before any calls to erase(). [6] |
| void clear\_deleted\_key() | Clears the distinguished "deleted" key. After this is called, calls to erase() are not valid on this object. [6] |
| void clear\_no\_resize() | Clears the hashtable like clear() does, but does not recover the memory used for hashtable buckets. (The memory used by the *items* in the hashtable is still recovered.) This can save time for applications that want to reuse a dense\_hash\_map many times, each time with a similar number of objects. |
| ``` data_type&  operator[](const key_type& k) [3] ``` | Returns a reference to the object that is associated with a particular key. If the dense\_hash\_map does not already contain such an object, operator[] inserts the default object data\_type(). [3] |
 void set\_resizing\_parameters(float shrink, float grow) | This function is DEPRECATED. It is equivalent to calling min\_load\_factor(shrink); max\_load\_factor(grow). || template <ValueSerializer, OUTPUT> bool serialize(ValueSerializer serializer, OUTPUT \*fp) | Emit a serialization of the hash\_map to a stream. See below. |
| template <ValueSerializer, INPUT> bool unserialize(ValueSerializer serializer, INPUT \*fp) | Read in a serialization of a hash\_map from a stream, replacing the existing hash\_map contents with the serialized contents. See below. |
| bool write\_metadata(FILE \*fp) | This function is DEPRECATED. See below. |
| bool read\_metadata(FILE \*fp) | This function is DEPRECATED. See below. |
| bool write\_nopointer\_data(FILE \*fp) | This function is DEPRECATED. See below. |
| bool read\_nopointer\_data(FILE \*fp) | This function is DEPRECATED. See below. |

### Notes

[1]
dense\_hash\_map::iterator is not a mutable iterator, because
dense\_hash\_map::value\_type is not Assignable.
That is, if i is of type dense\_hash\_map::iterator
and p is of type dense\_hash\_map::value\_type, then
\*i = p is not a valid expression. However,
dense\_hash\_map::iterator isn't a constant iterator either,
because it can be used to modify the object that it points to. Using
the same notation as above, (\*i).second = p is a valid
expression.

[2]
This member function relies on *member template* functions, which
may not be supported by all compilers. If your compiler supports
member templates, you can call this function with any type of input
iterator. If your compiler does not yet support member templates,
though, then the arguments must either be of type const
value\_type\* or of type dense\_hash\_map::const\_iterator.

[3]
Since operator[] might insert a new element into the
dense\_hash\_map, it can't possibly be a const member
function. Note that the definition of operator[] is
extremely simple: m[k] is equivalent to
(\*((m.insert(value\_type(k, data\_type()))).first)).second.
Strictly speaking, this member function is unnecessary: it exists only
for convenience.

[4]
In order to preserve iterators, erasing hashtable elements does not
cause a hashtable to resize. This means that after a string of
erase() calls, the hashtable will use more space than is
required. At a cost of invalidating all current iterators, you can
call resize() to manually compact the hashtable. The
hashtable promotes too-small resize() arguments to the
smallest legal value, so to compact a hashtable, it's sufficient to
call resize(0).

[5]
Unlike some other hashtable implementations, the optional *n* in
the calls to the constructor, resize, and rehash
indicates not the desired number of buckets that
should be allocated, but instead the expected number of items to be
inserted. The class then sizes the hash-map appropriately for the
number of items specified. It's not an error to actually insert more
or fewer items into the hashtable, but the implementation is most
efficient -- does the fewest hashtable resizes -- if the number of
inserted items is *n* or slightly less.

[6]
dense\_hash\_map **requires** you call
set\_empty\_key() immediately after constructing the hash-map,
and before calling any other dense\_hash\_map method. (This is
the largest difference between the dense\_hash\_map API and
other hash-map APIs. See implementation.html
for why this is necessary.)
The argument to set\_empty\_key() should be a key-value that
is never used for legitimate hash-map entries. If you have no such
key value, you will be unable to use dense\_hash\_map. It is
an error to call insert() with an item whose key is the
"empty key."

dense\_hash\_map also requires you call
set\_deleted\_key() before calling erase().
The argument to set\_deleted\_key() should be a key-value that
is never used for legitimate hash-map entries. It must be different
from the key-value used for set\_empty\_key(). It is an error to call
erase() without first calling set\_deleted\_key(), and
it is also an error to call insert() with an item whose key
is the "deleted key."

There is no need to call set\_deleted\_key if you do not
wish to call erase() on the hash-map.

It is acceptable to change the deleted-key at any time by calling
set\_deleted\_key() with a new argument. You can also call
clear\_deleted\_key(), at which point all keys become valid for
insertion but no hashtable entries can be deleted until
set\_deleted\_key() is called again.

[7]
dense\_hash\_map **requires** that data\_type has a
zero-argument default constructor. This is because
dense\_hash\_map uses the special value pair(empty\_key,
data\_type()) to denote empty buckets, and thus needs to be able
to create data\_type using a zero-argument constructor.

If your data\_type does not have a zero-argument default
constructor, there are several workarounds:

- Store a pointer to data\_type in the map, instead of
  data\_type directly. This may yield faster code as
  well, since hashtable-resizes will just have to move pointers
  around, rather than copying the entire data\_type.- Add a zero-argument default constructor to data\_type.- Subclass data\_type and add a zero-argument default
      constructor to the subclass.

### Input/Output

It is possible to save and restore dense\_hash\_map objects
to an arbitrary stream (such as a disk file) using the
serialize() and unserialize() methods.

Each of these methods takes two arguments: a *serializer*,
which says how to write hashtable items to disk, and a *stream*,
which can be a C++ stream (istream or its subclasses for
input, ostream or its subclasses for output), a
FILE\*, or a user-defined type (as described below).

The serializer is a functor that takes a stream and a
single hashtable element (a value\_type, which is a pair of
the key and data) and copies the hashtable element to the stream (for
serialize()) or fills the hashtable element contents from the
stream (for unserialize()), and returns true on success or
false on error. The copy-in and copy-out functions can be provided in
a single functor. Here is a sample serializer that read/writes a hashtable
element for an int-to-string hash\_map to a FILE\*:

```
struct StringToIntSerializer {
  bool operator()(FILE* fp, const std::pair<const int, std::string>& value) const {
    // Write the key.  We ignore endianness for this example.
    if (fwrite(&value.first, sizeof(value.first), 1, fp) != 1)
      return false;
    // Write the value.
    assert(value.second.length() <= 255);   // we only support writing small strings
    const unsigned char size = value.second.length();
    if (fwrite(&size, 1, 1, fp) != 1)
      return false;
    if (fwrite(value.second.data(), size, 1, fp) != 1)
      return false;
    return true;
  }
  bool operator()(FILE* fp, std::pair<const int, std::string>* value) const {
    // Read the key.  Note the need for const_cast to get around
    // the fact hash_map keys are always const.
    if (fread(const_cast<int*>(&value->first), sizeof(value->first), 1, fp) != 1)
      return false;
    // Read the value.
    unsigned char size;    // all strings are <= 255 chars long
    if (fread(&size, 1, 1, fp) != 1)
      return false;
    char* buf = new char[size];
    if (fread(buf, size, 1, fp) != 1) {
      delete[] buf;
      return false;
    }
    value->second.assign(buf, size);
    delete[] buf;
    return true;
  }
};
```

Here is the functor being used in code (error checking omitted):

```
   dense_hash_map<string, int> mymap = CreateMap();
   FILE* fp = fopen("hashtable.data", "w");
   mymap.serialize(StringToIntSerializer(), fp);
   fclose(fp);

   dense_hash_map<string, int> mymap2;
   FILE* fp_in = fopen("hashtable.data", "r");
   mymap2.unserialize(StringToIntSerializer(), fp_in);
   fclose(fp_in);
   assert(mymap == mymap2);
```

Note that this example serializer can only serialize to a FILE\*.
If you want to also be able to use this serializer with C++ streams,
you will need to write two more overloads of operator()'s,
one that reads from an istream, and one that writes to an
ostream. Likewise if you want to support serializing to a
custom class.

If both the key and data are "simple" enough, you can use the
pre-supplied functor NopointerSerializer. This copies the
hashtable data using the equivalent of a memcpy<>. Native C
data types can be serialized this way, as can structs of native C data
types. Pointers and STL objects cannot.

Note that NopointerSerializer() does not do any endian
conversion. Thus, it is only appropriate when you intend to read the
data on the same endian architecture as you write the data.

If you wish to serialize to your own stream type, you can do so by
creating an object which supports two methods:

```
   bool Write(const void* data, size_t length);
   bool Read(void* data, size_t length);
```

Write() writes length bytes of data to a
stream (presumably a stream owned by the object), while
Read() reads data bytes from the stream into
data. Both return true on success or false on error.

To unserialize a hashtable from a stream, you wil typically create
a new dense\_hash\_map object, then call unserialize()
on it. unserialize() destroys the old contents of the
object. You must pass in the appropriate ValueSerializer for
the data being read in.

Both serialize() and unserialize() return
true on success, or false if there was an error
streaming the data.

Note that serialize() is not a const method, since it
purges deleted elements before serializing. It is not safe to
serialize from two threads at once, without synchronization.

NOTE: older versions of dense\_hash\_map provided a
different API, consisting of read\_metadata(),
read\_nopointer\_data(), write\_metadata(),
write\_nopointer\_data(). These methods were never implemented
and always did nothing but return false. You should
exclusively use the new API for serialization.

### Validity of Iterators

erase() is guaranteed not to invalidate any iterators --
except for any iterators pointing to the item being erased, of course.
insert() invalidates all iterators, as does
resize().

This is implemented by making erase() not resize the
hashtable. If you desire maximum space efficiency, you can call
resize(0) after a string of erase() calls, to force
the hashtable to resize to the smallest possible size.

In addition to invalidating iterators, insert()
and resize() invalidate all pointers into the hashtable. If
you want to store a pointer to an object held in a dense\_hash\_map,
either do so after finishing hashtable inserts, or store the object on
the heap and a pointer to it in the dense\_hash\_map.

### See also

The following are SGI STL, and some Google STL, concepts and
classes related to dense\_hash\_map.

hash\_map,
Associative Container,
Hashed Associative Container,
Pair Associative Container,
Unique Hashed Associative Container,
set,
map
multiset,
multimap,
hash\_set,
hash\_multiset,
hash\_multimap,
sparse\_hash\_map,
sparse\_hash\_set,
dense\_hash\_set
