# Supplementary material for: Evaluation of de novo transcriptome assemblies from RNA-Seq data
Source: Genome Biol. 2014 Dec 21;15(12):553. doi: 10.1186/s13059-014-0553-5 (PMC4298084; doi:10.1186/s13059-014-0553-5)
Supplement: Additional file 3 — Version of the DETONATE source code used for the experiments in this paper. [file 13059_2014_553_MOESM3_ESM.zip › 13059_2014_553_MOESM3_ESM/detonate-1.8.1/ref-eval/sparsehash/sparsehash-2.0.2.tar.gz!/sparsehash-2.0.2/doc/performance.html]

Performance notes: sparse\_hash, dense\_hash, sparsetable


## Performance Numbers

Here are some performance numbers from an example desktop machine,
taken from a version of time\_hash\_map that was instrumented to also
report memory allocation information (this modification is not
included by default because it required a big hack to do, including
modifying the STL code to not try to do its own freelist management).

Note there are lots of caveats on these numbers: they may differ from
machine to machine and compiler to compiler, and they only test a very
particular usage pattern that may not match how you use hashtables --
for instance, they test hashtables with very small keys. However,
they're still useful for a baseline comparison of the various
hashtable implementations.

These figures are from a 2.80GHz Pentium 4 with 2G of memory. The
'standard' hash\_map and map implementations are the SGI STL code
included with gcc2. Compiled with gcc2.95.3 -g
-O2

```
======
Average over 10000000 iterations
Wed Dec  8 14:56:38 PST 2004

SPARSE_HASH_MAP:
map_grow                  665 ns
map_predict/grow          303 ns
map_replace               177 ns
map_fetch                 117 ns
map_remove                192 ns
memory used in map_grow    84.3956 Mbytes

DENSE_HASH_MAP:
map_grow                   84 ns
map_predict/grow           22 ns
map_replace                18 ns
map_fetch                  13 ns
map_remove                 23 ns
memory used in map_grow   256.0000 Mbytes

STANDARD HASH_MAP:
map_grow                  162 ns
map_predict/grow          107 ns
map_replace                44 ns
map_fetch                  22 ns
map_remove                124 ns
memory used in map_grow   204.1643 Mbytes

STANDARD MAP:
map_grow                  297 ns
map_predict/grow          282 ns
map_replace               113 ns
map_fetch                 113 ns
map_remove                238 ns
memory used in map_grow   236.8081 Mbytes
```

## A Note on Hash Functions

For good performance, the sparsehash hash routines depend on a good
hash function: one that distributes data evenly. Many hashtable
implementations come with sub-optimal hash functions that can degrade
performance. For instance, the hash function given in Knuth's \_Art of
Computer Programming\_, and the default string hash function in SGI's
STL implementation, both distribute certain data sets unevenly,
leading to poor performance.

As an example, in one test of the default SGI STL string hash
function against the Hsieh hash function (see below), for a particular
set of string keys, the Hsieh function resulted in hashtable lookups
that were 20 times as fast as the STLPort hash function. The string
keys were chosen to be "hard" to hash well, so these results may not
be typical, but they are suggestive.

There has been much research over the years into good hash
functions. Here are some hash functions of note.

- Bob Jenkins: http://burtleburtle.net/bob/hash/- Paul Hsieh: http://www.azillionmonkeys.com/qed/hash.html- Fowler/Noll/Vo (FNV): http://www.isthe.com/chongo/tech/comp/fnv/- MurmurHash: http://murmurhash.googlepages.com/
