# Supplementary material for: Evaluation of de novo transcriptome assemblies from RNA-Seq data
Source: Genome Biol. 2014 Dec 21;15(12):553. doi: 10.1186/s13059-014-0553-5 (PMC4298084; doi:10.1186/s13059-014-0553-5)
Supplement: Additional file 3 — Version of the DETONATE source code used for the experiments in this paper. [file 13059_2014_553_MOESM3_ESM.zip › 13059_2014_553_MOESM3_ESM/detonate-1.8.1/ref-eval/sparsehash/sparsehash-2.0.2.tar.gz!/sparsehash-2.0.2/doc/index.html]

Sparsehash Package (formerly Google Sparsehash)


# Sparsehash Package (formerly Google Sparsehash)

  

The sparsehash package consists of two hashtable
implementations: *sparse*, which is designed to be very space
efficient, and *dense*, which is designed to be very time
efficient. For each one, the package provides both a hash-map and a
hash-set, to mirror the classes in the common STL implementation.

Documentation on how to use these classes:

- sparse\_hash\_map- sparse\_hash\_set- dense\_hash\_map- dense\_hash\_set

In addition to the hash-map (and hash-set) classes, there's also a
lower-level class that implements a "sparse" array. This class can be
useful in its own right; consider using it when you'd normally use a
`sparse_hash_map`, but your keys are all small-ish
integers.

- sparsetable

There is also a doc explaining the implementation details of these
classes, for those who are curious. And finally, you can see some
performance comparisons, both between
the various classes here, but also between these implementations and
other standard hashtable implementations.

---


Craig Silverstein  
Last modified: Thu Jan 25 17:58:02 PST 2007
