# Supplementary material for: Evaluation of de novo transcriptome assemblies from RNA-Seq data
Source: Genome Biol. 2014 Dec 21;15(12):553. doi: 10.1186/s13059-014-0553-5 (PMC4298084; doi:10.1186/s13059-014-0553-5)
Supplement: Additional file 3 — Version of the DETONATE source code used for the experiments in this paper. [file 13059_2014_553_MOESM3_ESM.zip › 13059_2014_553_MOESM3_ESM/detonate-1.8.1/ref-eval/sparsehash/sparsehash-2.0.2.tar.gz!/sparsehash-2.0.2/doc/sparsetable.html]

sparsetable<T, GROUP\_SIZE>


*[Note: this document is formatted similarly to the SGI STL
implementation documentation pages, and refers to concepts and classes
defined there. However, neither this document nor the code it
describes is associated with SGI, nor is it necessary to have SGI's
STL implementation installed in order to use this class.]*

# sparsetable<T, GROUP\_SIZE>

A sparsetable is a Random
Access Container that supports constant time random access to
elements, and constant time insertion and removal of elements. It
implements the "array" or "table" abstract data type. The number of
elements in a sparsetable is set at constructor time, though
you can change it at any time by calling resize().

sparsetable is distinguished from other array
implementations, including the default C implementation, in its stingy
use of memory -- in particular, unused array elements require only 1 bit
of disk space to store, rather than sizeof(T) bytes -- and by
the ability to save and restore contents to disk. On the other hand,
this array implementation, while still efficient, is slower than other
array implementations.

A sparsetable distinguishes between table elements that
have been *assigned* and those that are *unassigned*.
Assigned table elements are those that have had a value set via
set(), operator(), assignment via an iterator, and
so forth. Unassigned table elements are those that have not had a
value set in one of these ways, or that have been explicitly
unassigned via a call to erase() or clear(). Lookup
is valid on both assigned and unassigned table elements; for
unassigned elements, lookup returns the default value
T().

This class is appropriate for applications that need to store large
arrays in memory, or for applications that need these arrays to be
persistent.

### Example

```
#include <sparsehash/sparsetable>

using google::sparsetable;      // namespace where class lives by default

sparsetable<int> t(100);
t[5] = 6;
cout << "t[5] = " << t[5];
cout << "Default value = " << t[99];
```

### Definition

Defined in the header sparsetable. This
class is not part of the C++ standard.

### Template parameters

| Parameter | Description | Default |
| --- | --- | --- |
| T | The sparsetable's value type: the type of object that is stored in the table. |  |
| GROUP\_SIZE | The number of elements in each sparsetable group (see the implementation doc for more details on this value). This almost never need be specified; the default template parameter value works well in all situations. |  |

### Model of

Random Access Container

### Type requirements

None, except for those imposed by the requirements of
Random
Access Container

### Public base classes

None.

### Members

| Member | Where defined | Description |
| --- | --- | --- |
| value\_type | Container | The type of object, T, stored in the table. |
| pointer | Container | Pointer to T. |
| reference | Container | Reference to T. |
| const\_reference | Container | Const reference to T. |
| size\_type | Container | An unsigned integral type. |
| difference\_type | Container | A signed integral type. |
| iterator | Container | Iterator used to iterate through a sparsetable. |
| const\_iterator | Container | Const iterator used to iterate through a sparsetable. |
| reverse\_iterator | Reversible Container | Iterator used to iterate backwards through a sparsetable. |
| const\_reverse\_iterator | Reversible Container | Const iterator used to iterate backwards through a sparsetable. |
| nonempty\_iterator | sparsetable | Iterator used to iterate through the assigned elements of the sparsetable. |
| const\_nonempty\_iterator | sparsetable | Const iterator used to iterate through the assigned elements of the sparsetable. |
| reverse\_nonempty\_iterator | sparsetable | Iterator used to iterate backwards through the assigned elements of the sparsetable. |
| const\_reverse\_nonempty\_iterator | sparsetable | Const iterator used to iterate backwards through the assigned elements of the sparsetable. |
| destructive\_iterator | sparsetable | Iterator used to iterate through the assigned elements of the sparsetable, erasing elements as it iterates. [1] |
| iterator begin() | Container | Returns an iterator pointing to the beginning of the sparsetable. |
| iterator end() | Container | Returns an iterator pointing to the end of the sparsetable. |
| const\_iterator begin() const | Container | Returns an const\_iterator pointing to the beginning of the sparsetable. |
| const\_iterator end() const | Container | Returns an const\_iterator pointing to the end of the sparsetable. |
| reverse\_iterator rbegin() | Reversible Container | Returns a reverse\_iterator pointing to the beginning of the reversed sparsetable. |
| reverse\_iterator rend() | Reversible Container | Returns a reverse\_iterator pointing to the end of the reversed sparsetable. |
| const\_reverse\_iterator rbegin() const | Reversible Container | Returns a const\_reverse\_iterator pointing to the beginning of the reversed sparsetable. |
| const\_reverse\_iterator rend() const | Reversible Container | Returns a const\_reverse\_iterator pointing to the end of the reversed sparsetable. |
| nonempty\_iterator nonempty\_begin() | sparsetable | Returns a nonempty\_iterator pointing to the first assigned element of the sparsetable. |
| nonempty\_iterator nonempty\_end() | sparsetable | Returns a nonempty\_iterator pointing to the end of the sparsetable. |
| const\_nonempty\_iterator nonempty\_begin() const | sparsetable | Returns a const\_nonempty\_iterator pointing to the first assigned element of the sparsetable. |
| const\_nonempty\_iterator nonempty\_end() const | sparsetable | Returns a const\_nonempty\_iterator pointing to the end of the sparsetable. |
| reverse\_nonempty\_iterator nonempty\_rbegin() | sparsetable | Returns a reverse\_nonempty\_iterator pointing to the first assigned element of the reversed sparsetable. |
| reverse\_nonempty\_iterator nonempty\_rend() | sparsetable | Returns a reverse\_nonempty\_iterator pointing to the end of the reversed sparsetable. |
| const\_reverse\_nonempty\_iterator nonempty\_rbegin() const | sparsetable | Returns a const\_reverse\_nonempty\_iterator pointing to the first assigned element of the reversed sparsetable. |
| const\_reverse\_nonempty\_iterator nonempty\_rend() const | sparsetable | Returns a const\_reverse\_nonempty\_iterator pointing to the end of the reversed sparsetable. |
| destructive\_iterator destructive\_begin() | sparsetable | Returns a destructive\_iterator pointing to the first assigned element of the sparsetable. |
| destructive\_iterator destructive\_end() | sparsetable | Returns a destructive\_iterator pointing to the end of the sparsetable. |
| size\_type size() const | Container | Returns the size of the sparsetable. |
| size\_type max\_size() const | Container | Returns the largest possible size of the sparsetable. |
| bool empty() const | Container | true if the sparsetable's size is 0. |
| size\_type num\_nonempty() const | sparsetable | Returns the number of sparsetable elements that are currently assigned. |
| sparsetable(size\_type n) | Container | Creates a sparsetable with n elements. |
| sparsetable(const sparsetable&) | Container | The copy constructor. |
| ~sparsetable() | Container | The destructor. |
| sparsetable& operator=(const sparsetable&) | Container | The assignment operator |
| void swap(sparsetable&) | Container | Swaps the contents of two sparsetables. |
| reference operator[](size\_type n) | Random Access Container | Returns the n'th element. [2] |
| const\_reference operator[](size\_type n) const | Random Access Container | Returns the n'th element. |
| bool test(size\_type i) const | sparsetable | true if the i'th element of the sparsetable is assigned. |
| bool test(iterator pos) const | sparsetable | true if the sparsetable element pointed to by pos is assigned. |
| bool test(const\_iterator pos) const | sparsetable | true if the sparsetable element pointed to by pos is assigned. |
| const\_reference get(size\_type i) const | sparsetable | returns the i'th element of the sparsetable. |
| reference set(size\_type i, const\_reference val) | sparsetable | Sets the i'th element of the sparsetable to value val. |
| void erase(size\_type i) | sparsetable | Erases the i'th element of the sparsetable. |
| void erase(iterator pos) | sparsetable | Erases the element of the sparsetable pointed to by pos. |
| void erase(iterator first, iterator last) | sparsetable | Erases the elements of the sparsetable in the range [first, last). |
| void clear() | sparsetable | Erases all of the elements. |
| void resize(size\_type n) | sparsetable | Changes the size of sparsetable to n. |
| bool write\_metadata(FILE \*fp) | sparsetable | See below. |
| bool read\_metadata(FILE \*fp) | sparsetable | See below. |
| bool write\_nopointer\_data(FILE \*fp) | sparsetable | See below. |
| bool read\_nopointer\_data(FILE \*fp) | sparsetable | See below. |
| ``` bool operator==(const sparsetable&, const sparsetable&) ``` | Forward Container | Tests two sparsetables for equality. This is a global function, not a member function. |
| ``` bool operator<(const sparsetable&, const sparsetable&) ``` | Forward Container | Lexicographical comparison. This is a global function, not a member function. |

### New members

These members are not defined in the Random
Access Container requirement, but are specific to
sparsetable.

| Member | Description |
| --- | --- |
| nonempty\_iterator | Iterator used to iterate through the assigned elements of the sparsetable. |
| const\_nonempty\_iterator | Const iterator used to iterate through the assigned elements of the sparsetable. |
| reverse\_nonempty\_iterator | Iterator used to iterate backwards through the assigned elements of the sparsetable. |
| const\_reverse\_nonempty\_iterator | Const iterator used to iterate backwards through the assigned elements of the sparsetable. |
| destructive\_iterator | Iterator used to iterate through the assigned elements of the sparsetable, erasing elements as it iterates. [1] |
| nonempty\_iterator nonempty\_begin() | Returns a nonempty\_iterator pointing to the first assigned element of the sparsetable. |
| nonempty\_iterator nonempty\_end() | Returns a nonempty\_iterator pointing to the end of the sparsetable. |
| const\_nonempty\_iterator nonempty\_begin() const | Returns a const\_nonempty\_iterator pointing to the first assigned element of the sparsetable. |
| const\_nonempty\_iterator nonempty\_end() const | Returns a const\_nonempty\_iterator pointing to the end of the sparsetable. |
| reverse\_nonempty\_iterator nonempty\_rbegin() | Returns a reverse\_nonempty\_iterator pointing to the first assigned element of the reversed sparsetable. |
| reverse\_nonempty\_iterator nonempty\_rend() | Returns a reverse\_nonempty\_iterator pointing to the end of the reversed sparsetable. |
| const\_reverse\_nonempty\_iterator nonempty\_rbegin() const | Returns a const\_reverse\_nonempty\_iterator pointing to the first assigned element of the reversed sparsetable. |
| const\_reverse\_nonempty\_iterator nonempty\_rend() const | Returns a const\_reverse\_nonempty\_iterator pointing to the end of the reversed sparsetable. |
| destructive\_iterator destructive\_begin() | Returns a destructive\_iterator pointing to the first assigned element of the sparsetable. |
| destructive\_iterator destructive\_end() | Returns a destructive\_iterator pointing to the end of the sparsetable. |
| size\_type num\_nonempty() const | Returns the number of sparsetable elements that are currently assigned. |
| bool test(size\_type i) const | true if the i'th element of the sparsetable is assigned. |
| bool test(iterator pos) const | true if the sparsetable element pointed to by pos is assigned. |
| bool test(const\_iterator pos) const | true if the sparsetable element pointed to by pos is assigned. |
| const\_reference get(size\_type i) const | returns the i'th element of the sparsetable. If the i'th element is assigned, the assigned value is returned, otherwise, the default value T() is returned. |
| reference set(size\_type i, const\_reference val) | Sets the i'th element of the sparsetable to value val, and returns a reference to the i'th element of the table. This operation causes the i'th element to be assigned. |
| void erase(size\_type i) | Erases the i'th element of the sparsetable. This operation causes the i'th element to be unassigned. |
| void erase(iterator pos) | Erases the element of the sparsetable pointed to by pos. This operation causes the i'th element to be unassigned. |
| void erase(iterator first, iterator last) | Erases the elements of the sparsetable in the range [first, last). This operation causes these elements to be unassigned. |
| void clear() | Erases all of the elements. This causes all elements to be unassigned. |
| void resize(size\_type n) | Changes the size of sparsetable to n. If n is greater than the old size, new, unassigned elements are appended. If n is less than the old size, all elements in position >n are deleted. |
| bool write\_metadata(FILE \*fp) | Write hashtable metadata to fp. See below. |
| bool read\_metadata(FILE \*fp) | Read hashtable metadata from fp. See below. |
| bool write\_nopointer\_data(FILE \*fp) | Write hashtable contents to fp. This is valid only if the hashtable key and value are "plain" data. See below. |
| bool read\_nopointer\_data(FILE \*fp) | Read hashtable contents to fp. This is valid only if the hashtable key and value are "plain" data. See below. |

### Notes

[1]
sparsetable::destructive\_iterator iterates through a
sparsetable like a normal iterator, but ++it may delete the
element being iterated past. Obviously, this iterator can only be
used once on a given table! One application of this iterator is to
copy data from a sparsetable to some other data structure without
using extra memory to store the data in both places during the
copy.

[2]
Since operator[] might insert a new element into the
sparsetable, it can't possibly be a const member
function. In theory, since it might insert a new element, it should
cause the element it refers to to become assigned. However, this is undesirable when
operator[] is used to examine elements, rather than assign
them. Thus, as an implementation trick, operator[] does not
really return a reference. Instead it returns an object that
behaves almost exactly like a reference. This object,
however, delays setting the appropriate sparsetable element to assigned to when it is actually assigned to.

For a bit more detail: the object returned by operator[]
is an opaque type which defines operator=, operator
reference(), and operator&. The first operator controls
assigning to the value. The second controls examining the value. The
third controls pointing to the value.

All three operators perform exactly as an object of type
reference would perform. The only problems that arise is
when this object is accessed in situations where C++ cannot do the
conversion by default. By far the most common situation is with
variadic functions such as printf. In such situations, you
may need to manually cast the object to the right type:

```
   printf("%d", static_cast<typename table::reference>(table[i]));
```

### Input/Output

It is possible to save and restore sparsetable objects
to disk. Storage takes place in two steps. The first writes the
table metadata. The second writes the actual data.

To write a sparsetable to disk, first call write\_metadata()
on an open file pointer. This saves the sparsetable information in a
byte-order-independent format.

After the metadata has been written to disk, you must write the
actual data stored in the sparsetable to disk. If the value is
"simple" enough, you can do this by calling
write\_nopointer\_data(). "Simple" data is data that can be
safely copied to disk via fwrite(). Native C data types fall
into this category, as do structs of native C data types. Pointers
and STL objects do not.

Note that write\_nopointer\_data() does not do any endian
conversion. Thus, it is only appropriate when you intend to read the
data on the same endian architecture as you write the data.

If you cannot use write\_nopointer\_data() for any reason,
you can write the data yourself by iterating over the
sparsetable with a const\_nonempty\_iterator and
writing the key and data in any manner you wish.

To read the hashtable information from disk, first you must create
a sparsetable object. Then open a file pointer to point
to the saved sparsetable, and call read\_metadata(). If you
saved the data via write\_nopointer\_data(), you can follow the
read\_metadata() call with a call to
read\_nopointer\_data(). This is all that is needed.

If you saved the data through a custom write routine, you must call
a custom read routine to read in the data. To do this, iterate over
the sparsetable with a nonempty\_iterator; this
operation is sensical because the metadata has already been set up.
For each iterator item, you can read the key and value from disk, and
set it appropriately. The code might look like this:

```
   for (sparsetable<int*>::nonempty_iterator it = t.nonempty_begin();
        it != t.nonempty_end(); ++it) {
       *it = new int;
       fread(*it, sizeof(int), 1, fp);
   }
```

Here's another example, where the item stored in the sparsetable is
a C++ object with a non-trivial constructor. In this case, you must
use "placement new" to construct the object at the correct memory
location.

```
   for (sparsetable<ComplicatedCppClass>::nonempty_iterator it = t.nonempty_begin();
        it != t.nonempty_end(); ++it) {
       int constructor_arg;   // ComplicatedCppClass takes an int to construct
       fread(&constructor_arg, sizeof(int), 1, fp);
       new (&(*it)) ComplicatedCppClass(constructor_arg);     // placement new
   }
```

### See also

The following are SGI STL concepts and classes related to
sparsetable.

Container,
Random Access Container,
sparse\_hash\_set,
sparse\_hash\_map
