# Supplementary material for: Evaluation of de novo transcriptome assemblies from RNA-Seq data
Source: Genome Biol. 2014 Dec 21;15(12):553. doi: 10.1186/s13059-014-0553-5 (PMC4298084; doi:10.1186/s13059-014-0553-5)
Supplement: Additional file 3 — Version of the DETONATE source code used for the experiments in this paper. [file 13059_2014_553_MOESM3_ESM.zip › 13059_2014_553_MOESM3_ESM/detonate-1.8.1/ref-eval/sparsehash/sparsehash-2.0.2.tar.gz!/sparsehash-2.0.2/doc/implementation.html]

Implementation notes: sparse\_hash, dense\_hash, sparsetable


# Implementation of sparse\_hash\_map, dense\_hash\_map, and sparsetable

This document contains a few notes on how the data structures in this
package are implemented. This discussion refers at several points to
the classic text in this area: Knuth, *The Art of Computer
Programming*, Vol 3, Hashing.

---

## sparsetable

For specificity, consider the declaration

```
   sparsetable<Foo> t(100);        // a sparse array with 100 elements
```

A sparsetable is a random container that implements a sparse array,
that is, an array that uses very little memory to store unassigned
indices (in this case, between 1-2 bits per unassigned index). For
instance, if you allocate an array of size 5 and assign a[2] = [big
struct], then a[2] will take up a lot of memory but a[0], a[1], a[3],
and a[4] will not. Array elements that have a value are called
"assigned". Array elements that have no value yet, or have had their
value cleared using erase() or clear(), are called "unassigned".
For assigned elements, lookups return the assigned value; for
unassigned elements, they return the default value, which for t is
Foo().

sparsetable is implemented as an array of "groups". Each group is
responsible for M array indices. The first group knows about
t[0]..t[M-1], the second about t[M]..t[2M-1], and so forth. (M is 48
by default.) At construct time, t creates an array of (99/M + 1)
groups. From this point on, all operations -- insert, delete, lookup
-- are passed to the appropriate group. In particular, any operation
on t[i] is actually performed on (t.group[i / M])[i % M].

Each group contains of a vector, which holds assigned values, and a
bitmap of size M, which indicates which indices are assigned. A
lookup works as follows: the group is asked to look up index i, where
i < M. The group looks at bitmap[i]. If it's 0, the lookup fails.
If it's 1, then the group has to find the appropriate value in the
vector.

### find()

Finding the appropriate vector element is the most expensive part of
the lookup. The code counts all bitmap entries <= i that are set to
1. (There's at least 1 of them, since bitmap[i] is 1.) Suppose there
are 4 such entries. Then the right value to return is the 4th element
of the vector: vector[3]. This takes time O(M), which is a constant
since M is a constant.

### insert()

Insert starts with a lookup. If the lookup succeeds, the code merely
replaces vector[3] with the new value. If the lookup fails, then the
code must insert a new entry into the middle of the vector. Again, to
insert at position i, the code must count all the bitmap entries <= i
that are set to i. This indicates the position to insert into the
vector. All vector entries above that position must be moved to make
room for the new entry. This takes time, but still constant time
since the vector has size at most M.

(Inserts could be made faster by using a list instead of a vector to
hold group values, but this would use much more memory, since each
list element requires a full pointer of overhead.)

The only metadata that needs to be updated, after the actual value is
inserted, is to set bitmap[i] to 1. No other counts must be
maintained.

### delete()

Deletes are similar to inserts. They start with a lookup. If it
fails, the delete is a noop. Otherwise, the appropriate entry is
removed from the vector, all the vector elements above it are moved
down one, and bitmap[i] is set to 0.

### iterators

Sparsetable iterators pose a special burden. They must iterate over
unassigned array values, but the act of iterating should not cause an
assignment to happen -- otherwise, iterating over a sparsetable would
cause it to take up much more room. For const iterators, the matter
is simple: the iterator is merely programmed to return the default
value -- Foo() -- when dereferenced while pointing to an unassigned
entry.

For non-const iterators, such simple techniques fail. Instead,
dereferencing a sparsetable\_iterator returns an opaque object that
acts like a Foo in almost all situations, but isn't actually a Foo.
(It does this by defining operator=(), operator value\_type(), and,
most sneakily, operator&().) This works in almost all cases. If it
doesn't, an explicit cast to value\_type will solve the problem:

```
   printf("%d", static_cast<Foo>(*t.find(0)));
```

To avoid such problems, consider using get() and set() instead of an
iterator:

```
   for (int i = 0; i < t.size(); ++i)
      if (t.get(i) == ...)  t.set(i, ...);
```

Sparsetable also has a special class of iterator, besides normal and
const: nonempty\_iterator. This only iterates over array values that
are assigned. This is particularly fast given the sparsetable
implementation, since it can ignore the bitmaps entirely and just
iterate over the various group vectors.

### Resource use

The space overhead for an sparsetable of size N is N + 48N/M bits.
For the default value of M, this is exactly 2 bits per array entry.
(That's for 32-bit pointers; for machines with 64-bit pointers, it's N
+ 80N/M bits, or 2.67 bits per entry.)
A larger M would use less overhead -- approaching 1 bit per array
entry -- but take longer for inserts, deletes, and lookups. A smaller
M would use more overhead but make operations somewhat faster.

You can also look at some specific performance numbers.

---

## sparse\_hash\_set

For specificity, consider the declaration

```
   sparse_hash_set<Foo> t;
```

sparse\_hash\_set is a hashtable. For more information on hashtables,
see Knuth. Hashtables are basically arrays with complicated logic on
top of them. sparse\_hash\_set uses a sparsetable to implement the
underlying array.

In particular, sparse\_hash\_set stores its data in a sparsetable using
quadratic internal probing (see Knuth). Many hashtable
implementations use external probing, so each table element is
actually a pointer chain, holding many hashtable values.
sparse\_hash\_set, on the other hand, always stores at most one value in
each table location. If the hashtable wants to store a second value
at a given table location, it can't; it's forced to look somewhere
else.

### insert()

As a specific example, suppose t is a new sparse\_hash\_set. It then
holds a sparsetable of size 32. The code for t.insert(foo) works as
follows:

1) Call hash<Foo>(foo) to convert foo into an integer i. (hash<Foo> is
the default hash function; you can specify a different one in the
template arguments.)

2a) Look at t.sparsetable[i % 32]. If it's unassigned, assign it to
foo. foo is now in the hashtable.

2b) If t.sparsetable[i % 32] is assigned, and its value is foo, then
do nothing: foo was already in t and the insert is a noop.

2c) If t.sparsetable[i % 32] is assigned, but to a value other than
foo, look at t.sparsetable[(i+1) % 32]. If that also fails, try
t.sparsetable[(i+3) % 32], then t.sparsetable[(i+6) % 32]. In
general, keep trying the next triangular number.

3) If the table is now "too full" -- say, 25 of the 32 table entries
are now assigned -- grow the table by creating a new sparsetable
that's twice as big, and rehashing every single element from the
old table into the new one. This keeps the table from ever filling
up.

4) If the table is now "too empty" -- say, only 3 of the 32 table
entries are now assigned -- shrink the table by creating a new
sparsetable that's half as big, and rehashing every element as in
the growing case. This keeps the table overhead proportional to
the number of elements in the table.

Instead of using triangular numbers as offsets, one could just use
regular integers: try i, then i+1, then i+2, then i+3. This has bad
'clumping' behavior, as explored in Knuth. Quadratic probing, using
the triangular numbers, avoids the clumping while keeping cache
coherency in the common case. As long as the table size is a power of
2, the quadratic-probing method described above will explore every
table element if necessary, to find a good place to insert.

(As a side note, using a table size that's a power of two has several
advantages, including the speed of calculating (i % table\_size). On
the other hand, power-of-two tables are not very forgiving of a poor
hash function. Make sure your hash function is a good one! There are
plenty of dos and don'ts on the web (and in Knuth), for writing hash
functions.)

The "too full" value, also called the "maximum occupancy", determines
a time-space tradeoff: in general, the higher it is, the less space is
wasted but the more probes must be performed for each insert.
sparse\_hash\_set uses a high maximum occupancy, since space is more
important than speed for this data structure.

The "too empty" value is not necessary for performance but helps with
space use. It's rare for hashtable implementations to check this
value at insert() time -- after all, how will inserting cause a
hashtable to get too small? However, the sparse\_hash\_set
implementation never resizes on erase(); it's nice to have an erase()
that does not invalidate iterators. Thus, the first insert() after a
long string of erase()s could well trigger a hashtable shrink.

### find()

find() works similarly to insert. The only difference is in step
(2a): if the value is unassigned, then the lookup fails immediately.

### delete()

delete() is tricky in an internal-probing scheme. The obvious
implementation of just "unassigning" the relevant table entry doesn't
work. Consider the following scenario:

```
    t.insert(foo1);         // foo1 hashes to 4, is put in table[4]
    t.insert(foo2);         // foo2 hashes to 4, is put in table[5]
    t.erase(foo1);          // table[4] is now 'unassigned'
    t.lookup(foo2);         // fails since table[hash(foo2)] is unassigned
```

To avoid these failure situations, delete(foo1) is actually
implemented by replacing foo1 by a special 'delete' value in the
hashtable. This 'delete' value causes the table entry to be
considered unassigned for the purposes of insertion -- if foo3 hashes
to 4 as well, it can go into table[4] no problem -- but assigned for
the purposes of lookup.

What is this special 'delete' value? The delete value has to be an
element of type Foo, since the table can't hold anything else. It
obviously must be an element the client would never want to insert on
its own, or else the code couldn't distinguish deleted entries from
'real' entries with the same value. There's no way to determine a
good value automatically. The client has to specify it explicitly.
This is what the set\_deleted\_key() method does.

Note that set\_deleted\_key() is only necessary if the client actually
wants to call t.erase(). For insert-only hash-sets, set\_deleted\_key()
is unnecessary.

When copying the hashtable, either to grow it or shrink it, the
special 'delete' values are **not** copied into the new table. The
copy-time rehash makes them unnecessary.

### Resource use

The data is stored in a sparsetable, so space use is the same as
for sparsetable. However, by default the sparse\_hash\_set
implementation tries to keep about half the table buckets empty, to
keep lookup-chains short. Since sparsehashmap has about 2 bits
overhead per bucket (or 2.5 bits on 64-bit systems), sparse\_hash\_map
has about 4-5 bits overhead per hashtable item.

Time use is also determined in large part by the sparsetable
implementation. However, there is also an extra probing cost in
hashtables, which depends in large part on the "too full" value. It
should be rare to need more than 4-5 probes per lookup, and usually
significantly less will suffice.

A note on growing and shrinking the hashtable: all hashtable
implementations use the most memory when growing a hashtable, since
they must have room for both the old table and the new table at the
same time. sparse\_hash\_set is careful to delete entries from the old
hashtable as soon as they're copied into the new one, to minimize this
space overhead. (It does this efficiently by using its knowledge of
the sparsetable class and copying one sparsetable group at a time.)

You can also look at some specific performance numbers.

---

## sparse\_hash\_map

sparse\_hash\_map is implemented identically to sparse\_hash\_set. The
only difference is instead of storing just Foo in each table entry,
the data structure stores pair<Foo, Value>.

---

## dense\_hash\_set

The hashtable aspects of dense\_hash\_set are identical to
sparse\_hash\_set: it uses quadratic internal probing, and resizes
hashtables in exactly the same way. The difference is in the
underlying array: instead of using a sparsetable, dense\_hash\_set uses
a C array. This means much more space is used, especially if Foo is
big. However, it makes all operations faster, since sparsetable has
memory management overhead that C arrays do not.

The use of C arrays instead of sparsetables points to one immediate
complication dense\_hash\_set has that sparse\_hash\_set does not: the
need to distinguish assigned from unassigned entries. In a
sparsetable, this is accomplished by a bitmap. dense\_hash\_set, on the
other hand, uses a dedicated value to specify unassigned entries.
Thus, dense\_hash\_set has two special values: one to indicate deleted
table entries, and one to indicated unassigned table entries. At
construct time, all table entries are initialized to 'unassigned'.

dense\_hash\_set provides the method set\_empty\_key() to indicate the
value that should be used for unassigned entries. Like
set\_deleted\_key(), set\_empty\_key() requires a value that will not be
used by the client for any legitimate purpose. Unlike
set\_deleted\_key(), set\_empty\_key() is always required, no matter what
hashtable operations the client wishes to perform.

### Resource use

This implementation is fast because even though dense\_hash\_set may not
be space efficient, most lookups are localized: a single lookup may
need to access table[i], and maybe table[i+1] and table[i+3], but
nothing other than that. For all but the biggest data structures,
these will frequently be in a single cache line.

This implementation takes, for every unused bucket, space as big as
the key-type. Usually between half and two-thirds of the buckets are
empty.

The doubling method used by dense\_hash\_set tends to work poorly
with most memory allocators. This is because memory allocators tend
to have memory 'buckets' which are a power of two. Since each
doubling of a dense\_hash\_set doubles the memory use, a single
hashtable doubling will require a new memory 'bucket' from the memory
allocator, leaving the old bucket stranded as fragmented memory.
Hence, it's not recommended this data structure be used with many
inserts in memory-constrained situations.

You can also look at some specific performance numbers.

---

## dense\_hash\_map

dense\_hash\_map is identical to dense\_hash\_set except for what values
are stored in each table entry.

---


Craig Silverstein  
Thu Jan 6 20:15:42 PST 2005
